# The arginine methyltransferase PRMT7 promotes extravasation of monocytes resulting in tissue injury in COPD

**DOI:** 10.1038/s41467-022-28809-4

**Published:** 2022-03-14

**Authors:** Gizem Günes Günsel, Thomas M. Conlon, Aicha Jeridi, Rinho Kim, Zeynep Ertüz, Niklas J. Lang, Meshal Ansari, Mariia Novikova, Dongsheng Jiang, Maximilian Strunz, Mariia Gaianova, Christine Hollauer, Christina Gabriel, Ilias Angelidis, Sebastian Doll, Jeanine C. Pestoni, Stephanie L. Edelmann, Marlene Sophia Kohlhepp, Adrien Guillot, Kevin Bassler, Hannelore P. Van Eeckhoutte, Özgecan Kayalar, Nur Konyalilar, Tamara Kanashova, Sophie Rodius, Carolina Ballester-López, Carlos M. Genes Robles, Natalia Smirnova, Markus Rehberg, Charu Agarwal, Ioanna Krikki, Benoit Piavaux, Stijn E. Verleden, Bart Vanaudenaerde, Melanie Königshoff, Gunnar Dittmar, Ken R. Bracke, Joachim L. Schultze, Henrik Watz, Oliver Eickelberg, Tobias Stoeger, Gerald Burgstaller, Frank Tacke, Vigo Heissmeyer, Yuval Rinkevich, Hasan Bayram, Herbert B. Schiller, Marcus Conrad, Robert Schneider, Ali Önder Yildirim

**Affiliations:** 1 Institute of Lung Health and Immunity (LHI), Comprehensive Pneumology Center (CPC), Helmholtz Munich, Member of the German Center for Lung Research (DZL), 85764 Munich, Germany; 2 Institute of Functional Epigenetics, Helmholtz Munich, 85764 Munich, Germany; 3 Institute of Computational Biology, Helmholtz Munich, 85764 Munich, Germany; 4 Institute of Metabolism and Cell Death, Helmholtz Munich, 85764 Munich, Germany; 5 Pirogov Russian National Research Medical University, Laboratory of Experimental Oncology, Ostrovityanova 1, Moscow, 117997 Russia; 6 Federal Center of Brain Research and Neurotechnologies, Federal Medical Biological Agency, Ostrovityanova1 bldg 10, 117997 Moscow, Russia; 7 Research Unit Molecular Immune Regulation, Helmholtz Munich, 81377 Munich, Germany; 8 Department of Hepatology & Gastroenterology, Charité - Universitätsmedizin Berlin, Campus Virchow-Klinikum (CVK) and Campus Charité Mitte (CCM), 13353 Berlin, Germany; 9 Department for Genomics & Immunoregulation, LIMES-Institute, University of Bonn, 53115 Bonn, Germany; 10 aimed analytics, 53121 Bonn, Germany; 11 Laboratory for Translational Research in Obstructive Pulmonary Diseases, Department of Respiratory Medicine, Ghent University, University Hospital Ghent, 9000 Ghent, Belgium; 12 Koç University Research Center for Translational Medicine (KUTTAM), 34010 Istanbul, Turkey; 13 Max-Delbrück Center for Molecular Medicine, 13125 Berlin, Germany; 14 Proteomics of cellular signalling, Luxembourg Institute of Health, 1272 Strassen, Luxembourg; 15 Division of Pulmonary Sciences and Critical Care Medicine, University of Colorado, Denver, CO 80045 USA; 16 Czech Centre for Phenogenomics, Institute of Molecular Genetics of the Czech Academy of Sciences, 25242 Vestec, Czech Republic; 17 Division of Pneumology, KU Leuven, 3000 Leuven, Belgium; 18 Antwerp Surgical Training, Anatomy and Research Centre, University of Antwerp, 2650 Edegem, Belgium; 19 Division of Pulmonary, Allergy and Critical Care Medicine, Department of Medicine, University of Pittsburgh, Pittsburgh, PA 15261 USA; 20 Faculty of Science, Technology and Medicine, University of Luxembourg, L-4365 Esch-sur-Alzette, Luxembourg; 21 Deutsches Zentrum für Neurodegenerative Erkrankungen (DZNE) e.V., PRECISE Platform for Single Cell Genomics and Epigenomics at DZNE and the University of Bonn, 53115 Bonn, Germany; 22 Pulmonary Research Institute at LungenClinic Grosshansdorf, Airway Research Center North (ARCN), German Center for Lung Research (DZL), 22927 Grosshansdorf, Germany; 23 Institute for Immunology, Biomedical Center, Faculty of Medicine, Ludwig-Maximilians-Universität in Munich, 82152 Planegg-Martinsried, Germany

**Keywords:** Immune cell death, Chronic obstructive pulmonary disease, Monocytes and macrophages, Histone post-translational modifications

## Abstract

Extravasation of monocytes into tissue and to the site of injury is a fundamental immunological process, which requires rapid responses via post translational modifications (PTM) of proteins. Protein arginine methyltransferase 7 (PRMT7) is an epigenetic factor that has the capacity to mono-methylate histones on arginine residues. Here we show that in chronic obstructive pulmonary disease (COPD) patients, PRMT7 expression is elevated in the lung tissue and localized to the macrophages. In mouse models of COPD, lung fibrosis and skin injury, reduced expression of PRMT7 associates with decreased recruitment of monocytes to the site of injury and hence less severe symptoms. Mechanistically, activation of NF-κB/RelA in monocytes induces PRMT7 transcription and consequential mono-methylation of histones at the regulatory elements of RAP1A, which leads to increased transcription of this gene that is responsible for adhesion and migration of monocytes. Persistent monocyte-derived macrophage accumulation leads to ALOX5 over-expression and accumulation of its metabolite LTB4, which triggers expression of ACSL4 a ferroptosis promoting gene in lung epithelial cells. Conclusively, inhibition of arginine mono-methylation might offer targeted intervention in monocyte-driven inflammatory conditions that lead to extensive tissue damage if left untreated.

## Abstract (web summary)

Chronic obstructive pulmonary disease is a progressive and incurable chronic condition that involves accumulation of inflammatory macrophages in the lung tissue. Authors here show in mouse models of lung disease that PRMT7, a protein arginine methyltransferase, is an important regulator of recruitment and the pro-inflammatory phenotype of macrophages.

## Introduction

Chronic obstructive pulmonary disease (COPD) is a highly prevalent inflammatory disease of the airways and alveoli that results in irreversible and progressive airflow limitation due to small airway disease and lung tissue injury (emphysema) driven by alveolar epithelial cell death^1–4^. Long-term exposure to toxic gases, in particular cigarette smoke (CS), results in complex inflammatory processes, with predominantly an increased number of monocyte-derived macrophages in the lung parenchyma and airways orchestrating immunopathogenesis^5^. However, the precise molecular mechanisms underpinning these responses remain to be elucidated, and require renewed attention, especially as only a percentage of smokers develop COPD and individual disease manifestations, as well as clinical courses, remain highly variable irrespective of therapy^3^.

The posttranslational ligation of methyl groups to arginine residues in proteins, carried out by protein arginine methyltransferases (PRMT) in mammals, is a powerful posttranslational modification (PTM) that can both epigenetically regulate transcription and modulate signal transduction^6^. Mammals express nine distinct family members that catalyze the transfer of a methyl group from *S*-adenosylmethionine to the guanidine nitrogen atoms of arginine, which are categorized into three types^7^. Type I generates asymmetric di-methylarginine (ADMA), type II generates symmetric di-methylarginine (SDMA) and type III is unique in its ability to only generate mono-methylarginine (MMA). Remarkably, among these PRMT7 is the only type III enzyme, as all other PRMTs catalyze MMA which can be followed by the addition of a second methyl group^8^. However, the contribution of PRMT7 to the pathogenesis of inflammatory diseases, particularly in the lung, and its therapeutic potential remain enigmatic.

In the present study, we demonstrate that PRMT7 expression is increased in the lung of COPD patients and positively correlates with lung tissue injury. Reduced expression of PRMT7 in COPD mouse models protects against disease development, due to decreased recruitment of pro-inflammatory monocytes to the site of injury. Expression of PRMT7 in monocytes is regulated through the activation of NF-kB/RelA and results in induced mono-methylation of histones, which crucially regulates RAP1A expression and subsequent adhesion and migration ability. In COPD mouse models with persistent monocyte-derived macrophage accumulation, pro-inflammatory macrophages upregulate ALOX5 and release LTB4 resulting in induced ACSL4 expression in lung alveolar epithelial cells causing enhanced lipid peroxidation and sensitization towards ferroptotic cell death. Collectively, these studies indicate that targeting PRMT7 may offer therapeutic potential against monocyte-driven inflammatory conditions.

## Results

### Arginine methylation impacts COPD pathogenesis

**Fig. 1:**
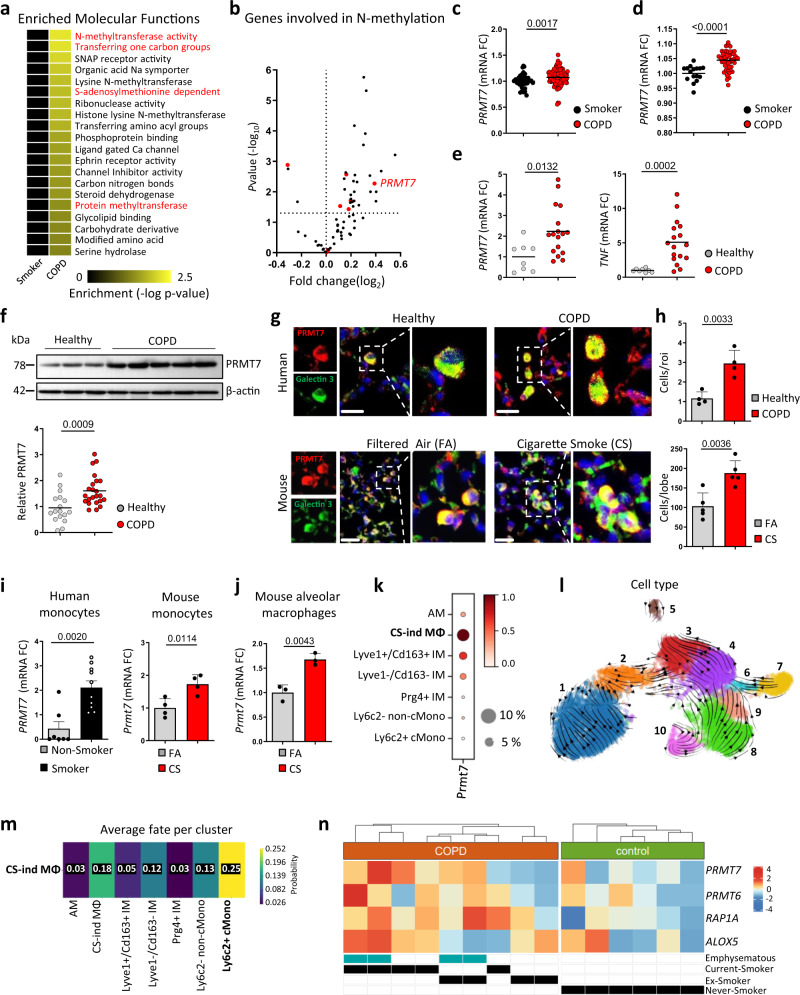
PRMT7 expression is increased in COPD lungs and localized to macrophages. **a** Heat map of the most significantly enriched gene lists in the lungs of COPD patients following gene set enrichment analysis (GSEA) of the GO molecular function set on publicly available array data from lung tissue (GSE76925) of healthy smokers (*n* = 40) v COPD patients (*n* = 111). Nominal *P* value generated by the GSEA software of the enrichment score relative to the null distribution^92^ shown. **b** Expression of genes involved in *N*-methyltransferase activity in COPD patients relative to healthy smokers taken from GSE76925, relative fold change, and *P* value calculated using the GEO2R interactive web tool running limma R. PRMT genes highlighted in red. **c**, **d** Expression of *PRMT7*, in the lungs of healthy smokers and COPD patients, individuals are shown, expression relative to healthy smokers, taken from GSE76925 (*n* = 40 smokers and *n* = 111 COPD patients) (**c**) and GSE27597 (*n* = 16 smokers and *n* = 48 COPD patients) (**d**). **e** qPCR analysis of *PRMT7* and *TNF* expression in human lung core biopsies from healthy controls (*n* = 8) and COPD patients (*n* = 18) relative to controls. **f** Western blot analysis of PRMT7 expression in lung core biopsies from healthy (*n* = 17) and COPD patients (*n* = 23), normalized to β-actin and shown relative to controls. **g** Representative images of immunofluorescence analysis for PRMT7 (Red) and the macrophage marker Galectin 3 (Green) in sections from core biopsies of healthy and COPD human lung (*n* = 4, scale bar 20 μm) and sections from filtered air (FA) and cigarette smoke (CS) mouse lung (*n* = 5, scale bar 25 μm). **h** Quantification of double-positive cells from (**g**). **i**, **j** mRNA expression levels of *PRMT7* determined by qPCR in human monocytes isolated from blood of non-smokers (*n* = 7) vs smokers (*n* = 10) (**i**) and circulating monocytes (*n* = 4 for FA and CS) (**i**) and alveolar macrophages (*n* = 4 for FA and *n* = 3 for CS) (**j**) from B6 mice exposed to FA or CS for 3 days, relative to controls. **k**–**m** scRNA-Seq (Drop-Seq) analysis on the lungs of mice following exposure to FA (*n* = 3) and CS for 2 (*n* = 5) and 4 months (*n* = 5). AM alveolar macrophages, CS-ind MØ CS-induced macrophages, IM interstitial macrophages, cMono classical monocytes. **k** Dot blot depicting *Prmt7* expression (log-transformed, normalized UMI counts) and percentage of cells positive for *Prmt7* in the myeloid cell compartment. **l** RNA velocity analysis of the myeloid compartment. (1) AM; (2) CS-ind MØ; (3) Lyve1 + /Cd163 + IM; (4) Lyve1−/Cd163− IM; (5) Prg4 + IM; (6) Ly6c2 + cMono; (7) Ly6c2- non-cMono; (8) Cd103 + /Clec9a + cDC; (9) Cd209 + /Cd11b + cDC; (10) Fscn1 + DC. **m** Fate probability mapping towards the CS-ind MØ population utilizing CellRank. **n** Heat map of gene expressions of *PRMT6/7*, *RAP1A*, and *ALOX5* in monocyte-like macrophages obtained from scRNA-Seq data of bronchoalveolar lavage from COPD patients (*n* = 9) and healthy controls (*n* = 6). Mean gene expression per donor is shown as a z-transformed value (across all donors). Data shown mean ± SD and individual patients or mice (**c**–**f** and **h**–**j**), *P* values shown in charts determined by two-tailed Mann–Whitney test (**c**–**f**), unpaired two-tailed Student’s *t*-test (**h**–**j**). Source data are provided as a Source Data file.

To unravel the mechanisms why only subsets of smokers develop COPD, we set out to elucidate the molecular networks and pathways that may underlie COPD development in these patients. We first undertook unbiased gene set enrichment analysis (GSEA) on transcriptomics data obtained from the lungs of 111 COPD patients with smoking history and 40 smoker controls (Supplementary Fig. 1a) (GSE76925)^9^. This analysis revealed multiple methyltransferase-related gene sets to be significantly enriched within the Gene Ontology (GO) Molecular Function collection, clustering under catalytic activity (Fig. 1a; Supplementary Fig. 1b; and Supplementary Data File 1). The top five ranked genes from the most enriched pathway, *N*-methyltransferase activity (Supplementary Fig. 1c and Supplementary Data File 2), were *SETD4, GNMT*, *EZH2*, and *PNMT* (Supplementary Fig. 1d) and *PRMT7* (Fig. 1b, c). An increase in *PRMT7* expression was the most—dysregulated *PRMT* gene, correlated with CS exposure (Supplementary Fig. 1e) and was validated by the established COPD marker genes *MMP9*, *SPP1*, and *POSTN* (Supplementary Fig. 1d). This was supported by determining increased *PRMT7* expression in the lungs of a further independent cohort of COPD patients (GSE27597) compared to smoker controls (Fig. 1d) but not *SETD4, GNMT*, *EZH2*, or *PNMT* (Supplementary Fig. 1f), where *PRMT7* expression positively correlated with lung tissue injury-induced airspace enlargement (Supplementary Fig. 1g). Moreover, analysis of the Genome-Wide Association Study (GWAS) Central database^10^ identified three single nucleotide polymorphisms (SNP) in the *PRMT7* gene, from a genome-wide association study composed of over 48,000 individuals^11^, to be associated with an altered lung function (−log*P* > 2, Supplementary Fig. 2a). Interestingly, the Genotype-Tissue Expression (GTEx) Portal^12^ revealed these three SNPs to be expression quantitative trait loci for *PRMT7* in the lung and whole blood (Supplementary Fig. 2b). Similarly, we confirmed an increase of PRMT7 in the lungs of a third COPD patient cohort, but not *SETD4, GNMT*, *EZH2*, or *PNMT* (Fig. 1e, f and Supplementary Fig. 1h), which was validated by increased inflammatory cytokines *TNF* (Fig. 1e), *CXCL1 CCL2*, *IL8*, and *IL6* (Supplementary Fig. 1h). By contrast, expression of PRMT1 and CARM1 remained similar between healthy and COPD patients, while PRMT5 slightly increased (Supplementary Fig. 1h), which are also involved in a variety of lung disorders^13–15^. PRMT6 was recently described to also regulate inflammation in an animal model of emphysema^16^, while PRMT6 expression increased in the lungs of COPD patients from one cohort (GSE76925) (Supplementary Fig. 1i, its expression did not significantly change in the two other cohorts (Supplementary Fig. 1h, j, k) or mice exposed to CS (Supplementary Fig. 1l). Finally, to delineate further the contribution of PRMT7 to COPD, we have analysed lung tissue from 118 participants with different smoking and disease status. There was a significant increase in *PRMT7* mRNA expression in COPD global initiative for chronic obstructive lung disease (GOLD) stage III-IV patients, compared to never-smokers, ex-smokers without COPD, and ex-smokers with COPD GOLD II (Supplementary Fig. 2c). Furthermore, in patients at the same disease stage (GOLD II), there was increased expression of PRMT7 in those patients that continued to smoke compared to those that stop smoking (Supplementary Fig. 2c). In addition, there were significant correlations between *PRMT7* mRNA expression and disease severity within COPD patients (Supplementary Fig. 2d).

In the lungs of COPD patients and mice exposed to CS, PRMT7 predominantly localized to macrophages (Fig. 1g, h), supported by the Human Protein Atlas database (https://www.proteinatlas.org)^17^ (Supplementary Fig. 2e). Crucially, circulating monocytes isolated from both humans and mice exposed to CS demonstrated increased *PRMT7* expression (Fig. 1i). Similarly, primary macrophages isolated from the lungs and a macrophage cell line exposed to CS, robustly increased expression of *Prmt7* (Fig. 1j and Supplementary Fig. 2h), but not *Prmt1* and *Carm1*, while primary macrophages isolated from the lungs of mice exposed to CS also increased expression of *Prmt5* (Supplementary Fig. 2f–h).

To further characterize the cellular expression of PRMT7, we investigated single-cell RNA sequencing (scRNA-seq) on the lungs of mice exposed to chronic CS. This identified a population of macrophages specific to cigarette smoke exposure (Supplementary Fig. 3a), referred to here as CS-induced macrophages, that co-express *Gpnmb*, *Lgals3*, and *Spp1* (Supplementary Fig. 3b). CS-induced macrophages had the greatest expression of *Prmt7* across all myeloid cells (Fig. 1k), second only to plasma cells across all 29 lung cell types stratified (Supplementary Fig. 3c). RNA velocity analysis based on splicing ratios and fate probability mapping utilizing CellRank^18^, a method that combines the robustness of similarity-based trajectory inference methods with directional information given by RNA velocity, revealed that CS-induced macrophages originated from classical inflammatory monocytes and differentiate toward a monocyte-derived alveolar macrophage phenotype (Fig. 1l, m). Inline, there was an increase in the percentage of monocyte-like macrophages in the bronchoalveolar lavage of COPD patients compared to healthy controls following scRNA-seq analysis (Supplementary Fig. 3d), with greater *PRMT7* expression in the monocyte-like macrophages of COPD patients, particularly in current smokers with emphysema, compared to controls (Fig. 1n and Supplementary Fig. 3e).

### NF-κB regulates PRMT7 in myeloid cells

To elucidate the mechanism enhancing PRMT7 expression, we first performed the assay for transposase-accessible chromatin using sequencing (ATAC-Seq) followed by promoter analysis using the Cistrome Data Browser^19^. ATAC-Seq reveals open chromatin at the transcriptional start site (TSS) overlapping with the active marks H3K27ac and H3K4me3. Further, ChIP-Seq analysis of the genomic binding regions of transcription factors crucial to inflammatory responses and myeloid development^20^ demonstrated that RelA was enriched on this promoter region of *Prmt7* (Supplementary Fig. 4a), rather than RelB, AP-1 (JUN/FOS), IRF1/3, and CEBPA/B. In support, JASPAR^21^ analysis of the ATAC-Seq track across the TSS of *Prmt7* revealed the presence of NFKB1/RelA consensus motifs (Supplementary Fig. 4b). Amongst other triggers, RelA translocates to the nucleus in response to inflammatory TLR4-NF-κB signaling^22^. *Prmt7* gene expression in mouse monocytes was increased significantly upon TLR4 stimulation with LPS, accompanied by the induction of inflammatory genes *Tnf*, *Il6*, and *Il1b* (Supplementary Fig. 4c, d). This was blunted by using an inhibitor of IκB-α phosphorylation, BAY11-7082^23^ (Supplementary Fig. 4c). Accordingly, a specific inhibitor of the nuclear translocation of RelA, JSH-23^24^, also reduced the effect of LPS stimulation on *Prmt7* expression (Supplementary Fig. 4d). Importantly, this correlated with both PRMT7 protein expression and functional enzymatic activity as demonstrated by levels of MMA (Supplementary Fig. 4e). In sum, monocytes strongly increase PRMT7 expression upon RelA-mediated NF-κB inflammatory signaling.

### PRMT7 is crucial for cellular migration and adhesion

**Fig. 2:**
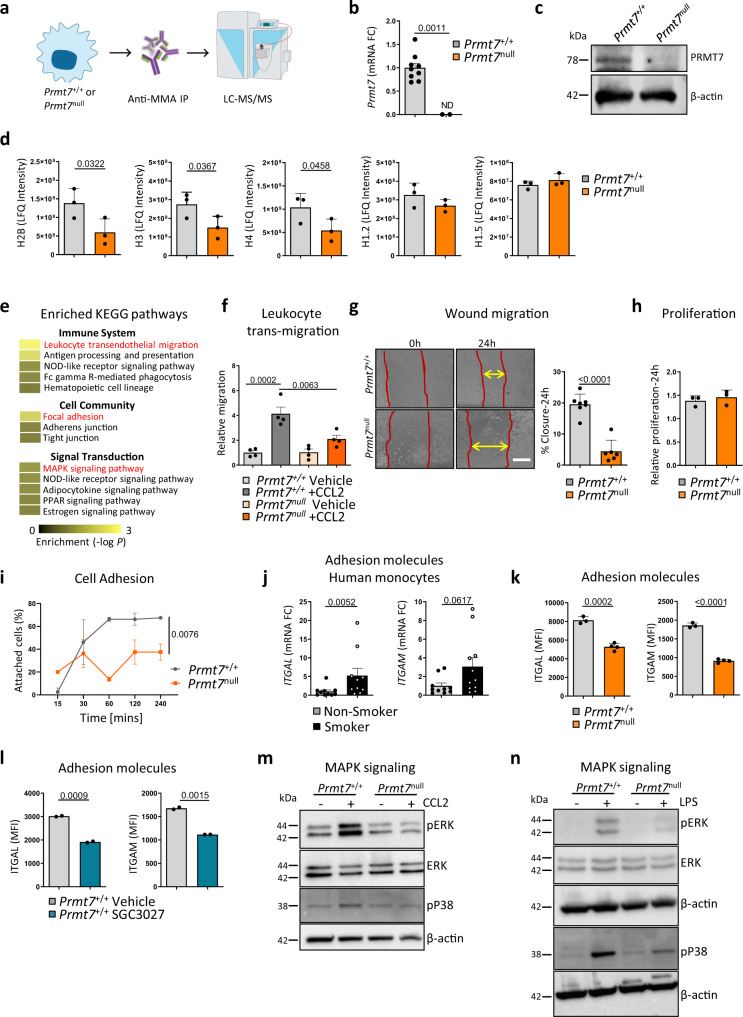
Loss of PRMT7 results in impaired migration, focal adhesion, and MAPK signaling. **a**–**d** Mono-methylated proteins were immunoprecipitated by anti-mono-methylarginine antibody from whole-cell lysates of *Prmt7*^+/+^ and *Prmt7*^null^ MH-S macrophage cells and analyzed by LC-MS/MS proteomics (*n* = 3 pull-downs per cell line). **a** Schematic representation of the experiment. **b** mRNA expression level of *Prmt7* determined by qPCR in *Prmt7*^+/+^ and *Prmt7*^null^ MH-S macrophage cells generated by CRISPR/Cas9-mediated targeting of *Prmt7* (*n* = 9 replicates per cell line, ND not detectable). **c** Western blot analysis of PRMT7 expression in *Prmt7*^+/+^ and *Prmt7*^null^ MH-S cells (repeated two times). **d** Immunoprecipitation intensity of core histones H2B, H3, H4, and linker H1.2 and H1.5 (Individual pull-downs shown). **e** Top enriched KEGG pathways under the immune system, cell community, and signal transduction following InCroMAP analysis of the differentially pulled down proteins with less abundance in *Prmt7*^null^ compared to *Prmt7*^+/+^ MH-S cells (FC <−1.6). *P* values were calculated using a hypergeometric test embedded in the InCroMAP software, no testing for multiple correction. **f** The fold change of trans-well migrating *Prmt7*^+/+^ and *Prmt7*^null^ MH-S macrophage cells in 24 h towards serum-free (SF) medium ± 100 ng/ml CCL2 (*n* = 4, repeated two times). **g** Wound migration assay in *Prmt7*^+/+^ (*n* = 7) and *Prmt7*^null^ (*n* = 6) MH-S cells grown to confluence and scratched. Representative images at 0 and 24 h post scratch plus the percentage wound closure at 24 h (repeated twice). Scale bar, 200 μm. **h** WST assay of *Prmt7*^+/+^ (*n* = 3) and *Prmt7*^null^ (*n* = 3) MH-S cells at 24 h. **i** Percentage of attached *Prmt7*^+/+^ and *Prmt7*^null^ MH-S cells at the time points indicated post-seeding (*n* = 2 per cell line, repeated twice). **j** mRNA expression levels of *ITGAL* and *ITGAM* determined by qPCR in the monocytes of smokers (*n* = 11) and non-smokers (*n* = 10) isolated from the peripheral blood. **k** MFI of ITGAL and ITGAM surface expression as determined by flow cytometry in *Prmt7*^+/+^ (*n* = 3) and *Prmt7*^null^ (*n* = 4) MH-S cells. **l** MFI of ITGAL and ITGAM surface expression as determined by flow cytometry in WT MH-S cells incubated with 5 μM SGC3027 (PRMT7 inhibitor) for 24 h and analyzed (*n* = 2). **m** Western blot analysis of phosphorylated ERK and p38 in *Prmt7*^+/+^ and *Prmt7*^null^ MH-S cells incubated for 30 min with 100 ng/ml CCL2 (repeated independently two times). **n** Western blot analysis of phosphorylated ERK and p38 in *Prmt7*^+/+^ and *Prmt7*^null^ MH-S cells incubated for 15 min with 1 μg/ml LPS (repeated independently two times). Data shown mean ± SD, *P* values shown in charts determined by one-way ANOVA Bonferroni’s multiple comparisons test (**f**, **i**), unpaired two-tailed Student’s *t*-test (**b**, **d**, **g**, **j**–**l**). Source data are provided as a Source Data file.

Next, we studied the function of PRMT7 in myeloid cells and performed proteomic analysis of enzymatic activity (MMA) in *Prmt7*^+/+^ versus *Prmt7*^null^ cells (Fig. 2a and Supplementary Data Files 3, 4). These were generated by CRISPR/Cas9 knockout of *Prmt7* in the MH-S macrophage cell line (Supplementary Fig. 5a–c), to enable the formation of a stable macrophage line with complete loss of PRMT7. The functional knockout of PRMT7 was validated by depletion of mRNA (Fig. 2b), protein (Fig. 2c), and reduced MMA (Supplementary Fig. 5d), with no reciprocal regulation of PRMT6 (Supplementary Fig. 5e, f). The proteomic analysis revealed that levels of the immune-precipitated mono-methylated core histones H2B, H3, and H4 were lower in *Prmt7*^null^ compared to *Prmt7*^+/+^ cells (Fig. 2d), while no difference was observed for the linker histone H1 proteins (Fig. 2d).

Enrichment analysis of proteomics highlighted that leukocyte transendothelial migration of the KEGG immune system pathways was strongly impaired in *Prmt7*^null^ cells, along with focal adhesion and MAPK signaling of the KEGG cell community and signal transduction pathways, respectively (Fig. 2e and Supplementary Data File 5). Impaired migratory ability of *Prmt7*^null^ cells was confirmed in trans-well leukocyte and wound migration assays (Fig. 2f-g), with no effect on cellular proliferation (Fig. 2h). Further, *Prmt7*^null^ cells also demonstrated diminished adhesion ability (Fig. 2i). Integrin activation is a crucial step in leukocyte adhesion and subsequent transendothelial migration^25,26^, as observed by increased expression of ITGAL and ITGAM in circulating monocytes from humans (Fig. 2j). In keeping, reduced surface ITGAL (CD11a) and ITGAM (CD11b) was demonstrated on *Prmt7*^null^ (Fig. 2k) and *Prmt7*^+/+^ cells treated with SGC3027 (Fig. 2l), a PRMT7 inhibitor^27^ (Supplementary Fig. 5g). To provide further support in an additional cell line, which originated from monocytes, we generated similarly a CRISPR/Cas9 stable knockout of *Prmt7* in RAW264.7 cells (Supplementary Fig. 5h, i). Similar to the MH-S cells, this showed a clear reduction in MMA, accompanied by reduced surface ITGAL (Supplementary Fig. 5j, k), with no reciprocal regulation of PRMT6 (Supplementary Fig. 5l, m**)**. In addition, stimulation with CCL2 confirmed that MAPK signaling was impaired in *Prmt7*^null^ cells (Fig. 2m). In support, stimulating with LPS, again demonstrated reduced phosphorylated ERK and p38 in *Prmt7*^null^ cells (Fig. 2n). Taken all together this data thus suggests that PRMT7 is crucial for adhesion and subsequent migratory ability.

### PRMT7 regulates *Rap1a* expression through histone methylation

**Fig. 3:**
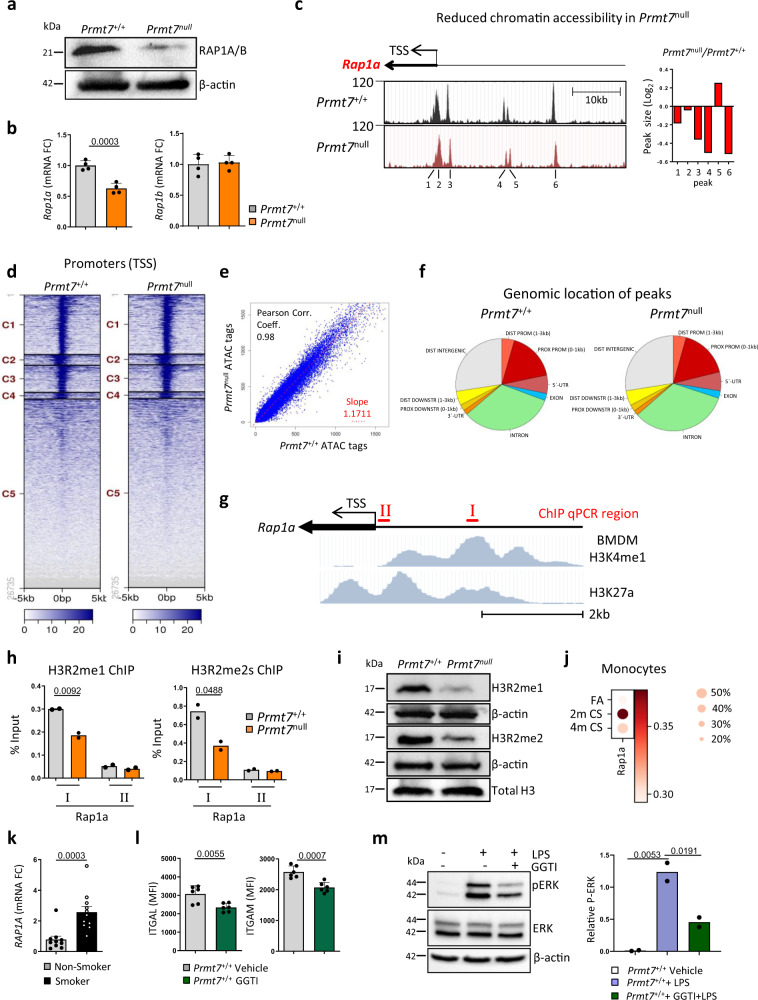
PRMT7 targets RAP1A expression through histone methylation. **a** Western blot analysis of RAP1A/RAP1B expression in *Prmt7*^+/+^ and *Prmt7*^null^ MH-S cells (repeated twice). **b** mRNA expression level of *Rap1a* and *Rap1b* determined by qPCR in *Prmt7*^+/+^ and *Prmt7*^null^ MH-S macrophage cells (*n* = 4 replicates per cell line). **c**–**f** ATAC-Seq analysis of *Prmt7*^+/+^ v *Prmt7*^null^ MH-S cells. **c** ATAC-seq signal enrichment peaks around the transcription start site (TSS) of the *Rap1a* gene in *Prmt7*^+/+^ and *Prmt7*^null^ MH-S cells and difference in peak height across the two lines. **d** Heat map of tag distributions across TSSs for *Prmt7*^+/+^ and *Prmt7*^null^ MH-S cells. **e** Peak correlation scatter plot. **f** Pie chart showing the genomic distribution of accessible regions in *Prmt7*^+/+^ and *Prmt7*^null^ MH-S cells. **g** Schematic representation of the location of the *Rap1a* regions targeted for qPCR following ChIP, plus H3K4me1 and H3K27a enrichment in BMDM (from UCSC genome browser Track accessions: wgEncodeEM002658 and wgEncodeEM002657). **h** Enrichment of H3R2 mono and dimethylation at *Rap1a* regions in *Prmt7*^+/+^ and *Prmt7*^null^ MH-S by qPCR following ChIP (*n* = 2 per cell line). **i** Western blot analysis of mono and dimethylation of H3R2 in *Prmt7*^+/+^ and *Prmt7*^null^ MH-S cells (repeated twice). **j** Dot blot depicting *Rap1a* expression (log-transformed, normalized UMI counts) and percentage of cells positive for *Rap1a* in monocytes from mouse lung single-cell RNA-seq data following exposure to FA (*n* = 3) and CS for 2 (*n* = 5) and 4 months (*n* = 5). **k** mRNA expression levels of *RAP1A* determined by qPCR in the monocytes of smokers (*n* = 10) and non-smokers (*n* = 11) isolated from the peripheral blood. **l** MFI of ITGAL and ITGAM surface expression as determined by flow cytometry in *Prmt7*^+/+^ MH-S cells incubated with 20 μM GGTI (RAP1 inhibitor) for 2 h and analysed 6 h later (*n* = 2, repeated three times). **m** Western blot analysis of phosphorylated ERK in WT MH-S cells pretreated with the RAP1 inhibitor GGTI (20 μM) for 2 h and incubated for 15 min with 1 μg/ml LPS. Quantification relative to actin shown (repeated two times). Data shown mean ± SD, *P* values shown in charts determined by unpaired two-tailed Student’s *t*-test (**b**, **h**, **k**, **l**), one-way ANOVA Bonferroni’s multiple comparisons test (**m**). Source data are provided as a Source Data file.

By overlapping the dysregulated pathways of leukocyte transendothelial migration, focal adhesion, and MAPK signaling we detected one common denominator, RAP1A/B, which is a small G protein of the Ras superfamily (Supplementary Fig. 6a, b). The MMA-pull down of RAP1A/B suggests a potential PRMT7 substrate, which we examined by undertaking proximity ligation assays (PLA). This revealed a clear co-localization of MMA with control histone H3 and RAP1A/B in macrophage cells which is lost in *Prmt7*^null^ cells (Supplementary Fig. 6c, d). Remarkably, protein analysis also revealed reduced levels of RAP1A/B in whole lysates from *Prmt7*^null^ cells (Fig. 3a). Since the RAP antibody does not discriminate between RAP1A/B, gene expression analysis was used showing *Rap1a* and not *Rap1b* was indeed reduced (Fig. 3b), which was also validated in the *Prmt7* knockout RAW264.7 cells (Supplementary Fig. 7a). To understand how *Rap1a* is regulated by PRMT7, we first performed ATAC-Seq analysis which showed the accessibility at the *Rap1a* gene was reduced in five regions in *Prmt7*^null^ cells (Fig. 3c). Interestingly, there were no major global changes in chromatin accessibility in *Prmt7*^null^ cells (Fig. 3d–f). To further determine the transcriptional regulator role of PRMT7 for *Rap1a*, we undertook ChIP-qPCR on chromatin isolated from *Prmt7*^+/+^ and *Prmt7*^null^ cells. We amplified a region upstream of the TSS (primer-I) that is a putative enhancer for *Rap1a* in monocyte-derived macrophages as determined by enrichment of H3K4me1 and H3K27a, and outside of this region (primer-II) (Fig. 3g). Notably, enrichment of H3R2me1 and the active histone mark H3R2me2s was greatest within the region of primer-I and was reduced in *Prmt7*^null^ cells (Fig. 3h). In accordance, both H3R2me1 and -me2s were reduced in *Prmt7*^null^ cells (Fig. 3i).

To delineate the role of MMA imprinted by PRMT7 from reductions in dimethylation, we exposed macrophage cells to increasing concentrations of the well-established Type I inhibitor MS023 which blocks Rme2a^28^ and the PRMT5 inhibitor EPZ015666 to block Rme2s^29^, in comparison to the PRMT7 inhibitor SGC30273^27^. Inhibition of PRMT7 with SGC3027 again caused a significant reduction in the levels of MMA, which was accompanied by reduced expression of RAP1A, ITGAL, and ITGAM both at the protein and transcript level (Supplementary Fig. 7b–e). Crucially, direct inhibition of the type I PRMTs (Supplementary Fig. 7g) or the type II PRMT5 (Supplementary Fig. 7h), did not lead to reduced expression of RAP1A, ITGAL, or ITGAM at the transcript level (Supplementary Fig. 7f) and surface expression (Supplementary Fig. 7j). Interestingly, inhibition of the type I PRMTs with MS023 leads to increased mono-methylation (Supplementary Fig. 7i) as described previously^28^, which resulted in increased surface expression of both ITGAL and ITGAM (Supplementary Fig. 7j). Taken altogether this strongly suggests that PRMT7 mediated mono-methylation is a crucial regulator for the expression of key transendothelial migratory proteins RAP1A, ITGAL, and ITGAM.

RAP1A is activated by GTP binding and plays a pivotal role in cellular adhesion and migration by enhancing integrin-dependent signaling and regulating MAPK activity^30,31^ (Supplementary Fig. 6b). Single-cell RNA-seq data revealed increased *Rap1a* expression in monocytes from the lungs of chronic CS-exposed mice (Fig. 3j), consistent with increased expression of *RAP1A* levels in circulating monocytes from the peripheral blood of smokers (Fig. 3k) and monocyte-like macrophages in the bronchoalveolar lavage of COPD patients as determined by scRNA-seq (Fig. 1n). Moreover, macrophages isolated from *Rap1a*^−/−^ mice demonstrated decreased adhesion ability and impaired migration towards chemokines^32^. Consistently, inhibiting the activity of RAP1 with GGTI^33^ in *Prmt7*^+/+^ cells reduced the surface expression of the adhesion molecules ITGAL (CD11a) and ITGAM (CD11b) (Fig. 3l), comparable to that seen in *Prmt7*^null^ cells (Fig. 2k). Additionally, GGTI inhibition of RAP1 in *Prmt7*^+/+^ cells impaired the phosphorylation of ERK post LPS stimulation (Fig. 3m), similar to that observed in *Prmt7*^null^ cells (Fig. 2n).

### Monocyte transendothelial migration is regulated by PRMT7

**Fig. 4:**
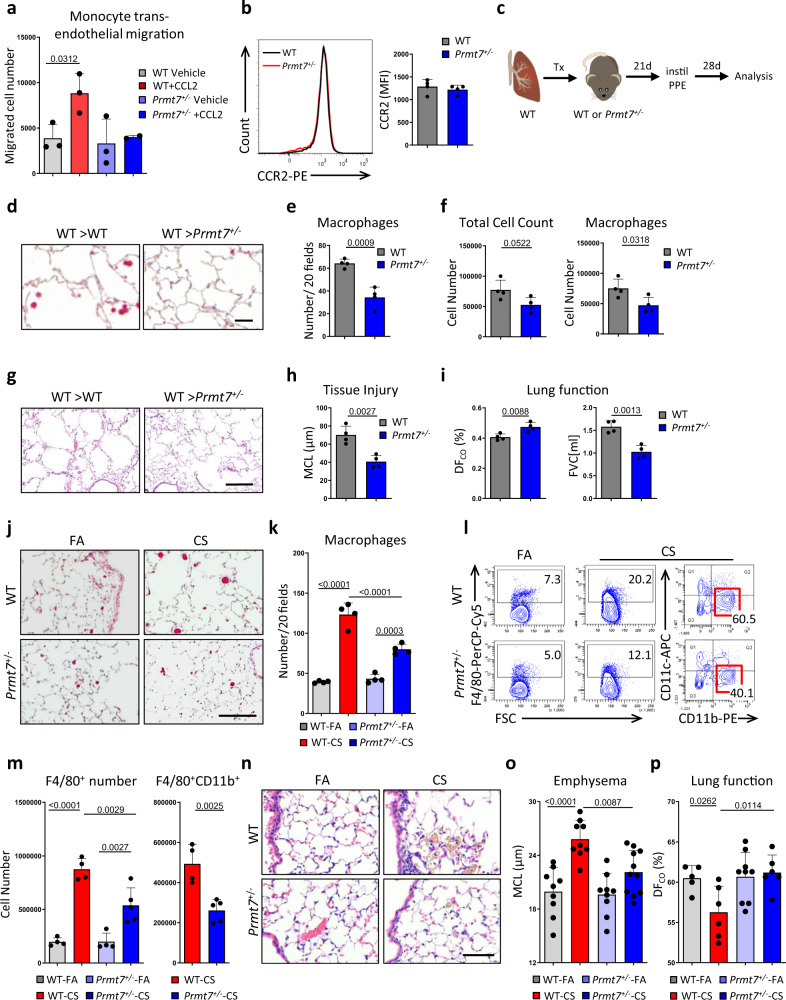
PRMT7 regulates monocyte recruitment. **a** The number of trans-well migrating monocytes isolated from the bone marrow of WT and *Prmt7*^+/−^ mice migrating in 4 h through TNF (10 ng/ml) activated SVECs towards serum-free (SF) medium ± 100 ng/ml CCL2 (*n* = 3 independent experiments). **b** Flow cytometry plot of CCR2 expression and MFI of CCR2 in monocytes isolated from the bone marrow of WT and *Prmt7*^+/−^ mice (*n* = 4 per genotype). **c**–**i** Left lungs from wild-type (WT) mice were orthotopically transplanted into WT or *Prmt7*^+/−^ recipients, left for 3 weeks to recover from surgery, and then exposed to a single dose of porcine pancreatic elastase (PPE) 40 U/Kg and analyzed 28 days later (*n* = 4 per group). **c** Schematic representation of the experiment. **d** Representative images of immunohistochemical analysis for Galectin 3 positive macrophages (red signal, hematoxylin counterstained, scale bar 50 μm) in sections from the transplanted lungs. **e** Quantification of macrophage number in lung sections from (**d**) given as the number of positive cells per 20 random fields of view. **f** Bronchoalveolar lavage fluid total and macrophage cell count. **g** Representative images of Hematoxylin and Eosin (H&E)-stained sections from the transplanted lungs (scale bar 200 μm). **h** Quantification of airspace enlargement as mean chord length (MCL) in lung sections from (**g**). **i** Lung function is measured as the diffusing capacity of carbon monoxide and forced vital capacity (FVC). **j**–**p** WT and *Prmt7*^+/−^ mice were exposed to FA or cigarette smoke (CS) for 4 months (*n* = 4–8 per group, repeated twice). **j** Representative images of immunohistochemical analysis for Galectin 3 positive macrophages (red signal, hematoxylin counterstained, scale bar 100 μm) in lung sections. **k** Quantification of macrophage number in lung sections (*n* = 4 per group) from (**j**) given as the number of positive cells per 20 random fields of view. **l** Representative flow cytometry plots of whole lung single-cell suspensions to assess F4/80^+^ macrophages gated on CD45^+^ cells and CD11b vs CD11c expression gated on the F4/80^+^ cells, see Supplementary Fig. 24 for gating strategy. **m** Quantification of total F4/80^+^ macrophages (*n* = 4 for WT FA, WT CS, *Prmt7*^*+/−*^ FA and *n* = 5 for *Prmt7*^*+/−*^ CS) and CD11b^+^ F4/80^+^ macrophages (*n* = 4 for WT CS and *n* = 5 for *Prmt7*^*+/*−^ CS). **n** Representative images of H&E stained lung sections (scale bar 50 μm). **o** Quantification of airspace enlargement as mean chord length (MCL) in lung sections (*n* = 9 for WT FA, WT CS, *Prmt7*^*+/−*^ FA and *n* = 11 for *Prmt7*^*+/−*^ CS) from (**n**). **p** Lung function measured as diffusing capacity of carbon monoxide (*n* = 5 for WT FA, *n* = 6 for WT CS, *n* = 9 for *Prmt7*^*+/−*^ FA, and *n* = 6 for *Prmt7*^*+/−*^ CS). Data shown mean ± SD, *P* values shown in charts determined by one-way ANOVA Bonferroni’s multiple comparisons test (**a**, **k**, **m**, **o**, **p**), unpaired two-tailed Student’s *t*-test (**e**, **f**, **h**, **i**). Source data are provided as a Source Data file.

We next assessed whether impaired migration of monocytes was observed in PRMT7 reduced (*Prmt7*^+/−^) mice, which are characterized in Supplementary Fig. 8a–d^34^. Indeed, similar to our in vitro studies, ex vivo transendothelial migration assay demonstrated diminished migration of primary *Prmt7*^+/−^ monocytes toward the chemokine CCL2 (Fig. 4a), with no difference in the surface expression of CCR2 (Fig. 4b). To support this finding in vivo, we took advantage of the orthotopic lung transplantation model^35^, where we can explicitly investigate monocyte migration to disease pathogenesis since donor lungs retain resident macrophages upon transplant^36^. Left lungs from wild-type mice were transplanted into wild-type or *Prmt7*^+/−^ recipients of the same background, left for 3 weeks, and then exposed to a single dose of porcine pancreatic elastase (PPE) to induce monocyte-driven inflammatory response and tissue injury^37,38^ (Fig. 4c). Macrophage numbers were reduced in *Prmt7*^+/−^ compared to wild-type recipients (Fig. 4d–f), the crucial difference being monocyte migration ability in the *Prmt7*^+/−^ mice. Consequently, this protected against tissue injury and led to improved lung function (Fig. 4g–i). To confirm an intrinsic defect in monocyte recruitment in PRMT7-deficient monocytes, we next generated competitive bone marrow chimeric mice in CD45.1 recipients, that had been lethally irradiated and then reconstituted intravenously with 50% wild-type competitor bone marrow (CD45.1/2 heterozygous) and 50% *Prmt7*^+/−^ bone marrow (CD45.2), as macrophage repopulation of the lung has been shown to be dependent upon CCL2 mediated recruitment of monocytes following radiation exposure^39^. Upon flow cytometry analysis of the peripheral blood 8 weeks later, full chimerism was detected, with slightly more circulating leukocytes of *Prmt7*^+/−^ rather than wild-type origin. Crucially, however, the ability of these cells to repopulate the lungs and BAL with macrophages was impaired when of *Prmt7*^+/−^ rather than wild-type origin (Supplementary Fig. 9a–c). Furthermore, close examination of monocytes revealed reduced surface expression of CD11b (ITGAM) when originating from *Prmt7*^+/-^ rather than wild-type bone marrow (Supplementary Fig. 9d). These data strongly suggested that PRMT7 regulates the recruitment of monocytes into the lung and subsequent lung tissue injury.

Next, *Prmt7*^+/−^ mice were exposed to acute CS for 3 days and demonstrated reduced macrophage recruitment into the bronchoalveolar (BAL) fluid and lung tissue (Supplementary Fig. 10a–e), while neutrophil numbers similarly increased (Supplementary Fig. 10a). However, expression levels of chemoattractants *Ccl2* (monocyte) and *Cxcl1* (neutrophil) were elevated in both *Prmt7*^+/−^ and wild-type mice (Supplementary Fig. 10f). Similar to acute exposure, reduced macrophages were detected in the lungs of *Prmt7*^+/−^ mice following chronic CS (Fig. 4j, k and Supplementary Fig. 10g, h), again with similar increases in CCL2 and CXCL1 (Supplementary Fig. 10i). In support, characterization by flow cytometry revealed that there was a reduction in F4/80^+^CD11b^+^ recruited monocyte-derived macrophages in *Prmt7*^+/−^ mice following chronic CS exposure (Fig. 4l, m) but not activated lymphocytes (Supplementary Fig. 10j, k). Crucially, expression of *Icam1* and *Vcam1*, key leukocyte adhesion molecules^40^, did not differ between the lungs of wild-type or *Prmt7*^+/−^ mice following both acute and chronic CS exposure (Supplementary Fig. 11a–d). Similarly, treatment of SVEC4-10 endothelial cells with the inhibitor of PRMT7 activity, SGC3027^27^, following TNF stimulation did not alter the expression of *Icam1* and *Vcam1* (Supplementary Fig. 11e). Finally, impaired recruitment of monocytes into the lungs of *Prmt7*^+/−^ mice prevented CS-induced lung injury^4,41,42^ (Fig. 4n, o) and concomitantly improved lung function (Fig. 4p).

Arginine methylation has been implicated in the regulation of myeloid development, particularly PRMT1 and PRMT5 in myeloid differentiation and myeloid leukemia^43–46^. It was therefore imperative to examine the effect of PRMT7 deficiency upon monocyte development and maturation to exclude this as a possible mechanism for the impaired recruitment to the lung. Flow cytometry analysis revealed the development of CD11b^+^ CD45^+^ cells and particularly Ly6c^+^ CCR2^+^ CD11b^+^ classical monocytes in the bone marrow of *Prmt7*^+/−^ and wild-type mice are similar (Supplementary Fig. 12a–c). Furthermore, isolation of mature monocytes from the bone marrow of both wild-type and *Prmt7*^+/−^ mice yielded similar numbers (Supplementary Fig. 12d). Crucially, their ability to then differentiate into macrophages (Supplementary Fig. 12e–h) and subsequently activate and polarize (Supplementary Fig. 13a–c) did not differ between wild-type and *Prmt7*^+/−^ bone marrow, likewise for dendritic cell (DC) development and activation (Supplementary Fig. 14a–d).

To validate the significance of these findings, we first assessed an additional model of macrophage-driven COPD^37,38^ by instilling PPE. Early (24 h) and late (28 days) post-exposure of PPE, *Prmt7*^+/−^ mice had less macrophage recruitment (Supplementary Fig. 15a–f) and again were protected from lung tissue injury (Supplementary Fig. 15g–i). Next, to comprehend a possible broader implication of PRMT7 regulated monocyte migration, we assessed conditional knockout mice that lacked PRMT7 expression restricted to the myeloid compartment (*Lyz2-Cre Prmt7*^*fl/fl*^). Initially, *Lyz2-Cre Prmt7*^*fl/fl*^ mice were exposed to CS, which similar to *Prmt7*^+/−^ mice, resulted in reduced monocyte-driven macrophages in their lungs and BAL compared to control animals (Supplementary Fig. 16a–e). Following this, we utilized the bleomycin model of human lung fibrosis^47^. Fourteen days following a single instillation of bleomycin, *Lyz2-Cre Prmt7*^*fl/fl*^ mice demonstrated reduced numbers of macrophages in their lungs (Supplementary Fig. 17a, b), accompanied by a less severe disease phenotype^48^ (Supplementary Fig. 17c, d). In support, using a 3-day acute skin injury model where an accumulation of monocyte-derived macrophages occurs^49^, recruitment was significantly reduced in the skin wounds of *Lyz2-Cre Prmt7*^*fl/fl*^ mice (Supplementary Fig. 17e, f).

Further evidence for a monocyte-specific role of PRMT7 in disease development was supported by the finding that primary alveolar epithelial type 2 (AT2) cells from both wild-type and *Prmt7*^+/−^ mice trans-differentiate into type 1 cells comparably (Supplementary Fig. 16f). In addition, siRNA knockdown of *Prmt7* in the murine AT2 cell line MLE12 (Supplementary Fig. 16g), did not alter the cells ability to proliferate and repair wounds (Supplementary Fig. 16h, i). Taken all together this data suggests that PRMT7 regulated monocyte recruitment to the lung contributes to lung injury.

### Inflammatory monocytes induce ferroptosis of lung alveolar epithelial cells in COPD

**Fig. 5:**
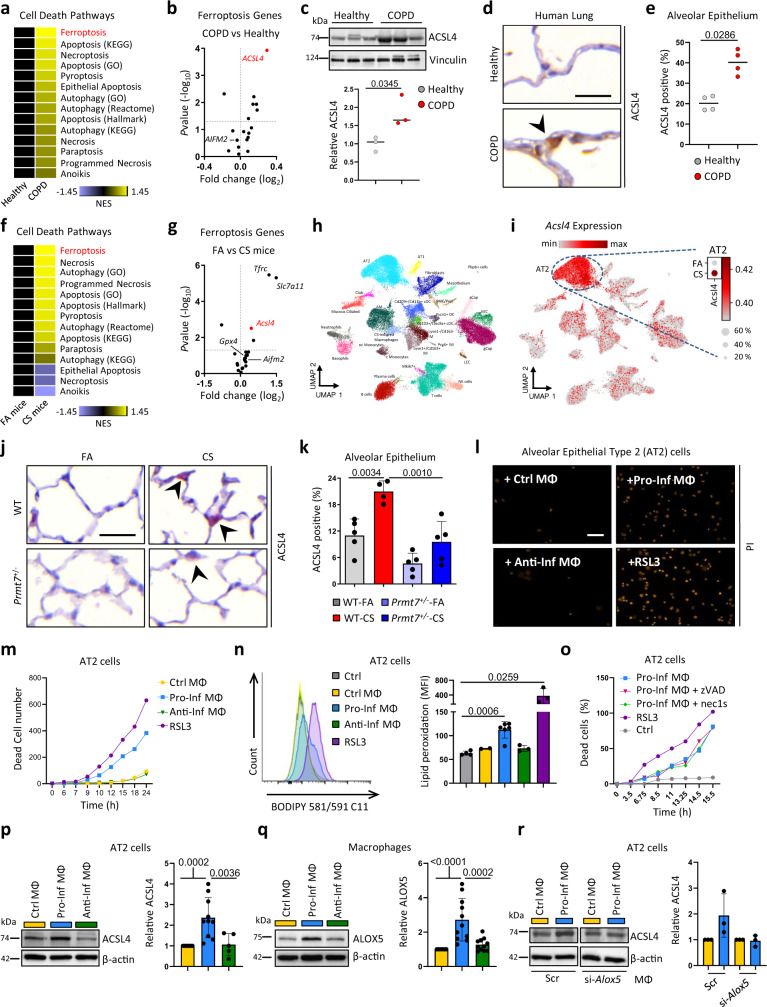
Ferroptosis of lung alveolar epithelial cells in COPD induced by inflammatory macrophages. **a** Heat map of normalized enrichment score (NES) following GSEA of cell death pathway gene lists on array data from lung tissue of COPD patients (GSE47460-GPL14550; 145 COPD patients vs 91 healthy controls). **b** Expression of ferroptotic genes in the lungs of COPD patients relative to healthy controls taken from GSE47460-GPL14550, relative fold change and *P* value calculated using the GEO2R interactive web tool running limma R**. c** Western blot analysis of ACSL4 expression in lung core biopsies from healthy (*n* = 3) and COPD patients (*n* = 3), quantification relative to vinculin shown for individual patients. **d** Representative images of immunohistochemical analysis for ACSL4 (brown signal indicated by arrowhead, hematoxylin counterstained, scale bar 25 μm) in lung sections from COPD patients (*n* = 4) and healthy controls (*n* = 4). **e** Quantification of alveolar epithelial cells positive for ACSL4. **f** Heat map of NES following GSEA of cell death pathway gene lists on array data from mice exposed to 4 m chronic cigarette smoke (CS, *n* = 3) v filtered air (FA, *n* = 3). **g** Expression of ferroptotic genes in the lungs of mice exposed to 4 m chronic CS (*n* = 3) relative to FA (*n* = 3) taken from array data used in (**f**), relative fold change and *P* value calculated using the GEO2R interactive web tool running limma R. **h**, **i** Cells from whole lung suspensions of mice exposed to FA (*n* = 3) or CS for 4 m (*n* = 5), were analysed by scRNA-Seq (Drop-Seq). **h** UMAP of scRNA-Seq profiles (dots) colored by cell type. **i** UMAP plots showing expression of ACSL4 in scRNA-Seq profiles. **j** Representative images of immunohistochemical analysis for ACSL4 (red signal indicated by arrowheads, hematoxylin counterstained, scale bar 25 μm) in lung sections from WT and *Prmt7*^+/−^ mice exposed to FA or CS for 4 months (*n* = 4–5 per group). **k** Quantification of alveolar epithelial cells positive for ACSL4 from (**j**) (*n* = 5 for WT FA, *Prmt7*^*+/*−^ FA, *Prmt7*^*+/−*^ CS and *n* = 4 for WT CS). **l**, **m** Mouse AT2 cells (MLE12) were treated with RSL3 (250 nM), or conditioned medium from control macrophages (MФ, RAW264.7 cells) or those polarized to a pro-inflammatory (IFNγ + LPS) or anti-inflammatory (IL4) phenotype for 48 h, representative data shown from a single experiment repeated twice. **l** Representative image showing PI uptake at 24 h (Scale bar 50 µm). **m** Number of dead AT2 cells at the time indicated, data shown from a single experiment repeated twice. **n** BODIPY oxidation in AT2 cells (MLE12) at 6 h after treatment with RSL3 (250 nM, *n* = 3) or conditioned medium from control macrophages (*n* = 2) or those polarized to a pro-inflammatory (*n* = 6) or anti-inflammatory (*n* = 3) phenotype**. o** Number of dead AT2 cells (MLE12) by PI uptake at the time indicated following treatment with RSL3 (250 nM), or conditioned medium from macrophages (MФ, RAW264.7 cells) polarized to pro-inflammatory (IFNγ + LPS) plus 20 μM zVAD or 10 μM necrostatin-1s (Nec1-s), representative data shown from a single experiment repeated twice. **p** Western blot analysis of ACSL4 in AT2 cells (MLE12) treated for 24 h with conditioned medium from control macrophages (*n* = 11) or those polarized to a pro-inflammatory (*n* = 10) or anti-inflammatory (*n* = 5) phenotype, normalized to β-actin and shown relative to control macrophages. **q** Western blot analysis of ALOX5 at 72 h in control macrophages (*n* = 11) or those polarized to a pro-inflammatory (*n* = 11) or anti-inflammatory (*n* = 11) phenotype, normalized to β-actin and shown relative to control macrophages. **r** Western blot analysis of ACSL4 in AT2 cells (MLE12) treated for 24 h with conditioned medium from control macrophages or those polarized to a pro-inflammatory phenotype that were treated with siRNA against *Alox5*, normalized to β-actin and shown relative to control macrophages, from individual experiments (*n* = 3). Data shown mean ± SD, *P* values shown in charts determined by unpaired two-tailed Student’s *t*-test (**c**, **n**), two-tailed Mann–Whitney test (**e**), one-way ANOVA Bonferroni’s multiple comparisons test (**k**, **p**, **q**). Source data are provided as a Source Data file.

COPD pathogenesis is characterized by lung tissue injury driven by alveolar epithelial cell death^50,51^ (Supplementary Fig. 18a). In light of recent advances in our understanding of different cell death modalities^52^, we undertook GSEA of cell death pathways upon transcriptomics data obtained from the lungs of 145 COPD patients and 91 healthy controls (GSE47460) and mice exposed to chronic cigarette smoke. Strikingly, ferroptosis, a regulated form of necrotic cell death characterized by iron-dependent lipid peroxidation^53^, was the most enriched cell death pathway in both data sets (Fig. 5a, f; Supplementary Fig. 18b; and Supplementary Data Files 6, 7), with the ferroptosis sensitizer ACSL4^54^, being the most upregulated pro-ferroptotic gene in the lungs of COPD patients (Fig. 5b). ACSL4 drives the esterification of CoA to free fatty acids, with acyl-CoA activating the corresponding fatty acids for fatty acid oxidation or phospholipid biosynthesis. ACSL4 has a preference for long-chain polyunsaturated fatty acids, importantly it is these longer and unsaturated fatty acids that exacerbate ferroptosis induction when esterified and peroxidized in membrane phospholipids^54^. Notably, ACSL4 upregulation was found to correlate with disease severity (Supplementary Fig. 18c) and was strongly upregulated in CS-exposed mice (Fig. 5g), along with two other genes, *Tfrc* and *Slc7a11*, another two canonical genes involved in ferroptosis control. ACSL4 was validated in our cohort of COPD patients and its expression was strongly enriched in alveolar epithelial cells (Fig. 5c-e and Supplementary Fig. 18d). Single-cell RNA-seq of the lungs from mice exposed to chronic CS revealed 29 cell type identities (Fig. 5h and Supplementary Fig. 18e), with *Acsl4* predominantly found in AT2 cells with its expression being increased in mice following CS exposure (Fig. 5i). This was confirmed by quantitative morphological analysis (Fig. 5j, k and Supplementary Fig. 18f). Importantly, ACSL4 in AT2 cells was crucial for ferroptotic cell death mediated by the GPX4 inhibitor RSL3^55^ (Supplementary Fig. 18h), which was prevented in *Acsl4*^−/−^ cells (Supplementary Fig. 18h) and reversible by co-treatment with the ferroptosis inhibitor liproxstatin-1 (Supplementary Fig. 18i). Additionally, *Prmt7*^+/−^ mice that were protected from emphysema development did not show increased expression of ACSL4 in alveolar epithelial cells following CS exposure (Fig. 5j, k), despite AT2 cells with impaired PRMT7 activity retaining the ability to upregulate ACSL4 (Supplementary Fig. 18g).

However, as *Prmt7*^+/−^ mice are characterized by impaired monocyte recruitment and subsequent reduced inflammatory macrophage accumulation in the lungs following CS exposure, as demonstrated by multiplex immunofluorescence analysis of iNOS and CD206 positive macrophages (Supplementary Fig. 19a–d), we examined the ability of inflammatory macrophages to induce ferroptotic cell death of alveolar epithelial cells. Conditioned medium from inflammatory monocyte-derived macrophages (IFNγ + LPS treated RAW264.7 macrophages) induced cell death of AT2 cells (MLE12) similar to RSL3 (Fig. 5l, m; Supplementary Fig. 20a; and Supplementary Movies 1, 2), and sensitized AT2 cells to RSL3 mediated death (Supplementary Fig. 20b). Conditioned medium from control or anti-inflammatory (IL4 treated) macrophages did not induce cell death (Fig. 5l, m and Supplementary Movies 3, 4), crucially neither did direct treatment of AT2 cells with pro- and anti-inflammatory cytokines (Supplementary Fig. 20c). Importantly, conditioned medium from inflammatory macrophages induced rapid lipid peroxidation as determined by BODIPY 581/591 C11 oxidation in AT2 cells (Fig. 5n), which was reversed by liproxstatin-1 (Supplementary Fig. 20d), by contrast, the resulting cell death was not prevented by zVAD-FMK (a pan-caspase inhibitor) or necrostatin-1s (a necroptosis inhibitor) (Fig. 5o). In addition, this was accompanied by increased expression of ACSL4 (Fig. 5p) in AT2 cells. Interestingly, CRISPR/Cas9 knockout of *Prmt7* in AT2 cells (MLE12) (Supplementary Fig. 20e–g) did not protect from increased lipid peroxidation, increased ACSL4 expression nor cell death following treatment with RSL3 or conditioned medium from inflammatory macrophages (Supplementary Fig. 20h–k). This data suggests that inflammatory macrophages can induce ferroptosis susceptibility of lung alveolar epithelial cells, which may aggravate the progression of lung tissue injury.

Interestingly, to establish a mechanistic link between inflammatory macrophages and epithelial cell death, as leukotrienes produced via the 5-lipoxygenase pathway can cause aberrant inflammation and impair tissue repair^56^, we clearly observed increased ALOX5 expression in pro-inflammatory macrophages (Fig. 5q and Supplementary Fig. 21a). This was accompanied by increased LOX activity (Supplementary Fig. 21b), reduced GPX4 expression (Supplementary Fig. 21c), an indirect inhibitor of LOX activity^57^, and increased secretion of leukotriene B4 (LTB4), an active metabolite of ALOX5 (Supplementary Fig. 21d). Crucially, when ALOX5 expression was suppressed by siRNA knockdown in pro-inflammatory macrophages, the conditioned medium from these cells no longer induced increased expression of ACSL4 in AT2 cells (Fig. 5r). Furthermore, we observed increased expression of ALOX5 in the CS-induced macrophage population following scRNA-seq of the lungs from CS-exposed mice (Supplementary Fig. 21e), likewise in monocyte-like macrophages from the bronchoalveolar lavage of COPD patients as determined by scRNA-seq (Fig. 1n). In support, a decreased number of ALOX5 positive macrophages was detected in the lungs of *Prmt7*^+/−^ mice that were protected from tissue injury following CS exposure (Supplementary Fig. 21f, g), accompanied by reduced levels of LTB4 in the BAL (Supplementary Fig. 21h). To highlight the importance of accumulating monocyte-derived macrophages in contributing to alveolar epithelial cell death in COPD, we cultured macrophages at increasing cell numbers, polarized towards a pro-inflammatory phenotype and then transferred the conditioned medium to AT2 cells. Supplementary Fig. 21i, j clearly indicates that as the macrophage number increases there is a cell number dependent increase in both lipid peroxidation and cell death of AT2 cells. This was not dependent upon PRMT7 expression as pro-inflammatory polarized *Prmt7*^null^ macrophages retained the ability to upregulate ALOX5 concomitant with reduced GPX4 expression (Supplementary Fig. 22a). As well as PRMT7-deficient pro-inflammatory polarized RAW cells retain the ability to increase ALOX5 expression, secrete LTB4 and drive increased ACSL4 expression accompanied by enhanced lipid peroxidation and ferroptotic cell death of AT2 cells (Supplementary Fig. 22b–f). Also, bone marrow of both wild-type and *Prmt7*^+/-^ mice differentiated in the presence of M-CSF or GM-CSF for 7 days and subsequently polarized towards a pro-inflammatory phenotype induced cell death of AT2 cells equivalently (Supplementary Fig. 22g, h). Finally, LTB4 increased ACSL4 expression sensitizing AT2 cells to RSL3 mediated death (Supplementary Fig. 22i, j). Conclusively, this data implies that ALOX5 derived LTB4 in pro-inflammatory monocyte-derived macrophages in a paracrine manner contributes to ferroptosis sensitization of alveolar epithelial cells.

## Discussion

Here, we identify arginine mono-methylation mediated by PRMT7 in monocytes to be an essential factor for tissue accumulation after inflammatory insult. Accumulating inflammatory macrophages induced susceptibility to ferroptotic cell death of alveolar epithelial cells, contributing to disease pathogenesis in COPD. Mechanistically, we have discovered that PRMT7 expression is increased by inflammatory NF-κB/RelA signaling. PRMT7 induced mono-methylation of histones at enhancers can regulate *Rap1a* expression, which is crucial for MAPK signaling downstream of G-protein coupled activation, integrin activation, and the subsequent adhesion and migration ability of monocytes (Supplementary Fig. 23). Further, inflammatory macrophages via ALOX5-mediated release of LTB4 induced increased expression of ACSL4 in AT2 cells increasing susceptibility to CS-induced ferroptotic cell death and tissue injury.

We found that PRMT7 is highly enriched in COPD patients as well as in CS-exposed mice. Interestingly, depletion of PRMT7 in mice protected these from multiple animal models of chronic lung disease. Our lung single-cell RNA-seq data revealed the greatest amount of expressed *Prmt7* across all lung cells to be in plasma cells followed by a macrophage subpopulation unique to CS exposure, that arises from classical inflammatory monocytes. Inline, monocyte-like macrophages were greater in the BAL of COPD patients concomitant with increased expression of *PRMT7*. Although previously published data showed a crucial role of PRMT7 in regulating plasma cell differentiation through *Bcl6*^34^, the here identified role of PRMT7 in regulating immune cell adhesion and transmigration is specific for monocytes, since neutrophil and B and T cell recruitment was not impaired in the absence of PRMT7 upon CS or elastase exposure. The population of CS-induced macrophages that we discovered in this study shows high expression of *Spp1*, a COPD marker, and a co-expression of *Gpnmb* and *Lgals3*. Albeit, the dysregulation of PRMTs in human and experimental pulmonary diseases such as COPD, asthma, pulmonary hypertension, lung fibrosis, or lung cancer have been characterized, PRMT7 was not yet described. Therefore, our data that shows the control of PRMT7 expression through the inflammatory NF-κB/RelA signaling pathway is particularly important to a multitude of inflammatory conditions. Noteworthy, a recent study shows the regulation of inflammation in a CS extract induced murine model of emphysema through PRMT6 and the NF_k_B pathway^16^, however, our data clearly shows no existing reciprocal regulatory mechanisms between PRMT6 and PRMT7.

PRMT7 catalyzes the mono-methylation of arginine residues in a variety of target proteins including histones^6^. By performing proteomic analysis of MMA enzymatic activity using wild-type and *Prmt7*^null^ cells, we found leukocyte transendothelial migration as well as leukocyte adhesion to be impaired in the absence of PRMT7. In a competitive bone marrow chimeric experiment and ex vivo transendothelial migration assays, we confirmed the impaired migration ability of PRMT7-deficient monocytes. Enrichment analysis of proteomics revealed a common player, the small G protein of the Ras superfamily, RAP1A, that showed reduced expression levels in the absence of PRMT7. RAP1A, activated by GTP binding was previously shown to enhance integrin-dependent signaling and by that regulates cellular adhesion and migration^30,31^. ATAC-Seq and ChIP-qPCR data confirmed the transcriptional regulator role of PRMT7 for RAP1A in conjunction with H3R2me2s which has been identified as an activation mark by excluding binding of multiple co-repressor complexes and by enhancing binding of WDR5, a common component of coactivator complexes^58^. Most crucially, increased transcription of *Prmt7* parallels the increased expression of RAP1A in monocytes from CS-exposed mice lungs. Furthermore, we used PLA assay analysis to also confirm direct mono-methylation of RAP1A protein. Importantly, we could exclude in all PRMT7-depleted cell systems any side effects due to the absence of PRMT7 upon the maturation, polarization, or activation of macrophages or their ability to upregulate ALOX5 under pro-inflammatory conditions. Crucially, the capability of AT2 cells to trans-differentiate into AT1 cells, to induce lipid peroxidation, to upregulate ACSL4, and to undergo ferroptotic cell death was not intrinsically dependent upon PRMT7. Taken together, this data suggests that monocyte migration is regulated by PRMT7 through RAP1A. Although, we demonstrated that decreased expression levels of RAP1A result in impaired MAPK signaling, reduced integrin surface expression, and diminished migration of monocytes, the mode of action of methylated RAP1A protein needs to be further investigated, in particular, because we found RAP1A to be a potential new substrate of PRMT7.

While the contribution of monocytes and differentiated macrophages to the development of COPD is well described^37,38,59^, their contribution to the alveolar epithelial cell death observed in emphysema remained unknown. Our work demonstrates that upregulation and enhanced activity of ALOX5 in inflammatory macrophages and the release of leukotriene B4 (LTB4) results in the induction of ACSL4 expression, lipid peroxidation, and ferroptotic cell death in AT2 cells. Importantly, downregulation of ALOX5 in pro-inflammatory macrophages failed to induce ferroptotic cell death in AT2 cells. Leukotrienes have been implicated in the pathogenesis of acute exacerbations of COPD and LTB4 has previously been found to be increased in the serum of cigarette smokers and COPD patients^60^. However, beyond its role as a neutrophilic chemoattractant^61^, we now describe a function of LTB4 in inducing ferroptotic cell death under smoking conditions. Studies on ALOX5 support our findings since ALOX5 deficiency or pharmacological targeting of the 5-lox pathway resulted in accelerated cutaneous healing^62^. Similarly, *5-lox* deficient mice were protected against acute inflammatory injury^63^. Furthermore, the inhibition of ALOX5 with the commercially available inhibitor zileuton has been shown in a number of clinical trials to improve airway function and inflammation^64^. Taken altogether, our data clearly shows that ALOX5 derived LTB4 in pro-inflammatory macrophages contributes to ferroptosis sensitization of alveolar epithelial cells in a paracrine manner. Although, our study focuses on macrophage released LTB4 as a ferroptotic cell death inducer and despite the fact that neutrophil recruitment was not impaired in the absence of PRMT7 in both CS and elastase mouse models, it is important to consider that neutrophil influx and activation also plays a significant role in the pathogenesis of COPD^65^. However, the absence of neutrophil elastase did not lead to complete protection from cigarette smoke-induced emphysema as was demonstrated for the depletion of macrophage elastase (MMP-12), which was accompanied by reduced macrophage accumulation in the lungs^59,65^.

Our study expands the understanding of epigenetic regulation and its role in modulating immune responses in COPD. Despite, we could show a correlation between smoking history and *PRMT7* expression based on the pack-years of patients, it is crucial to investigate besides pack-years, the duration of smoking and when COPD patients started smoking. Crucially, however, COPD patients demonstrated clear enrichment for methyltransferase activity and ferroptotic cell death in their lungs, with *PRMT7* and *ACSL4* expression correlating with disease severity. Thus, targeting epigenetic regulation mediated by arginine methylation offers therapeutic potential against inflammatory conditions driven by the recruitment of monocytes.

## Methods

### Human lung tissue core sampling (Cohort 3)

Lung core samples from explanted lungs of COPD patients undergoing lung transplantation were provided by Dr. Stijn Verleden (University of Leuven, Belgium) following ethical approval of the University of Leuven Institutional Review Board (ML6385). All participants provided informed written consent. For controls, unused donor lungs were collected under existing Belgian law which allows the use of declined donor lungs for research after second opinion inspection. Lungs were declined for various reasons (logistics, presence of microthrombi, kidney tumor). Patient demographics are highlighted in Supplementary Table 1. Lungs were air-inflated at 10 cm H_2_O pressure and fixed using constant pressure in the fumes of liquid nitrogen, sliced using a band saw, and then sampled using a core bore. Upon receipt, lung cores were portioned for fixation in 4% paraformaldehyde followed by paraffin embedding, and snap-frozen in liquid nitrogen for protein extraction or total RNA isolation (peqGOLD Total RNA Kit, Peqlab).

### Human study population (Cohort 4)

Lung resection specimens were obtained from 118 participants, of which 106 were from surgery for solitary pulmonary tumors (Ghent University Hospital, Ghent, Belgium) and 12 were from explant lungs of end-stage COPD patients undergoing lung transplantation (University Hospital Gasthuisberg, Leuven, Belgium). Lung tissue from the resection specimen was harvested by a pathologist at a maximum distance from the tumor. Based on medical history, smoking history, questionnaires, and pre-operative spirometry, the cohort of 118 participants was divided into six subgroups: 28 never-smokers, 22 current smokers without airflow limitation, 14 ex-smokers without airflow limitation, 28 current smoker patients with COPD GOLD stage II, 24 ex-smoker patients with COPD GOLD stage II and 12 smoker patients with COPD GOLD stage III-IV (see Supplementary Table 2 for participant characteristics). All patients with COPD had stable disease as patients with exacerbations within 2 months before the study were excluded. COPD severity was defined according to the Global Initiative for Chronic Obstructive Lung Disease (GOLD) classification. Participants were considered ex-smokers when they had quit smoking for more than 1 year. None of the participants were treated with neoadjuvant chemotherapy. Lung tissue of patients diagnosed with solitary pulmonary tumors was obtained at a maximum distance from the pulmonary lesions and without signs of retro-obstructive pneumonia or tumor invasion and collected by a pathologist. Lung tissue of patients with COPD GOLD III-IV was obtained from lung explants of end-stage COPD patients undergoing lung transplantation. Written informed consent was obtained from all participants. This study was approved by the medical ethical committees of the Ghent University Hospital (2011/0114; 2016/0132; 2019/0537) and the University Hospital Gasthuisberg, Leuven (S51577).

Total RNA from lung resection specimens was extracted using the miRNeasy Mini Kit (Qiagen, Hilden, Germany). Next, cDNA was prepared with the EvoScript Universal cDNA Master Kit (Roche), followed by RT-PCR analysis. Taqman Gene Expression Assays (Applied Biosystems, Forster City, CA, USA) were used to measure the expression of the reference genes glyceraldehyde-3-phosphate dehydrogenase (GAPDH; Hs99999905_m1), peptidylprolyl isomerase A (PPIA; Hs99999904_m1), and succinate dehydrogenase complex flavoprotein subunit A (SDHA; Hs00417200_m1). Data were analyzed using the standard curve method and expression of PRMT7 was calculated relative to the expression of the three reference genes.

### Human bronchoalveolar lavage samples for single-cell RNA-seq

Human data of BALF immune cells from COPD GOLD 2 patients (*n* = 9) and control donors (*n* = 6) were taken from a dataset that was recently described by Bassler et al.^66^. The data were processed as described by Bassler et al. To visualize the gene expression of the human orthologues of *PRMT6/7*, *RAP1A*, and *ALOX5*, we used the ComplexHeatmap package (version: 2.2.0)^67^ and ggplot2 (https://ggplot2-book.org/; version: 3.3.0).

### Isolation of human primary monocytes

The study was approved by the Ethics Committee of Koc University and informed written consent were taken from study volunteers (see Supplementary Table 3 for participant characteristics and demographics). As a first step, human PBMCs were separated via ficoll density gradient from blood. Blood was diluted twofold with PBS and carefully placed into falcons prefilled with pancoll (density 1.077 g/ml, Pan Biotech, P04-60500) without mixing blood and pancoll. Samples were centrifuged at 800×*g* for 20 min at 20 °C with no brake and low acceleration. The upper layer of plasma and platelets were removed and discarded while the PBMC layer above the pancoll was collected and transferred into a new falcon. Three volumes of PBS were added into PBMCs to resuspend and centrifuged again at 300×*g* for 10 min at 20 °C. Pellet was washed with PBS and replaced with 10 ml MACS buffer followed by cell counting. Finally, monocytes from PBMCs were isolated using a PAN monocyte isolation kit (Miltenyi, #130-096-537). Isolated monocytes were directly frozen for RNA isolation and further processing.

### Animals and maintenance

C57BL/6N-Tyr^c-Brd^ *Prmt7*^tm1a(EUCOMM)Wtsi^/WtsiCnbc mice were obtained from the European Conditional Mouse Mutagenesis Program (EUCOMM), The Wellcome Trust Sanger Institute, Cambridge, UK. Heterozygous mice carrying a single copy of the insertion (referred to as *Prmt7*^+/−^ mice), were bred with wild-type siblings to maintain the colony. Eight- to ten-week-old *Prmt7*^+/−^ mice and their wild-type littermate controls (males and females) were used in all experiments. C57BL/6N-Tyr^c-Brd^ *Prmt7*^tm1a(EUCOMM)Wtsi^/WtsiCnbc mice were further crossed with FLPe expressing mice, B6.129S4-*Gt(ROSA)26Sor*^tm1(FLP1)Dym^/RainJ (#009086, The Jackson Laboratory), to delete the FRT flanked insertion cassette, leaving a LoxP flanked exon 3 of *Prmt7*. FLPe expression was bred out of the mice, and the mice were then crossed with B6.129P2-*Lyz2*^tml(cre)Ifo^/J (#004781, The Jackson Laboratory) to generate *Lyz2- Cre Prmt7*^*flox/flox*^ mice to understand the role of PRMT7 specifically in macrophages. Eight- to ten-week-old *Lyz2- Cre Prmt7*^*flox/flox*^ mice and their *Prmt7*^*flox/flox*^ littermate controls not expressing Cre (males and females) were used in all experiments. Mice were housed under specific pathogen-free conditions and maintained at a constant temperature (20–24 °C) and humidity (45–65 %) with a 12-h light cycle, and were allowed food and water ad libitum. Mice were euthanized by terminal exsanguination following anesthetic. All animal experiments were approved by the ethics committee for animal welfare of the local government for the administrative region of Upper Bavaria (Regierungspräsidium Oberbayern) and were conducted under strict governmental and international guidelines in accordance with EU Directive 2010/63/EU.

### Cigarette smoke (CS) exposure

CS was generated from 3R4F Research Cigarettes (Tobacco Research Institute, University of Kentucky, Lexington, KY), with the filters removed. Mice were whole-body exposed to active 100% mainstream CS of 500 mg/m^3^ total particulate matter (TPM) for 50 min twice per day for 3 days or 4 months, in a manner mimicking natural human smoking habits as previously described^68^. The TPM level was monitored via gravimetric analysis of quartz fiber filters prior to and after sampling air from the exposure chamber and measuring the total air volume. Filtered air (FA)-exposed mice were used as controls. Mice were analyzed the day after the final smoking exposure.

### Elastase application

Emphysema was induced in mice by oropharyngeal application of a single dose of porcine pancreatic elastase (PPE, 40 U/kg body weight in 80 µl volume, Sigma-Aldrich) as previously described^69^. Control mice received 80 µl of sterile PBS. Mice were analyzed 24 h or 28 days after instillation.

### Bleomycin application

Lung fibrosis was induced in mice by oropharyngeal application of a single dose of bleomycin (2 U/kg body weight in 80 µl volume, Sigma-Aldrich). Control mice received 80 µl of sterile PBS. Mice were analyzed 14 days after instillation.

### Splinted full-thickness excisional wound model

Splinting rings are prepared from a 0.5 mm silicone sheet (Grace Bio-Labs, JTR-S-0.5) by cutting rings with an outer diameter of 12 mm and an inner diameter of 6 mm. After washing with detergent, rinsing with water, the splints are sterilized with 70% ethanol for 30 min and air-dried in a cell culture hood, and kept in a sterile bottle.

Mice are anesthetized with 100 µl of MMF (medetomidine, midazolam, and fentanyl). Dorsal hair is removed by a hair clipper (Aesculap Schermaschine Exacta), followed by hair removal cream for 2–3 min. Two full-thickness excisional wounds are created with a 5 mm diameter biopsy punch (Stiefel). One side of a splint is applied with silicone elastomer super glue (Kwik-Sil Adhesive, World Precision Instruments) and placed around the wound. The splint is secured with 6 sutures of 6.0 nylon, and the wound is covered with Tegaderm transparent dressing (3 M). Mice are recovered from anesthesia with MMF antagonist and are supplied with Metamizole (500 mg Metamizole/250 ml drinking water) as postoperative analgesia. On day 3 post-wounding, mice are sacrificed by cervical dislocation. The full-thickness wound tissues are harvested by cutting 2 mm away from the wound edge. The adjacent normal full-thickness skin is harvested with a biopsy punch, which serves as the control. The harvested wound and skin tissues are fixed in 2% paraformaldehyde (PFA) for further analysis.

### Orthotopic lung transplantation

Left lungs from wild-type mice were orthotopically transplanted into wild-type or *Prmt7*^+/−^ recipients as previously described^35^. These are syngeneic transplants, that macroscopically and histologically appear normal^35^, however, they were left for 3 weeks to recover from surgery before being exposed to a single dose of PPE (40 U/kg body weight) oropharyngeally and analyzed 28 days later.

### Competitive bone marrow chimeras

Female recipient C57BL/6 congenic mice 8–10 weeks old, B6.SJL-*Ptprc*^a^ *Pepc*^b^/BoyJ (CD45.1, #002014, The Jackson Laboratory), were lethally irradiated with two separate doses (2 × 550 cGy) on two consecutive days using an X-ray source (xstrahl CIX2). On the second day, 3.4 × 10^6^ bone marrow cells per recipient mouse were injected intravenously. The bone marrow (BM) consisted of 50% WT competitor BM (CD45.1/2 heterozygous) and 50% *Prmt7*^+/−^ BM (CD45.2) from female mice. Chimeras were supplied with antibiotics (0.04% Baytril) containing drinking water for 2 weeks and were analyzed 8 weeks after bone marrow transfer by flow cytometric analysis of peripheral blood, bronchoalveolar lavage, and whole lung. Single-cell suspensions were blocked with purified anti-mouse CD16/CD32 (1:100, clone 93, Cat. No. 14-0161-82, eBioscience, Thermo Fisher Scientific) before incubating for 30 min on ice with the following cocktails: VioGreen-conjugated anti-CD45.2 (1:10, clone: 104-2, Cat. No. 130-102-312, Miltenyi Biotec), FITC-conjugated anti-CD45.1 (1:50, clone: A20, Cat. No. 130-124-211, Miltenyi Biotec), VioBlue-conjugated anti-Ly6g (1:50, clone: REA526, Cat. No. 130-119-986, Mitenyi Biotec), PE-Vio770-conjugated anti-F4/80 (1:50, clone: REA126, Cat. No. 130-118-459, Miltenyi Biotec), PE-conjugated anti-CD11b (1:50, clone: REA592, Cat. No. 130-113-806, Miltenyi Biotec), and APC-conjugated anti-CD11c (1:50, clone: REA754, Cat. No. 130-110-839, Miltenyi Biotec). Cocktail 2: VioGreen-conjugated anti-CD45.2 (1:10, clone: 104-2, Cat. No. 130-102-312, Miltenyi Biotec), FITC-conjugated anti-CD45.1 (1:50, clone: A20, Cat. No. 130-124-211, Miltenyi Biotec), VioBlue-conjugated anti-Ly6g (1:50, clone: REA526, Cat. No. 130-119-986, Mitenyi Biotec), PE-conjugated anti-CD11b (1:50, clone: REA592, Cat. No. 130-113-806, Miltenyi Biotec), APC-conjugated anti-CD192 (CCR2) (1:50, clone: REA538, Cat. No. 130-119-658, Miltenyi Biotec) and PE-Vio770-conjugated anti-Ly6c (1:50, clone: REA796, Cat. No. 130-111-780, Miltenyi Biotec). Live/Dead discrimination using 7-AAD (Miltenyi Biotec). Cells were analyzed on a BD FACSCanto II flow cytometer (BD Biosciences) with BD FACSDiva v6.1.3 software.

### Lung function measurements

Mice were anesthetized with ketamine-xylazine, tracheostomized and the diffusing capacity for carbon monoxide (DFCO) was calculated^70^. In brief, 0.8 ml mixed gas (0.5% Ne, 21% O_2_, 0.5% CO, and 78% N_2_) was instilled into the mice lungs and withdrawn 2 s later for analysis on a 3000 Micro GC Gas Analyzer (Infinicon) running EZ IQ software v3.3.2 (Infinicon). DFCO was calculated as 1-(CO_1_/CO_0_)/(Ne_1_/Ne_0_) where 0 and 1 refer to the gas concentration before and after instillation respectively. Respiratory function was analyzed using a forced pulmonary maneuver system^71^ (Buxco Research Company, Data Sciences International) running FinePointe Software (version 6, Data Sciences International). At least three maneuvers were performed per mouse and the mean value was taken.

### Single-cell RNA sequencing using Drop-seq

Murine lobes were removed, minced, and digested for 20–30 min at 37 °C in an enzymatic mix containing dispase (50 caseinolytic U/ml), collagenase (2 mg/ml), elastase (1 mg/ml), and DNase (30 μg/ml). Single cells were harvested by straining through a 70-micron strainer. After centrifugation at 300×*g* for 5 min, single cells were taken up in 1 ml of PBS (with 10% fetal calf serum), counted, and critically assessed for single-cell separation and overall cell viability. Cells were aliquoted in PBS supplemented with 0.04% of bovine serum albumin at a final concentration of 100 cells/μl.

Drop-seq experiments were performed according to the original protocol^72^. Briefly, single cells (100/μl) were co‐encapsulated in droplets with barcoded beads (120/μl, ChemGenes Corporation, Wilmington, MA) at rates of 4000 μl/hr. Droplet emulsions were collected for 10–20 min/each prior to droplet breakage by perfluorooctanol (Sigma‐Aldrich). After breakage, beads were harvested and the hybridized mRNA transcripts were reverse transcribed (Maxima RT, Thermo Fisher). Unused primers were removed by the addition of exonuclease I (New England Biolabs), following which, beads were washed, counted, and aliquoted for pre‐amplification (2000 beads/reaction, equals ca. 100 cells/reaction) with 12 PCR cycles. For each sample, 1 ng of pre‐amplified cDNA from an estimated 1000 cells was tagmented by Nextera XT (Illumina) with a custom P5‐primer (Integrated DNA Technologies). Single‐cell libraries were sequenced in a 100 bp paired‐end run on the Illumina HiSeq4000 using 0.2 nM denatured sample and 5% PhiX spike‐in.

### Single-cell RNA-seq and data analysis

Following sequencing, the generation of count matrices was performed by using the Drop-seq core computational pipeline. The processing of next-generation sequencing reads of the scRNA-seq data was performed as previously described^72^. Briefly, the Drop-seq computational pipeline was used (version 2.3.0, STAR version 2.5.3a) as previously described for the alignment of reads to the mm10 reference genome (provided by the Drop-seq group, GSE63269). For barcode filtering, we excluded barcodes with less than 200 detected genes.

Downstream analysis was performed using the Scanpy package (Version 0.7, https://github.com/theislab/Scanpy)^73^. To gain greater statistical power our newly generated data (FA, 2 m CS, 4 m CS) was processed and annotated together with data previously generated (6 m CS and 6 m CS + treatment) as published by our group (GSE151674).

During preprocessing we assessed the quality of our libraries and applied suitable filter criteria motivated by previously described best practices^74^ with slight adjustments. We kept the top barcodes based on UMI count per cell, guided by the number of estimated cells per sample. After exploration of UMI counts and genes per cell for the combined count matrices, we retained barcodes with count numbers in the range of 400 to 6000 counts per cell and genes detected in at least three cells. A high proportion (>20%) of transcript counts derived from mitochondria-encoded genes may indicate low cell quality, and we removed such unqualified cells. The expression matrices were normalized using scran’s size factor-based approach and log-transformed via scanpy’s pp.log1p() function. In an additional step to mitigate the effects of unwanted sources of cell-to-cell variation, we regressed out the number of UMI counts and cell cycle scores for each cell. Genes variable in at least four samples (11,057 genes) were used as input for principal component analysis (PCA). Clustering was performed via scanpy’s Louvain method at resolution 2 and cell types manually annotated by using known marker genes. The final object encompassed 27,780 genes across 68,256 cells. Finally, we subsetted the data to FA, 2 m CS and 4 m CS only and recalculated highly variable genes, PCA, kNN graph and UMAP for visualization purposes to achieve the best possible low dimensional embedding of the data.

### Fine-grained annotation of the myeloid cells

First, a subset of the final object was generated, containing only myeloid cells. PCA was computed on Genes variable in at least five samples. The first 50 principle components were used to construct a kNN graph (n_neighbors = 20), which was then subjected to graph-based clustering (Louvain algorithm, resolution = 2.0). Cell types were annotated manually based on canonical marker genes from the literature. The complete code for this analysis is reported in the GitHub repository accompanying this manuscript. https://github.com/theislab/2021_PRMT7_regulates_Monocyte_Extravasation

### Cell fate prediction using CellRank

Cell fate probability mapping was restricted to non dendritic cells within the myeloid compartment and was undertaken to utilize the following computation tools: velocyto^75^ version (0.17), scVelo^76^ (version 0.2.3), and CellRank^18^ (version 1.2.0). In brief, spliced and unspliced mRNA counts were quantified with velocyto. RNA velocity vectors were then computed using scVelo’s “dynamical” mode on a per cell basis. Initial and terminal states were then computed using CellRank. Since we observed six cell types in our control condition (AMs, three different IM populations as well as classical and non-classical Monocytes) and one additional cell type, namely CS-induced Macrophages, after exposure to CS, we set the n_states = 6 when computing the initial states and n_states = 7 when computing the terminal states. Based on the inferred initial and terminal states, we then computed the fate probabilities of the initial cell states towards the terminal states. The complete code for this analysis is reported in the GitHub repository accompanying this manuscript. https://github.com/theislab/2021_PRMT7_regulates_Monocyte_Extravasation.

### Lung tissue processing

Right lung lobes were snap-frozen in liquid nitrogen, homogenized and total RNA (peqGOLD Total RNA Kit, Peqlab), and protein isolated. The left lobe was fixed at constant pressure (20 cm fluid column) by intratracheal instillation of PBS buffered 6% paraformaldehyde and embedded into paraffin for histological analysis of hematoxylin and eosin (H&E) stained sections, immunohistochemistry, and immunofluorescence.

For analysis of macrophage infiltration by flow cytometry, single-cell suspensions of right lung tissue were generated. Lung homogenization was performed via enzymatic digestion and mechanical dissociation steps using the lung dissociation kit and gentleMACS Dissociator from Miltenyi Biotec.

### Bronchoalveolar lavage (BAL)

BAL fluid was obtained to perform total and differential cell counts for inflammatory cell recruitment of neutrophils, macrophages, and lymphocytes. Lungs were lavaged by instilling the lungs with 3 × 500 μl aliquots of sterile PBS (Gibco, Life Technologies) supplemented with Complete Protease Inhibitor Cocktail tablets (Roche Diagnostics). For cytospins, cells were pelleted at 400×*g* and resuspended in RPMI-1640 medium (Gibco, Life Technologies). Total cell counts were determined in a hemocytometer. Differential cell counts were performed using morphological criteria on May-Grünwald-Giemsa-stained cytospins (200 cells/sample). BAL fluid was used to evaluate chemokine concentrations of CCL2 and CXCL1 using a commercially available kit for ELISA (eBioscience, Thermo Fisher Scientific).

### Generation of bone marrow-derived macrophages (BMDM)

Bone marrow was flushed from femurs and tibias of *Prmt7*^+/−^ mice and WT littermate controls with RPMI-1640 medium. Bone marrow cells were passed through 40 µm filters (Miltenyi Biotec), counted, and resuspended in 5% fetal bovine serum (Gibco, Life Technologies), 50 μM β-mercaptoethanol, and 100 U/ml penicillin and streptomycin (both Sigma-Aldrich) in RPMI-1640 medium (Gibco, Life Technologies). About 2 × 10^6^ cells/ml were plated in 24-well plates. The medium was additionally supplemented with 20 ng/mL of murine recombinant M-CSF (ImmunoTools). Cells were maintained at 37 °C, 5% CO_2_ for 7 days changing medium every 2–3 days and carefully discarding non-adherent cells. On day 7 fresh medium without M-CSF was added and left overnight. The next day adherent cells were harvested, counted, and seeded at a density of 1 × 10^6^ cells/ml in 24-well plates, and cultured for 24 h in fresh medium to obtain M0 cells, medium containing 1 μg/ml LPS (from Escherichia coli 0111:B4, Sigma-Aldrich) and 20 ng/ml recombinant murine IFNγ (ImmunoTools) for M1 or medium containing 20 ng/ml recombinant murine IL4 (ImmunoTools) for M2. Total RNA was isolated using the peqGOLD Total RNA Kit (Peqlab). In addition, freshly harvested bone marrow and day 7 bone marrow-derived macrophages including those polarized, from WT and *Prmt7*^+/−^ mice were analysed by flow cytometry. Single-cell suspensions were blocked with purified anti-mouse CD16/CD32 (1:100, clone 93, Cat. No. 14-0161-82, eBioscience, Thermo Fisher Scientific) before incubating for 30 min on ice with the following cocktails; VioGreen-conjugated anti-CD45 (1:10, clone: 30F11, Cat. No. 130-102-412, Miltenyi Biotec), PerCP-Vio700-conjugated (1:50, Cat. No. 130-102-422) or PE-Vio770-conjugated anti-F4/80 (1:50, Cat. No. 130-118-459, both clone: REA126, Miltenyi Biotec), PE-conjugated anti-CD11b (1:10, clone: M1/70.15.11.5, Cat. No. 130-091-240, Miltenyi Biotec), APC-conjugated anti-CD11c (1:10, clone: N418, Cat. No. 130-119-802, Miltenyi Biotec), APC-Vio770-conjugated anti-CD80 (1:50, clone: REA983, Cat. No. 130-116-463 Miltenyi Biotec), PerCP-Vio700-conjugated anti-CD86 (1:10, clone: PO3.3, Cat. No. 130-105-137, Miltenyi Biotec) and FITC-conjugated anti-MHC class II (1:50, clone: REA813, Cat. No. 130-112-229, Miltenyi Biotec). Cells were analyzed on a BD FACSCanto II flow cytometer (BD Biosciences) with BD FACSDiva v6.1.3 software.

### Generation of bone marrow-derived dendritic cells (BMDC)

Bone marrow was flushed from femurs and tibias of *Prmt7*^+/−^ mice and WT littermate controls with RPMI-1640 medium. Bone marrow cells were passed through 40 µm filters (Miltenyi Biotec), counted and 10 × 10^6^ bone marrow cells per well were cultured in tissue-culture-treated six-well plates in 4 ml of complete medium (RPMI-1640 supplemented with glutamine, penicillin, streptomycin, 2-mercaptoethanol, 10% heat-inactivated fetal calf serum [all from Gibco]). The cells were treated with 20 ng/ml of GM-CSF (Immunotools). Half of the medium was removed at day 2 and the new medium supplemented with 40 ng/ml of GM-CSF and warmed at 37 °C was added. The culture medium was entirely discarded at day 3 and replaced by a fresh warmed medium with 20 ng/ml of GM-CSF. On day 6, non-adherent cells in the culture supernatant and loosely adherent cells harvested by gentle washing with PBS were pooled and stimulated with 1 μg/ml LPS (from Escherichia coli 0111:B4, Sigma-Aldrich). Total RNA was isolated using the peqGOLD Total RNA Kit (Peqlab). used as the starting source of material for FACS analysis and RNA isolation. BMDCs were also analysed by flow cytometry as described for BMDMs.

### Primary AT2 isolation and trans-differentiation to AT1

Primary AT2 cells (pAT2) were isolated from the lungs of *Prmt7*^+/−^ mice and wild-type littermate controls as previously described^77,78^. In brief, lungs were lavaged with PBS and dispase (BD Bioscience) was instilled for digestion over 45 min at room temperature. Tissue was minced and filtered step-wise through 100, 20, and 10 μm nylon meshes (Sefar). The resultant single-cell suspension was centrifuged for 10 min at 200×*g* and the pellets resuspended in DMEM cell culture medium (Sigma-Aldrich). To isolate the AT2 cells, the cells were first incubated on Petri dishes coated with anti-CD16/CD32 and anti-CD45 antibodies (BD Bioscience) for 30 min at 37 °C to remove leukocytes. Next, fibroblasts were removed by adherence after 25 min to uncoated cell culture dishes. The purity of the AT2 population was routinely assessed by analysis of epithelial (EpCAM, panCK, and pro-SPC), mesenchymal (αSMA and CD90), endothelial (CD31), and hematopoietic cell (CD45) markers by immunofluorescence and flow cytometry. pAT2 cells were resuspended in DMEM cell culture medium supplemented with 10% fetal bovine serum, 10 mM HEPES (both PAA Laboratories), 2 mM l-glutamine, 100 U/ml penicillin and streptomycin (both Life Technologies), and 3.6 mg/ml glucose (Applichem GmbH). Cells were initially cultured for 24 h to attach, medium changed, and cultured at 37 °C, 5% CO_2_ for 5 days, changing medium every other day. To obtain protein, cells were washed twice with PBS, lysed in T-PER lysis buffer (Thermo Fisher Scientific) supplemented with Complete Protease Inhibitor Cocktail tablets (Roche Diagnostics), and lysates centrifuged at 5660×*g* and 4 °C. Supernatants were retained for Western blot analysis.

### Isolation and stimulation of mouse primary monocytes

Bone marrow was flushed from femurs and tibias of wild-type mice with RPMI-1640 medium (Gibco, Life Technologies), and monocytes isolated using the Monocyte Isolation Kit (BM) from Miltenyi as per manufactures instructions. Isolated monocytes were counted and plated into 24-well plates with 5% fetal bovine serum (Gibco, Life Technologies), 50 μM β-mercaptoethanol, and 100 U/ml penicillin and streptomycin (both Sigma-Aldrich) in RPMI-1640 medium (Gibco, Life Technologies). The seeded cell number per well was around 4 × 10^5^ in a 500 µl medium. The monocytes were then pretreated with 1 µM of BAY11-7082, NF-κB inhibitor, and 20 µM of JSH-23, RELA translocation inhibitor, separately for 1 h at 37 °C, 5% CO_2._ After 1 h pretreatment, 100 ng/ml LPS (*E. coli* O55:B5, Sigma-Aldrich) was added into plates to stimulate the cells for 6 and 24 h. Control plates received DMSO instead of inhibitors in the same amount. The treatment was stopped by adding RNA lysis buffer into the plates for RNA isolation and RIPA buffer for protein isolation.

### Isolation of primary alveolar macrophages from mouse lungs

BAL fluid was obtained to isolate alveolar macrophages from the lungs. To do so, lungs were lavaged by instilling the lungs with 10x1ml aliquots of sterile PBS (Gibco, Life Technologies) supplemented with Complete Protease Inhibitor Cocktail tablets (Roche Diagnostics). BAL fluid was centrifuged at 400×g for 20 min at 4 °C. BAL cells were suspended in complete RPMI-1640 medium supplemented with 10% FBS, 50 µM β-mercaptoethanol, and 100 U/ml penicillin and streptomycin (all from Gibco, Life Technologies). About 5 × 10^4^ cells in 1 ml were seeded in 24-well plates and allowed to adhere for 1–4 h. Non-adherent cells were removed by washing twice with PBS. The attached cells into the plate were collected and they were frozen for further experiments. In our case, RNA lysis buffer was added into alveolar macrophages for RNA isolation.

### Quantitative real-time RT-PCR

cDNA was synthesized from 1 μg total RNA using Random Hexamers and MuLV Reverse Transcriptase (Applied Biosystems). mRNA expression was analyzed using Platinum SYBR Green qPCR SuperMix (Applied Biosystems) on a StepOnePlus™ 96 well Real-Time PCR System (Applied Biosystems) running StepOne software v2.3 (Applied Biosystems). Primers were designed using Primer-BLAST software (https://www.ncbi.nlm.nih.gov/tools/primer-blast/) or obtained from PrimerBank^79^ (https://pga.mgh.harvard.edu/primerbank/). Primer sequences are listed in Supplementary Table 4. Relative expression of each gene was calculated relative to the housekeeping gene *HPRT1* or *Hprt1* as 2^−ΔCt^.

### Immunohistochemistry

Three-μm-thick sections from mouse left lung were deparaffinized in xylene and rehydrated before treatment with 1.8% (v/v) H_2_O_2_ solution (Sigma-Aldrich) to block endogenous peroxidase. Heat-induced epitope retrieval was performed in HIER citrate buffer (pH 6.0, Zytomed Systems) in a decloaking chamber (Biocare Medical). To inhibit nonspecific binding of antibodies, sections were treated with rodent blocking buffer (Biocare Medical). After overnight incubation with a primary antibody against Galectin 3 (Rabbit anti-Galectin 3, 1:100, Cat. No. sc-20157, Santa Cruz), CD68 (Rabbit polyclonal, Cat. No. ab125212, 1:800, Abcam), ACSL4 (Mouse monoclonal, Cat.No. sc-365230, 1:50, Santa Cruz), ALOX5 (Mouse monoclonal, Cat.No. sc-136195, 1:50, Santa Cruz), and Pro-SPC (Rabbit polyclonal, Cat.No. AB3786, 1:100, Sigma-Aldrich) at 4 °C, sections were incubated with an alkaline phosphatase (AP)- or horseradish peroxidase (HRP)-conjugated secondary antibody (Rabbit-on-Rodent AP-Polymer Cat. No. RMR625H, Rabbit-on-Rodent HRP-Polymer Cat. No. RMR622H, Mouse-on-Mouse AP-Polymer Cat. No. MM624H, all Biocare Medical) at room temperature for 1 h. Signals were amplified by adding chromogen substrate Vulcan fast red or 3,3′-diaminobenzidine (DAB) (Biocare Medical), respectively. Sections were counterstained with hematoxylin (Sigma-Aldrich) and dehydrated in xylene and mounted with Entellan (Merck). Histology images were acquired using Zeiss Mirax Microimaging slide scanner running MIRAXDESK v1.12.25.1 and Mirax viewer v1.12.22.0 software (Zeiss, 3D Histech).

### Masson Trichrome staining

Masson Trichrome staining is used for the detection of collagen fibers in lung tissues. Three-micrometer sections from paraffin-embedded lung were deparaffinized in the Xylol for 5 min (2 x) followed by 100% ethanol for 2 min (2 x). They were rinsed in running tap water followed by distilled water. For nuclear staining, the slides were stained with hemalumn and then put into the ethanol (70%) + HCl (0.1%) mix and washed with tap water. The slides were kept in Ponceau Fuschin staining followed by Phosphomolybdic acid and Light Green staining. To remove the staining, the slides were washed with 100% ethanol three times quickly. Before mounting, the slides were placed in ethanol 100% for 1 min (2 x), xylol for 1 min (2 x) for clearance. Sections were mounted with Entellan (Merck). Histology images were acquired using Zeiss Mirax Microimaging slide scanner running MIRAXDESK v1.12.25.1 and Mirax viewer v1.12.22.0 software (Zeiss, 3D Histech).

### Immunofluorescence

Three-μm sections from paraffin-embedded human core samples or mouse left lung were deparaffinized and rehydrated, followed by heat-induced epitope retrieval using HIER citrate buffer (pH 6.0, Zytomed Systems). Sections were blocked with 5% BSA for 30 min, incubated overnight at 4 °C with primary antibodies (mouse anti-Galectin 3, 1:50, Cat. No. sc-32790, Santa Cruz; rabbit anti-PRMT7, 1:50, Cat. No. sc-98882, Santa Cruz;) and 1 h at room temperature with secondary antibodies (anti-mouse Alexa Fluor 488, 1:500, Cat. No. A28175 and anti-rabbit Alexa Fluor 568, 1:500, Cat. No. A11011, Life Technologies) diluted in 1% BSA. Nuclei were counterstained with DAPI and the slides were mounted in a fluorescent mounting medium (Dako). Images were captured using Axioimager with an M2 microscope (Carl Zeiss) driven by Zen 2.3 (“blue version”) software (Carl Zeiss).

### Multiplex immunofluorescence staining

Sequential immunostaining was performed on 3-μm-thick Formalin-Fixed Paraffin-Embedded (FFPE) murine left lung sections as previously described^80^. Briefly, heat mediated antigen retrieval was performed in Universal HIER antigen retrieval reagent (Abcam, #ab208572). Antibody elution was performed between each staining cycle. Primary antibodies used were: rabbit anti-5-Lipoxygenase/5-LO (1:500, Abcam #ab169755), rabbit anti-Myeloperoxidase (1:1000, Abcam #ab208670), rabbit anti-IBA1 (1:1000, VWR #100369–764), rabbit anti-iNOS (1:250, Abcam #ab239990), and rabbit anti-CD206 (1:250, Abcam #ab64693). Secondary antibodies used were: anti-rabbit 555 (1:500, Cell Signaling #4413 S) and anti-rabbit 647 (1:1000, Cell Signaling #4414 S).

Acquired images were processed using FIJI and the FIJI plugin HyperStackReg V5.6^81^ (and Ved Sharma. 2018, December 13 Zenodo. 10.5281/zenodo.2252521). Autofluorescence acquired in nonrelevant channels was substracted as appropriate. Quantitation was performed using Ilastik (v1.3.3post3)^82^ and CellProfiler (4.1.3)^83^.

### Quantitative morphometry

Design-based stereology was used to analyze sections using an Olympus BX51 light microscope equipped with a computer-assisted stereological toolbox software Visopharm Integrator System (VIS) v6.0.0.1765 (newCAST, Visiopharm) on H&E, Galectin 3 or CD68 immunohistochemistry-stained lung tissue sections as previously described^2^. Airspace enlargement was assessed by quantifying mean linear chord length (MLI) on 30 fields of view per lung. Briefly, a line grid was superimposed on lung section images taken with the ×20 objective. Intercepts of lines with alveolar septa and points hitting airspace were counted to calculate MLI applying the formula MLI = ∑P_air_ × L(p)/∑I_septa_ × 0.5. P_air_ are the points of the grid hitting air spaces, L(p) is the line length per point, and I_septa_ is the sum of intercepts of alveolar septa with grid lines. To determine the number of macrophages across the lung, a frame grid was superimposed on lung section images taken with the ×40 objective, and Galectin 3 positive macrophages were counted across at least 20 random fields per lung. To quantify collagen deposition in the lung sections, a line grid was superimposed on lung section images taken with the ×40 objective. Intercepts of lines crossing with airways and vessels were counted to calculate collagen (µm^3^/µm^2^) = ∑P_fibrotic area_ × L(p)/∑I_intercept(A+V)_. P_fibrotic area_ are the points of the grid hitting fibrotic area which were stained as green by Masson trichrome staining, L(p) is the line length per point (9.79 μm as we measured), and I_septa_ is the sum of intercepts of airways and vessels to normalize the collagen deposition.

### Flow cytometry analysis of the whole lung

For flow cytometric analysis of single-cell suspensions, 10^6^ cells were blocked with purified anti-mouse CD16/CD32 (1:100, clone 93, Cat. No. 14-0161-82, eBioscience, Thermo Fisher Scientific) before incubating for 30 min on ice with the following cocktail; VioGreen-conjugated anti-CD45 (1:10, clone: 30F11, Cat. No. 130-102-412, Miltenyi Biotec), PerCP-Vio700-conjugated anti-F4/80 (1:50, clone: REA126, Cat. No. 130-102-422, Miltenyi Biotec), PE-conjugated anti-CD11b (1:10, clone: M1/70.15.11.5, Cat. No. 130-091-240, Miltenyi Biotec), and APC-conjugated anti-CD11c (1:10, clone: N418, Cat. No. 130-119-802, Miltenyi Biotec). Cells were analyzed on a BD FACSCanto II flow cytometer (BD Biosciences) with BD FACSDiva v6.1.3 software.

### Monocyte transendothelial migration

Transwell 24-well inserts with a 5.0 µm pore size (Permeable Polycarbonate Membrane Inserts, Corning, Fisher Scientific) were coated with 4 mg/ml collagen G (Biochrom). About 1 × 10^6^/ml SVEC4-10 (ATCC CRL-2181) endothelial cells were seeded onto the insert in 200 ul DMEM High glucose plus GlutaMAX medium (Gibco, Life Technologies) supplemented with 10% fetal bovine serum (Gibco, Life Technologies) and 100 U/ml penicillin and streptomycin (Sigma-Aldrich). About 600 ul of the medium was added to the lower wells and cells cultured for 48 h at 37 °C, 5% CO_2_. Endothelial cells were then activated with 10 ng/ml TNF (PeproTech) for 4 h.

Bone marrow was flushed from femurs and tibias of *Prmt7*^+/−^ mice and wild-type littermate controls with RPMI-1640 medium (Gibco, Life Technologies), and monocytes isolated using the Monocyte Isolation Kit (BM) from Miltenyi. The medium was discarded from the inserts and wells, and 600 ul serum-free RPMI-1640 medium was added to the wells ±  100 ng/ml CCL2 (R&D Systems). Monocytes were transferred to the endothelial lined inserts at 1 × 10^6^/ml in 200 ul serum-free RPMI-1640 medium, and cultured for 4 h at 37 °C, 5% CO_2_. The number of migrated monocytes in each well was then counted using BD Trucount Tubes analyzed on a BD FACSCanto II flow cytometer with BD FACSDiva v6.1.3 software (all BD Biosciences).

In addition, isolated monocytes from *Prmt7*^+/−^ mice and wild-type littermate controls were blocked with purified anti-mouse CD16/CD32 (1:100, clone 93, Cat. No. 14-0161-82, eBioscience, Thermo Fisher Scientific) before incubating for 30 min on ice with PE-conjugated anti-CCR2 (1:100, clone 475301, Cat. No. FAB5538P, R&D Systems) and analyzed on a BD FACSCanto II flow cytometer with BD FACSDiva v6.1.3 software.

### CRISPR/Cas9 targeted knockout of *Prmt7* in MH-S cells

Double-stranded CRISPR guide RNAs, guide A 5′-CACCGCACTCCAGGGATCCCGTGGTAGG-3′ and guide B 5′-CACCGTGAACACTATGATTACCACCAGG-3′ targeting exon 2 of *Prmt7* (Extended Data Fig. 3a) were both cloned using *BbsI* restriction sites into pX335-U6-Chimeric_BB-CBh-hSpCas9n(D10A) (Addgene plasmid #42335)^84^. The double-stranded DNA sequence 5′-TGTGGCCGTGCCAATCCTACCACGGGATCCCTGGAGTGGCTGGAGGAGGATGAACACTATGATTACCACCAGGAGATTGCCAGGTCAT-3′ was cloned using *EcoRV* restriction sites into pARv-RFP (Addgene plasmid #60021)^85^, to be used as a reporter plasmid to detect CRISPR/Cas9 activity in transfected cells.

The macrophage MH-S cell line (CRL-2019, ATCC) was seeded at 1.2 × 10^5^ cells/well in 24-well plates in 500 ul of RPMI-1640 medium (Gibco, Life Technologies) supplemented with 10% fetal bovine serum (Gibco, Life Technologies), 50 μM β-mercaptoethanol and 100 U/ml penicillin and streptomycin (both Sigma-Aldrich) and cultured at 37 °C in 5% CO_2_. Cells were then transfected with 375 ng pX335.guide A, 375 ng pX335.guide B, and 125 ng pARv-RFP with the Xfect^TM^ Transfection Reagent (Clontech) according to the manufacturer’s instructions. Cells were then cultured in full medium for a further 48 h with a medium change after 24 h. RFP positive cells were single-cell sorted into 96-well plates, along with negatively stained controls, using a BD FACS Aria III (BD Biosciences). Wells containing single colonies were expanded, genomic DNA isolated (DNeasy Blood and Tissue Kit, Qiagen), and amplified using primer pair: 5′-TGCCTTCCAGACCTGAGATTG-3′ and 5′-CCTAACAGAGACCTCAACTGC-3′ for sequencing (Eurofins Medigenomix GmbH) and agarose gel analysis.

### MH-S cell culture

Wild-type *Prmt7*^+/+^ and *Prmt7*^null^ clone MH-S macrophage cells were maintained in RPMI-1640 medium (Gibco, Life Technologies) supplemented with 10% fetal bovine serum (Gibco, Life Technologies), 50 μM β-mercaptoethanol, and 100 U/ml penicillin and streptomycin (both Sigma-Aldrich) at 37 °C in 5% CO_2_. Total RNA was isolated using the peqGOLD Total RNA Kit (Peqlab). To obtain protein, cells were washed with PBS and scrapped into ice-cold RIPA buffer (50 mM Tris HCl pH 7.4, 150 mM NaCl, 1% Triton X-100, 0.5% sodium deoxycholate, 1 mM EDTA, and 0.1% SDS) supplemented with protease and phosphatase inhibitor cocktails (Roche Diagnostics), incubated on ice for 30 min and centrifuged for 15 min at 4 °C and 13000 rpm. Supernatants were retained for Western blot analysis.

For cell adhesion assays, *Prmt7*^+/+^ and *Prmt7*^null^ clone MH-S cells were seeded at 1 × 10^5^ cells per well in 24-well plates, and unattached cells remaining in the medium at the given time point were counted using BD Trucount Tubes analyzed on a BD FACSCanto II flow cytometer with BD FACSDiva v6.1.3 software (all BD Biosciences).

For wound healing assays, cells were seeded at 1 × 10^5^ cells per well in 24-well plates, a scratch was induced 24 h after seeding (time point 0 h), cells were washed and cultured in full medium for 24 h. Wound closure was determined at 24 h using AxioVision software (v4.9.1.0, Zeiss).

For WST-1 cell proliferation assays, cells were seeded at 1 × 10^4^ cells per well in 96-well plates and WST-1 reagent (Sigma-Aldrich) added at 1:10 dilution at set time periods and left for 1 h incubation at 37 °C in 5% CO_2_ before the absorbance was read at 450 nm.

Additionally, *Prmt7*^+/+^ and *Prmt7*^null^ clone MH-S cells were seeded at 4 × 10^5^ cells per well in six-well plates and 24 h later stimulated with 100 ng/ml CCL2 (R&D Systems) or 1 μg/ml LPS (*E. coli* O55:B5, Sigma-Aldrich) in RPMI-1640 medium supplemented with 1% fetal bovine serum. The reaction was halted by washing with ice-cold PBS and protein obtained by scrapping into ice-cold RIPA buffer supplemented with protease and phosphatase inhibitor cocktails as described above.

Further, *Prmt7*^+/+^, *Prmt7*^null^, *Prmt7*^+/+^ MH-S cells were treated with 5 μM SGC3027 for 24 h (PRMT7 inhibitor^27^, Structural Genomics Consortium) and *Prmt7*^+/+^ MH-S cells treated with 20 μM GGTI for 2 h and analysed 6 h later (GGTI 298 trifluoroacetate salt hydrate, RAP1 inhibitor^33^, Sigma-Aldrich) were analysed by flow cytometry. Single-cell suspensions were blocked with purified anti-mouse CD16/CD32 (1:100, clone 93, Cat. No. 14-0161-82, eBioscience, Thermo Fisher Scientific) before incubating for 30 min on ice with PE-conjugated anti-CD11b (ITGAM, 1:10, clone: M1/70.15.11.5, Cat. No. 130-091-240, Miltenyi Biotec) and FITC-conjugated anti-CD11a (ITGAL, 1:100, clone: M17/4, Cat. No. 11-0111-82, eBioscience/Thermo Fisher Scientific). Cells were analyzed on a BD FACSCanto II flow cytometer (BD Biosciences) with BD FACSDiva v6.1.3 software.

Lastly, wild-type MH-S cells were treated with 7.5 μM SGC3027, 1 µM of MS023 (Type I inhibitor which blocks Rme2a^28^, Sigma-Aldrich), and 0.2 µM of EPZ015666 (PRMT5 inhibitor to block Rme2s^29^, Sigma-Aldrich) for 48 h. Total RNA and protein was isolated as above and cells were also analysed by flow cytometry for ITGAM and ITGAL expression as just described.

### Anti-mono-methylated arginine IP followed by LC-MS/MS proteome analysis

About 50 ul Pierce Protein A Agarose beads (Thermo Fisher Scientific) were crosslinked with 4 μg anti-mono-methylated arginine antibody (Cat. No. 8015, Cell Signaling Technology) per IP. About 50 ug of protein from wild-type and *Prmt7*^null^ clone MH-S cells was first precleared with Pierce Protein A Agarose beads, then incubated with the antibody-coated beads at 4 °C for 18 h under rotation. Beads were pelleted at 14,000 rpm for 1 min at 4 °C and washed 3x with PBS. Three IPs were undertaken for each cell type and processed as described^86^.

The samples were measured on a Q-Exactive Plus mass spectrometer (Thermo Fisher) coupled to a Proxeon nano-LC system (Thermo Fisher) in data-dependent acquisition mode, selecting the top ten peaks for HCD fragmentation. A 3-h gradient (solvent A: 5% acetonitrile, 0.1% formic acid; solvent B: 80% acetonitrile, 0.1% formic acid) was applied for the samples using an in-house prepared nano-LC column (0.075 mm × 250 mm, 3-μm Reprosil C18, Dr. Maisch GmbH). A volume of 5 μl sample was injected and the peptides were eluted with 3 h gradients of 4 to 76% ACN and 0.1% formic acid in water at flow rates of 0.25 μl/min. MS acquisition was performed at a resolution of 70,000 in the scan range from 300 to 1700 m/z. Dynamic exclusion was set to 30 s and the normalized collision energy to 26 eV. The mass window for precursor ion selection was set to 2.0 m/z.

Quantitative ratios were calculated and normalized by the Max Quant software package (version 1.6.12.0). LFQ intensity values supplied in Supplementary Data File 3 and a list of all detected peptides supplied in Supplementary Data File 4. InCroMAP software (http://www.ra.cs.uni-tuebingen.de/software/InCroMAP/downloads/index) Version 1.7.0 (University of Tübingen, Germany)^87^ was used to analyze for KEGG pathway enrichment in the differentially pulled down proteins with less abundance in *Prmt7*^null^ compared to WT MH-S cells (FC >−1.6), see Supplementary Data File 5. Heat maps of enriched pathways were generated by Genesis software (Release 1.7.7, Institute for Genomics and Bioinformatics, Graz University of Technology). Protein information was obtained from UniProtKB (http://www.uniprot.org).

### MLE12 cell culture and siRNA knockdown of *Prmt7*

The murine AT2 cell line MLE12 (CRL-2110, ATCC) was maintained in DMEM/F12 medium (Gibco, Life Technologies) supplemented with 10% fetal calf serum (Gibco, Life Technologies) and 100 U/ml penicillin-streptomycin (Sigma-Aldrich) at 37 °C in 5% CO_2_ atmosphere. For siRNA knockdown of *Prmt7*, cells were seeded at 4 × 10^4^ cells per well in a full medium in 24-well plates for 24 h and then transfected with 50 nM FlexiTube siRNA Mm_BC006705_4 or Mm_BC006705_5 (Qiagen) using HiPerFect Transfection Reagent according to the manufacturer’s instructions (Qiagen). Cells were then cultured in full medium for 48 h with a medium change after 24 h. A scratch was induced (time point 0 h), cells were washed and cultured in full medium for 24 h. Wound closure was determined at 6 and 24 h using AxioVision software (v4.9.1.0, Zeiss). Total RNA was then isolated using peqGOLD Total RNA Kit (Peqlab).

### Raw264.7 culture and siRNA-mediated knockdown of *Alox5*

Raw264.7 cells were maintained in DMEM/F12 medium (Gibco, Life Technologies) supplemented with 10% fetal calf serum (Gibco, Life Technologies) and 100 U/ml penicillin-streptomycin (Sigma-Aldrich) at 37 °C in a 5% CO_2_ atmosphere. For siRNA knockdown of *Alox5*, cells were seeded at 4 × 10^4^ cells per well in a full medium in 24-well plates for 24 h and then transfected with 50 nM FlexiTube siRNA (Qiagen) using HiPerFect Transfection Reagent according to the manufacturer’s instructions (Qiagen) and were cultured in FCS low medium (1%). The medium was exchanged the next day by full medium supplemented with polarizing stimulators. Knockdown efficiency was performed after 48 h post-transfection by isolating RNA using peqGOLD Total RNA Kit (Peqlab) and specific primer targeting *Alox5*.

### CRISPR/Cas9-mediated knockout of *Acsl4* and *Prmt7* in MLE12 cells

Single sgRNA guides targeting the critical exon of mouse *Acsl4* (TATCCAGAGTATCTGCTCCA; AAGTGTGTGACAGAGCGATA) and two critical exons of mouse *Prmt7* (GTCATGTAGCATGTCGGCAT; TGGCCGTGCCAATCCTACCA) plus single non-targeting sgRNA guides (TTTACTCATATCCAGTCAC; ATCTAGTCCTCTAATCGAT) as negative control were generated and cloned into the *BsmBI*-digested lentiCRISPRv2 blast and lentiCRISPRv2 puro lentiviral vectors (a gift from Brett Stringer; Addgene #98293, #98290). For the production of replication-incompetent ecotropic viral particles, HEK293T cells at 70% confluence were co-transfected with one of the lentiviral vectors and the packaging vectors—pEcoEnv-IRES-puro, pMDLg_pRRE, and pRSV_Rev (a kind gift from Prof. Timm Schroeder, ETH Zurich)^88^—in a fixed molar ratio (5:2:10:5; total 3 μg DNA) using X-tremeGene HP DNA Transfection reagent in a ratio of 1:3 (DNA: reagent). At 48 h post-transfection, the supernatants containing ecotropic lentiviral particles were filtered using a sterile 45 μm low protein binding syringe filter. MLE12 cells were trypsinized, seeded on a six-well plate, and infected with an equal amount of the viral supernatants harboring either blasticidin or puromycin resistance in the presence of 7.5 μg/ml protamine sulfate to enhance lentiviral transduction. At 48 h post-infection 10 μg/ml of blasticidin and 2 μg/ml puromycin were added to the cells to select a pool of *Acsl4* and *Prmt7* knockout cells. ACSL4 expression was analyzed by Western blotting, loss of PRMT7 expression and activity was confirmed by Western blotting.

### CRISPR/Cas9-mediated knockout of *Prmt7* in Raw264.7 cells

Single sgRNA guides targeting the two critical exons of mouse *Prmt7* (GTCATGTAGCATGTCGGCAT; CACTCCAGGGATCCCGTGGT) and single non-targeting sgRNA guides (TTTACTCATATCCAGTCAC; ATCTAGTCCTCTAATCGAT) as negative control were generated and cloned into the *BsmBI*-digested lentiCRISPRv2 blast and lentiCRISPRv2 puro lentiviral vectors (a gift from Brett Stringer; Addgene #98293, #98290). The establishment of a knockout cell line was undertaken as described for MLE12 cells with exception of using two different packaging vectors—psPAX2, pMD2.G, and the loss of PRMT7 expression and activity confirmed by Western blotting.

### Proximity ligation assay

About 3 × 10^4^ *Prmt7*^+/+^ and *Prmt7*^null^ Raw264.7 cells per well were seeded onto Millicell EZ slide from Merck 1 day prior to the experiment. On the next day, cells were fixed in ice-cold methanol for 2 min, washed twice in PBS, and incubated with blocking solution supplied from the PLA kit (Sigma, DUO92101) for 1 h at 37 °C. During the reactions, all incubations were performed in a humidified chamber. A reaction for 1 cm^2^ requires ~40 µl. After blocking, cells were incubated with primary antibodies against mono-methylated arginine (Cat. No. 8015, 1:100, Cell Signaling Technology), Total H3 (Cat. No. ab195277, 1:100, Abcam) and RAP1A/B (Cat. No. ab175329, 1:50, Abcam) for 1 h at 37 °C. After three washes in Buffer A, PLA probes (PLUS and MINUS from PLA kit, Sigma, DUO92101) containing the secondary antibody conjugated with complementary oligonucleotides were added and incubated for 1 h at 37 °C. Cells were washed three times in Buffer A and incubated in T4 DNA ligase diluted in ligase buffer for 30 min at 37 °C. After three washes in Buffer A, cells were incubated in DNA polymerase diluted in polymerase buffer with red fluorescent-labeled oligonucleotides for 100 min at 37 °C. Washes were performed twice for 10 min in 1X Buffer B, then 1 min in 0.01X Buffer B. The slides were mounted using a 7 µl volume of Duolink in situ mounting medium containing DAPI. Slides were kept at 4 °C and visualized the next day. Images were acquired under identical conditions using an inverted microscope stand with an LSM710 (Carl Zeiss) confocal module operated in multitrack mode using Plan-Apochromat W 63×/1.3 M27 objective. The microscope was driven by ZEN2009 (Carl Zeiss) software, version 5.5. Image acquisition was performed by imaging DAPI stained at a fixed Z position. Quantification of the samples were performed using ImageJ software (v1.50a). Random pictures were taken from three different fields and dots representing the signal and nuclei were counted in each field. The total number of dots were divided into cell number and the average dot number per cell was calculated.

### Assessment of lipid peroxidation using C11-BODIPY (581/591)

A total of 150,000 cells per well were seeded on 12-well plates 1 day prior to the experiment. On the next day, cells were treated with the conditional medium collected from differentially polarized macrophages or (*1* *S,3* *R*)-RSL3^54^ to induce ferroptosis for 6 h. Cells were incubated with C11-BODIPY (581/591) (1 μM, Invitrogen) for 20 min at 37 °C before they were washed with PBS and harvested by trypsinization. Subsequently, cells were resuspended in 100 μl fresh PBS and analyzed using the 488-nm laser of flow cytometer (FACSCanto II, BD Biosciences) for excitation. At least 10,000 events were analyzed per sample and data was collected from the FL1 detector (C11-BODIPY) with a 502LP and 502/30 BP filter. Data were analyzed using FlowJo Software (v8.4.1).

### Cell viability assay using the Aquabluer method

Cells were seeded on 96-well plates (20,000 cells per well) and treated with the conditional medium from polarized macrophages, (*1* *S,3* *R*)-RSL3^54^, LPS (1 μg/ml), IFNg (20 ng/ml), IL4 (20 ng/ml), or liproxstatin-1 (250 nM)^54^. Cell viability was assessed 24 h after treatment using AquaBluer as an indicator of viable cells according to the manufacturer’s recommendations (MultiTarget Pharmaceuticals, LLC).

### ELISA for leukotriene B4 (LTB4) and lipoxygenase activity assay

LTB4 concentrations in BAL fluid from mice and cell culture supernatants were determined using the LTB4 Parameter Assay Kit (R&D Systems) according to the manufacturer’s instructions. Lipoxygenase activity was determined in cell culture supernatants using the Lipoxygenase Assay Kit (Abcam) according to the manufacturer’s instructions.

### Live-cell microscopy

Cells were seeded on eight-well μl-slides (Ibidi, Germany) at a density of 20,000 cells per well and incubated overnight. On the next day, propidium iodide (1 μg/ml) was added to each well, and cells were treated with conditional medium from polarized macrophages, RSL3 (250 nM) in combination with zVAD, Nec1-s. Live-cell imaging was performed using an Axio Observer Z1 imaging system (Visitron Systems, Puchheim, Germany), equipped with LED excitation light (pE-4000, CoolLED, Andover, UK) for fluorescence illumination. For phase-contrast images, exposure time of 10 ms was used. For PI excitation, the LED module 550 nm (exposure time 400 ms) and Fluorescence filters ET-Cy3 (Chroma, US) were used. Images were obtained using a ×20 air objective (EC Plan-Neofluar 20x/0,5 Ph2) and acquired with a CCD camera (CoolSnap ES2, Photometrics, Tucson, AZ, USA) and the imaging software VisiView 4.0 (Visitron Systems, Puchheim, Germany). During the imaging overnight, cells were maintained at 37 °C and 5% CO_2_ conditions under a controlled incubation chamber (ibidi heating system with ibidi gas incubation system, ibidi, Germany).

### SVEC4-10 cell culture and stimulation with SGC3027 drug, PRMT7 inhibitor

SVEC4-10 endothelial cells were maintained in Dulbecco’s Modified Eagle Medium (DMEM) (Gibco, Life Technologies) supplemented with 10% fetal bovine serum (Gibco, Life Technologies) and 100 U/ml penicillin and streptomycin (Sigma-Aldrich) at 37 °C in 5% CO_2_. For stimulation of SVEC4-10 cells, the first 2 × 10^5^ cells per ml were seeded into 24-well plates with DMEM medium supplemented with 10% fetal bovine serum for 24 h to allow them to recover. The next day, treatment with 5 µM of SGC3027 drug and 100 ng/ml TNF (PeproTech) was applied with DMEM medium with 1% fetal bovine serum for 24 h. The reaction was stopped by adding RNA lysis buffer.

### Western blotting

Protein concentrations were determined using the Pierce BCA Protein Assay Kit (Thermo Fisher Scientific). About 20 μg of protein was separated by SDS-PAGE, transferred onto a polyvinylidene difluoride membrane (Bio-Rad), blocked with 5% nonfat milk, and immunoblotted overnight at 4 °C with antibodies against PRMT7 (Cat.No. NBP2-26135, 1:1000, Novus Biologicals), PRMT6 Cat. No. 14641, 1:1000, Cell Signaling Technology), mono-methylated arginine (Cat. No. 8015, 1:1000, Cell Signaling Technology), T1-alpha (Cat. No. AF3244, 1:1000, R&D Systems), VCAM-1 (Cat.No. ab134047, 1:10000, Abcam), RAP1A/B (Cat.No. ab187659, 1:10000, Abcam, phosphorylated ERK1/2 (Cat. No.4370 S, 1:2000, Cell Signaling Technology, total ERK1/2 (Cat.No. 4695 S, 1:1000, Cell Signaling Technology, phosphorylated p38 (Cat.No. 4631 S, 1:1000, Cell Signaling Technology, H3R2me1 (Cat.No. ab176844, 1:500, abcam, H3R2me2 (Cat.No. 04-808, 1:500, Merck Millipore), Total H3 (Cat. No. ab1791, 1:1000, Abcam), ACSL4 (Cat.No. sc-271800, 1:250, Santa Cruz), ALOX5 (Cat.No. sc-136195, 1:500, Santa Cruz) and GPX4 (Cat.No. ab125066, 1:1000, Abcam). Antibody binding was detected with HRP-conjugated secondary antibodies (1:2500, Goat anti-Rabbit IgG H&L (HRP), Cat. No. ab6721, Abcam; 1:4000, Rabbit anti-Goat IgG-HRP, Cat. No. sc-2768, Santa Cruz; 1:3000, Sheep anti-Mouse IgG-HRP, Cat. No. NA931VS, Amersham, GE Healthcare Life Sciences) and developed using Amersham ECL Prime reagent (GE Healthcare). Bands were detected and quantified using the Chemidoc XRS system+ running ImageLab v5.2.1 software (Bio-Rad), and normalized to β-actin levels (anti-β-actin-peroxidase-conjugated mouse monoclonal antibody, AC-15, Cat. No. A3854, 1:50000, Sigma-Aldrich). Uncropped and unprocessed scans can be found in the source data file.

### ChIP-qPCR

A total of 2 × 10^7^ fixed wild-type and *Prmt7*^null^ MH-S macrophage cells were sent to Active Motif (Carlsbad, CA) for ChIP reactions using anti-H3R2me1 (Cat. No. Ab176844, Abcam) and anti-H3R2me2s (Cat. No. ABE460, Millipore). Cells were fixed following sample preparation guidelines supplied by the company. Primers used for ChIP-qPCR are listed in Supplementary Table 5. All ChIP data were normalized to total input.

### ATAC-Seq analysis

About 100,000 wild-type and *Prmt7*^null^ MH-S macrophage cells were sent to Active Motif (Carlsbad, CA) for ATAC-Seq analysis, after cryopreserving the cells as described in the ATAC-Seq sample preparation guidelines supplied by the company. Briefly, barcoded libraries were sequenced 42 bp paired-end on NextSeq 500 (Illumina). The paired-end 42 bp sequencing reads (PE42) were mapped to the genome using the BWA algorithm with default settings. Only reads that pass Illumina’s purity filter, align with no more than two mismatches, and map uniquely to the genome were used in the subsequent analysis. In addition, duplicate reads (“PCR duplicates”) are removed. Genomic regions with high levels of transposition/tagging events were determined using the MACS2 peak-calling algorithm^89^. Since both reads (tags) from paired-end sequencing represent transposition events, both reads are used for peak-calling but treated as single, independent reads. To identify the density of transposition events along the genome, the genome is divided into 32 bp bins and the number of fragments in each bin is determined. For this purpose, reads were extended to 200 bp, which is close to the average length of the sequenced library inserts. In silico extension also helps to smooth the data. The ATAC-Seq Tracks were visualized in the UCSC Genome Browser (https://genome.ucsc.edu/). The ATAC-seq Track across the TSS of *Prmt7* in WT cells was further analysed by JASPAR^21,90^ http://jaspar.genereg.net for transcription factor binding sites.

### Gene set enrichment analysis (GSEA)

GSEA software (v4.0.1) from the Broad Institute (http://www.gsea-msigdb.org/gsea/index.jsp)^91,92^ was used to determine the enrichment of gene lists from the GO Molecular Function collection in transcriptomics data obtained from series matrix files downloaded from the NCBI GEO database GSE76925^9^, which contained lung transcriptomics data from 111 COPD patients and 40 smoker controls (Supplementary Data Files 1, 2). After collapsing into gene symbols, there were 24,776 genes. Gene set size filters of min = 15 and max = 500 were employed resulting in 642 gene sets of the original 901 from the GO Molecular Function collection being used in the analysis. GSEA software was also used to determine enrichment of cell death gene lists on transcriptomics data obtained from series matrix files downloaded from the NCBI GEO database from lung tissue of COPD patients (GSE47460-GPL14550; 145 COPD patients v 91 healthy controls) and mice exposed to 4 m chronic cigarette smoke (*n* = 3) v filtered air (*n* = 3) (GSE125521) (Supplementary Data Files 6, 7). Heat maps of enriched pathways were generated by Genesis software (Release 1.7.7, Institute for Genomics and Bioinformatics, Graz University of Technology).

### GWAS and eQTL analysis

The GWAS Central database (https://www.gwascentral.org)^10^ was screened to identify SNPs within the *PRMT7* locus that associated (−log *p* > 2) with an altered lung function (FEV1 or FEV1/FVC) in the GWAS study by Soler Artigas et al.^11^ that was composed of over 48,000 individuals. Three SNPs met these criteria (rs3785115, rs9928605, and rs11075672) and were analyzed in the GTEx Portal database (https://gtexportal.org)^12^ to identify if they were eQTL in the lung and blood of individuals that carried them. Data were downloaded as eQTL box plots of rank normalized gene expression in people homozygous for reference sequence, heterozygous or homozygous for the SNP, displaying normalized effect size and *P* value, with median, first and third quartiles, and 1.5 interquartile range of first and third quartiles highlighted.

### PRMT7 expression in human lung published data sets

Series matrix files from the NCBI GEO database for GSE76925^9^ and GSE27597^93^ were downloaded and gene expression normalized to control groups using Microsoft Excel v 14.0.7237.5000 (part of Microsoft Office Professional Plus 2010). The Human Protein Atlas database https://www.proteinatlas.org^17^ was also accessed to examine lung tissue expression.

### PRMT7 promoter analysis on publicly available ChIP-Seq data

PRMT7 promoter analysis on publicly available ChIP-Seq data was undertaken using the Cistrome Data Browser (http://cistrome.org)^19^, of five crucial myeloid Transcription Factors^20^.

### Quantification and statistical analysis

Results are presented as mean values ± SD, with sample size and number of repeats indicated in the figure legends. One-way ANOVA following Bonferroni post-test was used for all studies with more than two groups. For comparisons between two groups, unpaired two-tailed Student’s *t*-test was used. *P* values less than 0.05 were considered significant. Analyses were conducted using GraphPad Prism 6 and 8 software (GraphPad Software).

### Reporting summary

Further information on research design is available in the Nature Research Reporting Summary linked to this article.

## Supplementary information

Supplementary Information

Description of Additional Supplementary Files

Supplementary Data 1

Supplementary Data 2

Supplementary Data 3

Supplementary Data 4

Supplementary Data 5

Supplementary Data 6

Supplementary Data 7

Supplementary Movie 1

Supplementary Movie 2

Supplementary Movie 3

Supplementary Movie 4

Reporting Summary

## Data Availability

Source data are provided with this paper. Uncropped and unprocessed western blot scans can be found in the source data file. Proteomics data can be found in Supplementary Data Files 3 and 4. ATAC-Seq data was submitted to the NCBI Gene Expression Omnibus (GEO) database (GSE153666). Single-cell RNA-Seq data was submitted to the NCBI Gene Expression Omnibus (GEO) database (GSE185006) and micro-array data of our chronic cigarette smoke-exposed mice can be found at GSE125521. Source data are provided with this paper.

## Source data

Source Data

## Code availability

All code used for data visualization and analysis of the scRNA-seq data can be found at https://github.com/theislab/2021_PRMT7_regulates_Monocyte_Extravasation.

## References

[1] Hogg JC (2004). The nature of small-airway obstruction in chronic obstructive pulmonary disease. N. Engl. J. Med..

[2] John-Schuster G (2014). Cigarette smoke-induced iBALT mediates macrophage activation in a B cell-dependent manner in COPD. Am. J. Physiol. Lung Cell. Mol. Physiol..

[3] Rabe KF, Watz H (2017). Chronic obstructive pulmonary disease. Lancet.

[4] Conlon, T. M. et al. Inhibition of LTβR signalling activates WNT-induced regeneration in lung. *Nature*10.1038/s41586-020-2882-8 (2020). 10.1038/s41586-020-2882-8 PMC7718297 33149305

[5] Barnes PJ (2016). Inflammatory mechanisms in patients with chronic obstructive pulmonary disease. J. Allergy Clin. Immunol..

[6] Blanc RS, Richard S (2017). Arginine methylation: The coming of age. Mol. Cell.

[7] Bedford MT, Clarke SG (2009). Protein arginine methylation in mammals: who, what, and why. Mol. Cell.

[8] Zurita-Lopez CI, Sandberg T, Kelly R, Clarke SG (2012). Human protein arginine methyltransferase 7 (PRMT7) is a type III enzyme forming omega-NG-monomethylated arginine residues. J. Biol. Chem..

[9] Morrow JD (2017). Functional interactors of three genome-wide association study genes are differentially expressed in severe chronic obstructive pulmonary disease lung tissue. Sci. Rep..

[10] Beck T, Hastings RK, Gollapudi S, Free RC, Brookes AJ (2014). GWAS Central: a comprehensive resource for the comparison and interrogation of genome-wide association studies. Eur. J. Hum. Genet..

[11] Soler Artigas M (2011). Genome-wide association and large-scale follow up identifies 16 new loci influencing lung function. Nat. Genet..

[12] Consortium GT (2013). The genotype-tissue expression (GTEx) project. Nat. Genet..

[13] Sun Q (2012). Upregulated protein arginine methyltransferase 1 by IL-4 increases eotaxin-1 expression in airway epithelial cells and participates in antigen-induced pulmonary inflammation in rats. J. Immunol..

[14] Sarker RS (2015). Coactivator-associated arginine methyltransferase-1 function in alveolar epithelial senescence and elastase-induced emphysema susceptibility. Am. J. Respir. Cell Mol. Biol..

[15] Wang Q (2018). Identification of a novel protein arginine methyltransferase 5 inhibitor in non-small cell lung cancer by structure-based virtual screening. Front. Pharmacol..

[16] He X (2020). PRMT6 mediates inflammation via activation of the NF-kappaB/p65 pathway on a cigarette smoke extract-induced murine emphysema model. Tob. Induc. Dis..

[17] Uhlen M (2015). Proteomics. tissue-based map of the human proteome. Science.

[18] Lange, M. et al. CellRank for directed single-cell fate mapping. *Nat. Methods*10.1038/s41592-021-01346-6 (2022). 10.1038/s41592-021-01346-6 PMC8828480 35027767

[19] Mei S (2017). Cistrome data browser: a data portal for ChIP-Seq and chromatin accessibility data in human and mouse. Nucleic Acids Res..

[20] Sajti E (2020). Transcriptomic and epigenetic mechanisms underlying myeloid diversity in the lung. Nat. Immunol..

[21] Fornes O (2020). JASPAR 2020: update of the open-access database of transcription factor binding profiles. Nucleic Acids Res..

[22] Lu YC, Yeh WC, Ohashi PS (2008). LPS/TLR4 signal transduction pathway. Cytokine.

[23] Pierce JW (1997). Novel inhibitors of cytokine-induced IkappaBalpha phosphorylation and endothelial cell adhesion molecule expression show anti-inflammatory effects in vivo. J. Biol. Chem..

[24] Shin HM (2004). Inhibitory action of novel aromatic diamine compound on lipopolysaccharide-induced nuclear translocation of NF-kappaB without affecting IkappaB degradation. FEBS Lett..

[25] Yipp, B. G. et al. The lung is a host defense niche for immediate neutrophil-mediated vascular protection. *Sci. Immunol.*10.1126/sciimmunol.aam8929 (2017). 10.1126/sciimmunol.aam8929 PMC5472445 28626833

[26] Campa CC (2016). Rac signal adaptation controls neutrophil mobilization from the bone marrow. Sci. Signal..

[27] Szewczyk MM (2020). Pharmacological inhibition of PRMT7 links arginine monomethylation to the cellular stress response. Nat. Commun..

[28] Eram MS (2016). A potent, selective, and cell-active inhibitor of human type I protein arginine methyltransferases. ACS Chem. Biol..

[29] Chan-Penebre E (2015). A selective inhibitor of PRMT5 with in vivo and in vitro potency in MCL models. Nat. Chem. Biol..

[30] Stork PJ, Dillon TJ (2005). Multiple roles of Rap1 in hematopoietic cells: complementary versus antagonistic functions. Blood.

[31] Minato N, Kometani K, Hattori M (2007). Regulation of immune responses and hematopoiesis by the Rap1 signal. Adv. Immunol..

[32] Li Y (2007). Rap1a null mice have altered myeloid cell functions suggesting distinct roles for the closely related Rap1a and 1b proteins. J. Immunol..

[33] Lilja J (2017). SHANK proteins limit integrin activation by directly interacting with Rap1 and R-Ras. Nat. Cell Biol..

[34] Ying Z (2015). Histone arginine methylation by PRMT7 controls germinal center formation via regulating Bcl6 transcription. J. Immunol..

[35] Smirnova, N. F. et al. Inhibition of B cell-dependent lymphoid follicle formation prevents lymphocytic bronchiolitis after lung transplantation. *JCI insight*10.1172/jci.insight.123971 (2019). 10.1172/jci.insight.123971 PMC6413786 30728330

[36] Nayak DK (2016). Long-term persistence of donor alveolar macrophages in human lung transplant recipients that influences donor-specific immune responses. Am. J. Transplant..

[37] Houghton AM (2006). Elastin fragments drive disease progression in a murine model of emphysema. J. Clin. Investig..

[38] Ueno M (2015). Alendronate inhalation ameliorates elastase-induced pulmonary emphysema in mice by induction of apoptosis of alveolar macrophages. Nat. Commun..

[39] Groves AM, Johnston CJ, Williams JP, Finkelstein JN (2018). Role of infiltrating monocytes in the development of radiation-induced pulmonary fibrosis. Radiat. Res..

[40] Sager, H. B. et al. RNAi targeting multiple cell adhesion molecules reduces immune cell recruitment and vascular inflammation after myocardial infarction. *Sci. Transl. Med.* **8**, 342ra380 (2016). 10.1126/scitranslmed.aaf1435 PMC5125383 27280687

[41] Cloonan SM (2016). Mitochondrial iron chelation ameliorates cigarette smoke-induced bronchitis and emphysema in mice. Nat. Med..

[42] Jia, J. et al. Cholesterol metabolism promotes B-cell positioning during immune pathogenesis of chronic obstructive pulmonary disease. *EMBO Mol. Med.* **10**, e8349 (2018). 10.15252/emmm.201708349 PMC5938615 29674392

[43] Balint BL (2005). Arginine methylation provides epigenetic transcription memory for retinoid-induced differentiation in myeloid cells. Mol. Cell. Biol..

[44] Liu F (2015). Arginine methyltransferase PRMT5 is essential for sustaining normal adult hematopoiesis. J. Clin. Investig..

[45] Tarighat SS (2016). The dual epigenetic role of PRMT5 in acute myeloid leukemia: gene activation and repression via histone arginine methylation. Leukemia.

[46] He X (2019). PRMT1-mediated FLT3 arginine methylation promotes maintenance of FLT3-ITD(+) acute myeloid leukemia. Blood.

[47] Zepp JA (2017). Distinct mesenchymal lineages and niches promote epithelial self-renewal and myofibrogenesis in the lung. Cell.

[48] McCubbrey AL (2018). Deletion of c-FLIP from CD11b(hi) macrophages prevents development of bleomycin-induced lung fibrosis. Am. J. Respir. Cell Mol. Biol..

[49] Eming SA, Wynn TA, Martin P (2017). Inflammation and metabolism in tissue repair and regeneration. Science.

[50] Petrache I (2005). Ceramide upregulation causes pulmonary cell apoptosis and emphysema-like disease in mice. Nat. Med..

[51] Calabrese F (2005). Marked alveolar apoptosis/proliferation imbalance in end-stage emphysema. Respir. Res..

[52] Galluzzi L (2018). Molecular mechanisms of cell death: recommendations of the Nomenclature Committee on Cell Death 2018. Cell Death Differ..

[53] Conrad M, Pratt DA (2019). The chemical basis of ferroptosis. Nat. Chem. Biol..

[54] Doll S (2017). ACSL4 dictates ferroptosis sensitivity by shaping cellular lipid composition. Nat. Chem. Biol..

[55] Friedmann Angeli JP (2014). Inactivation of the ferroptosis regulator Gpx4 triggers acute renal failure in mice. Nat. Cell Biol..

[56] Guimaraes FR (2018). The inhibition of 5-Lipoxygenase (5-LO) products leukotriene B4 (LTB4) and cysteinyl leukotrienes (cysLTs) modulates the inflammatory response and improves cutaneous wound healing. Clin. Immunol..

[57] Proneth B, Conrad M (2019). Ferroptosis and necroinflammation, a yet poorly explored link. Cell Death Differ..

[58] Migliori V (2012). Symmetric dimethylation of H3R2 is a newly identified histone mark that supports euchromatin maintenance. Nat. Struct. Mol. Biol..

[59] Hautamaki RD, Kobayashi DK, Senior RM, Shapiro SD (1997). Requirement for macrophage elastase for cigarette smoke-induced emphysema in mice. Science.

[60] Seggev JS, Thornton WH, Edes TE (1991). Serum leukotriene B4 levels in patients with obstructive pulmonary disease. Chest.

[61] Metzemaekers M, Gouwy M, Proost P (2020). Neutrophil chemoattractant receptors in health and disease: double-edged swords. Cell Mol. Immunol..

[62] Cottrell JA, O’Connor JP (2009). Pharmacological inhibition of 5-lipoxygenase accelerates and enhances fracture-healing. J. Bone Jt. Surg. Am..

[63] Martinez-Clemente M (2010). 5-lipoxygenase deficiency reduces hepatic inflammation and tumor necrosis factor alpha-induced hepatocyte damage in hyperlipidemia-prone ApoE-null mice. Hepatology.

[64] Berger W, De Chandt MT, Cairns CB (2007). Zileuton: clinical implications of 5-Lipoxygenase inhibition in severe airway disease. Int J. Clin. Pr..

[65] Shapiro SD (2003). Neutrophil elastase contributes to cigarette smoke-induced emphysema in mice. Am. J. Pathol..

[66] Baßler, K. et al. Alterations of multiple alveolar macrophage states in chronic obstructive pulmonary disease. Preprint at *bioRxiv* (2020).

[67] Gu Z, Eils R, Schlesner M (2016). Complex heatmaps reveal patterns and correlations in multidimensional genomic data. Bioinformatics.

[68] John G (2014). The composition of cigarette smoke determines inflammatory cell recruitment to the lung in COPD mouse models. Clin. Sci..

[69] Yildirim AO (2010). Palifermin induces alveolar maintenance programs in emphysematous mice. Am. J. Respir. Crit. Care Med..

[70] Fallica J, Das S, Horton M, Mitzner W (2011). Application of carbon monoxide diffusing capacity in the mouse lung. J. Appl. Physiol..

[71] Vanoirbeek JA (2010). Noninvasive and invasive pulmonary function in mouse models of obstructive and restrictive respiratory diseases. Am. J. Respir. Cell Mol. Biol..

[72] Macosko EZ (2015). Highly parallel genome-wide expression profiling of individual cells using nanoliter droplets. Cell.

[73] Wolf, F. A., Angerer, P. & Theis, F. J. SCANPY: large-scale single-cell gene expression data analysis. *Genome Biol.* **19**, 10.1186/s13059-017-1382-0 (2018). 10.1186/s13059-017-1382-0 PMC5802054 29409532

[74] Luecken MD, Theis FJ (2019). Current best practices in single-cell RNA-seq analysis: a tutorial. Mol. Syst. Biol..

[75] La Manno G (2018). RNA velocity of single cells. Nature.

[76] Bergen V, Lange M, Peidli S, Wolf FA, Theis FJ (2020). Generalizing RNA velocity to transient cell states through dynamical modeling. Nat. Biotechnol..

[77] Mutze K, Vierkotten S, Milosevic J, Eickelberg O, Konigshoff M (2015). Enolase 1 (ENO1) and protein disulfide-isomerase associated 3 (PDIA3) regulate Wnt/beta-catenin-driven trans-differentiation of murine alveolar epithelial cells. Dis. Model Mech..

[78] Konigshoff M (2009). WNT1-inducible signaling protein-1 mediates pulmonary fibrosis in mice and is upregulated in humans with idiopathic pulmonary fibrosis. J. Clin. Investig..

[79] Wang X, Spandidos A, Wang H, Seed B (2012). PrimerBank: a PCR primer database for quantitative gene expression analysis, 2012 update. Nucleic Acids Res..

[80] Guillot, A., Kohlhepp, M. S., Bruneau, A., Heymann, F. & Tacke, F. Deciphering the immune microenvironment on a single archival formalin-fixed paraffin-embedded tissue section by an immediately implementable multiplex fluorescence immunostaining protocol. *Cancers* **12**, 2449 (2020). 10.3390/cancers12092449 PMC7565194 32872334

[81] Schindelin J (2012). Fiji: an open-source platform for biological-image analysis. Nat. Methods.

[82] Berg S (2019). ilastik: interactive machine learning for (bio)image analysis. Nat. Methods.

[83] Wahlby C (2012). An image analysis toolbox for high-throughput C. elegans assays. Nat. Methods.

[84] Cong L (2013). Multiplex genome engineering using CRISPR/Cas systems. Science.

[85] Kasparek P (2014). Efficient gene targeting of the Rosa26 locus in mouse zygotes using TALE nucleases. FEBS Lett..

[86] Kanashova T (2015). Differential proteomic analysis of mouse macrophages exposed to adsorbate-loaded heavy fuel oil derived combustion particles using an automated sample-preparation workflow. Anal. Bioanal. Chem..

[87] Wrzodek C, Eichner J, Buchel F, Zell A (2013). InCroMAP: integrated analysis of cross-platform microarray and pathway data. Bioinformatics.

[88] Seiler A (2008). Glutathione peroxidase 4 senses and translates oxidative stress into 12/15-lipoxygenase dependent- and AIF-mediated cell death. Cell Metab..

[89] Zhang Y (2008). Model-based analysis of ChIP-Seq (MACS). Genome Biol..

[90] Sandelin A, Alkema W, Engstrom P, Wasserman WW, Lenhard B (2004). JASPAR: an open-access database for eukaryotic transcription factor binding profiles. Nucleic Acids Res..

[91] Mootha VK (2003). PGC-1alpha-responsive genes involved in oxidative phosphorylation are coordinately downregulated in human diabetes. Nat. Genet..

[92] Subramanian A (2005). Gene set enrichment analysis: a knowledge-based approach for interpreting genome-wide expression profiles. Proc. Natl Acad. Sci. USA.

[93] Campbell JD (2012). A gene expression signature of emphysema-related lung destruction and its reversal by the tripeptide GHK. Genome Med..

