# Supplementary material for: The arginine methyltransferase PRMT7 promotes extravasation of monocytes resulting in tissue injury in COPD
Source: Nat Commun. 2022 Mar 14;13:1303. doi: 10.1038/s41467-022-28809-4 (PMC8921220; doi:10.1038/s41467-022-28809-4)
Supplement: Supplementary file 1 — Supplementary Information [file 41467_2022_28809_MOESM1_ESM.pdf]

Supplementary Information

The Arginine Methyltransferase PRMT7 Promotes Extravasation of Monocytes resulting in tissue injury in COPD

Gizem Günes Günsel[1,†], Thomas M. Conlon[1,†], Aicha Jeridi[1,†], Rinho Kim[2], Zeynep Ertüz[1], Niklas J. Lang[1], Meshal Ansari[1,3], Mariia Novikova[4,5,6], Dongsheng Jiang[1], Maximilian Strunz[1], Mariia Gaianova[1], Christine Hollauer[1], Christina Gabriel[1], Ilias Angelidis[1], Sebastian Doll[3], Jeanine C. Pestoni[1], Stephanie L. Edelmann[7], Marlene Sophia Kohlhepp[8], Adrien Guillot[8], Kevin Bassler[9,10], Hannelore P. Van Eeckhoutte[11], Özgecan Kayalar[12], Nur Konyalilar[12], Tamara Kanashova[13], Sophie Rodius[14], Carolina Ballester-López[1], Carlos M. Genes Robles[2], Natalia Smirnova[1,15], Markus Rehberg[1], Charu Agarwal[1], Ioanna Krikki[1], Benoit Piavaux[16], Stijn E. Verleden[17,18], Bart Vanaudenaerde[17], Melanie Königshoff[1,19], Gunnar Dittmar[14,20], Ken R. Bracke[11], Joachim L. Schultze[9,21], Henrik Watz[22], Oliver Eickelberg[19], Tobias Stoeger[1], Gerald Burgstaller[1], Frank Tacke[8], Vigo Heissmeyer[7,23], Yuval Rinkevich[1], Hasan Bayram[12], Herbert B. Schiller[1], Marcus Conrad[4,5], Robert Schneider[2], Ali Önder Yildirim[1, 12]*

Supplementary Information Contents:

Supplementary Figures 1-24

Supplementary Tables 1-5

Supplementary Figures 25-52 (Uncropped blots from Supplementary Figures 1-24)

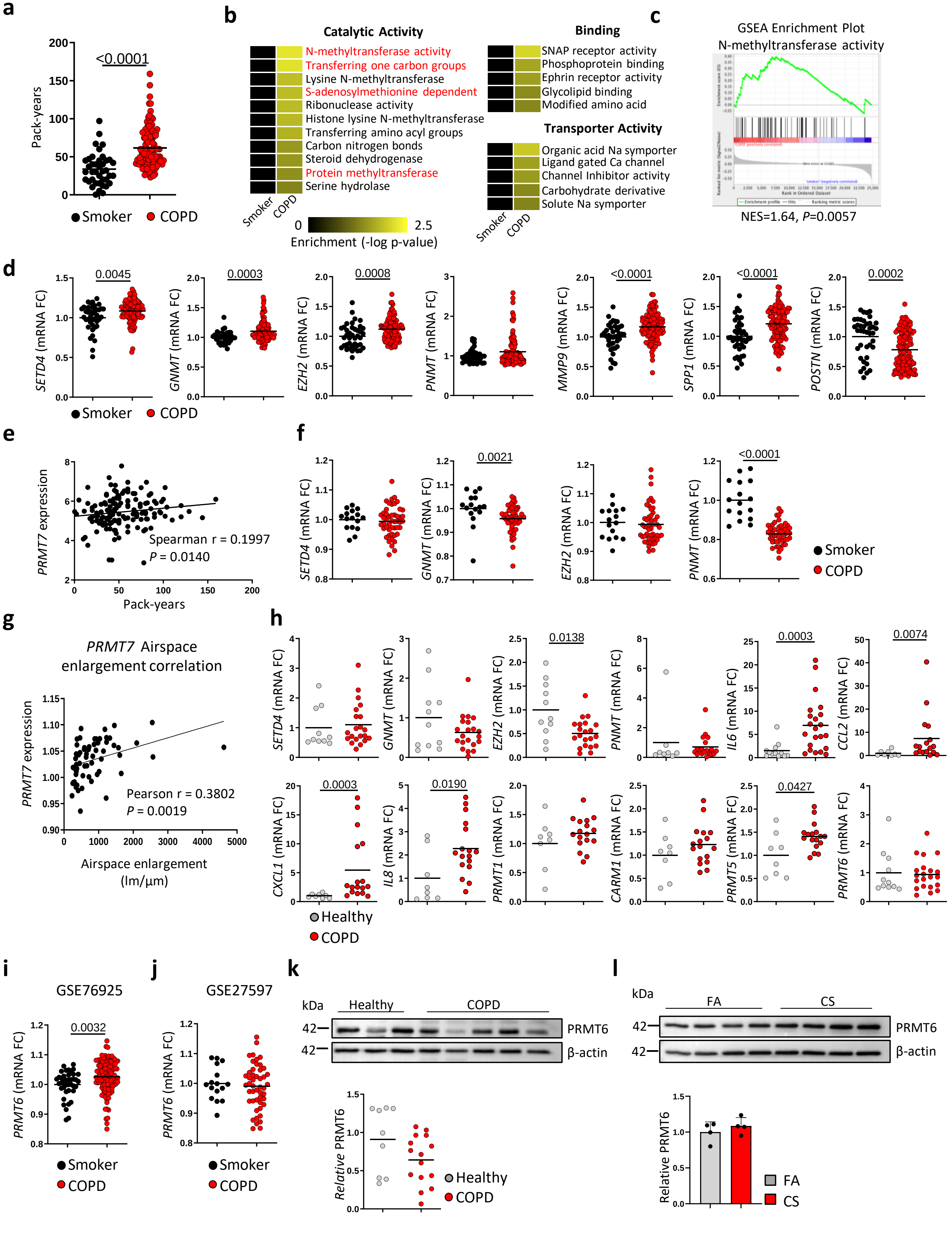

Supplementary Figure 1

Supplementary Figure 1. PRMT7 expression correlates with COPD severity. a, Smoking history of patients analysed in the publicly available array data of lung tissue (GSE76925) from healthy smokers (n=40) vs COPD patients (n=111). **b**, Most significantly enriched gene lists in the lungs of COPD patients, separated according to function, following GSEA of the GO Molecular Function set (642 gene sets) on GSE76925. Nominal *P* value generated by the GSEA software of the enrichment score relative to the null distribution. **c**, Enrichment plot for GO N-methyltransferase activity (GO:0008170) following GSEA described in b. NES, normalised enrichment score. Nominal *P* value generated by the GSEA software of the enrichment score relative to the null distribution. **d**, Expression of genes indicated in the lungs of COPD patients (n=111) relative to healthy smokers (n=40) taken from GSE76925. **e**, Correlation of smoking history (pack-years) with *PRMT7* gene expression from GSE76925. **f**, Expression of genes indicated in the lungs of COPD patients relative to healthy smokers taken from GSE27597 (n=16 smokers and n=48 COPD patients). **g**, Correlation of emphysema with *PRMT7* gene expression from GSE27597. **h**, mRNA expression levels of genes indicated, determined by qPCR, in lung core biopsies from COPD patients (n=17) relative to healthy controls (n=11), individual patients shown. **i-j**, Expression of *PRMT6*, in the lungs of COPD patients relative to healthy smokers, taken from GSE76925 (n=40 smokers and n=111 COPD patients) (i) and GSE27597 (n=16 smokers and n=48 COPD patients) (j). **k**, Western blot analysis of PRMT6 expression in lung core biopsies from healthy (n=9) and COPD patients (n=15), normalised to β-actin and shown relative to controls. **l**, Western blot analysis of PRMT6 expression in the lungs of mice exposed to filtered air (FA, n=4) or cigarette smoke (CS, n=4) for 4 months, normalised to β-actin and shown relative to FA controls. Data shown mean ± SD. *P* values shown in charts determined by two tailed-Mann Whitney test (a,d,f,h-k), Spearman Correlation two-tailed (e), and Pearson Correlation two-tailed (g), unpaired two-tailed Student's t test (l). Uncropped blots can be found in Supplementary Figures 25-26. Source data are provided as a Source Data file.

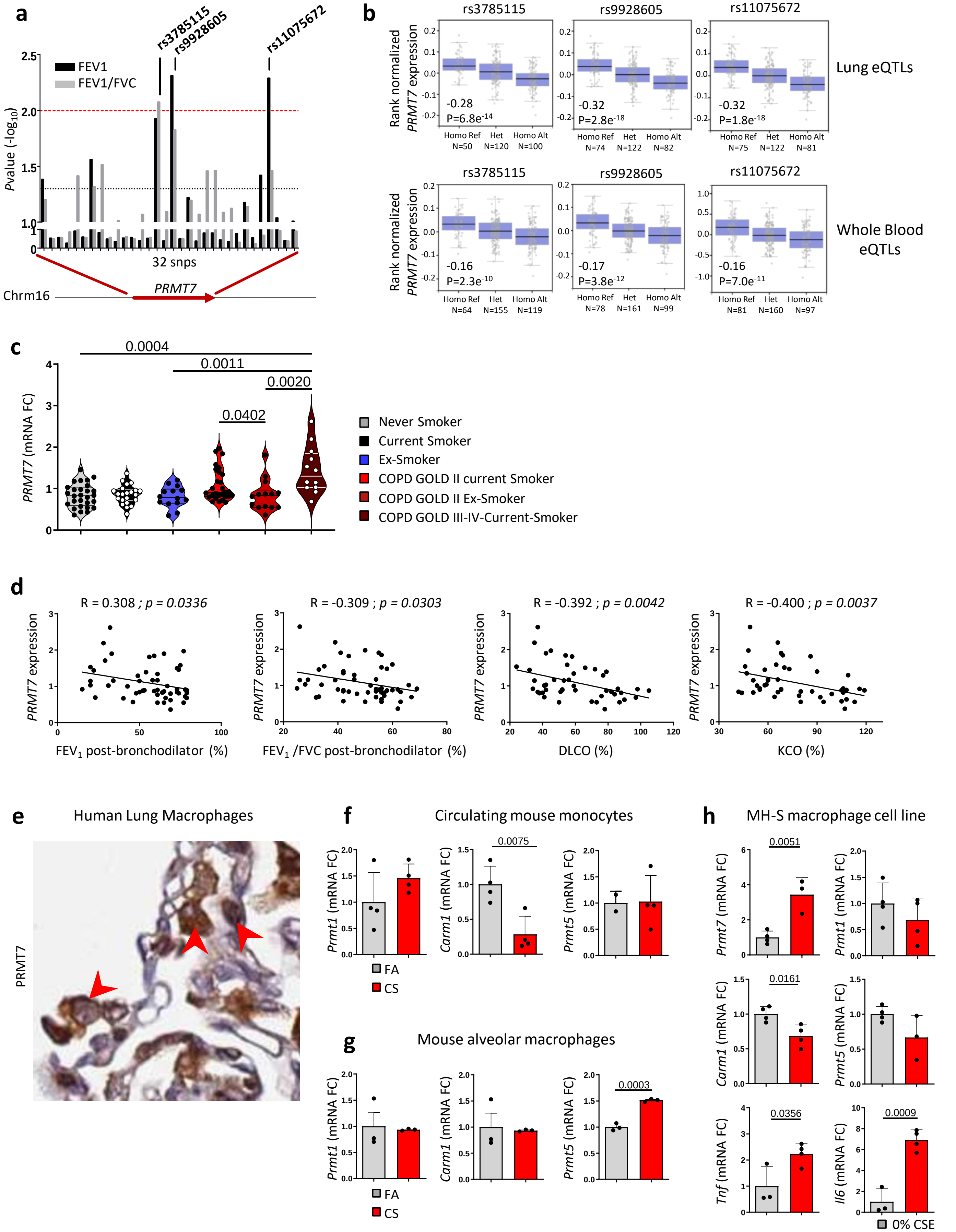

Supplementary Figure 2

Supplementary Figure 2. PRMT7 expression pattern. **a**, Genome-wide association study (GWAS) association results for FEV1 and FEV1/FVC of SNPs within the *PRMT7* locus. SNPs with an association of –log*P* > 2 indicated. Data was obtained from the GWAS Central database (www.gwascentral.org) from a genome-wide association study composed of over 48,000 individuals[11]. **b**, *PRMT7* expression levels in the lung and whole blood of individuals with the 3 SNPs highlighted as either heterozygotes or homozygotes compared to reference sequence, presented as eQTL (expression quantitative trait loci) box plots. Data taken from the GTEx Portal database (www.gtexportal.org/home). Insert shows normalised effect size and *P* value (Nominal *P* values were generated for each variant-gene pair by testing the alternative hypothesis that the slope of a linear regression model between genotype and expression deviates from 0). Individuals represented as dots with n numbers on the x-axis, with median, 1st and 3rd quartiles and 1.5 interquartile range of 1st and 3rd quartiles highlighted. **c**, *PRMT7* mRNA expression levels in lung tissue from never smokers (n=28), current smokers (n=22), ex-smokers (n=14), current smoker patients with COPD GOLD stage II (n=28), ex-smoker patients with COPD GOLD stage II (n=28) and smoker patients with COPD GOLD stage III-IV (n=12), determined by qPCR, individual patients shown. **d**, Correlation of *PRMT7* mRNA expression and disease severity within the COPD patients in c, as determined by FEV1 % post-bronchodilator, FEV1/FVC % post-bronchodilator, DLCO (%) and KCO (%). **e**, PRMT7 expression in macrophages (red arrows) taken from the human protein atlas v19.0 image https://www.proteinatlas.org/ENSG00000132600-PRMT7/tissue/lung, image credit: Human Protein Atlas. **f**-**g**, mRNA expression levels of *Prmt1*, *Carm1* and *Prmt5* determined by qPCR in circulating monocytes (n=4 per group) (**f**) and in alveolar macrophages (n=3 per group) (**g**) from B6 mice after 3 days of cigarette smoke exposure relative to filtered air (FA) controls. **h**, mRNA expression levels of *Prmt7* (n=4 0%, n=3 5%), *Prmt1* (n=4 0%, n=4 5%), *Carm1* (n=4 0%, n=4 5%), *Prmt5* (n=4 0%, n=3 5%), *Tnf* (n=3 0%, n=4 5%) and *Il6* (n=3 0%, n=4 5%) determined by qPCR in the MH-S macrophage cell line exposed to 5% cigarette smoke extract for 24 h relative to 0% CSE controls. Data shown mean ± SD or violin plot with lines representing median and quartiles (c). *P* values shown in charts determined by two tailed-Mann Whitney test (c), F test of linear regression (d) and unpaired two-tailed Student's t test (f-h). Source data are provided as a Source Data file.

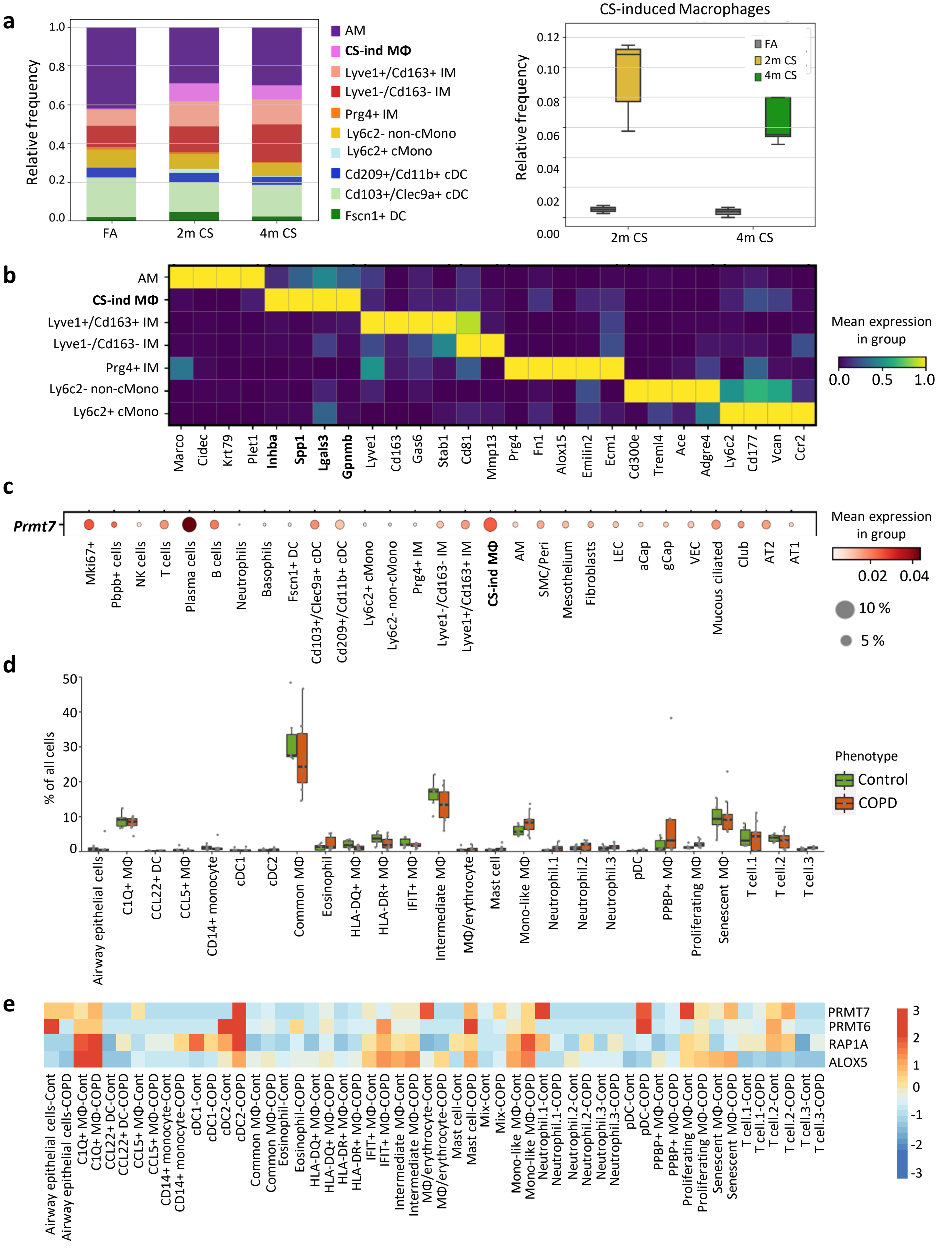

Supplementary Figure 3

Supplementary Figure 3. scRNA-seq analysis of lungs from mice exposed to cigarette smoke and the bronchoalveolar lavage of COPD patients. a-c, scRNA-Seq (Drop-Seq) analysis on the lungs of mice following exposure to filtered air (FA, n=3) and cigarette smoke (CS) for 2 (n=5) and 4 months (n=5). AM, alveolar macrophages; CS-ind MØ, CS-induced macrophages; IM, interstitial macrophages; cMono, classical monocytes. **a**, Relative frequency in the lungs of the myeloid cells indicated. In the right panel the boxes represent the interquartile range, the horizontal line in the box is the median, and the whiskers represent 1.5 times the interquartile range. **b**, Heat map depicting the expression of key genes used in identifying the macrophage populations. **c**, Dot blot depicting the expression level (log-transformed, normalised UMI counts) and percentage of cells positive for *Prmt7* across all lung cell populations identified. **d-e**, scRNA-Seq data of bronchoalveolar lavage from COPD patients (n=9) and healthy controls (n=6). **d**, Box plot visualization (boxes represent the interquartile range, the horizontal line in the box is the median, and the whiskers represent 1.5 times the interquartile range) of the relative population sizes of human alveolar immune cells between COPD (n=9) and control (n=6). **e**, Heatmap of gene expressions of *PRMT6/7*, *RAP1A* and *ALOX5* in alveolar immune cells. Mean gene expression per cell type and condition is presented as a z-transformed value (across all cell types). Source data are provided as a Source Data file.

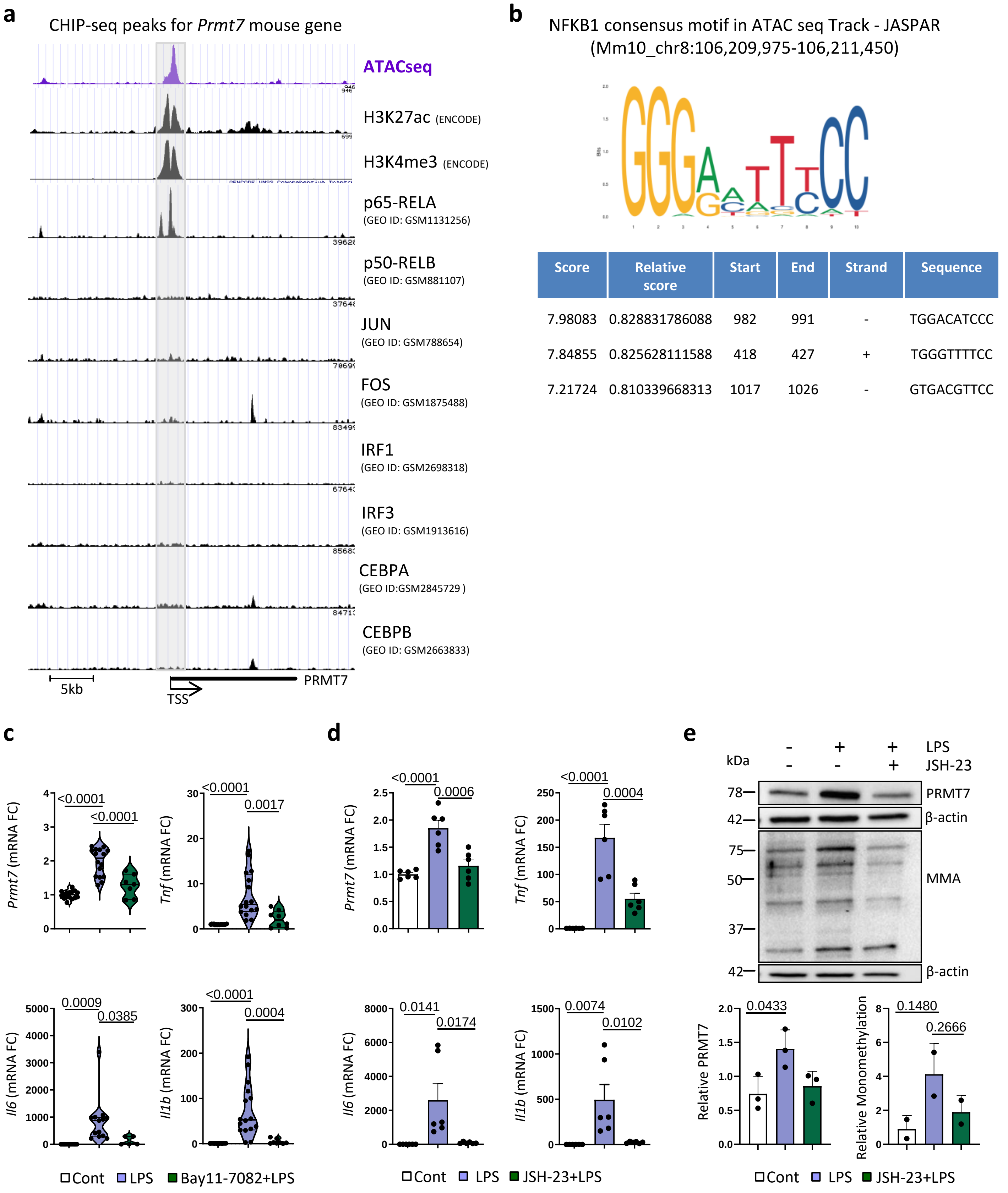

Score	Relative score	Start	End	Strand	Sequence
7.98083	0.828831786088	982	991	-	TGGACATCCC
7.84855	0.825628111588	418	427	+	TGGGTTTTCC
7.21724	0.810339668313	1017	1026	-	GTGACGTTCC

Supplementary Figure 4

Supplementary Figure 4. Regulation of PRMT7 expression in myeloid cells. a, Enrichment of several key transcriptional factors at the transcriptional start site (TSS) of mouse *Prmt7* gene by CHIP-Seq analysis with the help of Custom tracks on the UCSC genome browser. ATAC-Seq from our MH-S macrophage cell line for the same region highlighted. **b**, JASPAR analysis of the ATAC seq Track across the TSS of *Prmt7* revealed the presence of NFKB1 consensus motifs. **c**, mRNA expression levels of *Prmt7* (n=16 for Cont, LPS and n=7 for Bay11-7082 +LPS), *Tnf* (n=16 for Cont, LPS and n=8 for Bay11-7082 +LPS), *Il6* (n=14 for Cont, LPS and n=5 for Bay11-7082 +LPS), and *Il1b* (n=16 for Cont, LPS and n=8 for Bay11-7082 +LPS), determined by qPCR in primary mouse monocytes stimulated with 100 ng of LPS or LPS combined with 1 µM of BAY11-7082 drug for 24 h , relative to controls. **d**, mRNA expression levels of *Prmt7, Tnf, Il6* and *Il1b* (n=6 per group) determined by qPCR in primary mouse monocytes stimulated with 100 ng of LPS or LPS combined with 20 µM of JSH-23 drug for 6 h , relative to controls. **e**, Western blot analysis of PRMT7 expression (n=3 per group) as well as total mono-methylation levels (n=2 per group) in primary monocytes stimulated with LPS and JSH-23 as indicated for 24 h (representative of two independent experiments). Data shown mean ± SD or violin plot with lines representing median and quartiles (c). *P* values shown in charts determined by one-way ANOVA Bonferroni's multiple comparisons test (c-e). Uncropped blots can be found in Supplementary Figure 27. Source data are provided as a Source Data file.

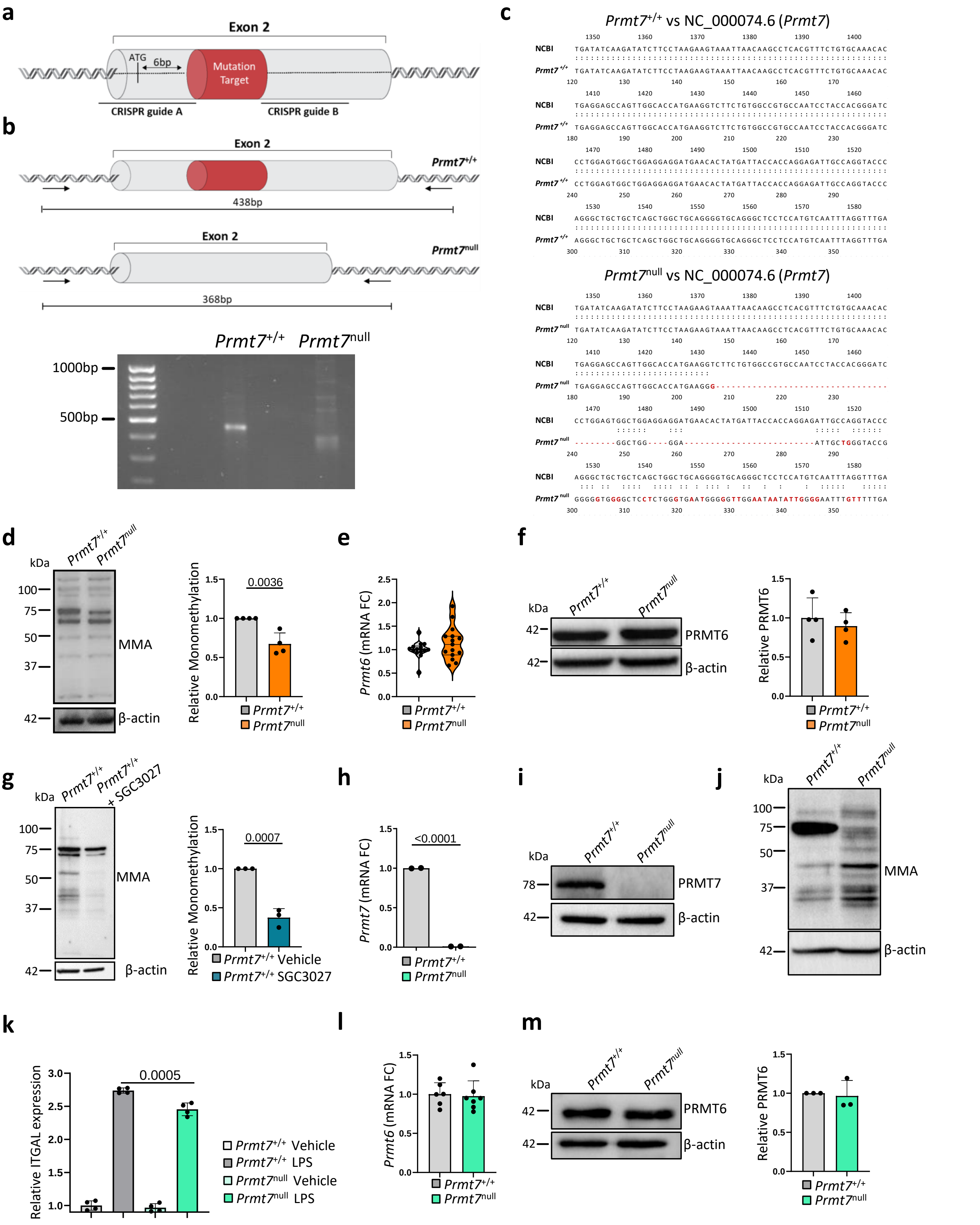

Supplementary Figure 5

Supplementary Figure 5. CRISPR/Cas9 targeting of *Prmt7* in the murine MH-S and RAW264.7 macrophage cell lines. a, Schematic representation of the location of the CRISPR guide sequences and mutation target site within exon 2 of the *Prmt7* gene, which contains the ATG start site used on the MH-S cells. **b**, PCR of genomic DNA with primers flanking the mutation target area generates a 438 bp band on DNA from *Prmt7*$^{+/+}$ MH-S cells which is 368 bp in size in *Prmt7*^{null} MH-S cells (repeated two times). **c**, Sequence analysis of genomic DNA from *Prmt7*$^{+/+}$ and *Prmt7*^{null} MH-S cells, compared to *Prmt7* sequence from NC_000074.6 (NCBI), confirms the deletion in exon 2 of *Prmt7* in *Prmt7*^{null} MH-S cells. **d**, Western blot analysis of total mono-methylated arginine residues in *Prmt7*$^{+/+}$ and *Prmt7*^{null} MH-S cells. Quantification relative to *Prmt7*$^{+/+}$ after normalization to β-actin (repeated four times). **e**, mRNA expression level of *Prmt6* determined by qPCR in *Prmt7*$^{+/+}$ and *Prmt7*^{null} MH-S cells relative to *Prmt7*$^{+/+}$ (n=14 replicates per cell line). **f**, Western blot analysis of PRMT6 expression in *Prmt7*$^{+/+}$ and *Prmt7*^{null} MH-S cells. Quantification relative to *Prmt7*$^{+/+}$ after normalization to β-actin (repeated four times). **g**, Western blot analysis of monomethylation levels in *Prmt7*$^{+/+}$ MH-S cells treated with 5 µM of SGC3027 drug to validate its function. Quantification relative to vehicle after normalization to β-actin (repeated three times). **h**, mRNA expression level of *Prmt7* determined by qPCR in *Prmt7*$^{+/+}$ and *Prmt7*^{null} RAW264.7 cells relative to *Prmt7*$^{+/+}$ (n=2 replicates per cell line). **i**, Western blot analysis of PRMT7 expression in *Prmt7*$^{+/+}$ and *Prmt7*^{null} RAW264.7 cells (repeated two times). **j**, Western blot analysis of total mono-methylated arginine residues in *Prmt7*$^{+/+}$ and *Prmt7*^{null} RAW264.7 cells. **k**, MFI of ITGAL surface expression as determined by flow cytometry in *Prmt7*$^{+/+}$ and *Prmt7*^{null} RAW264.7 cells (n=2 repeated twice). **l**, mRNA expression level of *Prmt6* determined by qPCR in *Prmt7*$^{+/+}$ (n=6) and *Prmt7*^{null} (n=7) RAW264.7 cells relative to *Prmt7*$^{+/+}$. **m**, Western blot analysis of PRMT6 expression in *Prmt7*$^{+/+}$ and *Prmt7*^{null} RAW264.7 cells. Quantification relative to *Prmt7*$^{+/+}$ after normalization to β-actin (repeated three times). Data shown mean ± SD or violin plot with lines representing median and quartiles (e). *P* values shown in charts determined by unpaired two-tailed Student's t test (d-g, k-m). Uncropped blots can be found in Supplementary Figures 28-33. Source data are provided as a Source Data file.

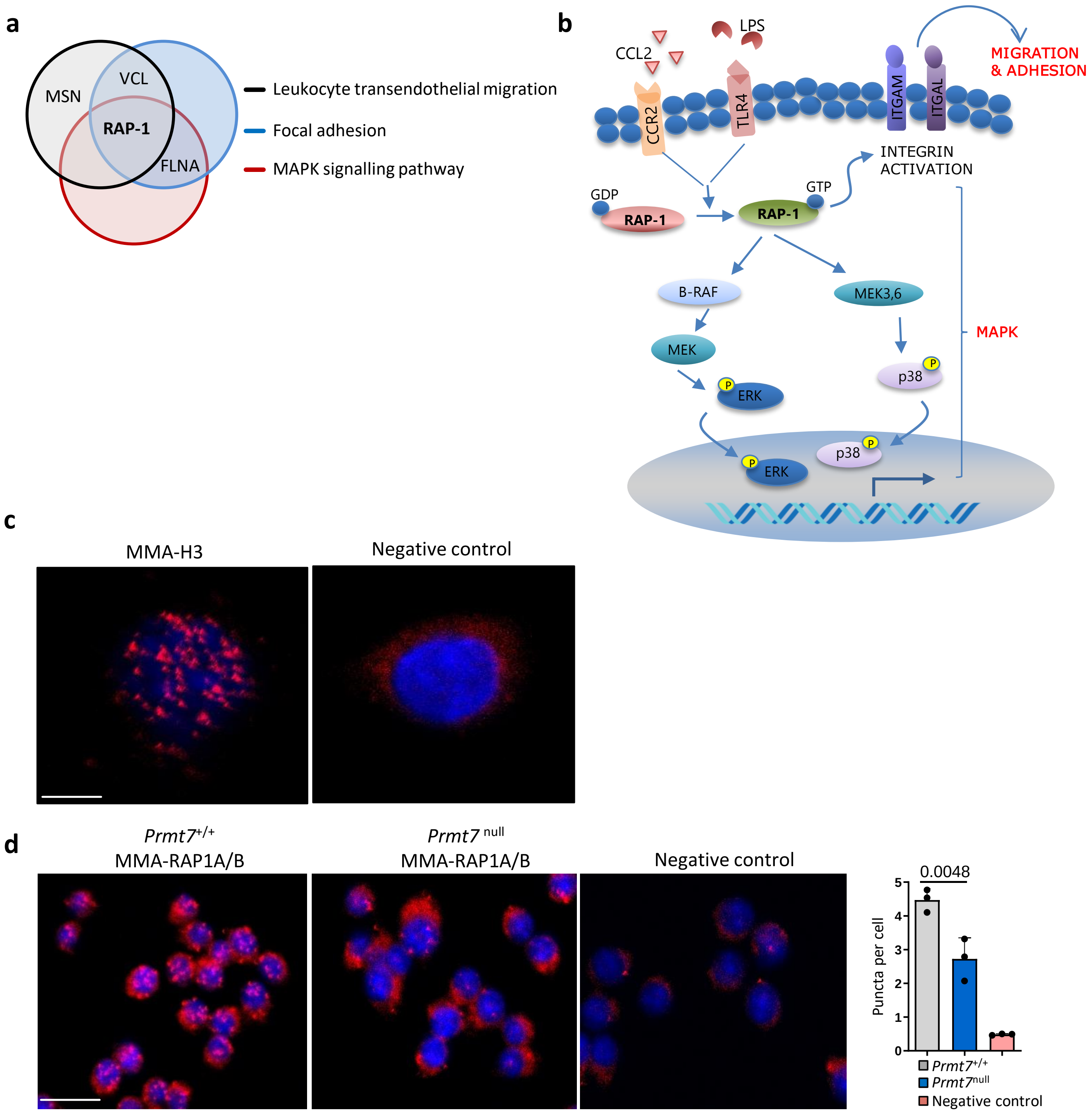

Supplementary Figure 6

Supplementary Figure 6. Arginine monomethylation of RAP1A/B. a, Venn diagram of proteins from the leukocyte trans-endothelial migration pathway, focal adhesion and MAPK signaling pathway dysregulated in *Prmt7*^{null} cells. **b**, Schematic representation of RAP1A/B downstream signalling pathways. **c-d**, Representative images of proximity ligation assays in RAW264.7 macrophages, counterstained with DAPI (blue). **c**, Representative image showing PLA signals between monomethylated arginine (MMA) and Histone H3 (red puncta) and secondary antibody only control (scale bar, 5 µm). **d**, Representative images showing PLA signals between MMA and RAP1A/B (red puncta) in *Prmt7*$^{+/+}$ and *Prmt7*^{null} RAW264.7 cells and secondary antibody only control from *Prmt7*$^{+/+}$ cells (scale bar, 20 µm). Quantification of Puncta per cell shown, mean ± SD, *P* value shown in chart determined by one-way ANOVA Tukey's multiple comparisons test, from 3 random fields of view 33-117 cells per view. Source data are provided as a Source Data file.

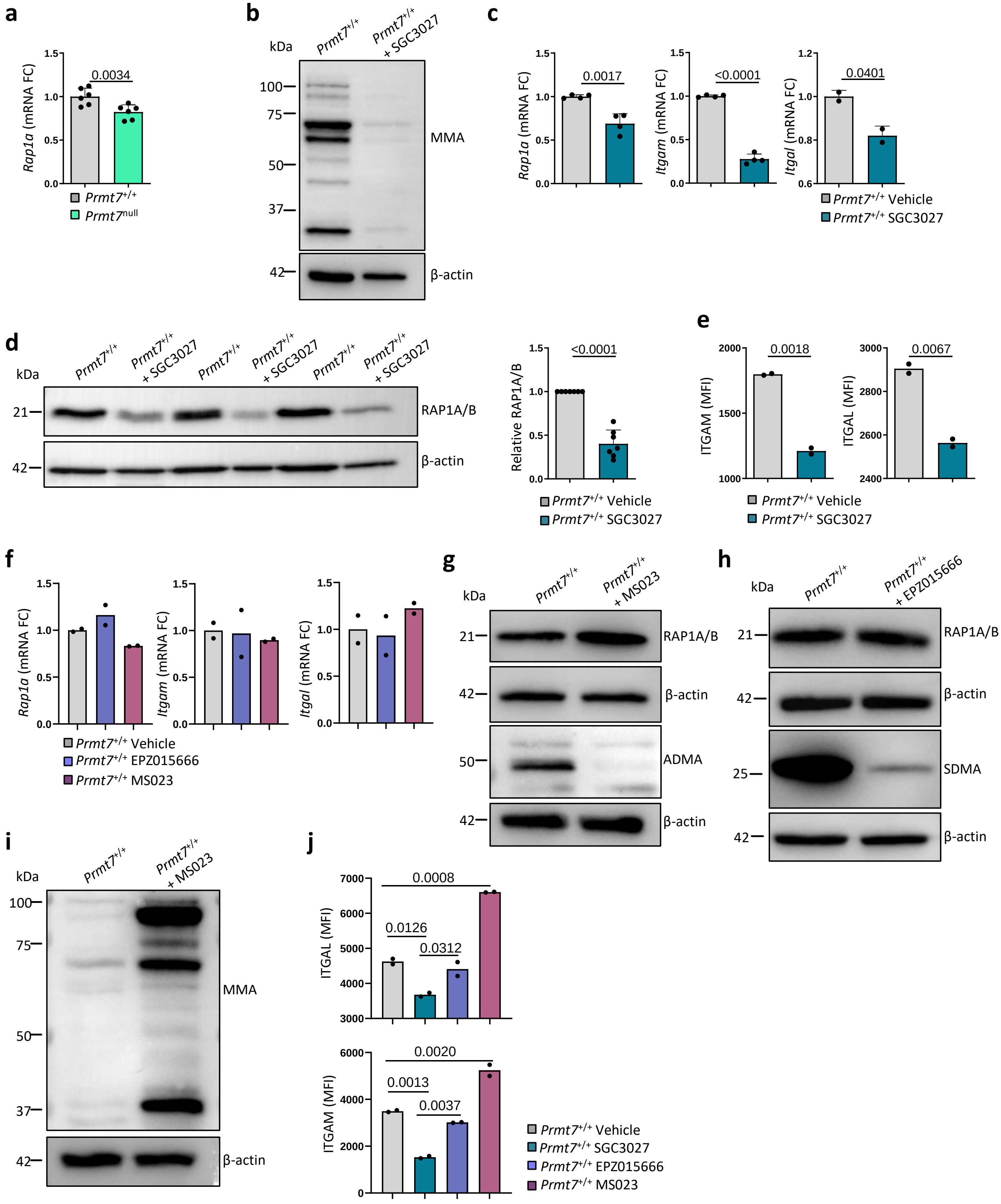

Supplementary Figure 7

Supplementary Figure 7. Arginine monomethylation is a crucial regulator for the expression of trans-endothelial migratory proteins. a, mRNA expression level of *Rap1a* determined by qPCR in *Prmt7*$^{+/+}$ and *Prmt7*^{null} RAW264.7 cells relative to *Prmt7*$^{+/+}$ (n=6 replicates per cell line). **b**, Western blot analysis of monomethylation (MMA) levels in *Prmt7*$^{+/+}$ MH-S cells treated with 7.5 µM of SGC3027 for 48 h. **c**, mRNA expression level of *Rap1a*, *Itgam* and *Itgal* determined by qPCR in *Prmt7*$^{+/+}$ MH-S cells treated with 7.5 µM of SGC3027 for 48 h relative to vehicle (n=2, repeated twice). **d**, Western blot analysis of RAP1A/B in *Prmt7*$^{+/+}$ MH-S cells treated with 7.5 µM of SGC3027 for 48 h. Quantification relative to vehicle after normalization to β-actin (repeated seven times). **e**, MFI of ITGAM and ITGAL surface expression as determined by flow cytometry in *Prmt7*$^{+/+}$ MH-S cells treated with 7.5 µM of SGC3027 for 48 h (n=2). **f**, mRNA expression level of *Rap1a*, *Itgam* and *Itgal* determined by qPCR in *Prmt7*$^{+/+}$ MH-S cells treated with 0.2 µM of EPZ015666 or 1 µM of MS023 for 48h relative to vehicle (n=2). **g**, Western blot analysis of RAP1A/B and asymmetric dimethylated arginine (ADMA) in *Prmt7*$^{+/+}$ MH-S cells treated with 1 µM of MS023 for 48 h (repeated twice). **h**, Western blot analysis of RAP1A/B and symmetric dimethylated arginine (SDMA) in *Prmt7*$^{+/+}$ MH-S cells treated with 0.2 µM of EPZ015666 for 48 h (repeated twice). **i**, Western blot analysis of MMA levels in *Prmt7*$^{+/+}$ MH-S cells treated with 1 µM of MS023 for 48 h (repeated two times). **j**, MFI of ITGAM and ITGAL surface expression as determined by flow cytometry in *Prmt7*$^{+/+}$ MH-S cells treated with 7.5 µM of SGC3027, 0.2 µM of EPZ015666 or 1 µM of MS023 for 48 h (n=2). Data shown mean ± SD, *P* values shown in charts determined by unpaired two-tailed Student's t test (a, c-f) and one-way ANOVA Tukey's multiple comparisons test (j). Uncropped blots can be found in Supplementary Figures 34-38. Source data are provided as a Source Data file.

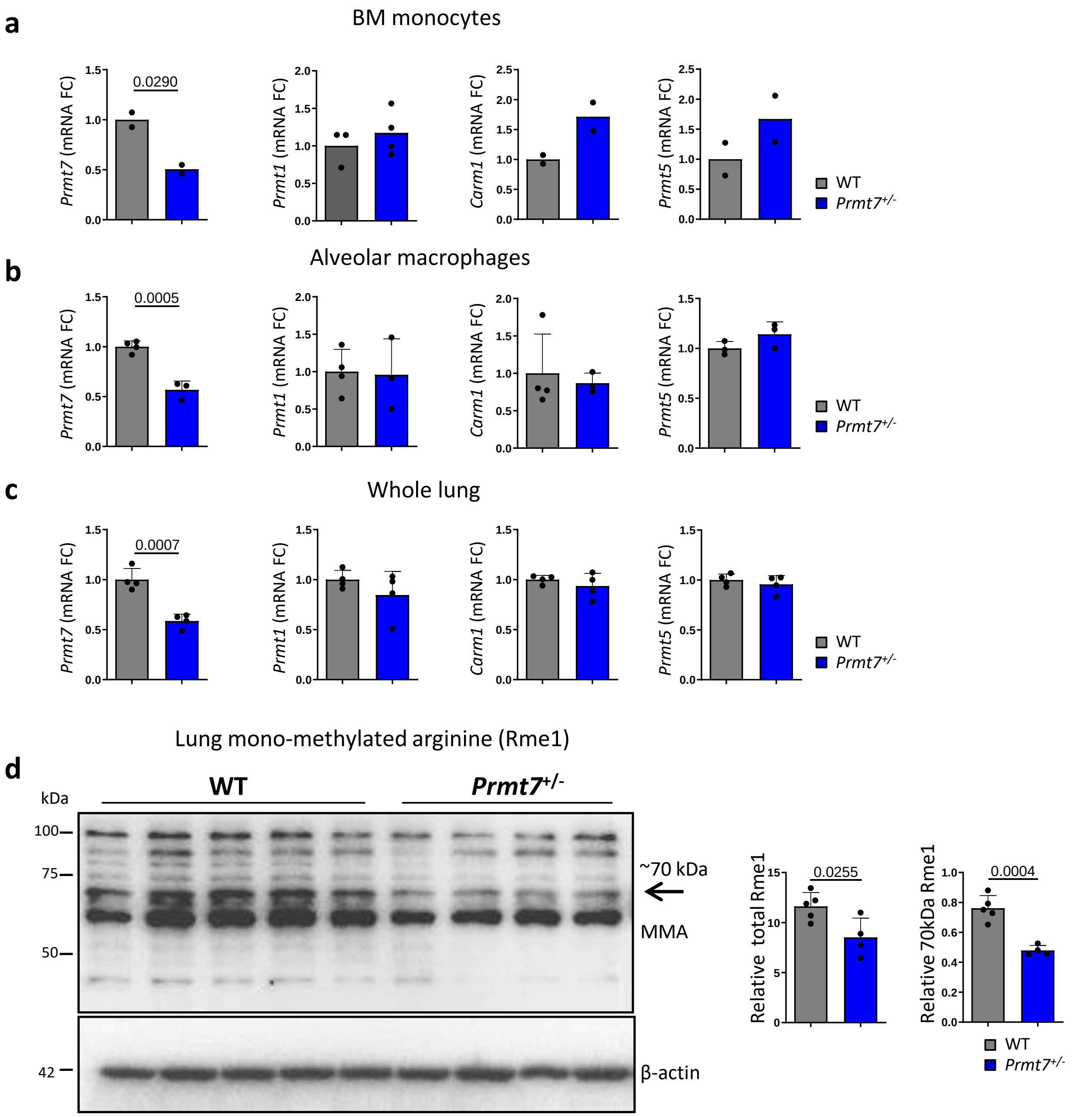

Supplementary Figure 8.

Supplementary Figure 8. Reduced PRMT7 expression and function in the lungs of *Prmt7*$^{+/-}$ mice. a, mRNA expression levels of *Prmt7*, *Prmt1*, *Carm1* and *Prmt5* by qPCR in bone marrow monocytes isolated from wild-type (WT, n=2) and *Prmt7*$^{+/-}$ (n=2) mice relative to WT. **b**, mRNA expression levels of *Prmt7*, *Prmt1*, *Carm1* and *Prmt5* by qPCR in alveolar macrophages isolated from the lungs of WT (n=4) and *Prmt7*$^{+/-}$ (n=3) mice relative to WT. **c**, mRNA expression levels of *Prmt7*, *Prmt1*, *Carm1* and *Prmt5* by qPCR in whole lung of WT (n=4) and *Prmt7*$^{+/-}$ (n=4) mice relative to WT. **d**, Western blot analysis of total mono-methylated arginine (Rme1) residues in whole lung of WT (n=5) and *Prmt7*$^{+/-}$ (n=4) mice. Quantification of total mono-methylation across all protein bands and bands at 70kDa, normalised to β-actin. Data shown mean ± SD, *P* values shown in charts determined by unpaired two-tailed Student's t test. Uncropped blots can be found in Supplementary Figure 39. Source data are provided as a Source Data file.

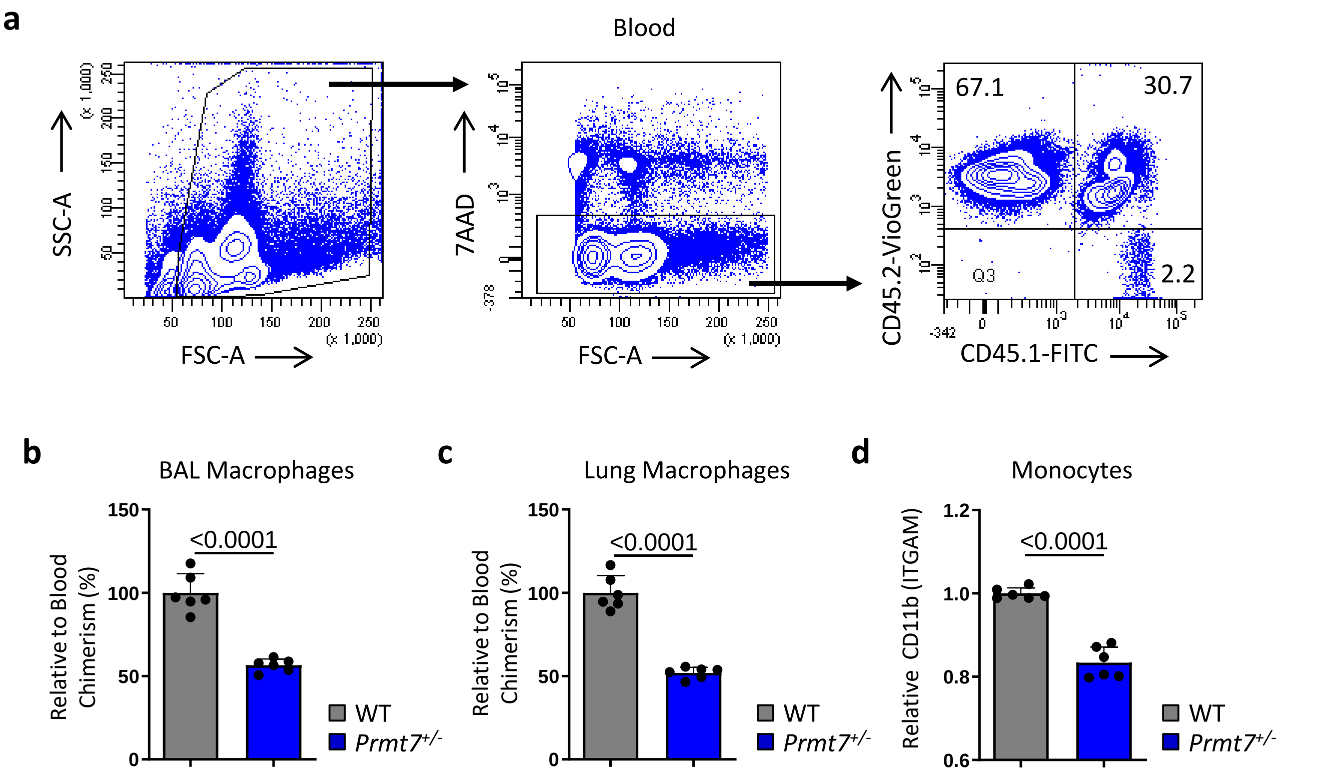

Supplementary Figure 9. Impaired monocyte migration to the lungs from *Prmt7*$^{+/-}$ bone marrow in chimeric mice. Competitive bone marrow chimeric mice were generated in CD45.1 recipients that had been lethally irradiated and then reconstituted intravenously with 50% wild-type (WT) competitor bone marrow (CD45.1/2 heterozygous) and 50% *Prmt7*$^{+/-}$ bone marrow (CD45.2) (n=6 mice) and analysed by flow cytometry 8 weeks later. **a**, Representative flow cytometry analysis of CD45 populations in the peripheral blood. **b-c**, Relative frequency of F/480+ macrophages originating from WT (CD45.1/2) or *Prmt7*$^{+/-}$ (CD45.2) bone marrow in the bronchoalveolar lavage (BAL) (b) and lung (c) compared to the blood. **d**, Relative surface expression of CD11b (ITGAM) on CD11b^{+} Ly6g^{-} lung monocytes originating from WT (CD45.1/2) or *Prmt7*$^{+/-}$ (CD45.2) bone marrow, relative to WT. Data shown mean ± SD, *P* values shown in charts determined by unpaired two-tailed Student's t test. Source data are provided as a Source Data file.

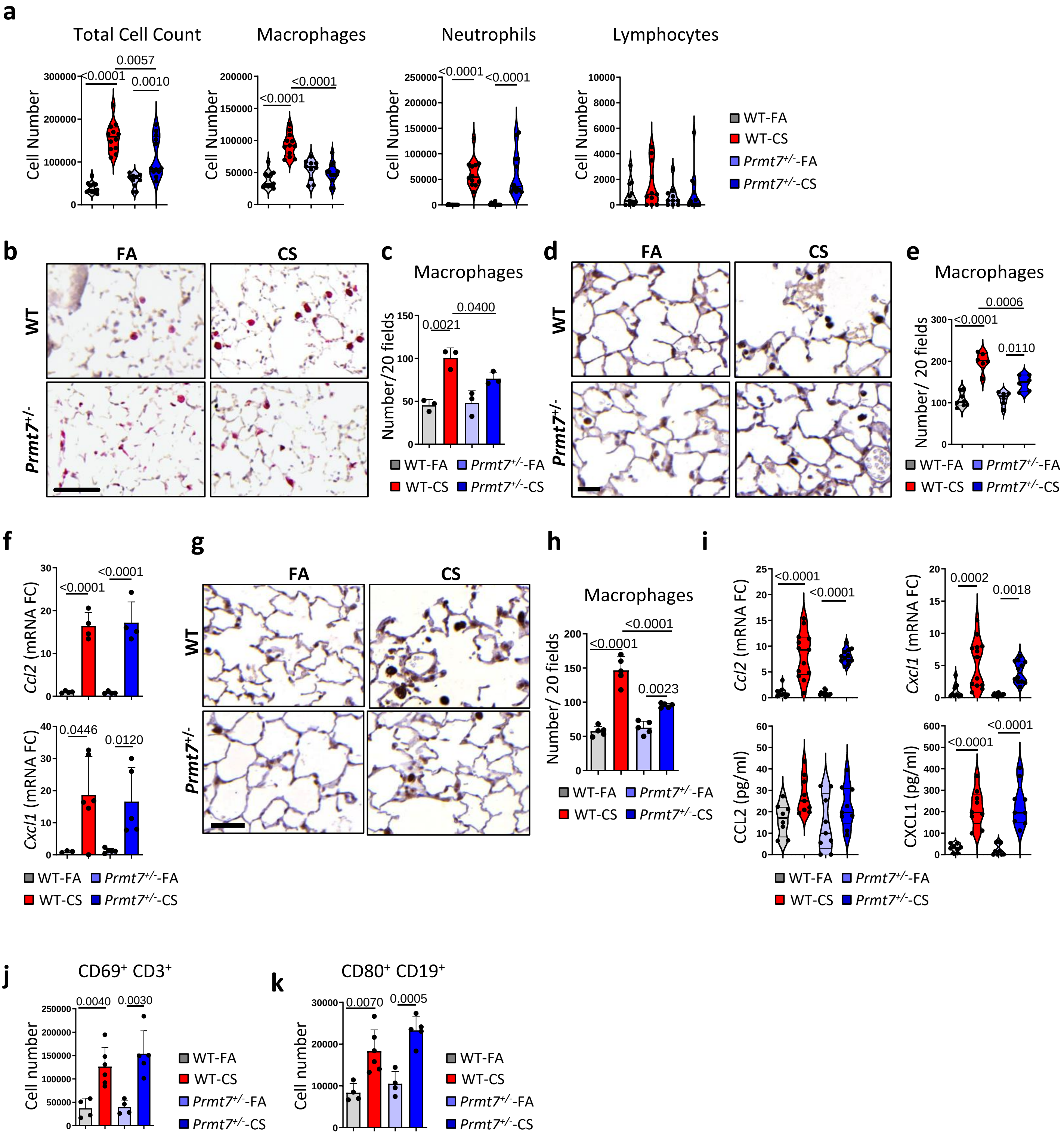

Supplementary Figure 10

Supplementary Figure 10. Macrophage accumulation in the lungs of *Prmt7*$^{+/-}$ mice is impaired following exposure to cigarette smoke. a-f, Wild-type (WT) and *Prmt7*$^{+/-}$ mice were exposed to filtered air (FA) or cigarette smoke (CS) for 3 days and analyzed on day 4 (n=10-12 per group, from three independent experiments). **a**, Bronchoalveolar lavage fluid (BALF) total, macrophage, neutrophil and lymphocyte cell counts (n=11 for WT FA, n=12 for WT CS, n=10 for *Prmt7*$^{+/-}$ FA and n=12 for *Prmt7*$^{+/-}$ CS). **b**, Representative images of immunohistochemical analysis for Galectin 3 positive macrophages (red signal, hematoxylin counter stained, scale bar 100μm) in lung sections. **c**, Quantification of macrophage number in lung sections (n=3 per group) from (b) given as number of positive cells per 20 random fields of view. **d**, Representative images of immunohistochemical analysis for CD68 positive macrophages (brown signal, hematoxylin counter stained, scale bar 25μm) in lung sections. **e**, Quantification of macrophage number in lung sections (n=8 for WT FA and n=6 for WT CS, *Prmt7*$^{+/-}$ FA, *Prmt7*$^{+/-}$ CS) from (d) given as number of positive cells per 20 random fields of view. **f**, mRNA expression levels of *Ccl2* (n=4 per group) and *Cxcl1*(n=3 for WT FA, n=6 for WT CS, n=5 for *Prmt7*$^{+/-}$ FA and *Prmt7*$^{+/-}$ CS) determined by qPCR in whole lung relative to WT FA controls. **g-k**, WT and *Prmt7*$^{+/-}$ mice were exposed to FA or CS for 4 months (n= 4-8 per group, repeated twice). **g**, Representative images of immunohistochemical analysis for CD68 positive macrophages (brown signal, hematoxylin counter stained, scale bar 25μm) in lung sections. **h**, Quantification of macrophage number in lung sections (n=5 per group) from (g) given as number of positive cells per 20 random fields of view. **i**, mRNA expression levels of *Ccl2* and *Cxcl1* determined by qPCR (n=9 for WT FA, n=13 for WT CS, n=12 for *Prmt7*$^{+/-}$ FA and *Prmt7*$^{+/-}$ CS) in whole lung relative to WT FA controls and protein levels of CCL2 and CXCL1 in BALF determined by ELISA (n=8 for WT FA, n=9 for WT CS, *Prmt7*$^{+/-}$ FA and *Prmt7*$^{+/-}$ CS). **j**, Quantification of total CD69^{+} CD3^{+} activated T cells in the lungs as determined by flow cytometry (n=4 for WT FA, n=6 for WT CS, n=4 for *Prmt7*$^{+/-}$ FA and n=5 for *Prmt7*$^{+/-}$ CS). **k**, Quantification of total CD80+ CD19+ activated B cells in the lungs as determined by flow cytometry (n=4 for WT FA, n=6 for WT CS, n=4 for *Prmt7*$^{+/-}$ FA and n=5 for *Prmt7*$^{+/-}$ CS). Data shown mean ± SD or violin plot with lines representing median and quartiles (a,e,i). *P* values shown in charts determined by one-way ANOVA Bonferroni's multiple comparisons test. Source data are provided as a Source Data file.

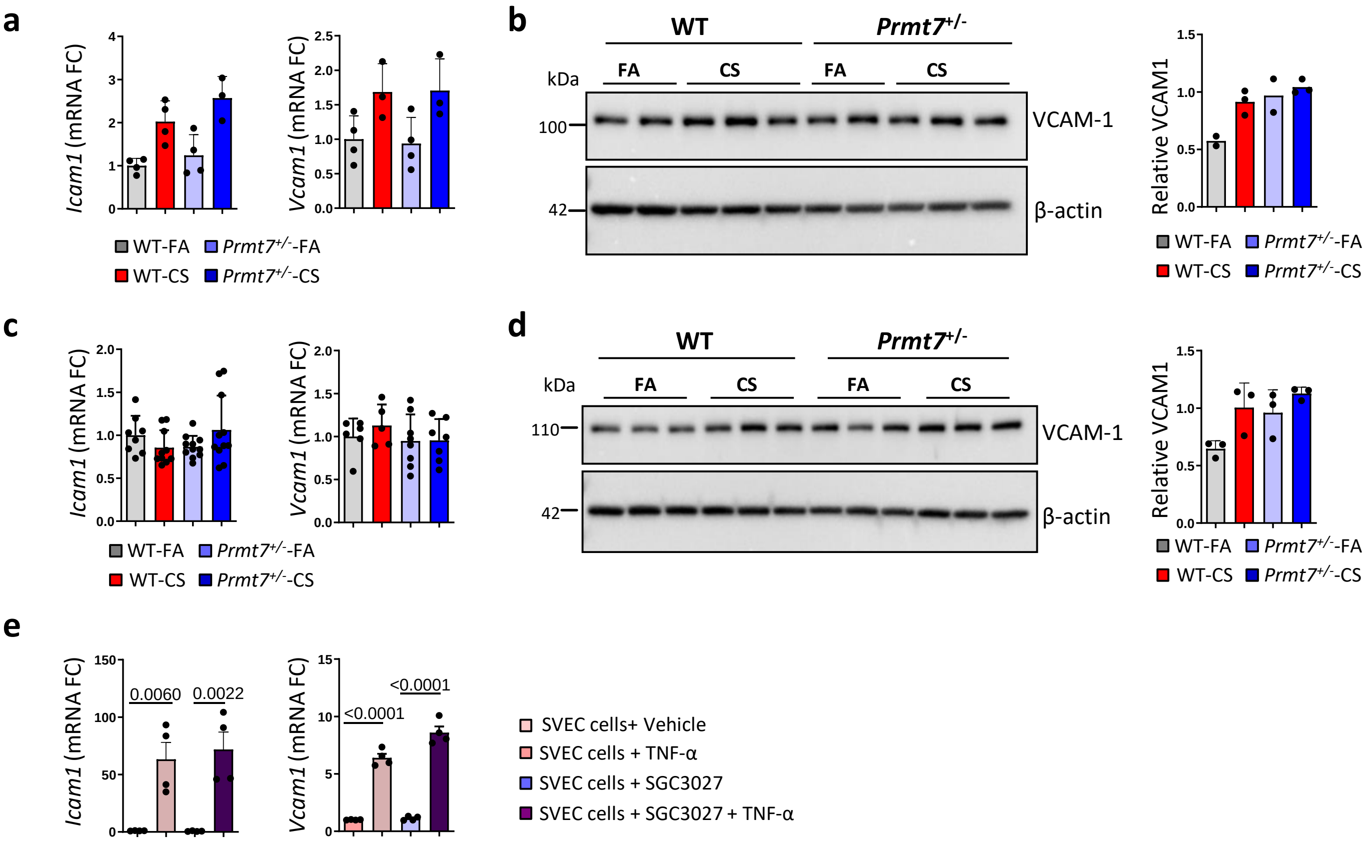

Supplementary Figure 11. Expression of ICAM1 and VCAM1 did not differ between the lungs of wild-type or *Prmt7*$^{+/-}$ mice. a-b, Wild-type (WT) and *Prmt7*$^{+/-}$ mice were exposed to filtered air (FA) or cigarette smoke (CS) for 3 days and analyzed on day 4. **a**, mRNA expression levels of *Icam1*(n=4 for WT FA, WT CS, *Prmt7*$^{+/-}$ FA and n=3 for *Prmt7*$^{+/-}$ CS) and *Vcam1* (n=4 for WT FA, n=3 for WT CS, n=4 for *Prmt7*$^{+/-}$ FA and n=3 for *Prmt7*$^{+/-}$ CS) by qPCR in whole lung relative to WT FA controls **b**, Western blot analysis of VCAM1 protein levels in whole lung. Quantification relative to β-actin shown (n=2/FA group and n=3/CS group). **c-d**, WT and *Prmt7*$^{+/-}$ mice were exposed to FA or CS for 4 months. **c**, mRNA expression levels of *Icam1* (n=8 for WT FA, n=10 for WT CS, n=11 for *Prmt7*$^{+/-}$ FA and *Prmt7*$^{+/-}$ CS) and *Vcam1* (n=6 for WT FA, n=5 for WT CS, n=8 for *Prmt7*$^{+/-}$ FA and n=7 for *Prmt7*$^{+/-}$ CS) by qPCR in whole lung relative to WT FA controls **d**, Western blot analysis of VCAM1 protein levels in whole lung. Quantification relative to β-actin shown (n=3/group). **e**, mRNA expression levels of *Icam1* and *Vcam1* by qPCR in SVEC4-10 endothelial cell lines with 1 h pretreatment of 5 μM SGC3027 (PRMT7 inhibitor) followed by TNF (100ng) stimulation for 24 h (n=4, from 2 independent experiments). Data shown mean ± SD, *P* values shown in chart determined by one-way ANOVA Bonferroni's multiple comparisons test (e). Uncropped blots can be found in Supplementary Figures 40-41. Source data are provided as a Source Data file.

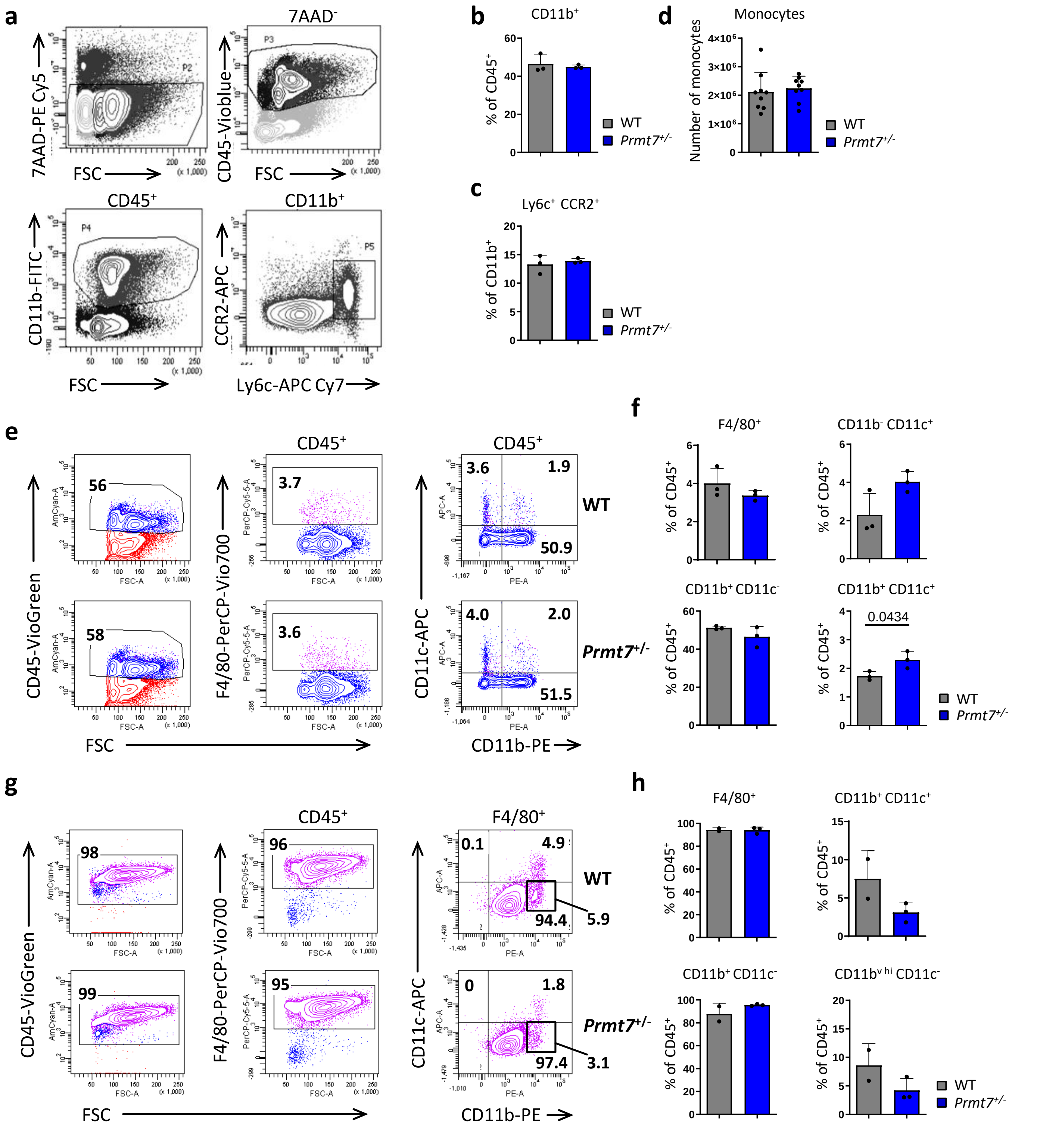

Supplementary Figure 12

Supplementary Figure 12. Monocyte and macrophage development is similar in WT and *Prmt7*$^{+/-}$ mice. a-c, Flow cytometry analysis of monocytes in freshly isolated bone marrow from wild-type (WT, n=3) and *Prmt7*$^{+/-}$ (n=3) mice **a**, gating structure. **b**, Percentage of CD11b^{+} cells as a fraction of the CD45 population. **c**, Percentage of Ly6c^{+} CCR2^{+} cells as a fraction of the CD11b^{+} population. **d**, Number of monocytes isolated from the bone marrow of WT (n=9) and *Prmt7*$^{+/-}$ (n=9) mice. **e**, Representative flow cytometry plots of freshly isolated bone marrow single cell suspensions from WT and *Prmt7*$^{+/-}$ mice to assess different subpopulations of macrophages gated according to CD45, F/480, CD11b and CD11c surface markers. **f**, Quantification of F/480^{+}, CD11b^{-} CD11c^{+}, CD11b^{+} CD11c^{-} and CD11b^{+} CD11c^{+} macrophages as shown in (**e**). (n=3 mice per group). **g**, Representative flow cytometry plots of 7 day bone marrow derived macrophages isolated from WT and *Prmt7*$^{+/-}$ mice to compare different subpopulations of macrophages gated according to CD45, F/480, CD11b and CD11c surface markers. **h**, Quantification of F4/80^{+}, CD11b^{-}CD11c^{+}, CD11b^{+} CD11c^{-} and CD11b^{+} CD11c^{+} macrophages as shown in (**g**). (n=3 mice per group). Data shown mean ± SD, *P* values shown in charts determined by unpaired two-tailed Student's t test. Source data are provided as a Source Data file.

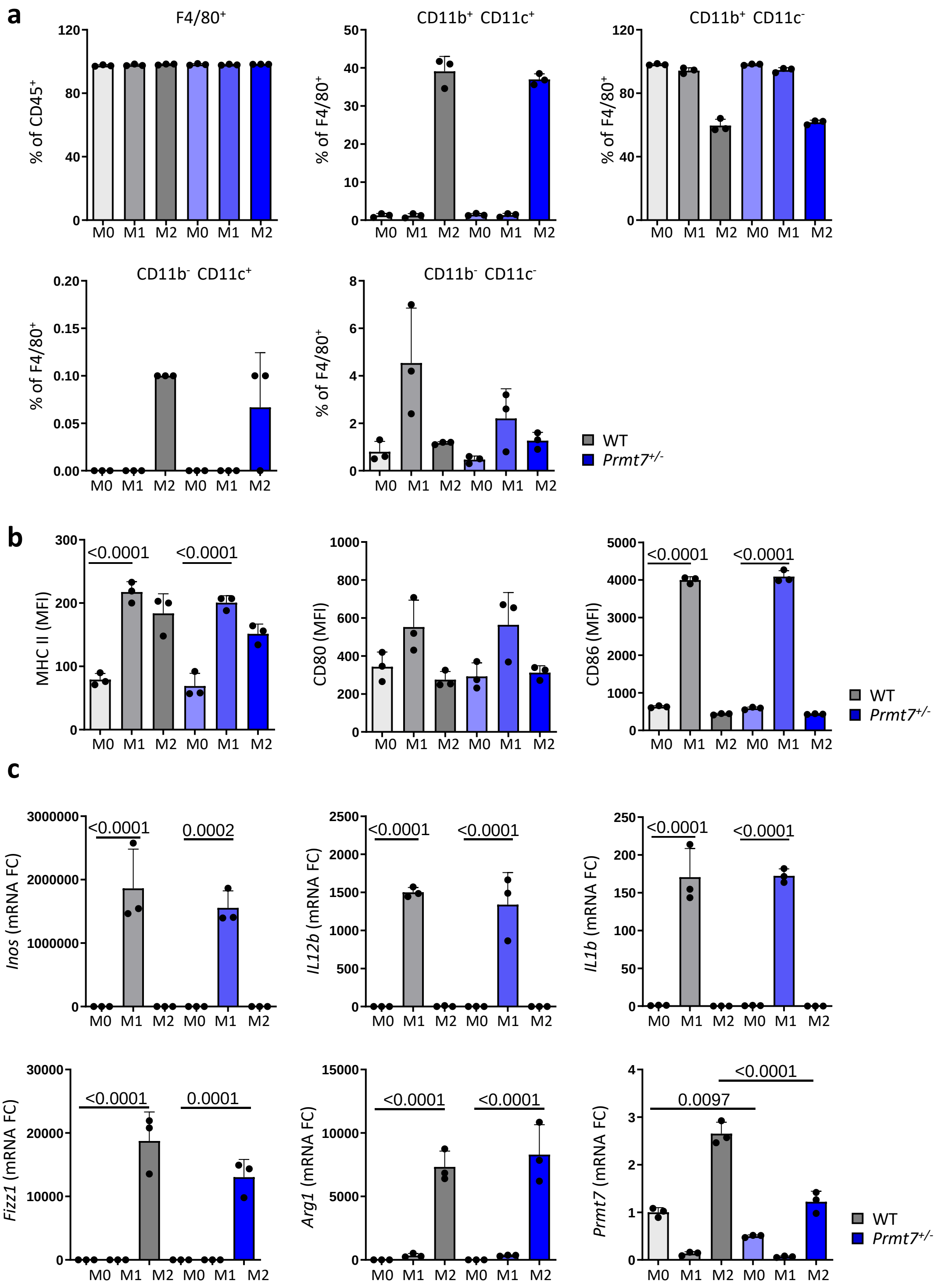

Supplementary Figure 13

Supplementary Figure 13. Macrophage activation and polarization is similar in WT and *Prmt7*$^{+/-}$ mice. a-c, 7 day bone marrow derived macrophages (BMDM) isolated from wild-type (WT, n=3) and *Prmt7*$^{+/-}$ (n=3) mice were treated with 1 μg/ml LPS plus 20 ng/ml IFNγ (M1), 20 ng/ml IL-4 (M2) or medium alone (M0) for 24 h. **a**, Quantification of F/480^{+} cells as a fraction of CD45^{+} cells and quantification of CD11b^{-} CD11c^{+}, CD11b^{+} CD11c^{-} and CD11b^{+} CD11c^{+} as a fraction of F/480^{+} BMDMs following flow cytometry as described in Supplementary Figure 12. **b**, Mean fluorescence intensity (MFI) of MHCII, CD80 and CD86 on the surface of F/480^{+} BMDMs. **c**, mRNA expression levels of *Inos*, *Il12b*, *Il1b*, *Fizz1*, *Arg1* and *Prmt7* by qPCR relative to WT M0 BMDMs. Data shown mean ± SD. *P* values shown in charts determined by one-way ANOVA Tukey's multiple comparisons test (b,c). Source data are provided as a Source Data file.

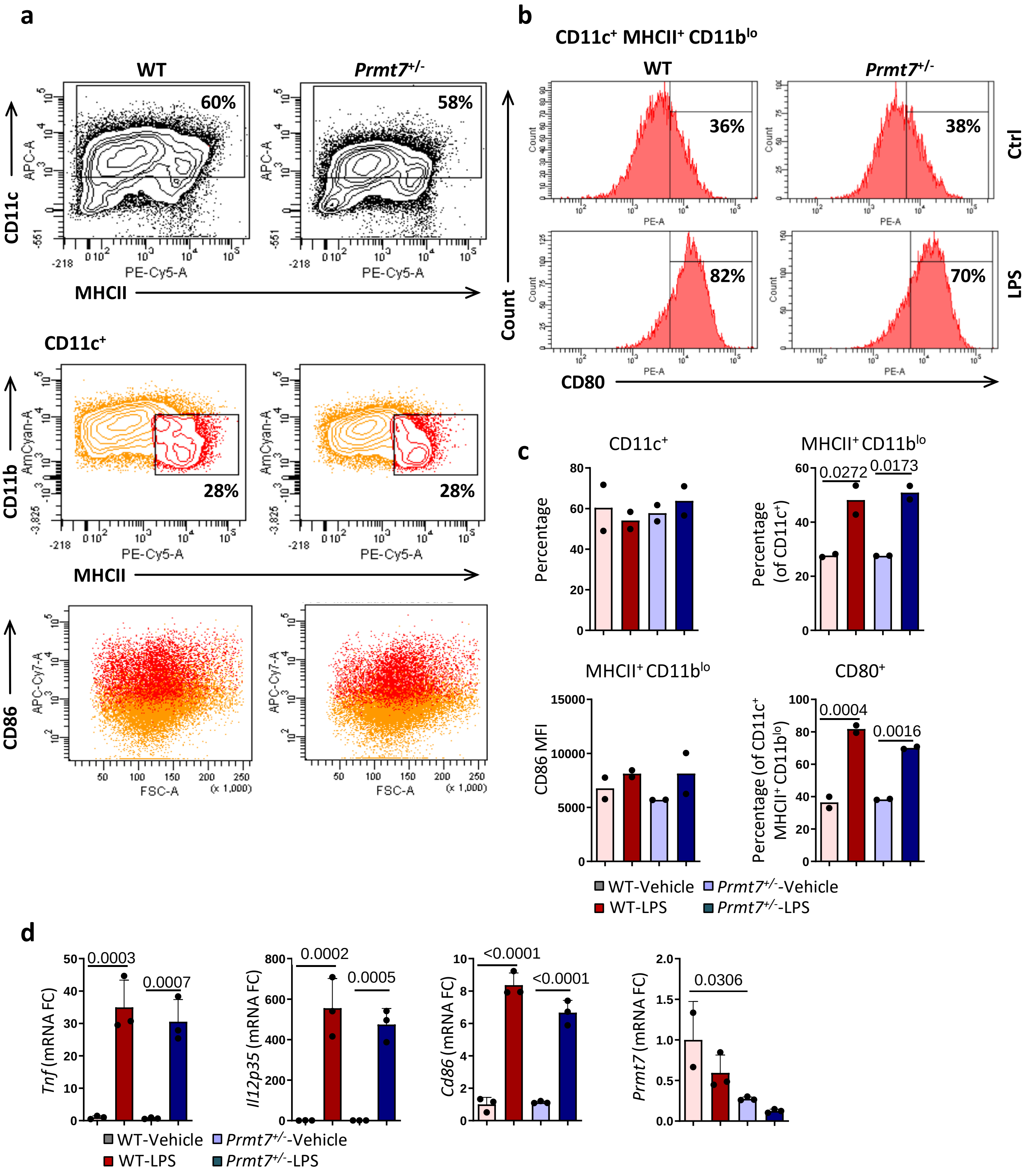

Supplementary Figure 14

Supplementary Figure 14. DC development and differentiation is similar in WT and *Prmt7*$^{+/-}$ mice. **a**, Representative flow cytometry plots of bone marrow derived dendritic cells (BMDC) isolated and differentiated from wild-type (WT) and *Prmt7*$^{+/-}$ mice for 7 d gated with CD11c, MHCII, CD11b and CD86 markers. **b**, Flow cytometry plot of CD80 MFI of CD11c^{+} MHCII^{+} CD11b^{lo} BMDCs stimulated with 1 μg/ml LPS for 24 h. **c**, Quantification of the percentage of CD11c^{+}, MHCII^{+} Cd11b^{lo} and CD80^{+} DCs and quantification of MFI of CD86^{+} DCs upon LPS treatment (n=2 per group). **d**, mRNA expression levels of *Tnf*, *Il12p35*, *Cd86* and *Prmt7* determined by qPCR stimulated with 1 μg/ml LPS for 24 h relative to WT-vehicle (n=3 per group). Data shown mean ± SD, *P* values shown in charts determined by one-way ANOVA Tukey's multiple comparisons test. Source data are provided as a Source Data file.

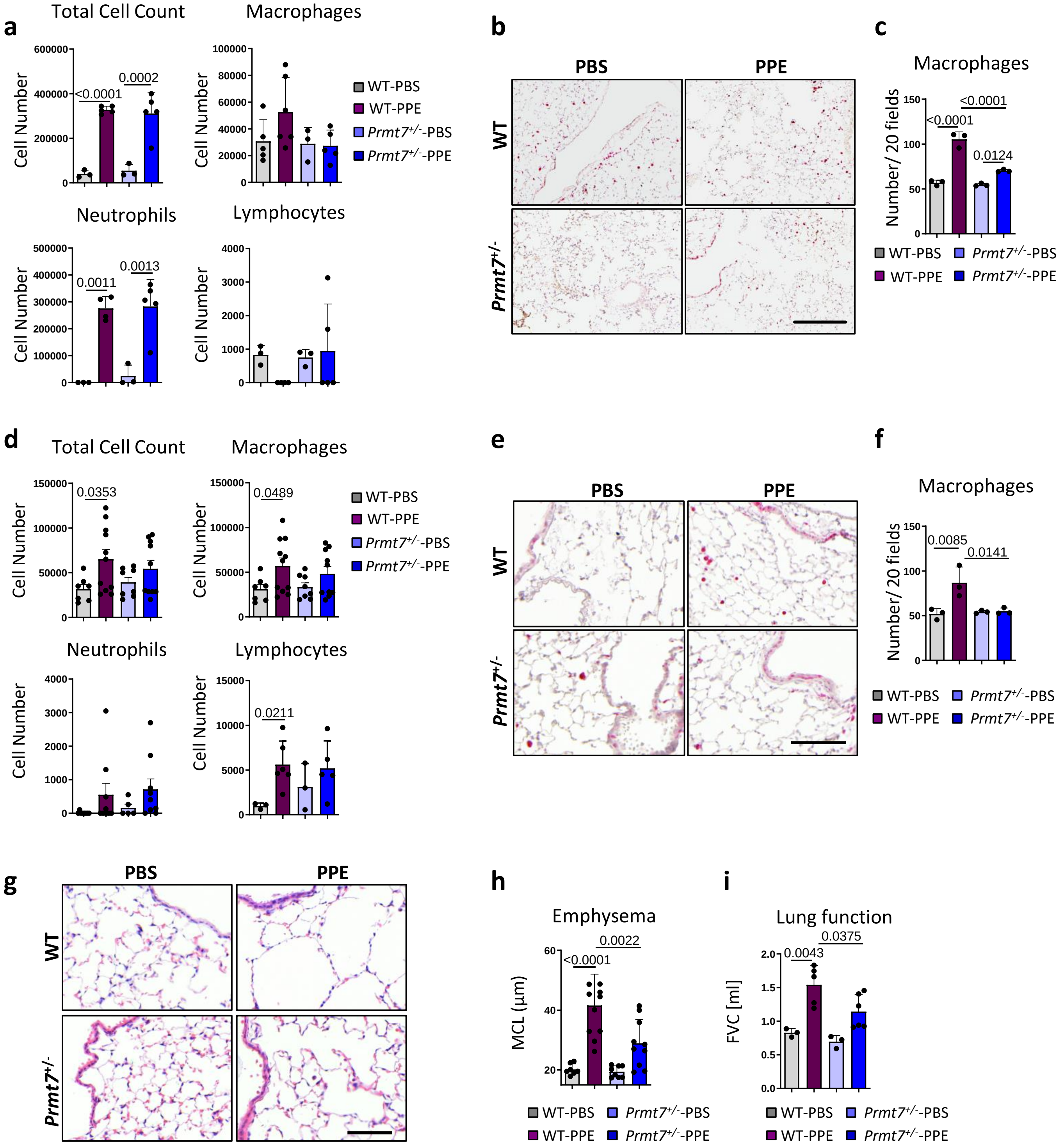

Supplementary Figure 15

Supplementary Figure 15. Elastase-induced macrophage accumulation and emphysema is impaired in the lungs of *Prmt7*$^{+/-}$ mice. a-c, Wild-type (WT) and *Prmt7*$^{+/-}$ mice were treated with a single oropharyngeal application of PBS or porcine pancreatic elastase (PPE) 40 U/Kg and analysed 24 h later (n=3-5 per group). **a**, Bronchoalveolar lavage fluid (BALF) total and differential cell counts (n=3 for WT PBS, n=5 for WT PPE, n=3 for *Prmt7*$^{+/-}$ PBS and n=5 for *Prmt7*$^{+/-}$ PPE). **b**, Representative images of immunohistochemical analysis for Galectin 3 positive macrophages (red signal, hematoxylin counter stained, scale bar 200µm) in lung sections. **c**, Quantification of macrophage number in lung sections (n=3 per group) from (b) given as number of positive cells per 20 random fields of view. **d**-**i**, WT and *Prmt7*$^{+/-}$ mice were treated with a single oropharyngeal application of PBS or PPE (porcine pancreatic elastase) 40 U/Kg and analyzed 28 days later (n=4-6 per group, repeated twice). **d**, BALF total and differential cell counts from WT and *Prmt7*$^{+/-}$ mice (n=7 for WT PBS, n=11 for WT PPE, n=8 for *Prmt7*$^{+/-}$ PBS and n=10 for *Prmt7*$^{+/-}$ PPE). **e**, Representative images of immunohistochemical analysis for Galectin 3 positive macrophages (red signal, hematoxylin counter stained, scale bar 200µm) in lung sections. **f**, Quantification of macrophage number in lung sections (n=3 per group) from (e) given as number of positive cells per 20 random fields of view. **g,** Representative images of Hematoxylin and Eosin (H&E) stained lung sections (scale bar 50µm). **h**, Quantification of airspace enlargement (emphysema) as mean chord length (MCL) in lung sections (n=7 for WT PBS, n=11 for WT PPE, n=8 for *Prmt7*$^{+/-}$ PBS and n=10 for *Prmt7*$^{+/-}$ PPE) from (g). **i**, Lung function measured as forced vital capacity (FVC) (n=3 for WT PBS, n=5 for WT PPE, n=3 for *Prmt7*$^{+/-}$ PBS and n=6 for *Prmt7*$^{+/-}$ PPE). Data shown mean ± SD, *P* values shown in charts determined by one-way ANOVA Bonferroni's multiple comparisons test (a, c, f, h and i), unpaired two-tailed Student's t test (d). Source data are provided as a Source Data file.

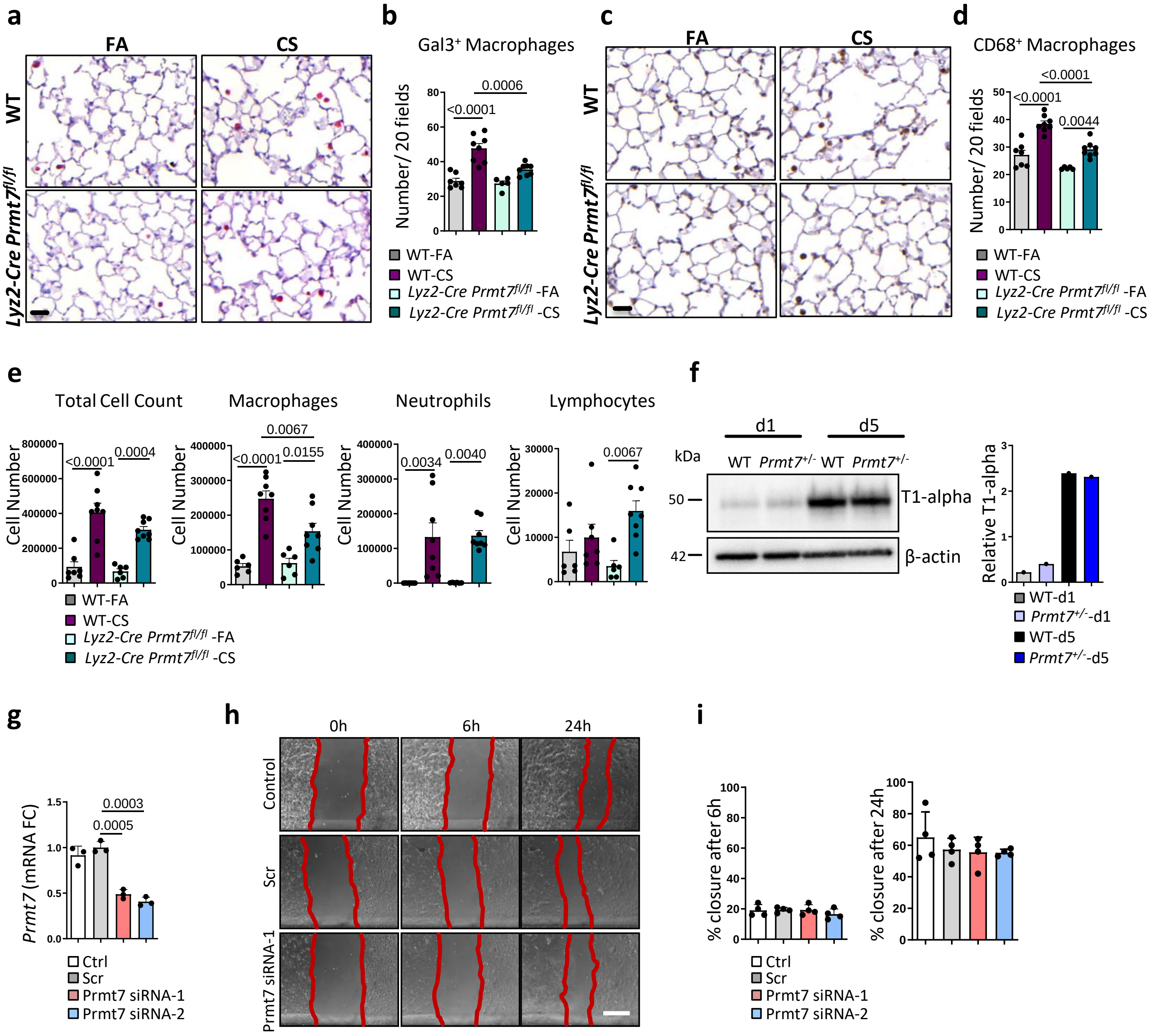

Supplementary Figure 16

Supplementary Figure 16. Cigarette smoke-induced macrophage accumulation is impaired in the lungs of *Lyz2-Cre Prmt7*$^{fl/fl}$ mice. a-e, Wild-type (WT) and *Lyz2-Cre Prmt7*$^{fl/fl}$ mice were exposed to filtered air (FA) or cigarette smoke (CS) for 3 days and analyzed on day 4 (n=5-8 mice/group). **a**, Representative images of immunohistochemical analysis for Galectin 3 positive macrophages (red signal, hematoxylin counter stained, scale bar 25 µm) in lung sections. **b**, Quantification of macrophage number in lung sections (n=7 for WT FA, n=8 for WT CS, n=5 for *Lyz2- Cre Prmt* $^{fl/fl}$ FA and n=8 for *Lyz2- Cre Prmt* $^{fl/fl}$ CS) from (a) given as number of positive cells per 20 random fields of view. **c**, Representative images of immunohistochemical analysis for CD68 positive macrophages (brown signal, hematoxylin counter stained, scale bar 25µm) in lung sections. **d**, Quantification of macrophage number in lung sections (n=7 for WT FA, n=8 for WT CS, n=5 for *Lyz2- Cre Prmt* $^{fl/fl}$ FA and n=8 for *Lyz2- Cre Prmt* $^{fl/fl}$ CS) from (c) given as number of positive cells per 20 random fields of view. **e**, Bronchoalveolar lavage fluid (BALF) total and differential cell counts (n=7 for WT FA, n=8 for WT CS, n=6 for *Lyz2- Cre Prmt* $^{fl/fl}$ FA and n=8 for *Lyz2- Cre Prmt* $^{fl/fl}$ CS). **f**, Western blot analysis of T1-alpha (podoplanin) levels as a marker for alveolar type I cells at d1 and d5 after culturing primary alveolar epithelial type II cells isolated from the lungs of wild-type (WT) and *Prmt7*$^{+/-}$ mice (3 mice per genotype). Expression relative to β-actin shown (n=1 per group). **g**, mRNA expression level of *Prmt7* in the murine alveolar epithelial type II cell line MLE12 left untreated (Ctrl), treated with scramble siRNA (Scr), or two siRNAs targeted against *Prmt7* (siRNA-1 and siRNA-2) (n=3 per group). **h-i**, Wound healing assay in confluent cultures of MLE12 cells left untreated (Ctrl), treated with scramble siRNA (Scr), or two siRNAs targeted against *Prmt7* (siRNA-1 and siRNA-2). Cells were scratched with a p200 pipette tip and photographed at time 0, 6 and 24 h (n=4 per group, repeated three times). Representative images shown (**h**), wound outlined in red. Scale bar, 200 µm. Wound surface area was measured and percentage wound closure at 6 h and 24 h calculated (**i**). Data shown mean ± SD, *P* values shown in charts determined by one-way ANOVA Bonferroni's multiple comparisons test (b, d-e), unpaired two-tailed Student's t test (g). Uncropped blots can be found in Supplementary Figure 42. Source data are provided as a Source Data file.

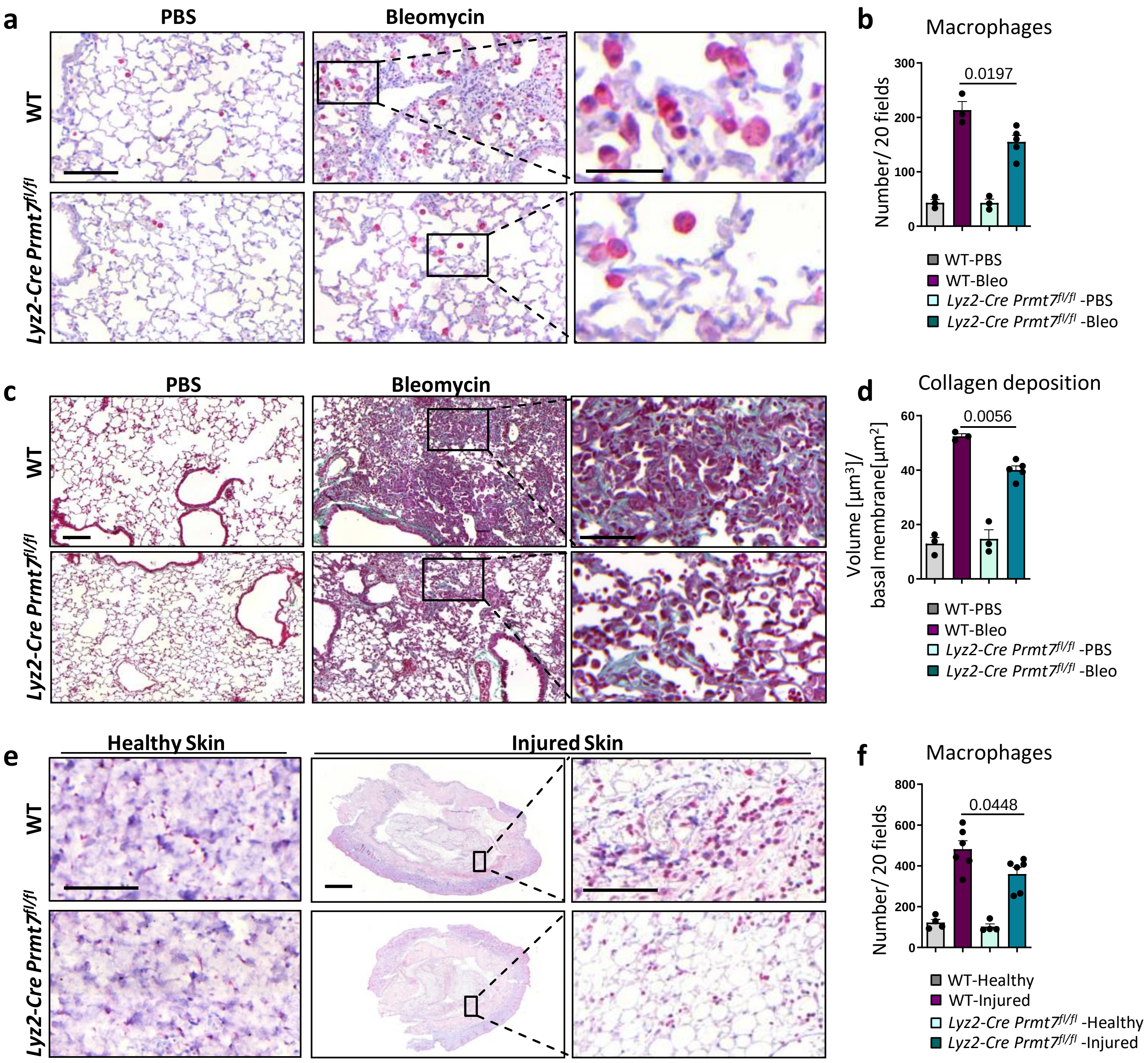

Supplementary Figure 17

Supplementary Figure 17. PRMT7 regulates monocyte migration in multiple disease models. **a-d,** Wild-type (WT) and *Lyz2-Cre Prmt7*$^{fl/fl}$ mice were treated with a single oropharyngeal application of PBS or bleomycin 2 U/Kg and analyzed 14 days later (n=3-5 per group). **a**, Representative images of immunohistochemical analysis for Galectin 3 positive macrophages (red signal, hematoxylin counter stained, scale bar 100µm) in lung sections. **b**, Quantification of macrophage number in lung sections (n=3 for WT PBS, WT Bleo, *Lyz2- Cre Prmt* $^{fl/fl}$ PBS and n=5 for *Lyz2- Cre Prmt* $^{fl/fl}$ Bleo) from (a) given as number of positive cells per 20 random fields of view. **c**, Representative images of Masson's trichrome stained lung sections (scale bar 100µm). **d**, Quantification of collagen deposition in the lung sections (n=3 for WT PBS, WT Bleo, *Lyz2- Cre Prmt* $^{fl/fl}$ PBS and n=5 for *Lyz2- Cre Prmt* $^{fl/fl}$ Bleo) stained in (c) as volume of collagen relative to the surface area of airway and vessel basement membranes. **e-f**, 5 mm splinted full-thickness excisional dorsal wounds were undertaken on WT and *Lyz2-Cre Prmt7*$^{fl/fl}$ mice and analyzed 3 days later with adjacent normal full-thickness skin biopsied as a control (n=6 wounds per genotype, n=4 normal biopsies). **e**, Representative images of wounds and adjacent normal skin stained for Galectin 3 positive macrophages (red signal, hematoxylin counter stained, scale bar 1 mm low magnification, 100 µm high magnification). **f**, Quantification of macrophage number in sections of wounded or healthy skin from (e) given as number of positive cells per 20 random fields of view. Data shown mean ± SD, *P* values shown in charts determined by one-way ANOVA Bonferroni's multiple comparisons test (b, d, f). Source data are provided as a Source Data file.

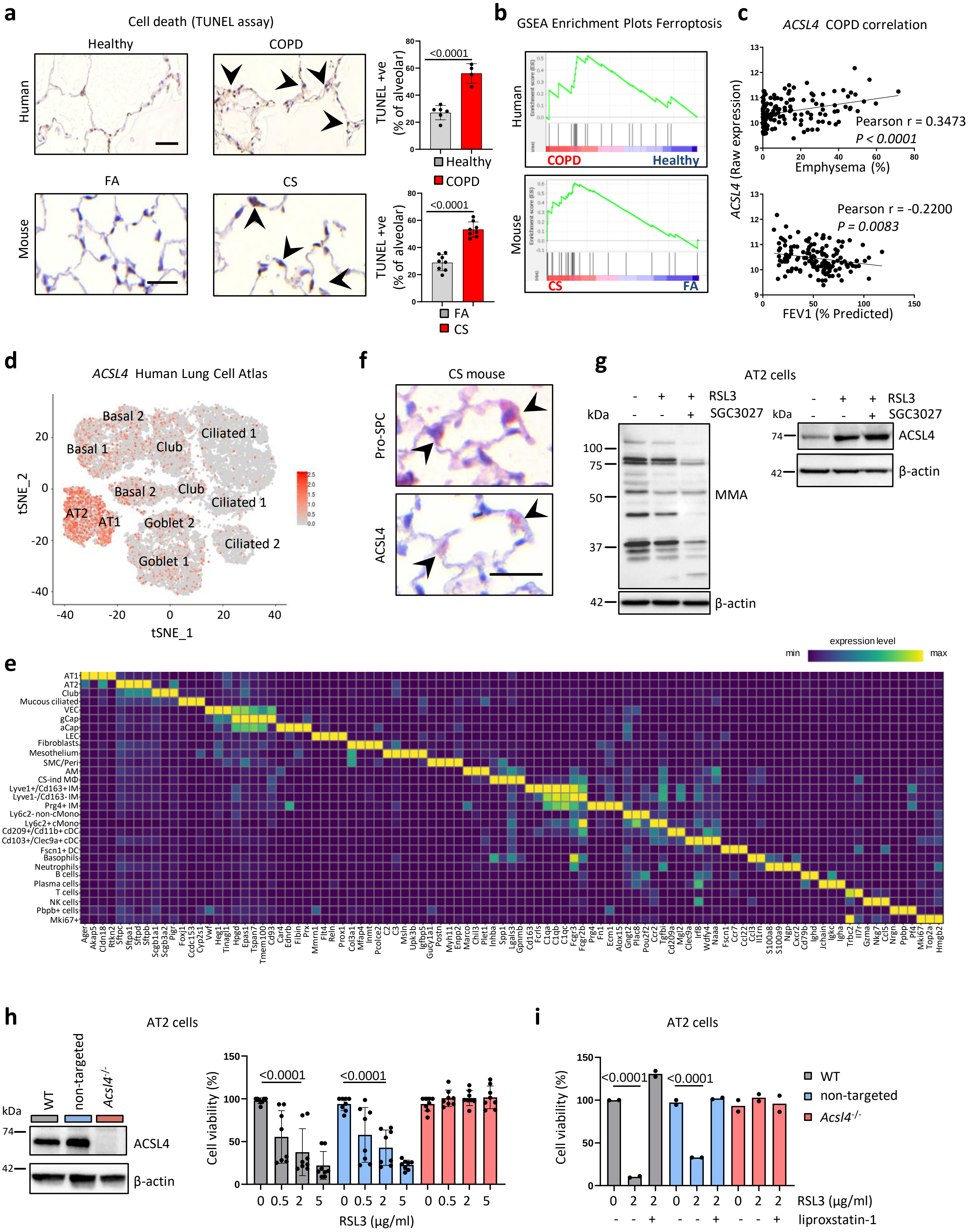

Supplementary Figure 18

Supplementary Figure 18. ACSL4 expression correlates with COPD severity. a, Representative images of TUNEL staining (brown signal dead cells indicated by arrow head, hematoxylin counter stained, scale bar 25 µm for mouse and 50 µm for human) in lung sections from COPD patients (n=4) and healthy controls (n=4) as well as from 4 m cigarette smoke (CS, n=4) and filtered air (FA, n=4) exposed mice. Quantification of alveolar epithelial cells TUNEL positive indicated. **b**, Enrichment plot for ferroptosis following GSEA on array data from lung tissue of COPD patients (GSE47460-GPL14550; 145 COPD patients v 91 healthy controls) and mice exposed to 4 m chronic cigarette smoke (CS, n=3) v filtered air (FA, n=3). **c**, *ACSL4* expression relative to emphysema severity and FEV1 from array data of lung tissue and clinical scores of COPD patients (n=145, GSE47460-GPL14550). **d**, tSNE plot of ACSL4 expression in human lung epithelial cells taken from the Human Lung Cell Atlas (https://lungcellatlas.org). **e**, Heat map depicting the expression of key genes used in identifying individual cell populations following scRNA-Seq (Drop-Seq) analysis of whole lung suspensions from mice exposed to FA (n=3) or CS for 4 m (n=5). AM, alveolar macrophages; CS-ind MØ, CS-induced macrophages; IM, interstitial macrophages; cMono, classical monocytes.. **f**, Representative images of immunohistochemical analysis for Pro-SPC and ACSL4 (red signal indicated by arrow head, hematoxylin counter stained, scale bar 25µm) in serial lung sections from mice exposed to CS for 4 m (n=4). **g**, Western blot analysis of monomethylation (MMA) and ACSL4 in AT2 cells (MLE12) 24 h pre-treatment with or without 10 µM of PRMT7 inhibitor, SGC3027, followed by 24 h treatment with RSL3 (500 nM) (repeated two times). **h**, Western blot analysis of ACSL4 expression in WT MLE12 cells, non-targeted MLE12 cells and *Acsl4*$^{-/-}$ MLE12 cells generated by CRISPR-Cas9 technology (Left). Cell viability (Aquabluer) of cells indicated 24 h after treatment with RSL3 (Right) (n=8) data combined from four independent experiments. **i**, Cell viability (Aquabluer) of WT, non-targeted and *Acsl4*$^{-/-}$ MLE12 cells 24 h after treatment with or without RSL3 (2 µM) and Liproxstatin-1 (250 nM), (n=2) representative of three independent experiments. Data shown mean ± SD, *P* values shown in charts determined by one-way ANOVA Tukey's multiple comparisons test (h-i), unpaired two-tailed Student's t test (a). Uncropped blots can be found in Supplementary Figures 43-44. Source data are provided as a Source Data file.

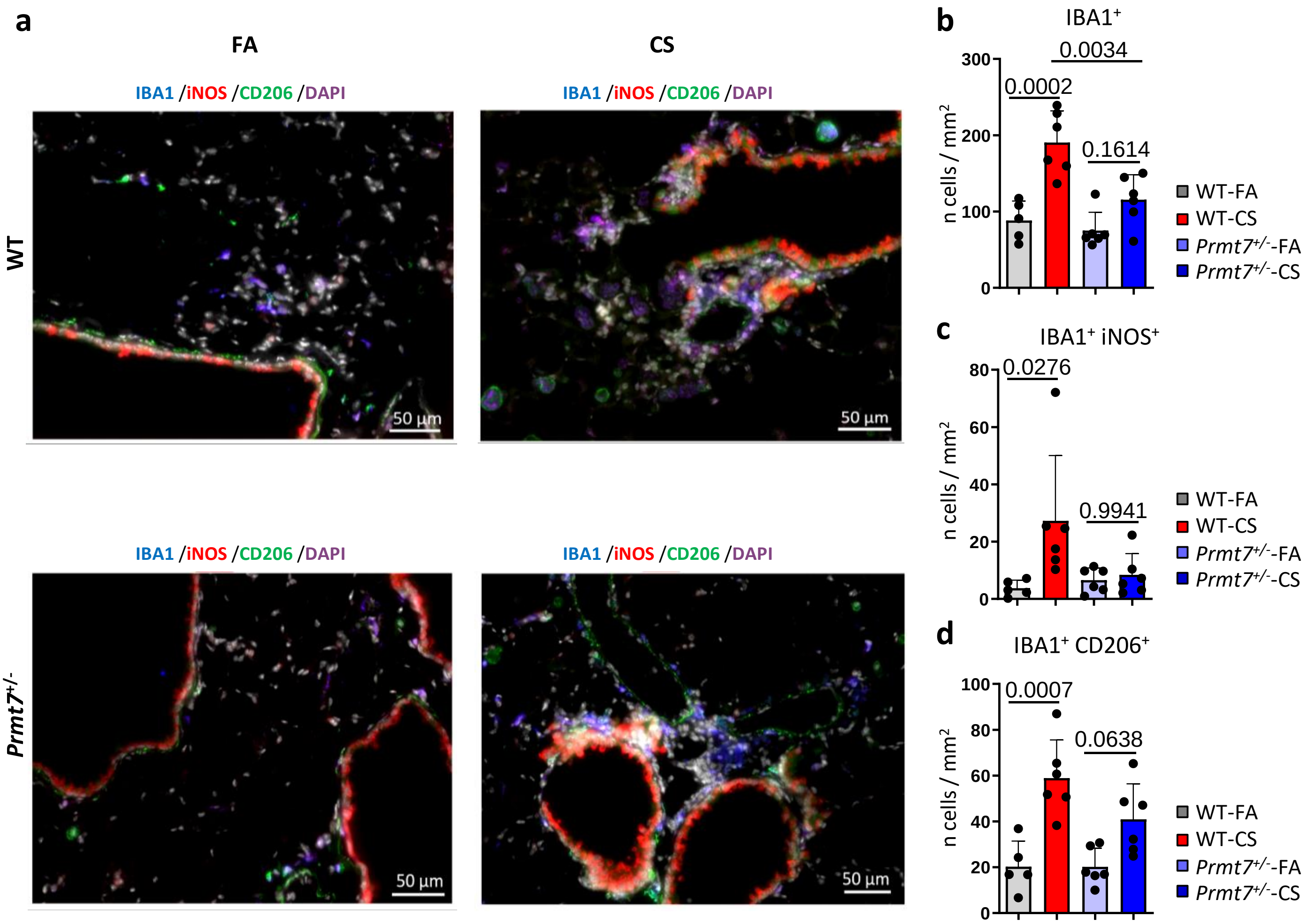

Supplementary Figure 19. Inflammatory status of macrophages in the lungs of CS-exposed *Prmt7*$^{+/-}$ mice. a, Representative images of Multiplex immunofluorescence staining to identify IBA1 (turquoise), iNOS (red), CD206 (green) and DAPI (purple) counterstained lung sections from WT and *Prmt7*$^{+/-}$ mice exposed to filtered air (FA) or cigarette smoke (CS) for 4 months (n = 5-6 mice per group). Scale bars 50 µm. **b-d**, Quantification of IBA1 positive macrophages (n=5 for WT FA and n=6 for WT CS, *Prmt7*$^{+/-}$ FA, *Prmt7*$^{+/-}$ CS) (**b**), iNOS and IBA1 double-positive macrophages (n=5 for WT FA and n=6 for WT CS, *Prmt7*$^{+/-}$ FA, *Prmt7*$^{+/-}$ CS) (**c**) and CD206 and IBA1 double-positive macrophages (n=5 for WT FA and n=6 for WT CS, *Prmt7*$^{+/-}$ FA, *Prmt7*$^{+/-}$ CS) (**d**) from Multiplex immunofluorescence staining described in (a) using Ilastik and CellProfiler. Data shown mean ± SD, P values shown in charts determined by one-way ANOVA Tukey's multiple comparisons test (b-d) and Pearson Correlation two-tailed (c). Source data are provided as a Source Data file.

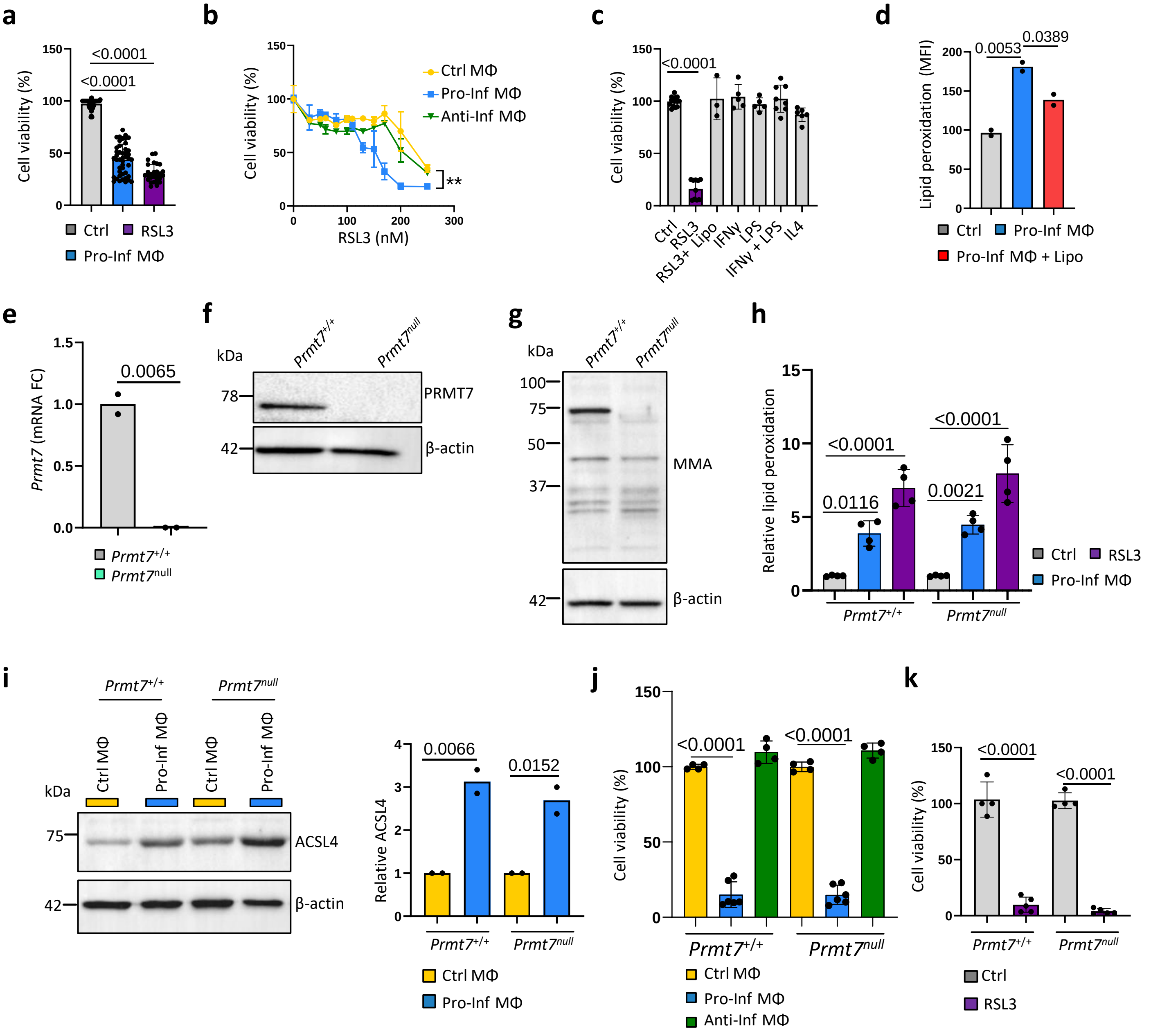

Supplementary Figure 20

Supplementary Figure 20. Inflammatory macrophages induce susceptibility to ferroptosis in lung alveolar epithelial cells independent of PRMT7. a, Cell viability (Aquabluer) of AT2 cells (MLE12) 24 h after treatment with RSL3 (250 nM, n=16) or conditioned medium from RAW264.7 macrophages polarized to a pro-inflammatory phenotype (n=16) from 8 independent experiments**.** **b**, Cell viability (PI uptake) of AT2 cells at 24 h after treatment with RSL3 at the concentrations indicated, in the presence of conditioned medium from control RAW264.7 macrophages (n=2) or those polarized to a pro-inflammatory (n=2) or anti-inflammatory (n=2) phenotype, from two independent experiments**.** **c**, Cell viability (Aquabluer) of AT2 cells (MLE12) after 24 h following direct treatment with RSL3 (250 nM, n=10), RSL3+liproxstatin-1 (250 nM, n=3), IFNγ (20 ng/ml, n=5), LPS (1 μg/ml, n=5), IFNγ + LPS (n=8) or IL4 (20 ng/ml, n=6), from 3 independent experiments**. d,** BODIPY lipid peroxidation in AT2 cells (MLE12) treated for 6 h with conditioned medium from RAW264.7 macrophages polarized to a pro-inflammatory phenotype (n=2) +/- liproxstatin-1 (250 nM, n=2) from 2 independent experiments. **e**, mRNA expression level of *Prmt7* determined by qPCR in *Prmt7*$^{+/+}$ and *Prmt7*^{null} MLE12 cells generated by CRISPR/Cas9 relative to *Prmt7*$^{+/+}$ (n=2 replicates per cell line). **f**, Western blot analysis of PRMT7 expression in *Prmt7*$^{+/+}$ and *Prmt7*^{null} MLE12 cells (repeated two times). **g**, Western blot analysis of total mono-methylated arginine residues in *Prmt7*$^{+/+}$ and *Prmt7*^{null} MLE12 cells (repeated two times). **h**, BODIPY lipid peroxidation in *Prmt7*$^{+/+}$ and *Prmt7*^{null} MLE12 AT2 cells at 6 h after treatment with RSL3 (500 nM) or conditioned medium from RAW264.7 macrophages polarized to a pro-inflammatory phenotype relative to conditioned medium from control macrophages (n=4 from 2 independent experiments). **i**, Western blot analysis of ACSL4 expression in *Prmt7*$^{+/+}$ and *Prmt7*^{null} MLE12 cells treated for 24 h with conditioned medium from RAW264.7 macrophages polarized to a pro-inflammatory phenotype or control macrophages. Normalised to β-actin and shown relative to controls, from 2 independent experiments. **j**, Cell viability (Aquabluer) of *Prmt7*$^{+/+}$ and *Prmt7*^{null} MLE12 AT2 cells treated for 24 h with conditioned medium from control RAW264.7 macrophages (n=4) or those polarized to a pro-inflammatory (n=6) or anti-inflammatory phenotype (n=4) (2 independent experiments). **k,** Cell viability (Aquabluer) of *Prmt7*$^{+/+}$ and *Prmt7*^{null} MLE12 AT2 cells treated for 24 h with RSL3 (500 nM) (n=4-5, from 2 independent experiments). Data shown mean ± SD, *P* values shown in charts determined by two-way ANOVA (b) and one-way ANOVA Tukey's multiple comparisons test (a, c-d, h-k). Uncropped blots can be found in Supplementary Figures 45-47. Source data are provided as a Source Data file.

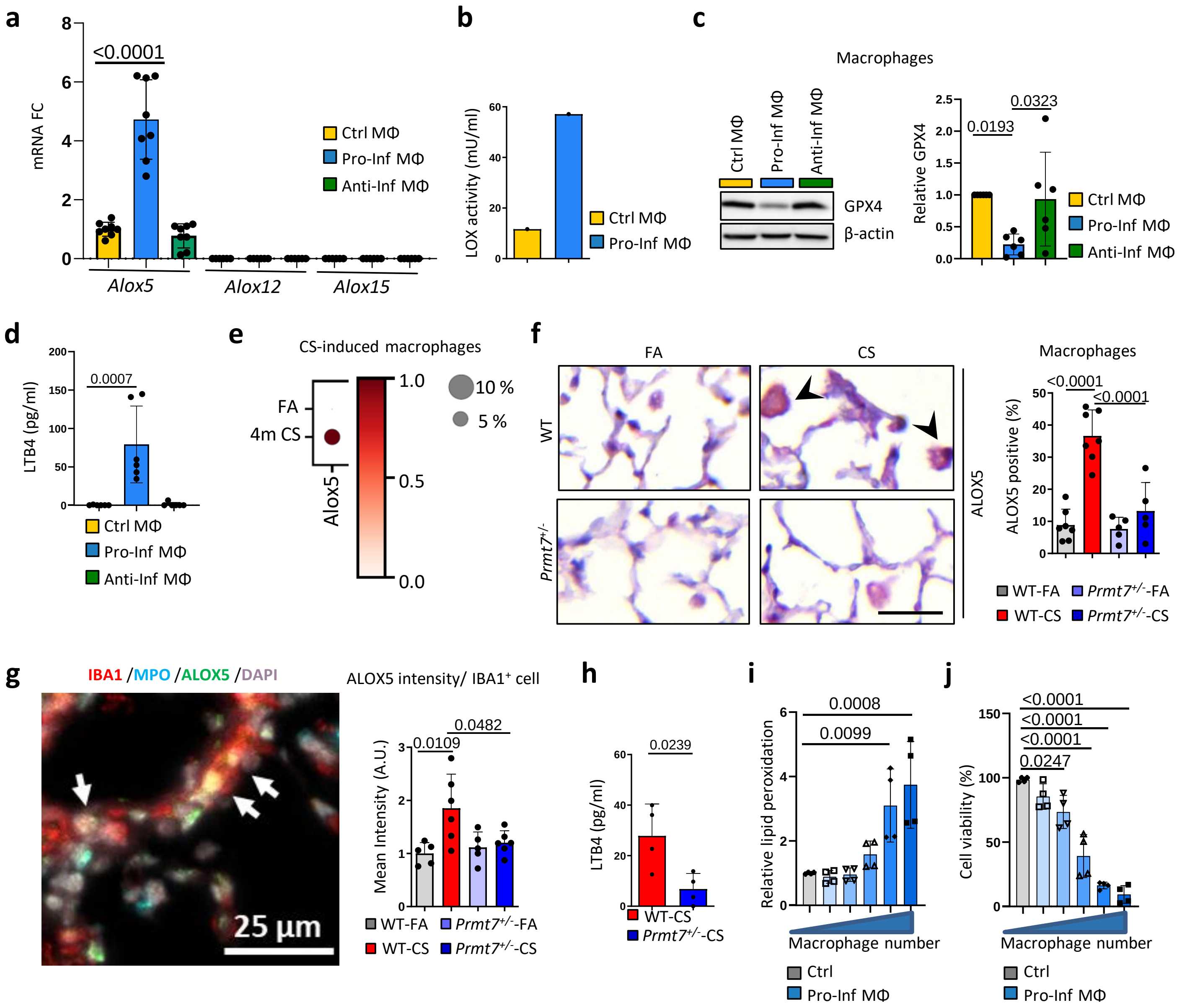

Supplementary Figure 21

Supplementary Figure 21. Increased ALOX5 expression in pro-inflammatory macrophages. **a**, mRNA expression level of *Alox5, Alox12 and Alox15* determined by qPCR at 72 h in control RAW264.7 macrophages (n=8) or those polarized to a pro-inflammatory (n=8) or anti-inflammatory (n=8) phenotype, relative to *Alox5* in control macrophages (from 4 independent experiments). **b**, Lipoxygenase activity at 72 h in control RAW264.7 macrophages or those polarized to a pro-inflammatory phenotype (from one experiment). **c**, Western blot analysis of GPX4 expression at 72 h in control RAW264.7 macrophages or those polarized to a pro-inflammatory or anti-inflammatory phenotype. Normalised to β-actin and shown relative to control macrophages, from 6 independent experiments. **d**, Leukotriene B4 (LTB4) levels detected by ELISA, at 72h in conditioned medium from control RAW264.7 macrophages or those polarized to a pro-inflammatory or anti-inflammatory phenotype (n=6, from 3 independent experiments). **e**, Dot blot depicting *Alox5* expression (log transformed, normalised UMI counts) and percentage of cells positive for *Alox5* in CS-induced macrophages from scRNA-Seq of whole lung suspensions from mice exposed to FA (n=3) or CS for 4m (n=5). **f**, Representative images of immunohistochemical analysis for ALOX5 (red signal indicated by arrow heads, hematoxylin counter stained, scale bar 25µm) in lung sections (n=7 for WT FA, WT CS, and n=5 for *Prmt7*$^{+/-}$ FA, *Prmt7*$^{+/-}$ CS) from WT and *Prmt7*$^{+/-}$ mice exposed to FA or CS for 4 months, and quantification of macrophages positive for ALOX5. **g**, Representative image of Multiplex immunofluorescence staining to identify IBA1 (red), MPO (turquoise), ALOX5 (green) and DAPI (purple) counterstained lung sections (n=5 for WT FA and n=6 for WT CS, *Prmt7*$^{+/-}$ FA, *Prmt7*$^{+/-}$ CS) (scale bar 25 µm) from WT and *Prmt7*$^{+/-}$ mice exposed to filtered air (FA) or cigarette smoke (CS) for 4 months Quantification of mean ALOX5 intensity per IBA1^{+} cell using Ilastik and CellProfiler. **h**, LTB4 levels detected by ELISA in the bronchoalveolar lavage fluid of wild-type (WT) and *Prmt7*$^{+/-}$ mice exposed to CS for 4 months (n=4 mice per group). **i-j**, RAW264.7 macrophages were cultured at increasing density (25 000, 50 000, 75 000, 100 000 and 150 000 cells/well) and polarized to a pro-inflammatory phenotype. **i**, BODIPY lipid peroxidation in AT2 cells (MLE12) 6 h after treatment with conditioned medium from the RAW264.7 macrophages polarized to a pro-inflammatory phenotype relative to control macrophages (150000 cells/well) (n=4, from two independent experiments). **j,** Cell viability (Aquabluer) of AT2 cells (MLE12) 24 h after treatment with conditioned medium from the RAW264.7 macrophages polarized to a pro-inflammatory phenotype relative to control macrophages (150000/well) (n=4, from two

independent experiments). Data shown mean ± SD, *P* values shown in charts determined by one-way ANOVA Tukey's multiple comparisons test (a-d, f-g, i-j), unpaired two-tailed Student's t test (h). Uncropped blots can be found in Supplementary Figure 48. Source data are provided as a Source Data file.

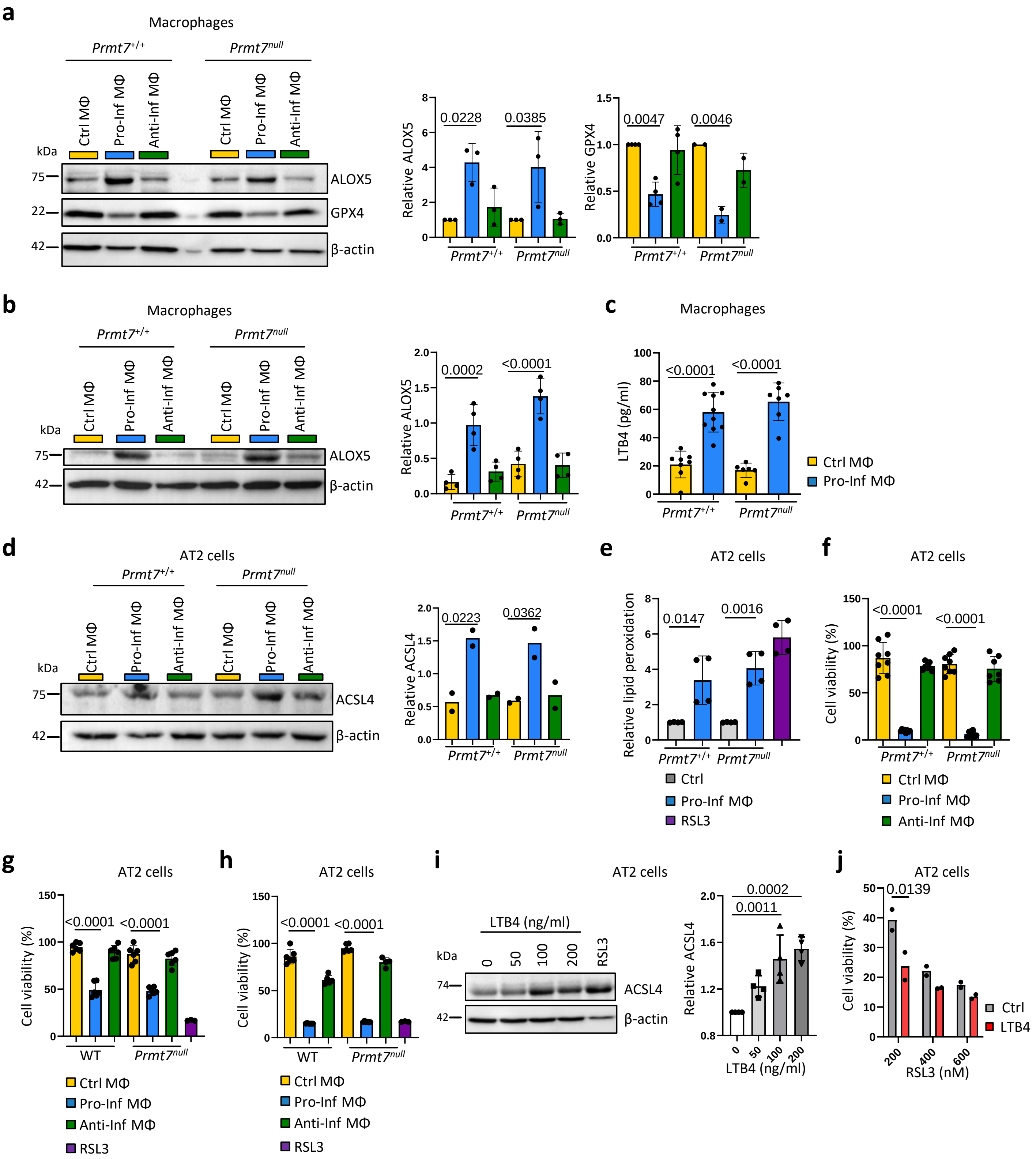

Supplementary Figure 22

Supplementary Figure 22. PRMT7-deficient macrophages retain the ability to upregulate ALOX5 and induce susceptibility to ferroptosis in lung alveolar epithelial cells. a, Western blot analysis of ALOX5 and GPX4 in *Prmt7*$^{+/+}$ and *Prmt7*^{null} MH-S macrophages at 72h polarized to a pro-inflammatory or anti-inflammatory phenotype. Normalised to β-actin and shown relative to controls, from 3 independent experiments. **b**, Western blot analysis of ALOX5 expression in *Prmt7*$^{+/+}$ and *Prmt7*^{null} RAW264.7 macrophages at 72h polarized to a pro-inflammatory or anti-inflammatory phenotype. Relative to β-actin, from 4 independent experiments. **c**, Leukotriene B4 (LTB4) levels detected by ELISA, at 72h in conditioned medium from control *Prmt7*$^{+/+}$ and *Prmt7*^{null} RAW264.7 macrophages or those polarized to a pro-inflammatory phenotype (n=8, from 4 independent experiments). **d**, Western blot analysis of ACSL4 expression in AT2 cells (MLE12) treated for 24 h with conditioned medium from control *Prmt7*$^{+/+}$ and *Prmt7*^{null} RAW264.7 macrophages or those polarized to a pro-inflammatory or anti-inflammatory phenotype. Relative to β-actin, from 2 independent experiments. **e**, BODIPY lipid peroxidation in AT2 cells (MLE12) 6 h after treatment with RSL3 (500 nM) or conditioned medium from control *Prmt7*$^{+/+}$ and *Prmt7*^{null} RAW264.7 macrophages or those polarized to a pro-inflammatory phenotype relative to control macrophages (n=4, from 2 independent experiments). **f**, Cell viability (Aquabluer) of AT2 cells (MLE12) 24 h after treatment with conditioned medium from control *Prmt7*$^{+/+}$ and *Prmt7*^{null} RAW264.7 macrophages or those polarized to a pro-inflammatory or anti-inflammatory phenotype (n=8, from 2 independent experiments). **g-h**, Cell viability (Aquabluer) of AT2 cells (MLE12) 24 h after treatment with RSL3(n=3) (500 nM) or conditioned medium from control bone marrow (n=6) isolated from wild-type (WT) and *Prmt7*$^{+/-}$ mice differentiated with MCSF (**g**) or GMCSF (**h**) and polarized to a pro-inflammatory (n=6 for MCSF and GMCSF treated) or anti-inflammatory phenotype (n=6 for MCSF and n=4 for GMCSF treated) (3 independent experiments). **i**, Western blot analysis of ACSL4 in AT2 cells (MLE12) treated for 24 h with LTB4 at the concentrations indicated, normalised to β-actin and shown relative to controls (from 4 independent experiments). **j**, Cell viability (Aquabluer) of AT2 cells (MLE12) 24 h after treatment with RSL3 at the concentrations indicated +/- LTB4 (200ng/ml) from 2 independent experiments**.** Data shown mean ± SD, *P* values shown in charts determined by one-way ANOVA Tukey's multiple comparisons test (a-i) and two-way ANOVA (j). Uncropped blots can be found in Supplementary Figures 49-52. Source data are provided as a Source Data file.

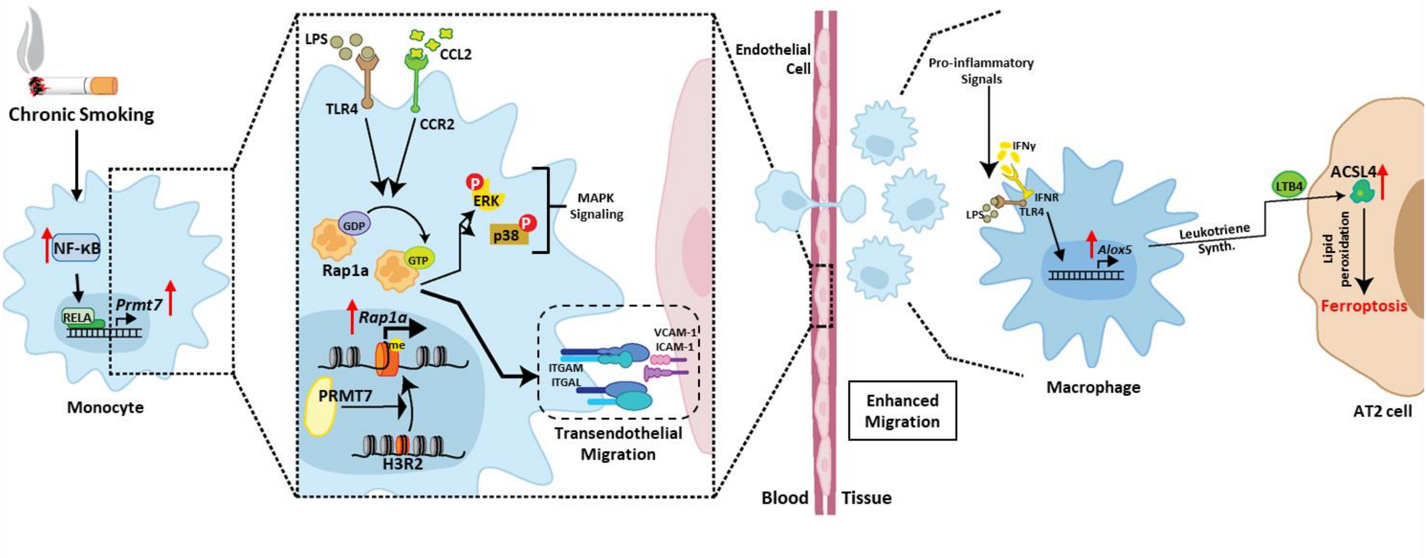

Supplementary Figure 23. Schematic representation of PRMT7 regulated monocyte transendothelial migration and subsequent sensitization of AT2 cells to ferroptosis. Inflammatory signals lead to NF-κB signalling and the upregulation of PRMT7. Histone arginine methylation imparted by PRMT7 enhances RAP1A expression which drives MAPK signalling, integrin activation and leucocyte transendothelial migration in response to chemokines such as CCL2. Subsequent accumulation of pro-inflammatory macrophages induces ALOX5 derived LTB4 which contributes to ferroptosis sensitization of AT2 cells in a paracrine manner.

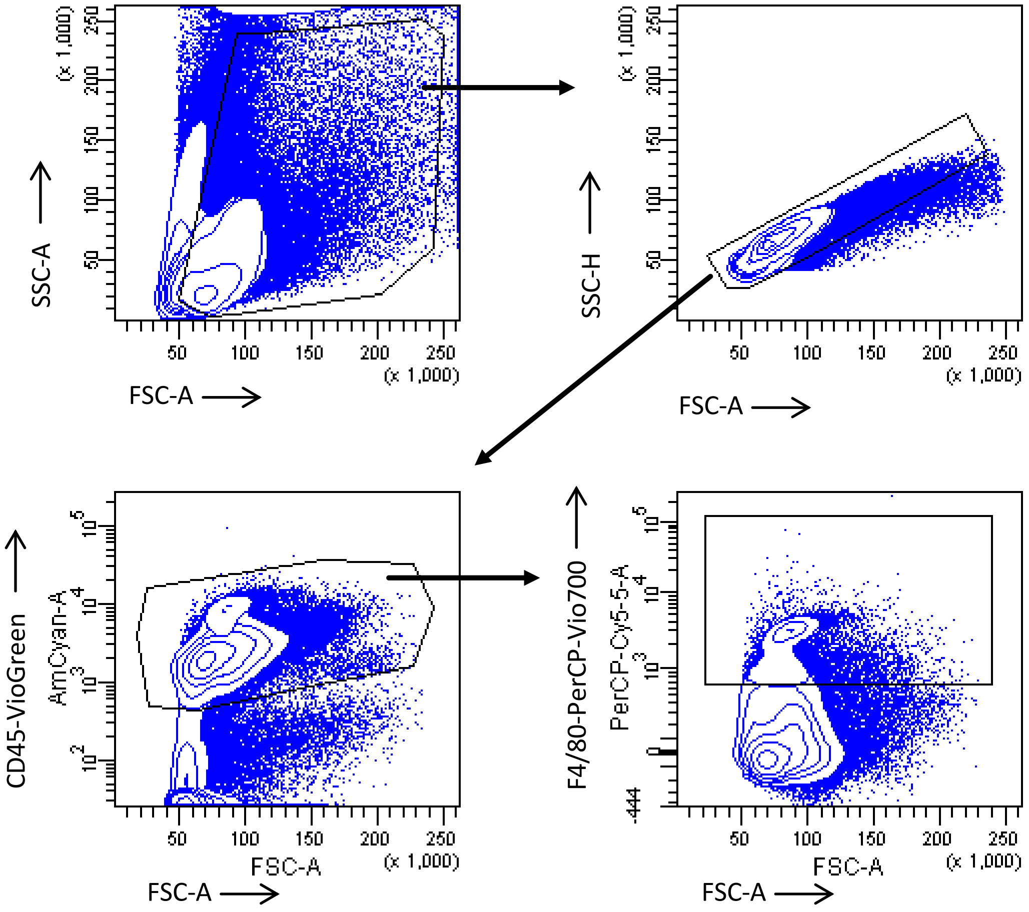

Supplementary Figure 24. Flow cytometry gating strategy for lung macrophages related to Figure 4l.

Supplementary Table 1. Demographics and clinical characteristics of COPD patients (Cohort 3) (Mean ± SEM).

	COPD	Control
**Subjects (n)**	16	11
**Mean age years**	57.06 ± 1.23	46 ± 4.93
**Sex**		
**Male**	7	10
**Female**	9	1
**Height (m)**	1.65 ± 0.02	1.75 ± 0.03
**Weight (kg)**	59.44 ± 3.18	80.45 ± 2.97
**Smoking (Pack-years)**	39.00 ± 7.55	
**FEV1 (%)**	34.38 ± 5.55	
**FVC (%)**	82.73 ± 5.84	
**FEV1/FVC (%)**	31.01 ± 5.07	

FEV1: Forced expiratory volume in the first second

FVC: Forced vital capacity

Supplementary Table 2. Patient characteristics of human study population (Cohort 4).

	Never smokers	Current smokers without COPD	Ex-smokers without COPD	Current smokers with COPD GOLD II	Ex-smokers with COPD GOLD II	Ex-smokers with COPD GOLD III-IV
**Number**	28	22	14	28	14	12
**Age (years)**	65 (53-71)	60 (55-68)	70 (57-73)	65 (57-69)	68 (65-72)	57 (54-60)$^{§¥}$
**Sex (male/female)**	14/14$^{\#}$	12/10$^{\#}$	8/6$^{\#}$	20/8$^{\#}$	13/1$^{\#}$	6/6$^{\#}$
**BMI**	26 (23-28)	24 (20-27)	28 (24-30)	23 (20-26)	28 (27-32)^{†δ}	21 (20-23)$^{*§¥}$
**Smoking**						
Current/ex-smoker	NA	22/0$^{\#}$	0/14$^{\#}$	28/0$^{\#}$	0/14$^{\#}$	0/12$^{\#}$
Pack-years	NA	38 (25-46)*	18 (12-24)*	45 (40-56)*§	41 (30-54)*	30 (24-30)*
**Lung function**						
FEV_1 post (L)	2.8 (2.3-3.3)	2.8 (2.3-3.1)	2.5 (2.0-3.4)	2.0 (1.6-2.2)*†	1.8 (1.5-2.0)*†	0.8 (0.7-1.0)*†§
FEV_1 post (% predicted)	97 (91-110)	96 (92-111)	94 (91-110)	67 (59-75)*†§	60 (51-66)*†§	26 (20-32)*†§
FEV_1/FVC post	78 (74-83)	76 (72-81)	75 (71-79)	55 (43-58)*†§	55 (46-58)*†§	33 (26-35)*†§
DL_{CO} (% predicted)	88 (80-103)	76 (57-88)	97 (79-105)	58 (47-81)*§	73 (55-83)	35 (33-42)$^{*†§¥}$
K_{CO} (% predicted)	96 (92-119)	84 (67-99)	106 (92-119)	68 (52-89)*§	106 (83-109)^{δ}	59 (52-65)$^{*§¥}$
**Medication**						
ICS (yes/no)	4/24$^{\#}$	2/20$^{\#}$	0/14$^{\#}$	13/15$^{\#}$	8/6$^{\#}$	11/1$^{\#}$
OCS (yes/no)	0/28$^{\#}$	0/22$^{\#}$	2/12$^{\#}$	5/23$^{\#}$	4/10$^{\#}$	2/10$^{\#}$
SABA (yes/no)	0/28$^{\#}$	2/20$^{\#}$	0/14$^{\#}$	3/25$^{\#}$	6/8$^{\#}$	11/1$^{\#}$
LABA (yes/no)	2/26$^{\#}$	2/20$^{\#}$	0/14$^{\#}$	12/16$^{\#}$	9/5$^{\#}$	11/1$^{\#}$
SAMA (yes/no)	0/28$^{\#}$	2/20$^{\#}$	0/14$^{\#}$	3/25$^{\#}$	6/8$^{\#}$	9/3$^{\#}$
LAMA (yes/no)	0/28$^{\#}$	2/20$^{\#}$	0/14$^{\#}$	10/18$^{\#}$	7/7$^{\#}$	12/0$^{\#}$
Statins (yes/no)	4/24	7/15	1/13	7/21	5/9	3/9

FEV_1 (forced expiratory volume in 1 second); FVC (forced vital capacity); DL_{CO} (diffusing capacity of the lungs for carbon monoxide); ICS (inhaled corticosteroids); OCS (oral corticosteroids); SABA (short acting β2-agonists); LABA (long acting β2-agonists); SAMA (short acting muscarinic antagonists); LAMA (long acting muscarinic antagonists); NA (not applicable). Data are presented as median (IQR). Kruskal-Wallis with Dunn's multiple comparisons test (two-sided): * $P < 0.05$ versus never smokers, † $P < 0.05$ versus current smokers without COPD, § $P < 0.05$ versus ex-smokers without COPD, ^{δ} $P < 0.05$ versus current smokers with COPD GOLD II, $^{¥}$ $P < 0.05$ versus ex-smokers with COPD GOLD II. Fisher's exact test (two-sided): $^{\#}$ $P<0.001$.

Supplementary Table 3. Clinical characteristics and demographics of blood donors (Mean ± SEM).

	NON-SMOKERS	SMOKERS
**SUBJECTS**	10	11
**AGE**	28.8±3.30	31.55±1.77
**GENDER**	7F/3M	6F/5M
**SMOKING (PACKS/ YEAR)**	-	7.92±2.37
**%FEV_1/FVC**	-	-
**%FEV_1**	-	-
**%FVC**	-	-

Supplementary Table 4. Primer sequences used for the quantitative real time RT-PCR.

Gene	Forward primer	Reverse Primer
CARM1	ACA GCG TCC TCA ATC CAG TTC	GCT GGG ACA GGT AGG CAT AA
CCL2	TTC CCC TAG CTT TCC CCA GA	TCC CAG GGG TAG AAC TGT GG
CXCL1	CTT CCT CCT CCC TTC TGG TC	CCA AAC CGA AGT CAT AGC CA
EZH2	ATT GCT GGC ACC ATC TGA CG	TGC ATC CAC CAC AAA ATC ATT G
GNMT	CTG GGG TGG ACT CCA TTA TGC	GAT GAC CCA CTT GTC GAA GGC
HPRT1	AGG AAA GCA AAG TCT GCA TTG TT	GGT GGA GAT GAT CTC TCA ACT TTA A
IL6	CCT GAA CCT TCC AAA GAT GGC	TTC ACC AGG CAA GTC TCC TCA
IL8	GCA GAG CAC ACA AGC TTC TAG GA	CCA GCT TGG AAG TCA TGT TTA CAC
PNMT	CTC ATT GAG GGC AAG GGG GAA T	GAT GGG CAG GAC CCG TTT C
PRMT1	GCT GAG GAC ATG ACA TCC AA	GAA GAG GTG CCG GTT ATG AA
PRMT5	GGC CAT CAC TCT TCC ATG TT	CCA CAT CCA CGT TTT CTC CT
PRMT6	TCT GGT TCC AGG TGA CCT TC	GTC CGT GTC TTG CTC CAC TT
PRMT7	GCT GCT GTG AAG ATT GTG GA	CCG ATC AGC TCT GTG TCA AA
SETD4	GGA GAA CAA GCC GGA TCA GAA	AGC AGG CGC TAA GTT TGA ATC

TNF	GCC TCT TCT CCT TCC TGA TCG	AGC TTG AGG GTT TGC TAC AAC A
Acta2	TCC CTG GAG AAG AGC TAC GAA CT	GAT GCC CGC TGA CTC CAT
Arg1	GGA ACC CAG AGA GAG CAT GA	TTT TTC CAG CAG ACC AGC TT
Carm1	GTG GGC AGA CAG TCC TTC AT	GTC CGC TCA CTG AAC ACA GA
Ccl2	CTT CTG GGC CTG CTG TTC A	CCA GCC TAC TCA TTG GGA TCA
Cd86	CTG GAC TCT ACG ACT TCA CAA TG	AGT TGG CGA TCA CTG ACA GTT
Cxcl1	GCT GGG ATT CAC CTC AAG AA	TCT CCG TTA CTT GGG GAC AC
Cxcl12	TGC ATC AGT GAC GGT AAA CCA	TTC TTC AGC CGT GCA ACA ATC
Fizz1	TGC CAA TCC AGC TAA CTA TCC C	ACG AGT AAG CAC AGG CAG TT
Hprt1	AGC TAC TGT AAT GAT CAG TCA ACG	AGA GGT CCT TTT CAC CAG CA
Icam1	ACC CAA CTG GTG TTT G	CAC ACT CTC CAC GAA T
Il10	CCA AGC CTT ATC GGA AAT CA	TCA CTC TTC ACC TGC TCC
Il12p35	ACT AGA GAG ACT TCT TCC ACA ACA AGA G	GCA CAG GGT CAT CAT CAA AGA C
Il1b	AGT TGA CGG ACC CCA AAA GAT	GGA CAG CCC AGG TCA AAG G
Il6	GTT CTC TGG GAA ATC GTG GA	TGT ACT CCA GGT AGC TAT GG
Inos	CGG CAA ACA TGA CTT CAG GC	GCA CAT CAA AGC GGC CAT AG
Itga4	TCC AAA CCA GAC CTG CGA AC	TGT GCC CAC AAG TCA CGA TAG

Itgal	CCA GAC TTT TGC TAC TGG GAC	GCT TGT TCG GCA GTG ATA GAG
Itgam	GTT TGT TGA AGG CAT TTC CC	ATT CGG TGA TCC CTT GGA TT
Mmp13	CTT TGG CTT AGA GGT GAC TGG	AGG CAC TCC ACA TCT TGG TTT
Prkca	AAC GAA CTC ATG GCA CCT CT	CAC TGC ACC GAC TTC ATC TG
Prmt1	CGA ACT GCA TCA TGG AGA AT	AGC GTT GGG CTT CTC CAC TAC
Prmt5	TGA CCA ACC ACA TCC ACA CT	GTG TGT AGT CGG GGC ATT CT
Prmt6	AAA CCT CTG GTG CTG TCC AC	TTT CCT CAT GGT CTC CCA CT
Prmt7	TAC TGC AGG GGC TGA CTT CT	TCA CCT CAG TGG AGT GCT TG
Rap1a	ATGCGTGAGTACAAGCTAGTAGT	AATCTACCTCGACTTGCTTTCTG
Rap1b	GCT CGT CGT GCT TGG ATC AG	ATT GCT CCG TTC CTG CAG TGT
Timp3	CAA CTC CGA CAT CGT GAT CC	CAC GTG GGG CAT CTT ACT GA
Tnf	CAC CAC GCT CTT CTG TCT	GGC TAC AGG CTT GTC ACT C
Vcam1	CAA TGG GGT TGG AAT G	CAC CTG GGT TTT TCC A

Supplementary Table 5. Primer sequences used for the ChIP-qPCR.

Gene	Forward primer	Reverse Primer
Rap1a (I)	AAA GTT CCG GTG CAG AGA CA	ACC GTG CTA GGA CTT GTT GC
Rap1a (II)	GGT GGT TAA GGC CAA CAT TGA	ACT AAC AGC TGT CCC ATG GTT G

The red box indicates cropped area used in the figure

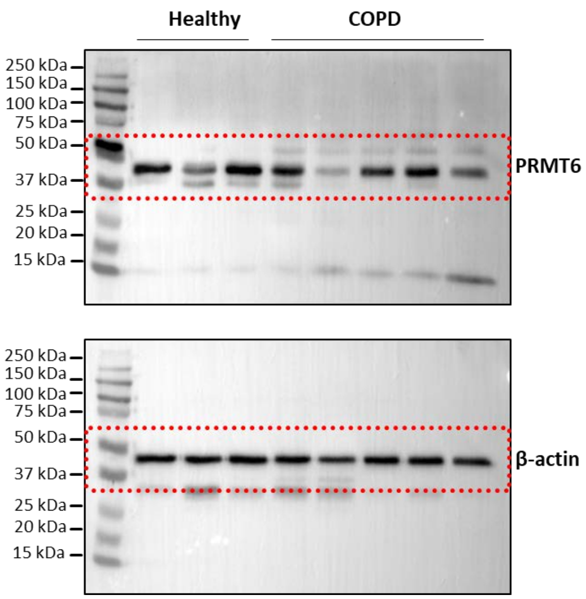

Blue box indicates repeats used in densitometry

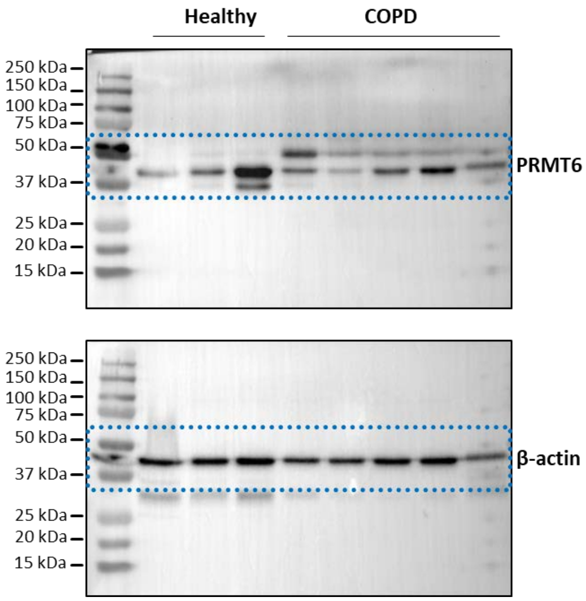

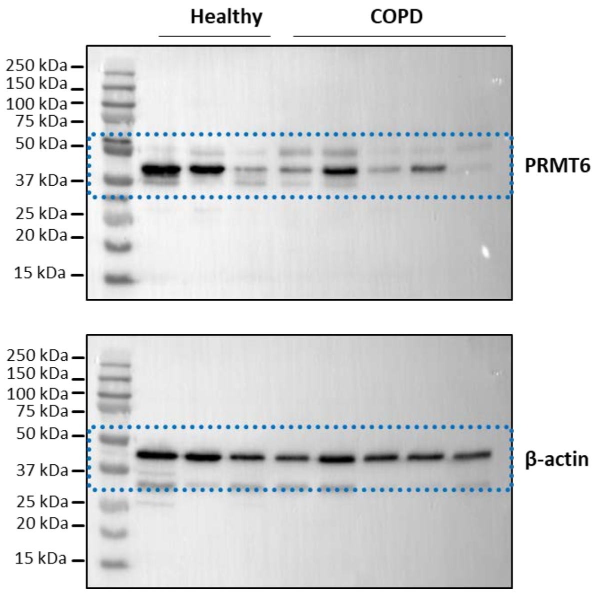

Supplementary Figure 25. Uncropped Blots from Supp. Fig 1k

The red box indicates cropped area used in the figure

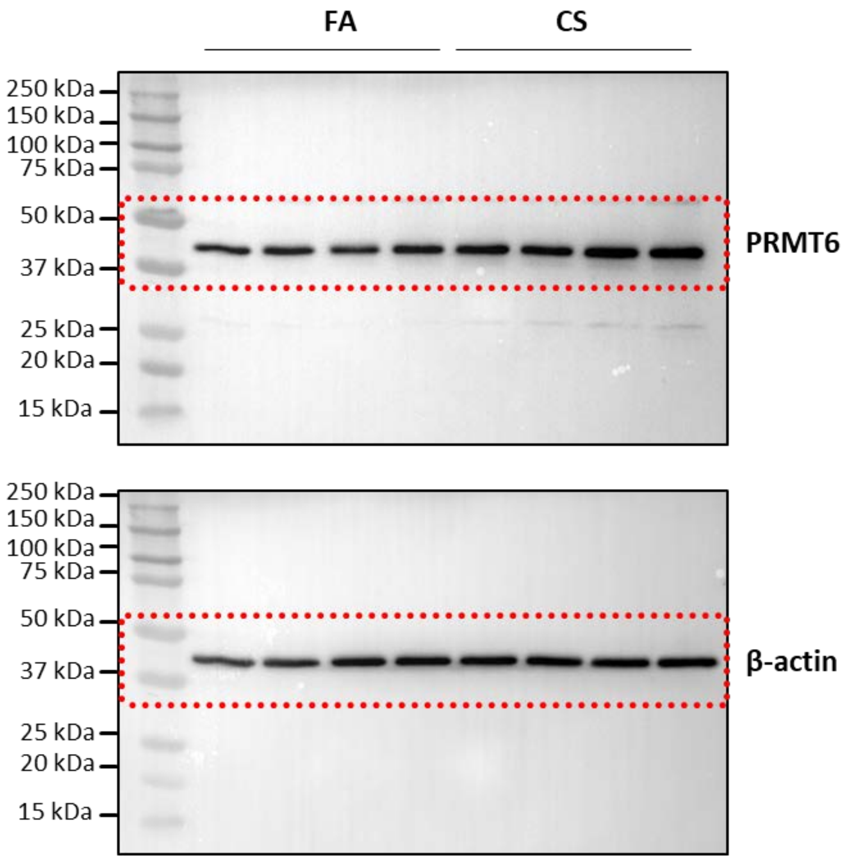

Supplementary Figure 26. Uncropped Blots from Supp. Fig 1l

The red box indicates cropped area used in the figure

Blue box indicates repeats used in densitometry

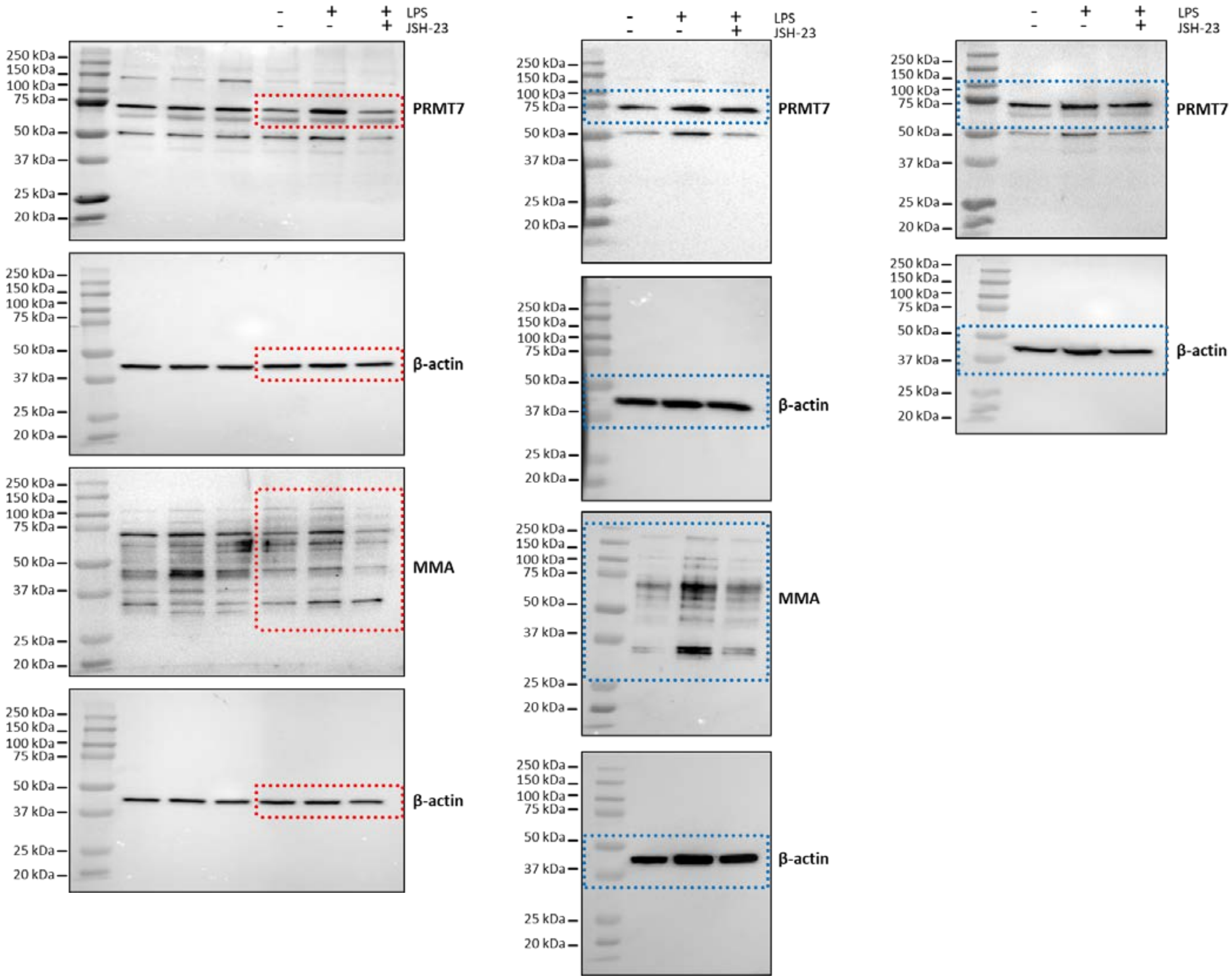

Supplementary Figure 27. Uncropped Blots Supp. Fig 4e

The red box indicates cropped area used in the figure

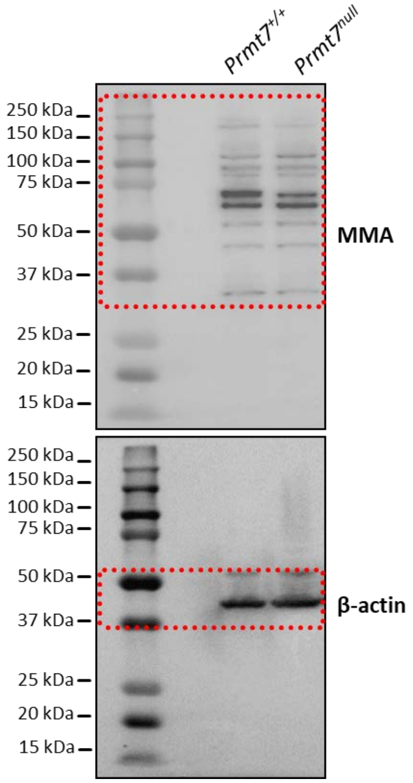

Blue box indicates repeats used in densitometry

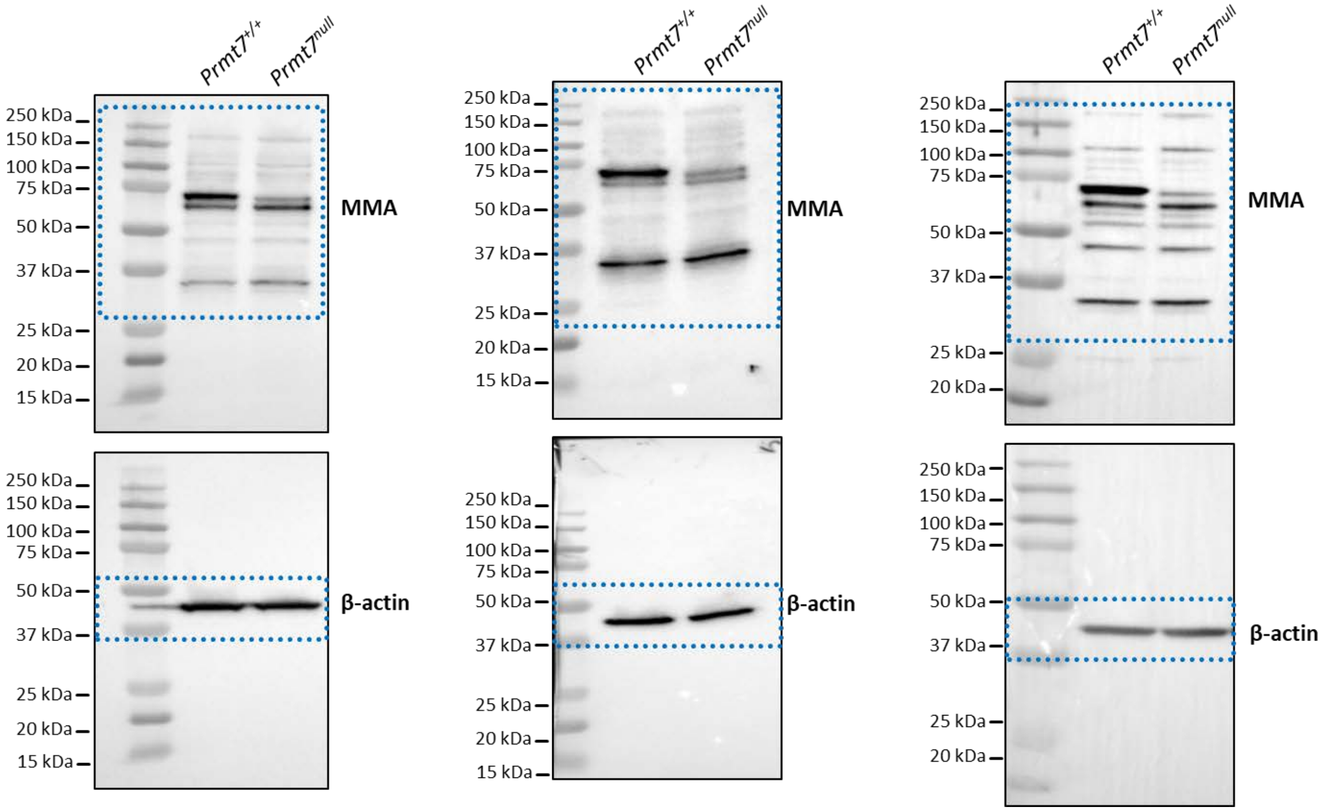

Supplementary Figure 28. Uncropped Blots Supp. Fig 5d

The red box indicates cropped area used in the figure

Blue box indicates repeats used in densitometry

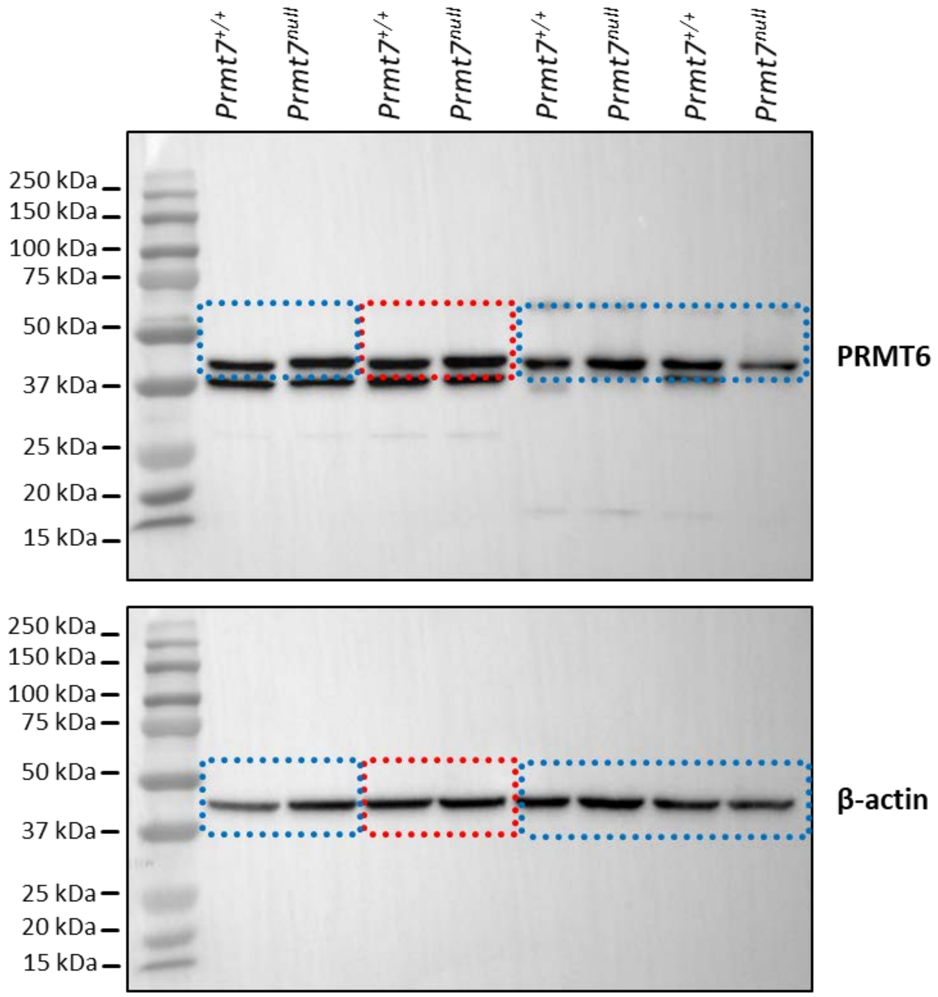

Supplementary Figure 29. Uncropped Blots from Supp. Fig 5f

The red box indicates cropped area used in the figure

Blue box indicates repeats used in densitometry

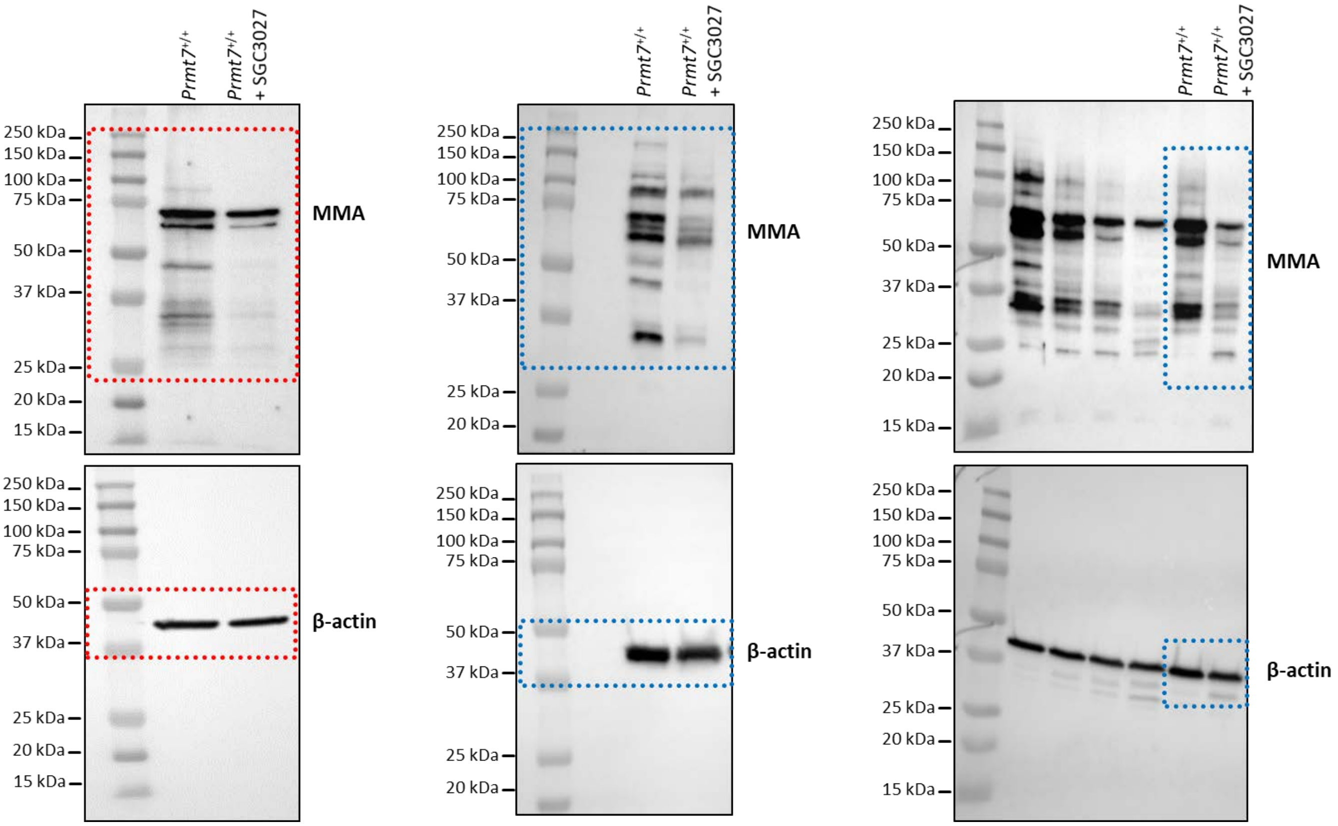

Supplementary Figure 30. Uncropped Blots from Supp. Fig 5g

The red box indicates cropped area used in the figure

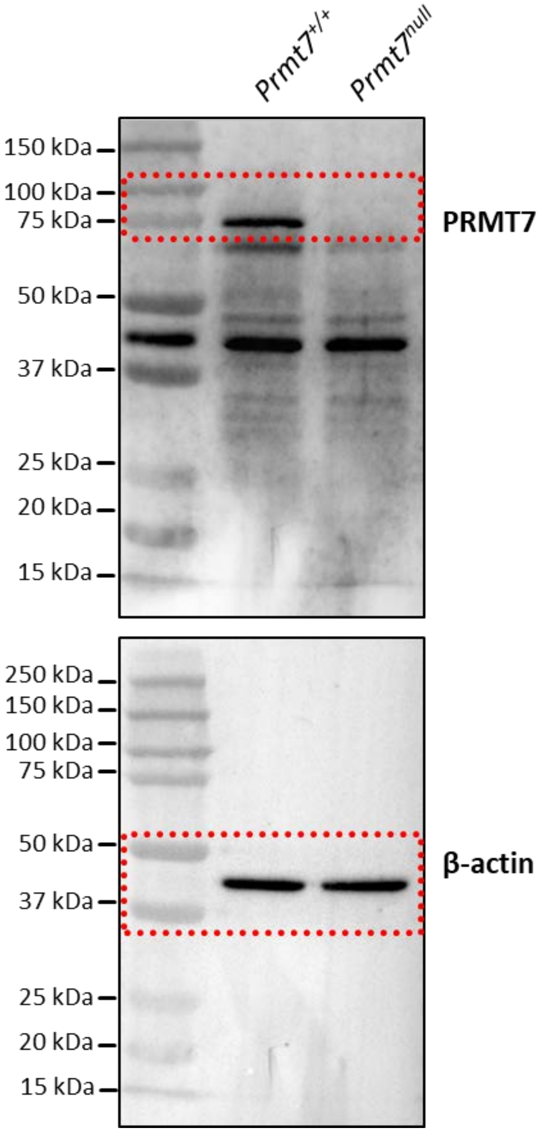

Supplementary Figure 31. Uncropped Blots from Supp. Fig 5i

The red box indicates cropped area used in the figure

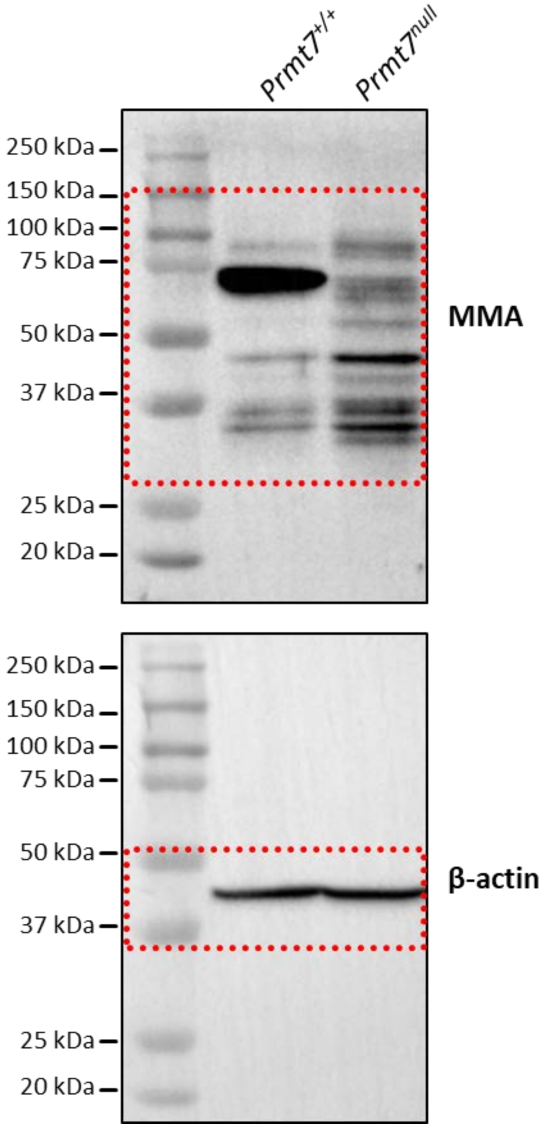

Supplementary Figure 32. Uncropped Blots from Supp. Fig 5j

The red box indicates cropped area used in the figure

Blue box indicates repeats used in densitometry

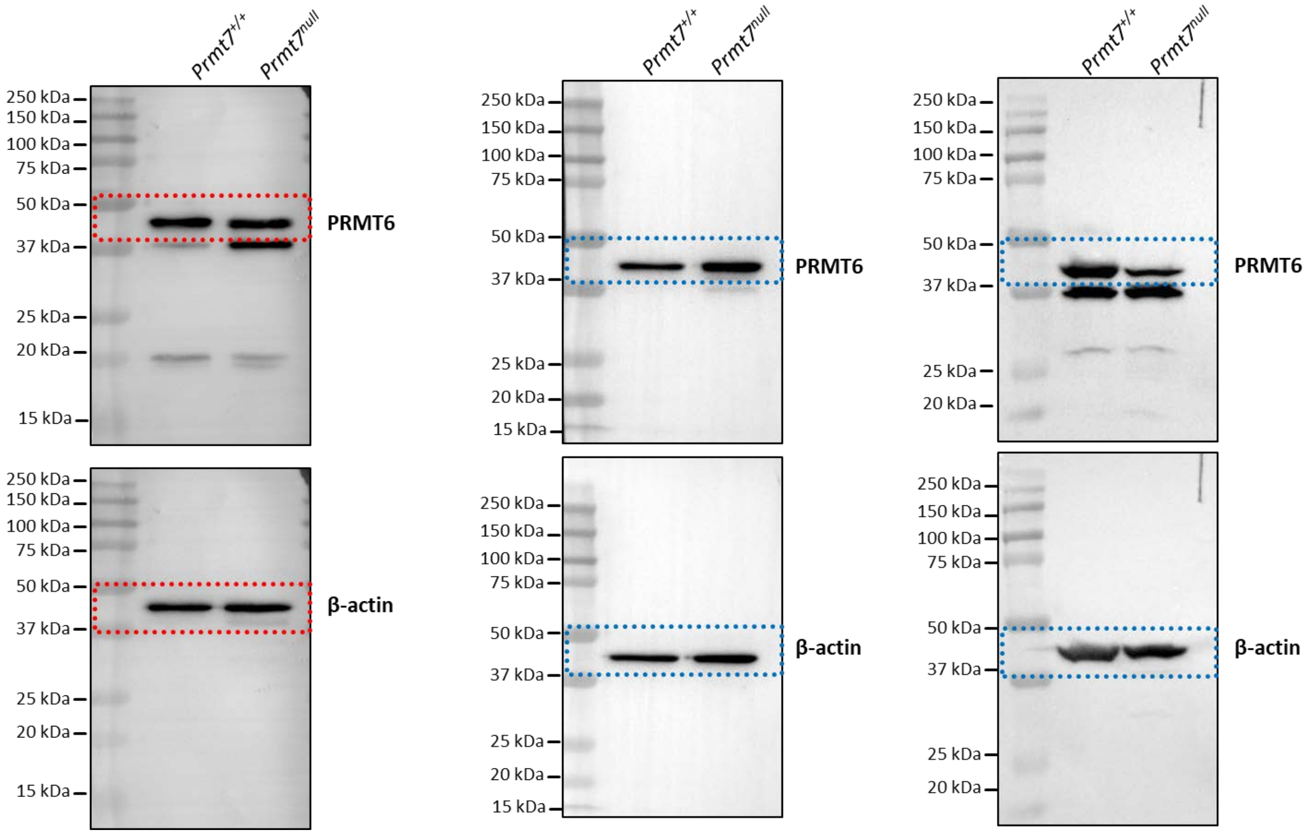

Supplementary Figure 33. Uncropped Blots from Supp. Fig 5m

The red box indicates cropped area used in the figure

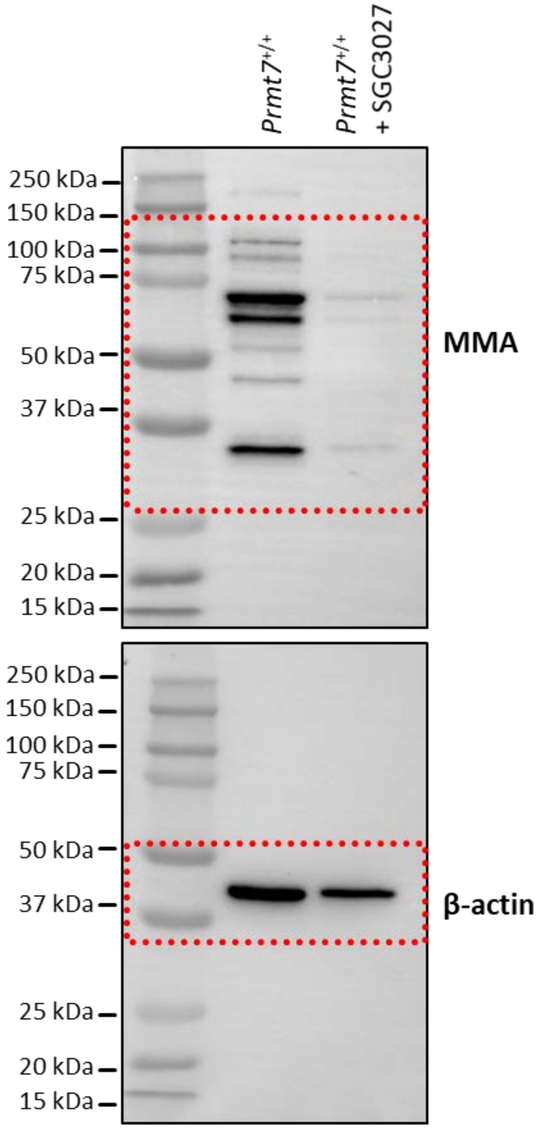

Supplementary Figure 34. Uncropped Blots from Supp. Fig 7b

The red box indicates cropped area used in the figure

Blue box indicates repeats used in densitometry

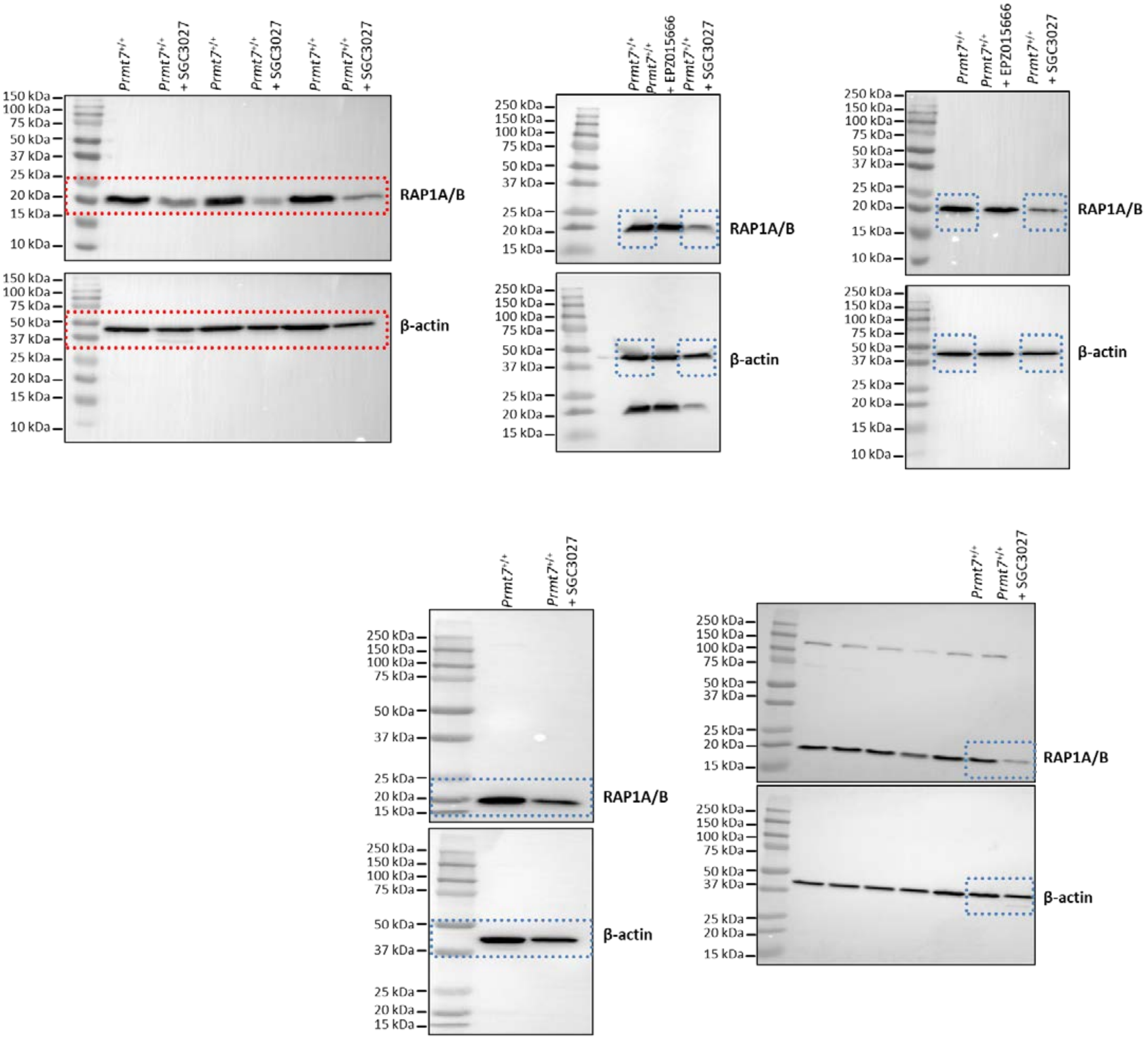

Supplementary Figure 35. Uncropped Blots from Supp. Fig 7d

The red box indicates cropped area used in the figure

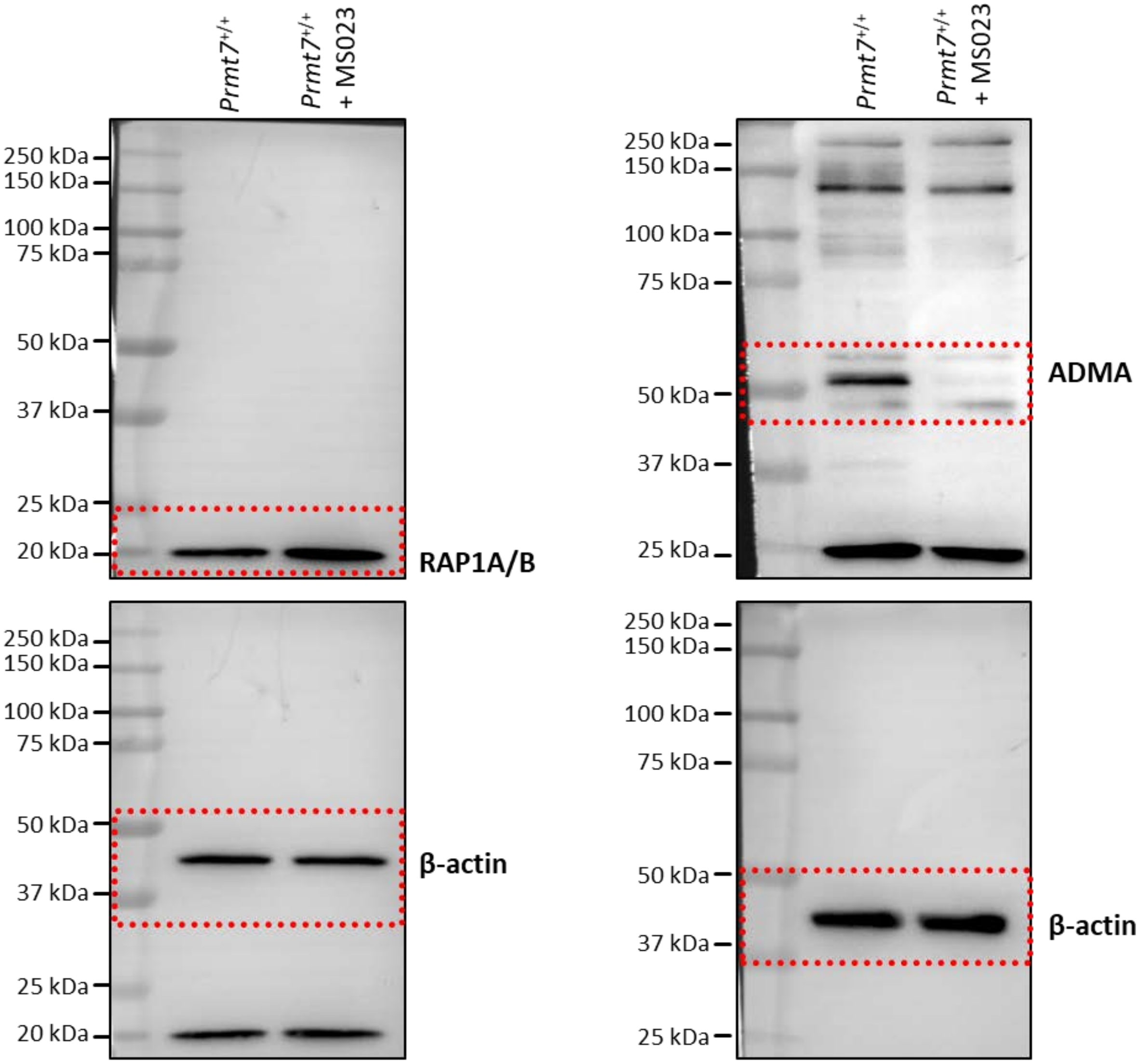

Supplementary Figure 36. Uncropped Blots from Supp. Fig 7g

The red box indicates cropped area used in the figure

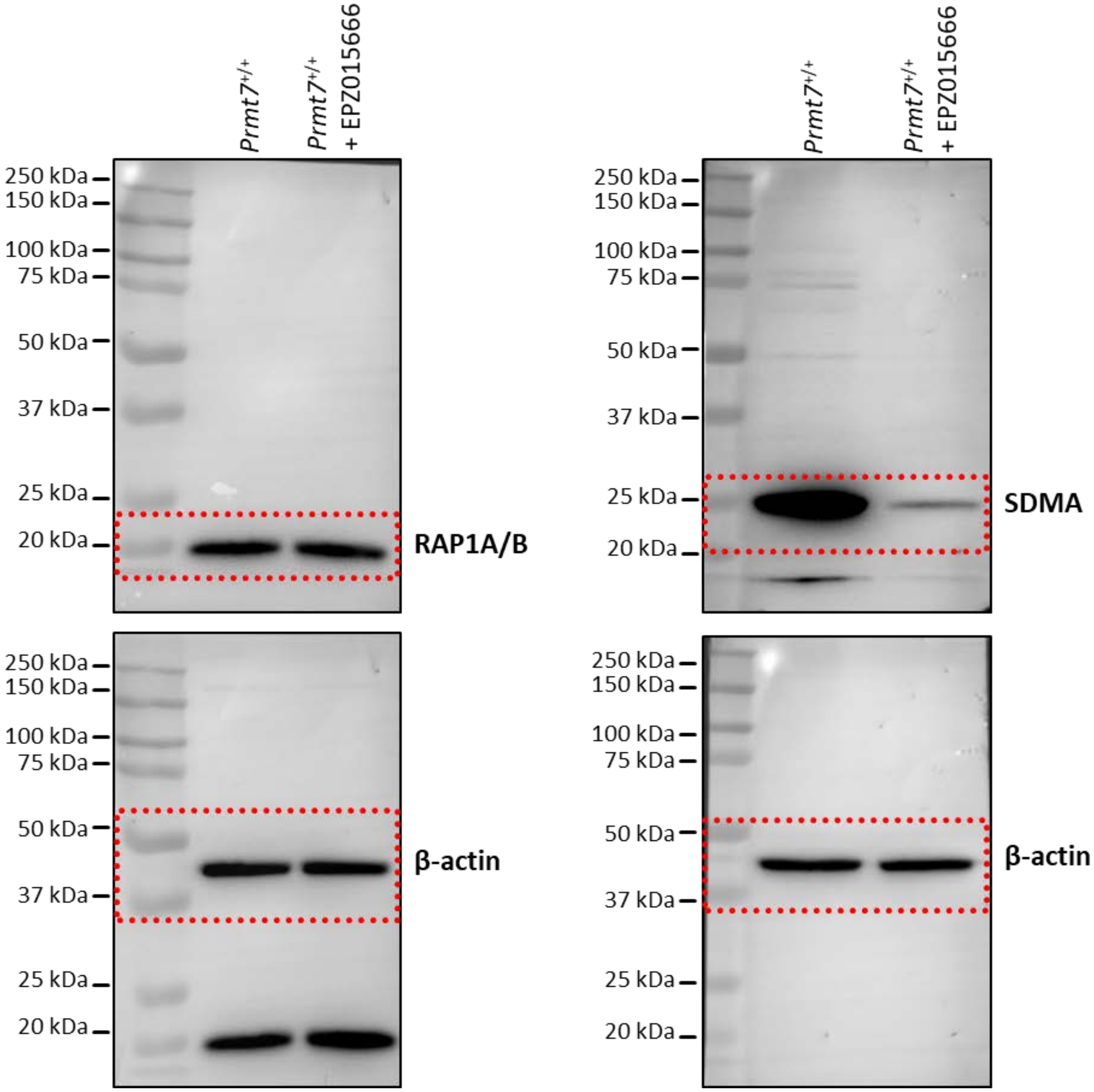

Supplementary Figure 37. Uncropped Blots from Supp. Fig 7h

The red box indicates cropped area used in the figure

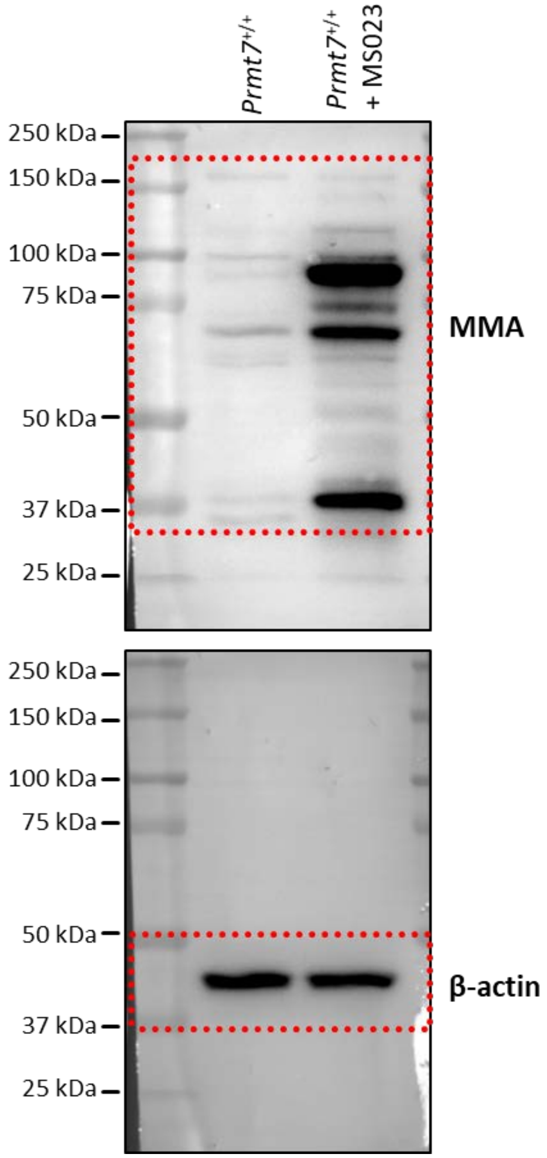

Supplementary Figure 38. Uncropped Blots from Supp. Fig 7i

The red box indicates cropped area used in the figure

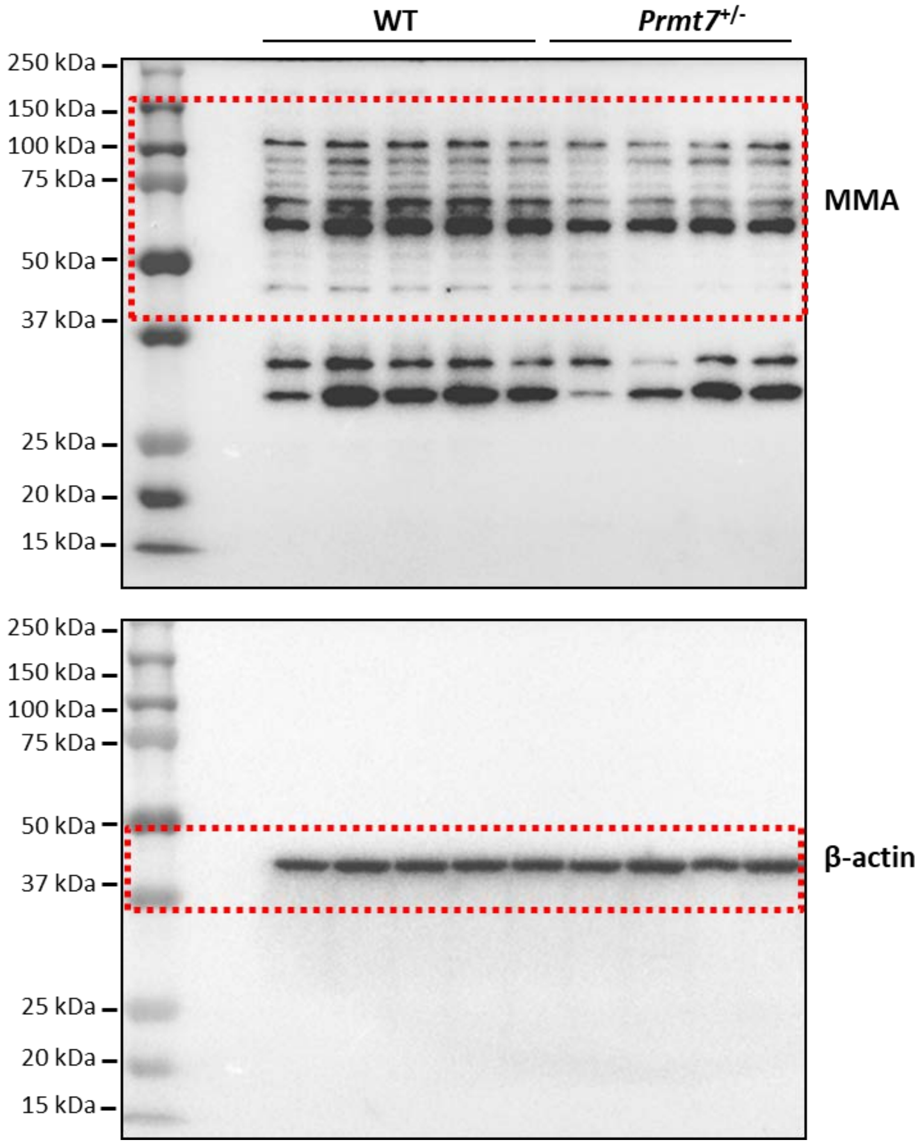

Supplementary Figure 39. Uncropped Blots from Supp. Fig 8d

The red box indicates cropped area used in the figure

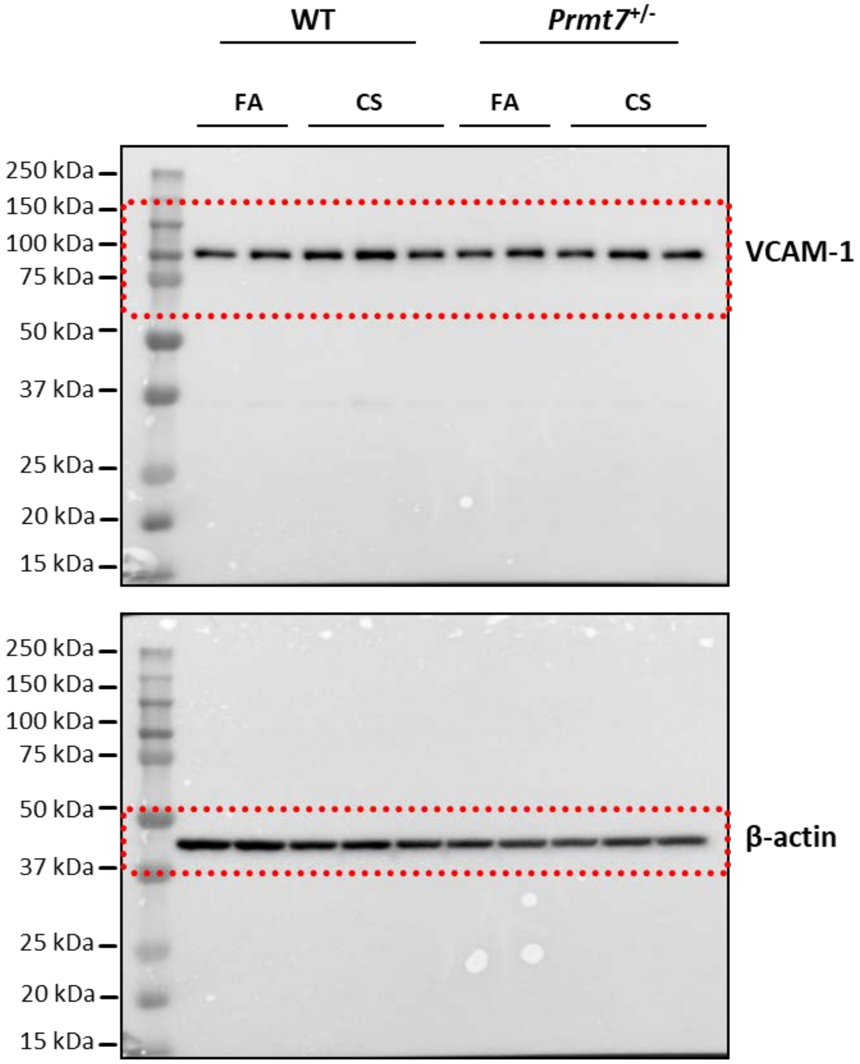

Supplementary Figure 40. Uncropped Blots from Supp. Fig 11b

The red box indicates cropped area used in the figure

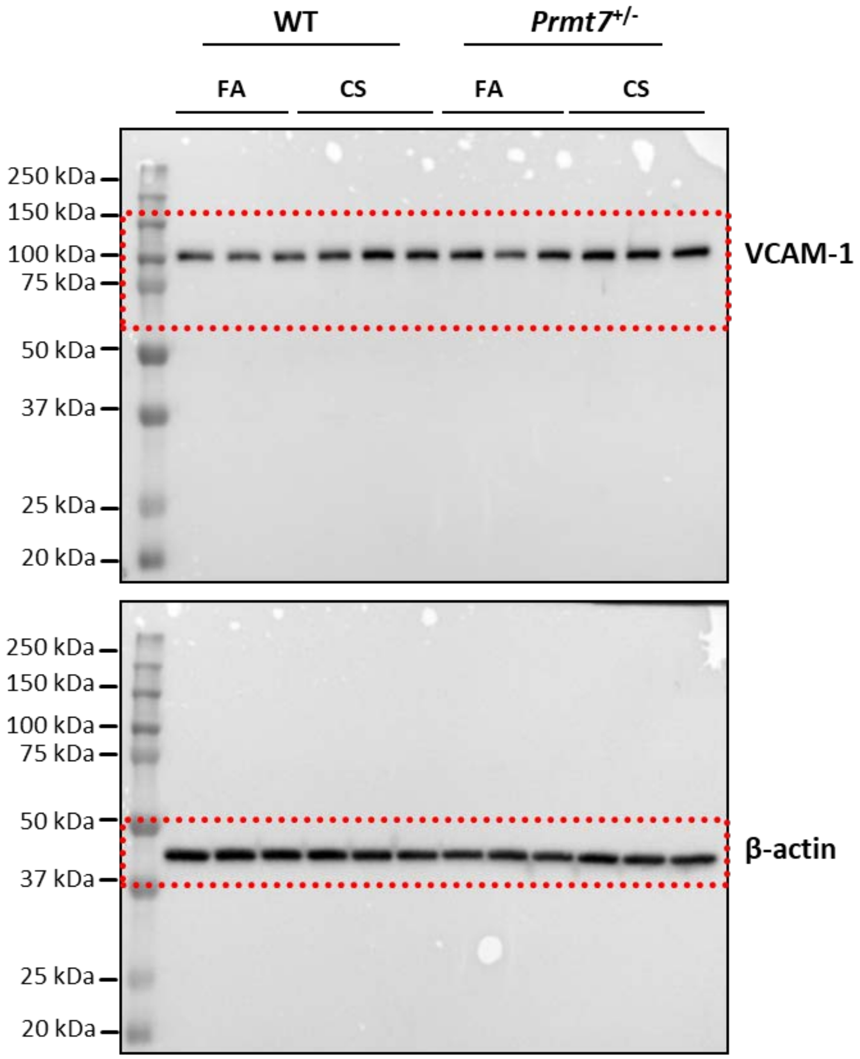

Supplementary Figure 41. Uncropped Blots from Supp. Fig 11d

The red box indicates cropped area used in the figure

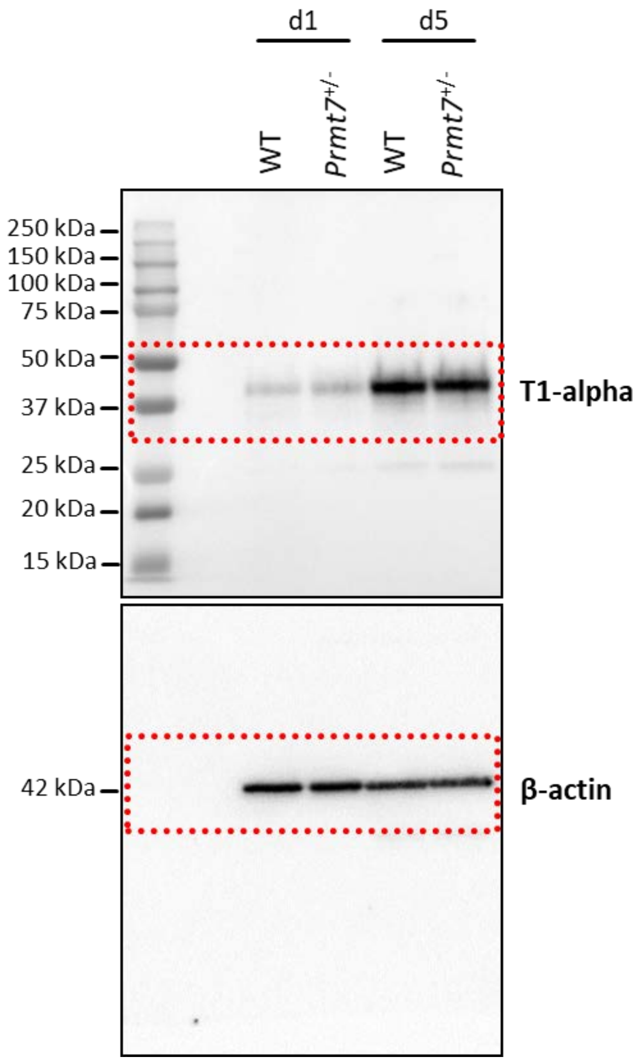

Supplementary Figure 42. Uncropped Blots from Supp. Fig 16f

The red box indicates cropped area used in the figure

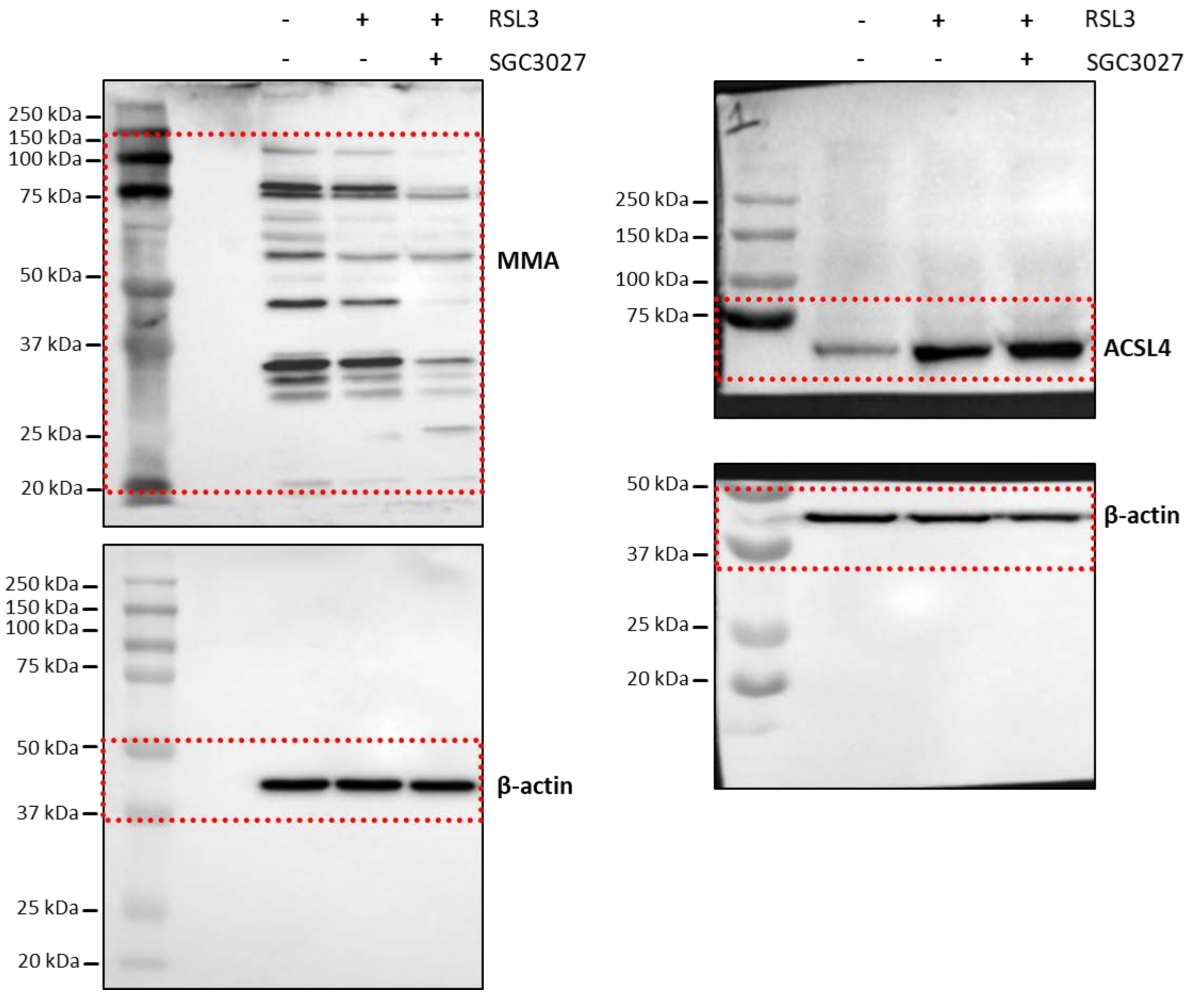

Supplementary Figure 43. Uncropped Blots Supp. Fig 18g

The red box indicates cropped area used in the figure

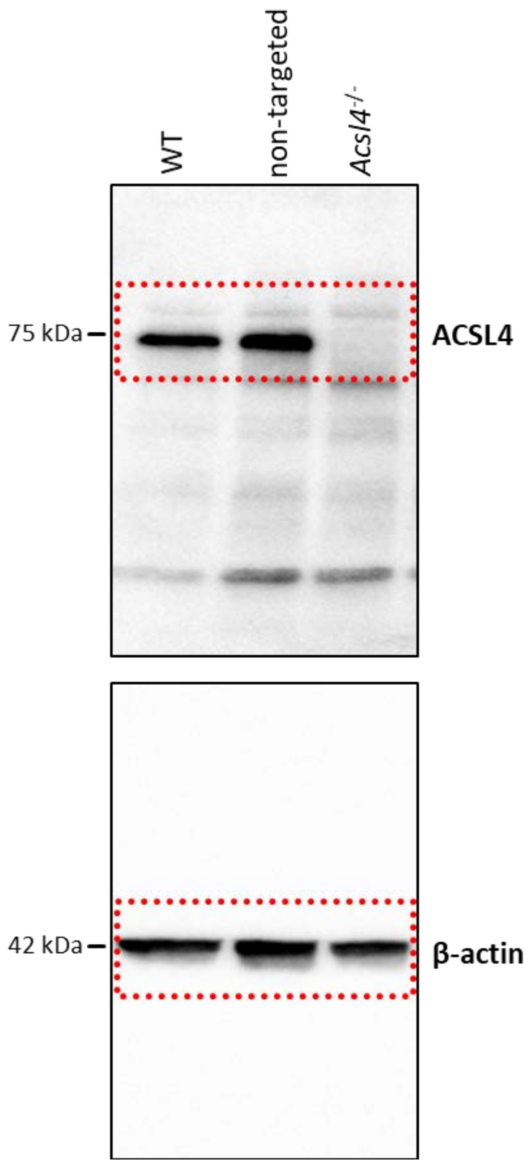

Supplementary Figure 44. Uncropped Blots from Supp. Fig 18h

The red box indicates cropped area used in the figure

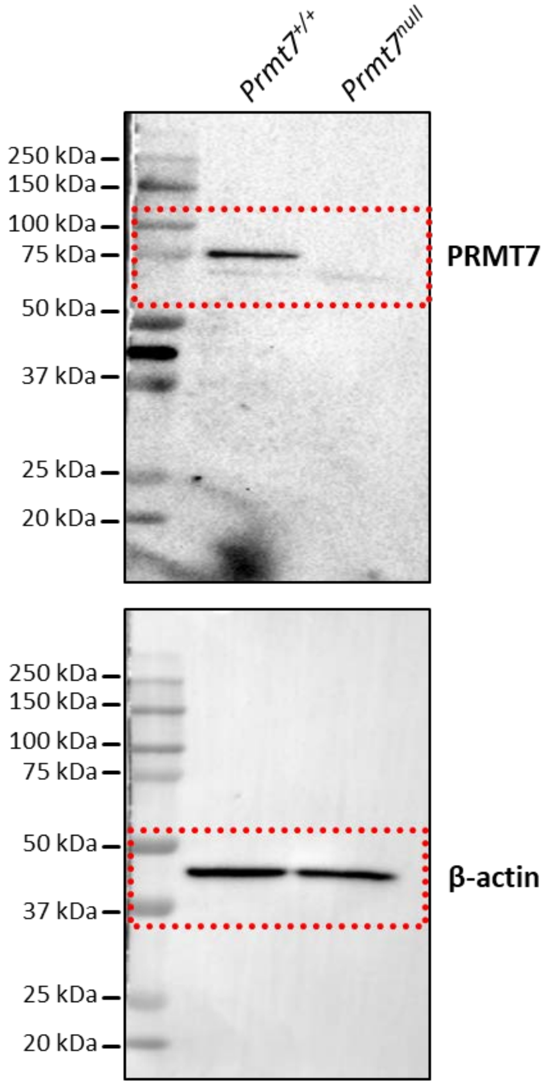

Supplementary Figure 45. Uncropped Blots from Supp. Fig 20f

The red box indicates cropped area used in the figure

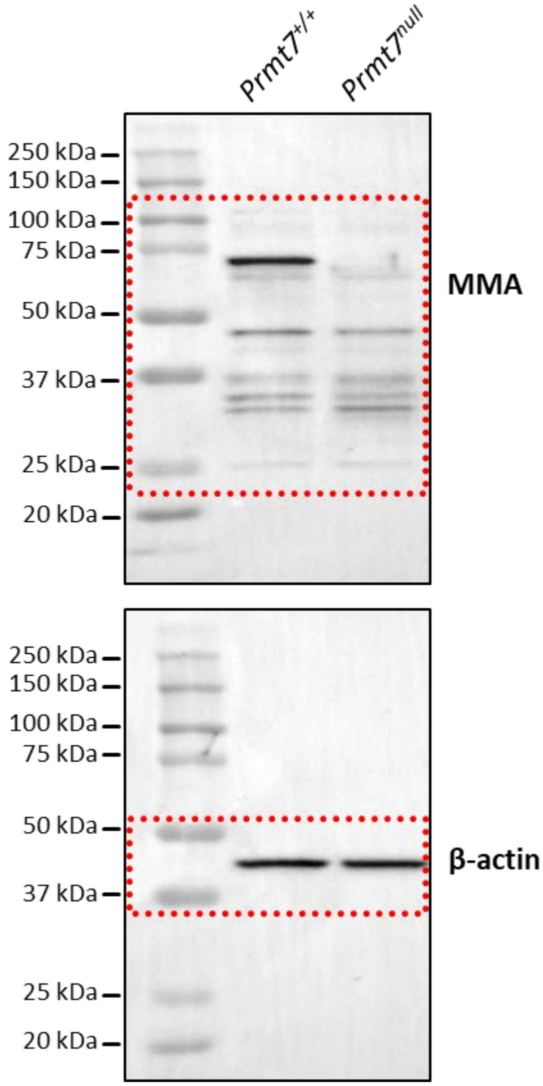

Supplementary Figure 46. Uncropped Blots from Supp. Fig 20g

The red box indicates cropped area used in the figure

Blue box indicates repeats used in densitometry

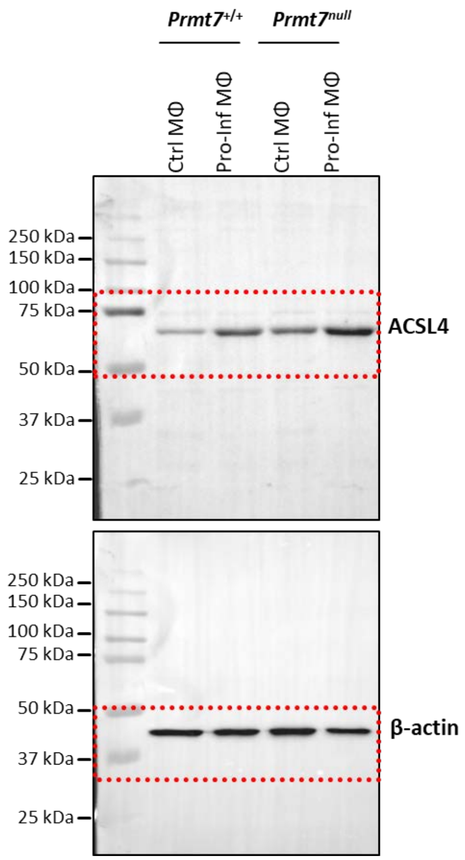

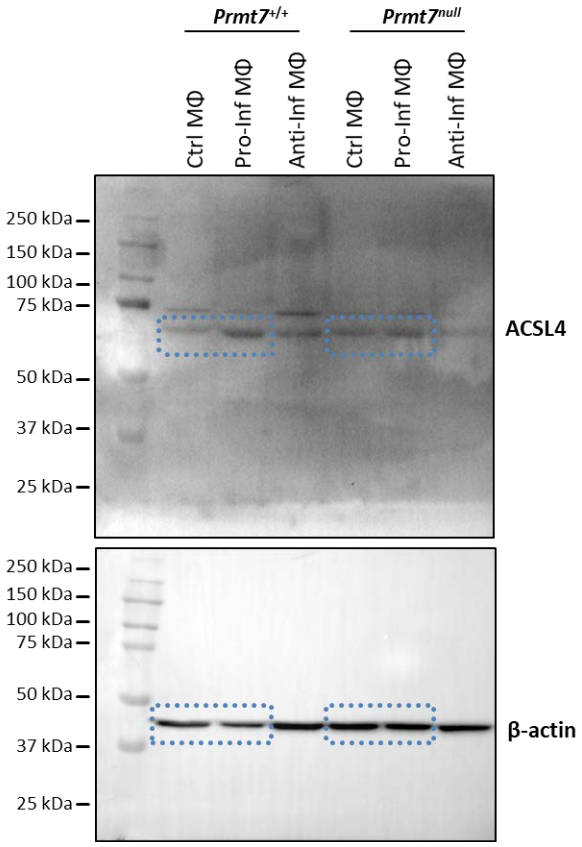

Supplementary Figure 47. Uncropped Blots from Supp. Fig 20i

The red box indicates cropped area used in the figure

Blue box indicates repeats used in densitometry

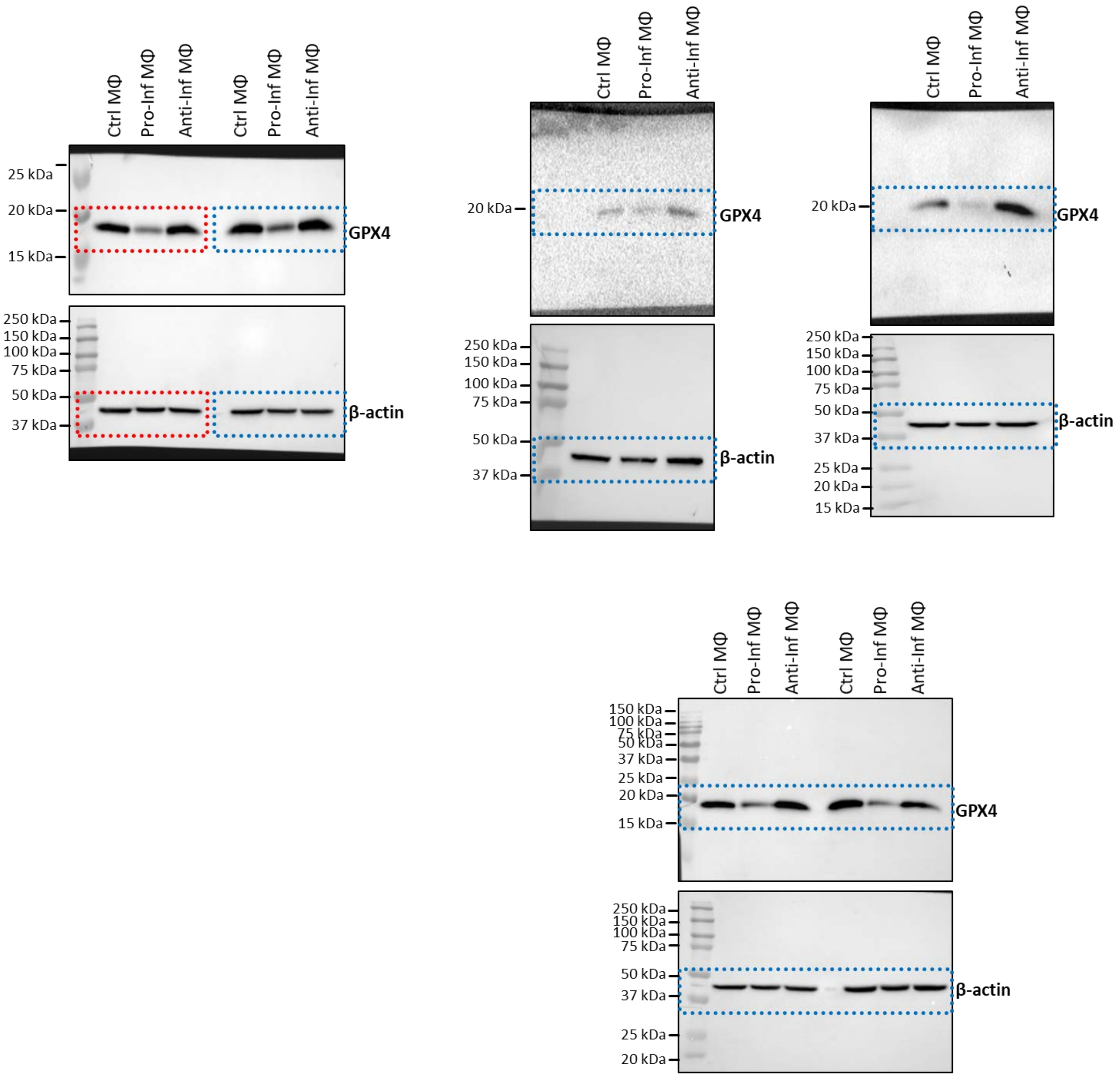

Supplementary Figure 48. Uncropped Blots from Supp. Fig 21c

The red box indicates cropped area used in the figure

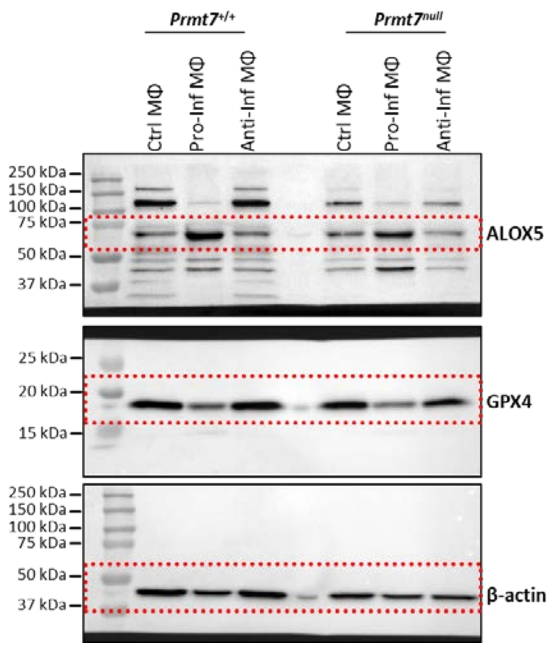

Blue box indicates repeats used in densitometry

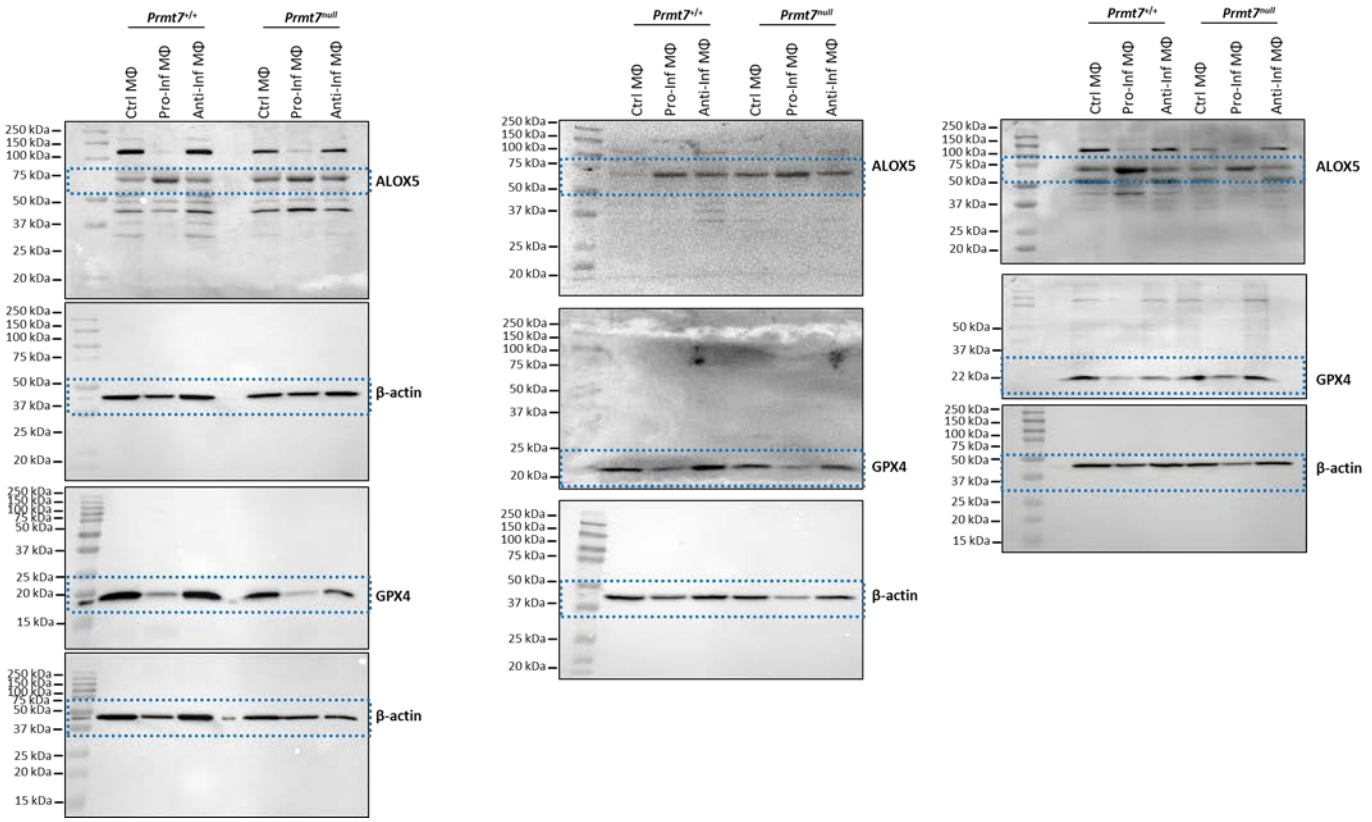

Supplementary Figure 49. Uncropped Blots from Supp. Fig 22a

The red box indicates cropped area used in the figure

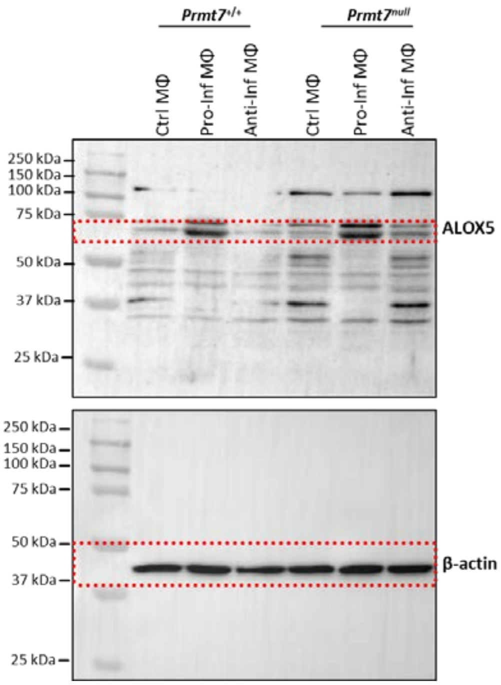

Blue box indicates repeats used in densitometry

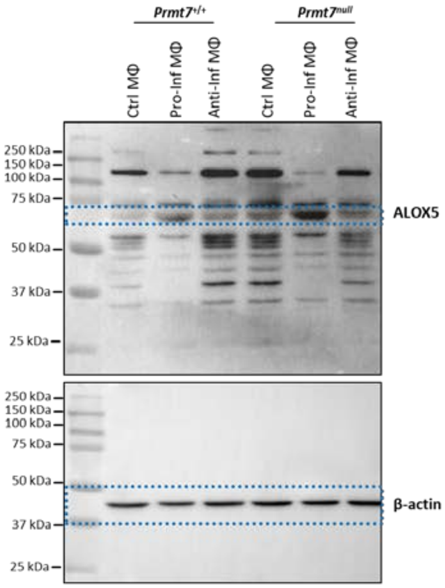

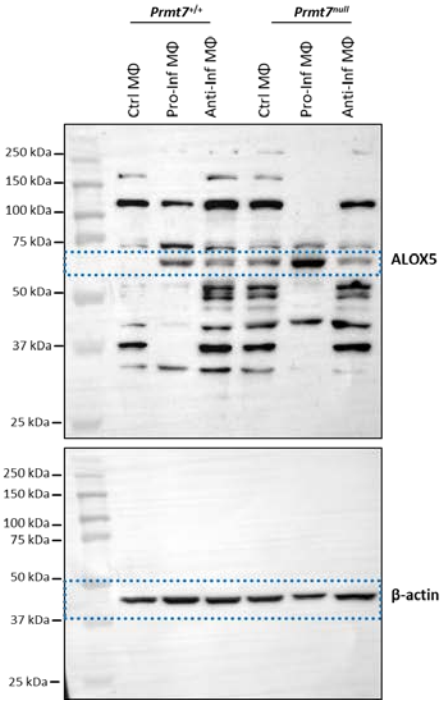

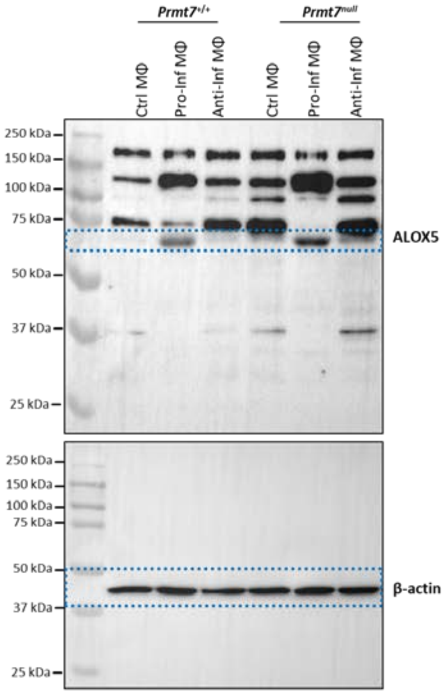

Supplementary Figure 50. Uncropped Blots from Supp. Fig 22a

The red box indicates cropped area used in the figure

Blue box indicates repeats used in densitometry

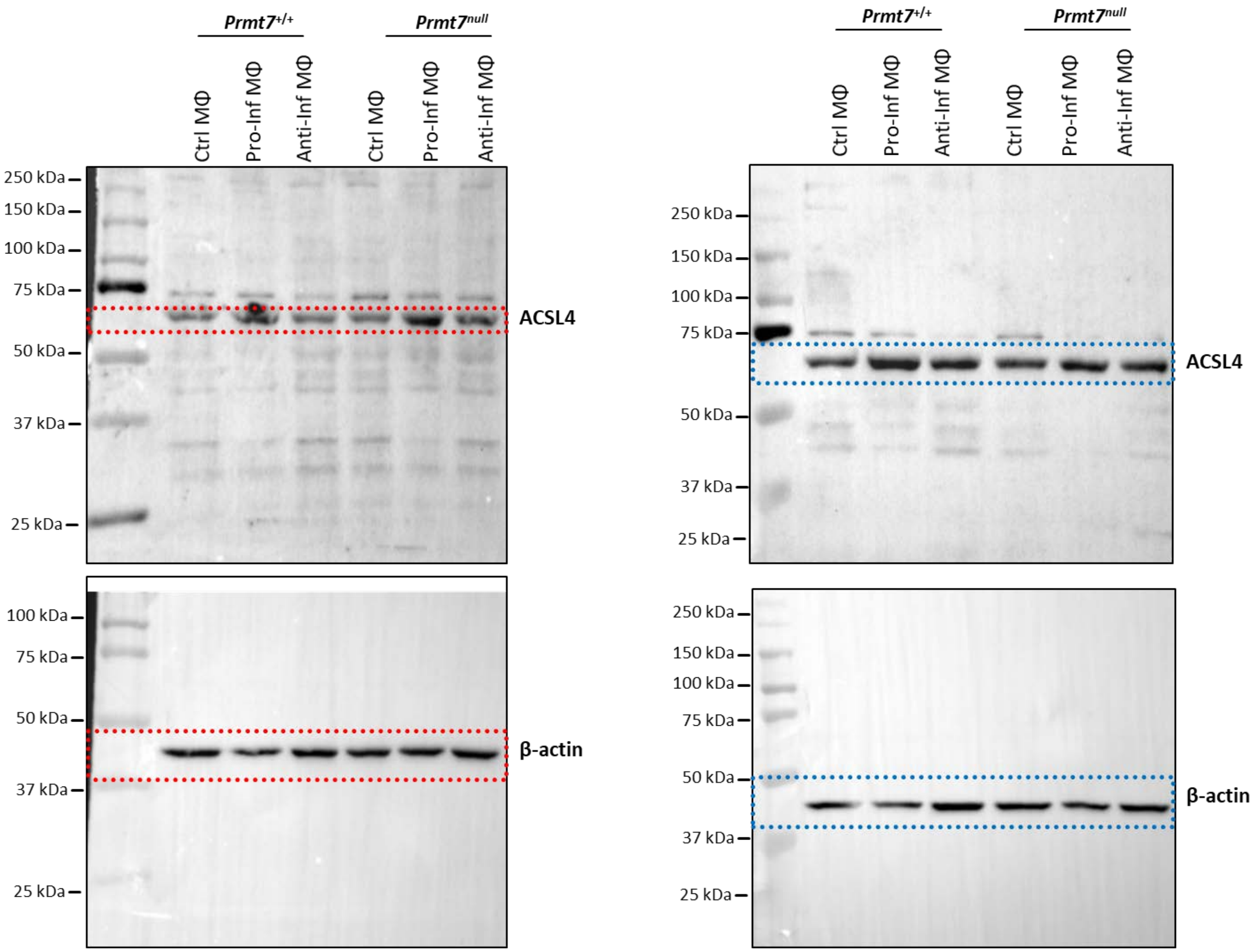

Supplementary Figure 51. Uncropped Blots from Supp. Fig 22d

The red box indicates cropped area used in the figure

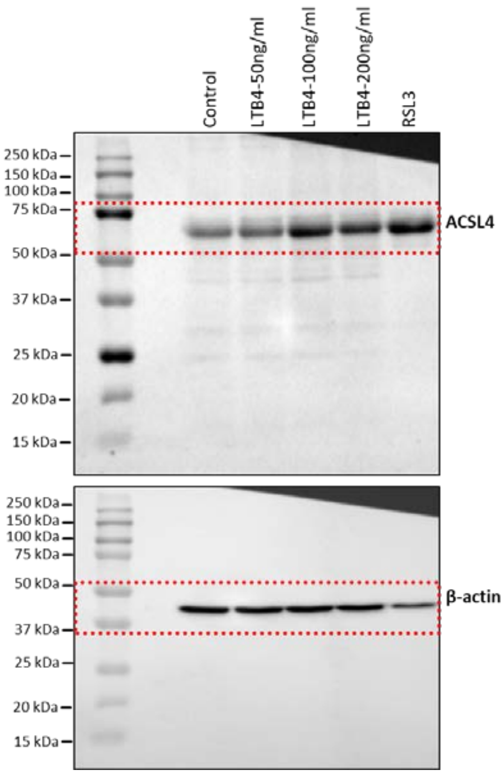

Blue box indicates repeats used in densitometry

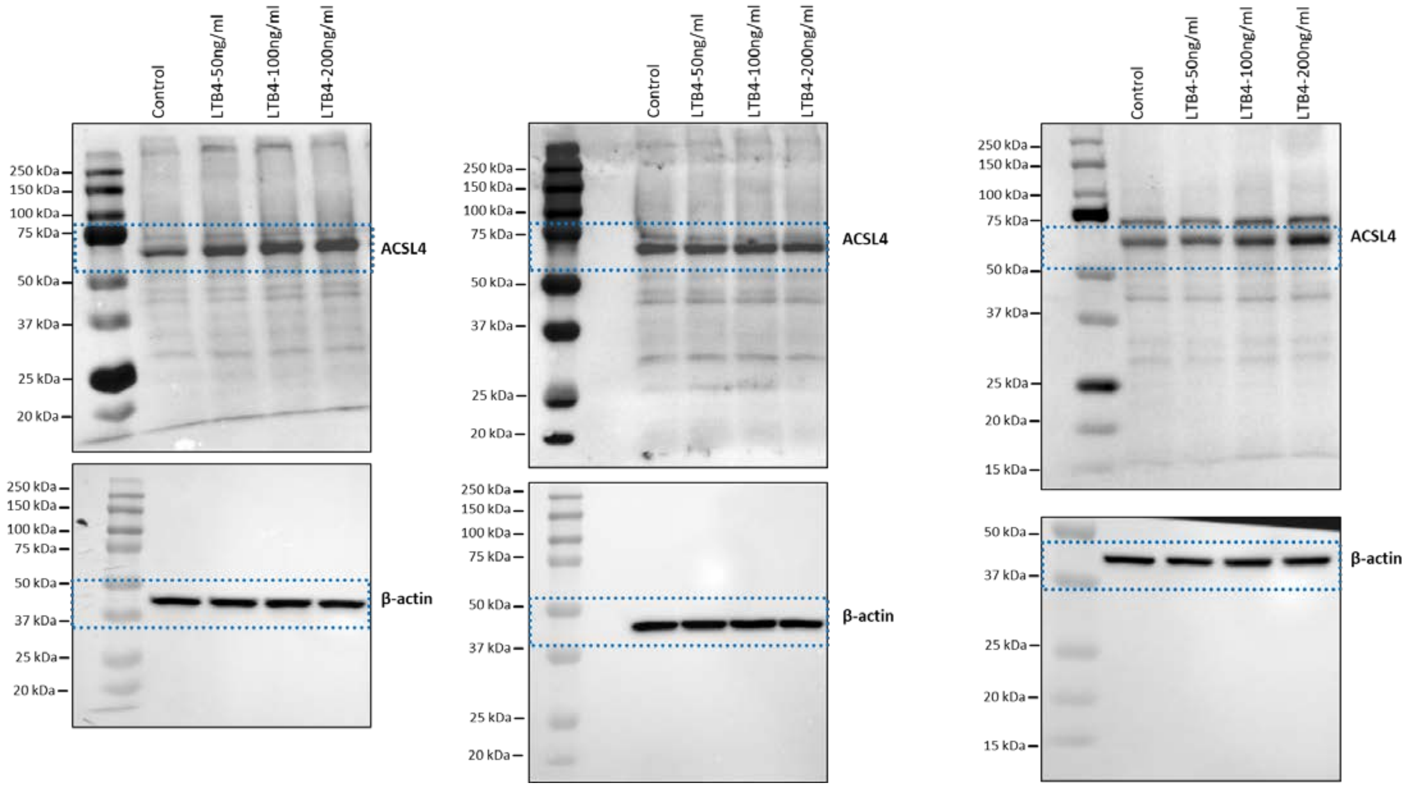

Supplementary Figure 52. Uncropped Blots from Supp. Fig 22i
