# Supplementary material for: The arginine methyltransferase PRMT7 promotes extravasation of monocytes resulting in tissue injury in COPD
Source: Nat Commun. 2022 Mar 14;13:1303. doi: 10.1038/s41467-022-28809-4 (PMC8921220; doi:10.1038/s41467-022-28809-4)
Supplement: Supplementary file 2 — Description of Additional Supplementary Files [file 41467_2022_28809_MOESM2_ESM.pdf]

Description of additional supplementary files

The Arginine Methyltransferase PRMT7 Promotes Extravasation of Monocytes resulting in tissue injury in COPD

Gizem Günes Günsel[1,†], Thomas M. Conlon[1,†], Aicha Jeridi[1,†], Rinho Kim[2], Zeynep Ertüz[1], Niklas J. Lang[1], Meshal Ansari[1,3], Mariia Novikova[4,5,6], Dongsheng Jiang[1], Maximilian Strunz[1], Mariia Gaianova[1], Christine Hollauer[1], Christina Gabriel[1], Ilias Angelidis[1], Sebastian Doll[3], Jeanine C. Pestoni[1], Stephanie L. Edelmann[7], Marlene Sophia Kohlhepp[8], Adrien Guillot[8], Kevin Bassler[9,10], Hannelore P. Van Eeckhoutte[11], Özgecan Kayalar[12], Nur Konyalilar[12], Tamara Kanashova[13], Sophie Rodius[14], Carolina Ballester-López[1], Carlos M. Genes Robles[2], Natalia Smirnova[1,15], Markus Rehberg[1], Charu Agarwal[1], Ioanna Krikki[1], Benoit Piavaux[16], Stijn E. Verleden[17,18], Bart Vanaudenaerde[17], Melanie Königshoff[1,19], Gunnar Dittmar[14,20], Ken R. Bracke[11], Joachim L. Schultze[9,21], Henrik Watz[22], Oliver Eickelberg[19], Tobias Stoeger[1], Gerald Burgstaller[1], Frank Tacke[8], Vigo Heissmeyer[7,23], Yuval Rinkevich[1], Hasan Bayram[12], Herbert B. Schiller[1], Marcus Conrad[4,5], Robert Schneider[2], Ali Önder Yildirim[1, 12]*

Excel File of Supplementary Data File 1. Enrichment of GO Molecular Function activities in the lungs of COPD patients following GSEA of GSE76925.

Excel File of Supplementary Data File 2. N-methyltransferase gene list (GO:0008170) from the GSEA enrichment plot of array data from lung tissue of COPD patients v healthy smokers (GSE76925).

Excel File of Supplementary Data File 3. LFQ intensities following anti-MMA IP proteome analysis.

Excel File of Supplementary Data File 4. Peptides identified following anti-MMA IP proteome analysis.

Excel File of Supplementary Data File 5. Enriched KEGG pathways following InCroMAP analysis of the differentially pulled down proteins with less abundance in *Prmt7*^{null} compared to *Prmt7*$^{+/+}$ MHS cells.

Excel File of Supplementary Data File 6. Enrichment of cell death modalities in the lungs of COPD patients following GSEA of GSE47460.

Excel File of Supplementary Data File 7. Enrichment of cell death modalities in the lungs of mice exposed to chronic CS for 4m following GSEA of GSE125521.

MP4 file of Supplementary Movie 1. Live cell imaging of PI uptake by MLE12 cells treated with conditioned medium from pro-inflammatory macrophages over 24h.

MP4 file of Supplementary Movie 2. Live cell imaging of PI uptake by MLE12 cells treated with RSL3 over 24h.

MP4 file of Supplementary Movie 3. Live cell imaging of PI uptake by MLE12 cells treated with conditioned medium from anti-inflammatory macrophages over 24h.

MP4 file of Supplementary Movie 4. Live cell imaging of PI uptake by MLE12 cells treated with conditioned medium from control macrophages over 24h.
