# Supplementary material for: The arginine methyltransferase PRMT7 promotes extravasation of monocytes resulting in tissue injury in COPD
Source: Nat Commun. 2022 Mar 14;13:1303. doi: 10.1038/s41467-022-28809-4 (PMC8921220; doi:10.1038/s41467-022-28809-4)
Supplement: Supplementary file 14 — Reporting Summary [file 41467_2022_28809_MOESM14_ESM.pdf]

nature research

Corresponding author(s): Ali Önder Yildirim

Last updated by author(s): Jan 12, 2022

Reporting Summary

Nature Research wishes to improve the reproducibility of the work that we publish. This form provides structure for consistency and transparency in reporting. For further information on Nature Research policies, see our Editorial Policies and the Editorial Policy Checklist.

Statistics

For all statistical analyses, confirm that the following items are present in the figure legend, table legend, main text, or Methods section.

n/a	Confirmed	
☐	☒	The exact sample size (n) for each experimental group/condition, given as a discrete number and unit of measurement
☐	☒	A statement on whether measurements were taken from distinct samples or whether the same sample was measured repeatedly
☐	☒	The statistical test(s) used AND whether they are one- or two-sided<br>*Only common tests should be described solely by name; describe more complex techniques in the Methods section.*
☒	☐	A description of all covariates tested
☐	☒	A description of any assumptions or corrections, such as tests of normality and adjustment for multiple comparisons
☐	☒	A full description of the statistical parameters including central tendency (e.g. means) or other basic estimates (e.g. regression coefficient) AND variation (e.g. standard deviation) or associated estimates of uncertainty (e.g. confidence intervals)
☐	☒	For null hypothesis testing, the test statistic (e.g. F, t, r) with confidence intervals, effect sizes, degrees of freedom and P value noted<br>*Give P values as exact values whenever suitable.*
☒	☐	For Bayesian analysis, information on the choice of priors and Markov chain Monte Carlo settings
☒	☐	For hierarchical and complex designs, identification of the appropriate level for tests and full reporting of outcomes
☐	☒	Estimates of effect sizes (e.g. Cohen's d, Pearson's r), indicating how they were calculated

Our web collection on statistics for biologists contains articles on many of the points above.

Software and code

Policy information about availability of computer code

Data collection	Respiratory function was assessed using a forced pulmonary maneuver system (Buxco Research Company, Data Sciences International) running FinePointe Software (version 6, Data Sciences International) and gases for diffusion capacity using a 3000 Micro GC Gas Analyser (Infinicon) running EZ IQ software v3.3.2 (Infinicon).<br>Single cell RNA-sequencing was carried out using the Drop-seq pipeline v2.3.0 (https://github.com/broadinstitute/Drop-seq) and STAR (v2.5.3a) for the alignment of reads to the mm10 reference genome.<br>Flow cytometry data was acquired using FACSDiva software v6.1.3 ran on a BD FACSCanto II.<br>qPCR data was obtained using a StepOnePlus™ 96 well Real-Time PCR System running StepOne software v2.3 (Applied Biosystems).<br>Western blot bands were detected and quantified using the Chemidoc XRS system+ running ImageLab v5.2.1 software (Bio-Rad).<br>Fluorescence images were captured using Axioimager with an M2 microscope (Carl Zeiss) driven by Zen 2.3 ("blue version") software (Carl Zeiss).<br>Histology images were aquired using Zeiss Mirax Microimmaging slide scanner running MIRAXDESK v1.12.25.1 and Mirax viewer v1.12.22.0 software (Zeiss, 3D Histech).<br>Design-based stereology was used to analyze sections using an Olympus BX51 light microscope equipped with a computer-assisted stereological toolbox software Visopharm Integrator System (VIS) v6.0.0.1765 (newCAST, Visiopharm).<br>Wound closure was determined at 6h and 24h using AxioVision software V4.9.1.0 (Zeiss).<br>Multiplex immunofluorescence images were processed using FIJI and the FIJI plugin HyperStackReg V5.6.<br>Live cell imaging was performed using an Axio Observer Z1 imaging system (Visitron Systems, Puchheim, Germany) running the imaging software VisiView 4.0 (Visitron Systems, Puchheim, Germany).<br>PLA images were obtained using an LSM710 system (Carl Zeiss) driven by ZEN2009 (Zeiss) software, version 5.5.
Data analysis	Single cell RNA-sequencing downstream analysis was performed using the Scanpy package Version 0.7 (https://github.com/theislab/Scanpy). Graph based clustering (Louvain algorithm, resolution = 2.0) was used to annotate the myeloid cells. Cell fate probability mapping was undertaken utilizing the following computation tools: velocyto74 version (0.17), scVelo75 (version 0.2.3) and CellRank18 (version 1.2.0). The complete code for this analysis is reported in the GitHub repository (https://github.com/

theislab/2021_PRMT7_regulates_Monocyte_Extravasation).
ComplexHeatmap package (version: 2.2.0) and ggplot2 (https://ggplot2-book.org/; version: 3.3.0) was used to visualize gene expression of the human BALF immune cell scRNA-seq data.
The MaxQuant (https://www.biochem.mpg.de/6304115/maxquant) software (version 1.6.12.0) was used to process proteomics data.
InCroMAP software (http://www.ra.cs.uni-tuebingen.de/software/InCroMAP/downloads/index) Version 1.7.0 (University of Tübingen, Germany) was used to analyze for KEGG pathway enrichment.
GSEA software v4.0.1 from the Broad Institute (http://www.gsea-msigdb.org/gsea/index.jsp) was used to determine the enrichment of gene lists from the GO Molecular Function collection in transcriptomics data obtained from series matrix files downloaded from the NCBI GEO database.
GEO2R interactive web tool (https://www.ncbi.nlm.nih.gov/geo/info/geo2r.html) was used to analyse gene expression in data obtained from series matrix files downloaded from the NCBI GEO database.
Microsoft Excel v 14.0.7237.5000 (part of Microsoft Office Professional Plus 2010) for normalisation and analysis of downloaded series matrix files from the NCBI GEO database.
Heat maps of enriched pathways were generated by Genesis software (Release 1.7.7, Institute for Genomics and Bioinformatics, Graz University of Technology).
The GWAS Central database (https://www.gwascentral.org) was screened to identify SNPs within the PRMT7 locus and were analyzed in the GTEx Portal database (https://gtexportal.org) to identify if they were eQTL.
PRMT7 promoter analysis on publically available CHIP-Seq data was undertaken using the Cistrome Data Browser (http://cistrome.org).
ATAC Seq analysis was undertaken by the company Active Motif and utilized the BWA algorithm to map paired-end 42 bp sequencing reads (PE42) to the genome and genomic regions with high levels of transposition/tagging events were determined using the MACS2 peak calling algorithm.
The ATAC Seq Tracks were visualized in the UCSC Genome Browser (https://genome.ucsc.edu/).
The ATAC seq Track across the TSS of Prmt7 in WT cells was analysed by JASPAR (http://jaspar.genereg.net).
Flow cytometry data was analysed using FACSDiva v6.1.3 and FlowJo v8.4.1 software.
Multiplex immunofluorescence quantitation was performed using Ilastik (v1.3.3post3) and CellProfiler (4.1.3).
Quantification of PLA images was performed using ImageJ software (v1.50a).
Statistical analyses were conducted using GraphPad Prism 6 or 8.

For manuscripts utilizing custom algorithms or software that are central to the research but not yet described in published literature, software must be made available to editors and reviewers. We strongly encourage code deposition in a community repository (e.g. GitHub). See the Nature Research guidelines for submitting code & software for further information.

Data

Policy information about availability of data

All manuscripts must include a data availability statement. This statement should provide the following information, where applicable:

- Accession codes, unique identifiers, or web links for publicly available datasets
- A list of figures that have associated raw data
- A description of any restrictions on data availability

Source data are provided with this paper.
Series matrix files from the NCBI GEO database for GSE76925, GSE27597, GSE47460 and GSE125521 were down loaded (https://www.ncbi.nlm.nih.gov/geo/).
The GWAS Central database (https://www.gwascentral.org) was screened to identify SNPs within the PRMT7 locus.
Promoter analysis on publically available CHIP-Seq data was undertaken using the Cistrome Data Browser (http://cistrome.org).
GSEA data can be found in Supplementary Data Files 1, 2, 6 and 7.
Proteomics data can be found in Supplementary Data Files 3 and 4 and enriched KEGG pathways following InCroMAP analysis Supplementary Data File 5.
ATAC-Seq data was submitted to the NCBI Gene Expression Omnibus (GEO) database (GSE153666).
Single cell RNA-Seq data was submitted to the NCBI Gene Expression Omnibus (GEO) database (GSE185006).
Micro-array data of our chronic cigarette smoke exposed mice can be found at GSE125521.

All code used for data visualization and analysis of the scRNA-seq data can be found at:
https://github.com/theislab/2021_PRMT7_regulates_Monocyte_Extravasation.

Field-specific reporting

Please select the one below that is the best fit for your research. If you are not sure, read the appropriate sections before making your selection.

[x] Life sciences   [ ] Behavioural & social sciences   [ ] Ecological, evolutionary & environmental sciences

For a reference copy of the document with all sections, see nature.com/documents/nr-reporting-summary-flat.pdf

Life sciences study design

All studies must disclose on these points even when the disclosure is negative.

Sample size	No statistical methods were used to predetermine sample size. Sample sizes were chosen based upon similar studies from the literature see Conlon et al. 2020 Nature, PMID 33149305, https://doi.org/10.1038/s41586-020-2882-8, and sufficient to detect differences between groups. All sample sizes for every group can be found in the figure legends.
Data exclusions	In all experiments values measured met QC criteria (eg. effective staining or gene expression), which was predetermined.
Replication	All findings were reliably reproduced, with group sizes and the number of independent repeats described in the figure legends.

Randomization	Mice were randomly assigned into experimental groups. Human lung tissue from explanted lungs of transplant patients was taken based upon a clinical diagnosis of COPD see Supplementary Table 1 and 2, for controls random unused donor lungs were collected from lungs declined for transplant after second opinion inspection because of kidney tumor, logistics or presence of microthrombi and randomized for processing and analysis. Blood donors for PBMCs and isolation of monocytes were randomly recruited among non-smokers and smokers working at Koç University Hospital and randomly assigned into wells. For in vitro cell culture experiments wells were randomly assigned to experimental groups.
Blinding	Quantitative morphometry on lung sections was undertaken by readers blinded to the study groups. Blinding was not carried out for other experiments to verify that each experiment contained all groups and appropriate controls, plus sample collection and processing of non-clinical samples were carried out by the same researchers.

Reporting for specific materials, systems and methods

We require information from authors about some types of materials, experimental systems and methods used in many studies. Here, indicate whether each material, system or method listed is relevant to your study. If you are not sure if a list item applies to your research, read the appropriate section before selecting a response.

Materials & experimental systems

n/a	Involved in the study
☐	☒ Antibodies
☐	☒ Eukaryotic cell lines
☒	☐ Palaeontology and archaeology
☐	☒ Animals and other organisms
☐	☒ Human research participants
☒	☐ Clinical data
☒	☐ Dual use research of concern

Methods

n/a	Involved in the study
☒	☐ ChIP-seq
☐	☒ Flow cytometry
☒	☐ MRI-based neuroimaging

Antibodies

Antibodies used

Immunohistochemistry:
Rabbit anti-Galectin 3, polyclonal, Cat. No. sc-20157, Santa Cruz
Rabbit anti-CD68, polyclonal, Cat. No. ab125212, Abcam
Mouse anti-ACSL4, clone F-4, Cat. No. sc-365230, Santa Cruz
Mouse anti-ALOX5, clone 33, Cat.No. sc-136195, Santa Cruz
Rabbit anti-Pro-SPC, polyclonal, Cat. No. AB3786, Sigma-Aldrich
Rabbit-on-Rodent AP-Polymer, Cat. No. RMR625H, Biocare Medical
Rabbit-on-Rodent HRP-Polymer, Cat. No. RMR622H, Biocare Medical
Mouse-on-Mouse AP-Polymer, Cat. No. MM624H, Biocare Medical

Immunofluorescence:
Mouse anti-Galectin 3, clone B2C10, Cat. No. sc-32790, Santa Cruz
Rabbit anti-PRMT7, polyclonal, Cat. No. sc-98882, Santa Cruz
Rabbit anti-5 Lipoxygenase/5-LO, clone EP6072(2), Cat. No. ab169755, Abcam
Rabbit anti-Myeloperoxidase, clone EPR20257, Cat. No. ab208670, Abcam
Rabbit anti-IBA1, polyclonal, Cat. No. 100369–764, VWR
Rabbit anti-iNOS, clone SP126, Cat. No. ab239990, Abcam
Rabbit anti-CD206, polyclonal, Cat. No. ab64693, Abcam
anti-mouse Alexa Fluor 488, Cat. No. A28175, Life Technologies
anti-rabbit Alexa Fluor 568, Cat. No. A-11011, Life Technologies
anti-Rabbit 555, Cat. No. 4413S, Cell Signaling
anti-Rabbit 647, Cat. No. 4414S, Cell Signaling

Western Blotting:
Goat anti-PRMT7, polyclonal, Cat.No. NBP2-26135, Novus Biologicals
Rabbit anti-PRMT6, clone D5A2N, Cat. No. 14641, Cell Signaling Technology
Rabbit anti-mono-methylated arginine, MultiMab mono-clonal mix, Cat. No. 8015, Cell Signaling Technology
Goat anti-T1-alpha, polyclonal, Cat. No. AF3244, R&D Systems
Rabbit anti-VCAM-1, clone EPR5047, Cat.No. ab134047, Abcam
Rabbit anti-RAP1A/B, clone EPR14814, Cat.No. ab187659, Abcam
Rabbit anti-phosphorylated ERK1/2, clone D13.14.4E, Cat.No.4370, Cell Signaling Technology
Rabbit anti-total ERK1/2, clone 137F5, Cat.No. 4695, Cell Signalling Technology
Rabbit anti-phosphorylated p38, clone 12F8, Cat.No. 4631, Cell Signalling Technology
Rabbit anti-H3R2me1, clone EPR17704, Cat.No. ab176844, Abcam
Rabbit anti-H3R2me2, clone 20.2, Cat.No. 04-808, Merck Millipore
Rabbit anti-Histone H3, polyclonal, Cat. No. ab1791, Abcam
Mouse anti-ACSL4, clone A-5, Cat.No. sc-271800, Santa Cruz
Mouse anti-ALOX5, clone 33, Cat.No. sc-136195, Santa Cruz
Rabbit anti-GPX4, clone EPNCIR144, Cat.No. ab125066, Abcam
beta-actin, clone AC-15, Cat. No. A3854, Sigma-Aldrich

Goat anti-Rabbit IgG H&L (HRP), Cat. No. ab6721, Abcam
Rabbit anti-Goat IgG-HRP, Cat. No. sc-2768, Santa Cruz
Sheep anti-Mouse IgG-HRP, Cat. No. NA931VS, Amersham,GE healthcare Life Sciences

Flowcytometry:
purified anti-mouse CD16/CD32, clone 93, Cat. No. 14-0161-82, eBioscience/ThermoFisher
VioGreen-conjugated anti-CD45, clone 30F11, Cat. No. 130-102-412, Miltenyi Biotec
PerCP-Vio700-conjugated anti-F4/80, clone REA126, Cat. No. 130-102-422, Miltenyi Biotec
PE-Vio770-conjugated anti-F4/80, clone REA126, Cat. No. 130-118-459, Miltenyi Biotec
PE-conjugated anti-CD11b, clone M1/70.15.11.5, Cat. No. 130-091-240, Miltenyi Biotec
PE-conjugated anti-CD11b, clone: REA592, Cat. No. 130-113-806, Miltenyi Biotec
APC-conjugated anti-CD11c, clone N418, Cat. No. 130-102-493, Miltenyi Biotec
APC-conjugated anti-CD11c, clone: REA754, Cat. No. 130-110-839, Miltenyi Biotec
APC-Vio770-conjugated anti-CD80, clone REA983, Cat. No. 130-116-463, Miltenyi Biotec
PerCP-Vio700-conjugated anti-CD86, clone PO3.3, Cat. No. 130-105-137, Miltenyi Biotec
FITC-conjugated anti-MHC class II, clone REA813, Cat. No. 130-112-229, Miltenyi Biotec
VioGreen-conjugated anti-CD45.2, clone: 104-2, Cat. No. 130-102-312, Miltenyi Biotec
FITC-conjugated anti-CD45.1, clone: A20, Cat. No. 130-124-211, Miltenyi Biotec
VioBlue-conjugated anti-Ly6g, clone: REA526, Cat. No. 130-119-986, Mitenyi Biotec
APC-conjugated anti-CD192 (CCR2), clone: REA538, Cat. No. 130-119-658, Miltenyi Biotec
PE-Vio770-conjugated anti-Ly6c, clone: REA796, Cat. No. 130-111-780, Miltenyi Biotec
PE-conjugated anti-CCR2, clone 475301, Cat. No. FAB5538P, R&D Systems
FITC-conjugated anti-CD11a, clone M17/4, Cat. No. 11-0111-82, eBioscience/ThermoFisher

MMA IP:
Rabbit anti-mono-methylated arginine, MultiMab mono-clonal mix, Cat. No. 8015, Cell Signaling Technology

ChIP-qPCR:
Rabbit anti-H3R2me1, clone EPR17704, Cat. No. ab176844, Abcam
Rabbit anti-H3R2me2s, polyclonal, Cat. No. ABE460, Millipore

PLA assay:
Mouse anti-RAP1A/B, clone 1D2-1C64, Cat. No. ab175329, Abcam
Rabbit anti-mono-methylated arginine, MultiMab mono-clonal mix, Cat. No. 8015, Cell Signaling Technology
Mouse anti-Histone 3, ChIP grade, Cat. No. ab195277, Abcam

Validation

Antibodies were titred out based on recommendations by the respective manufactures and validated before use in the study in-house and in the publications below. For immunohistochemistry secondary only controls were used through out the study.

Immunohistochemistry:
Rabbit anti-Galectin 3, polyclonal, Cat. No. sc-20157, Santa Cruz – PMID:32973111
Rabbit anti-CD68, polyclonal, Cat. No. ab125212, Abcam – PMID:33441701 and PMID:25153994
Mouse anti-ACSL4, clone F-4, Cat. No. sc-365230, Santa Cruz – PMID:29450800
Mouse anti-ALOX5, clone 33, Cat.No. sc-136195, Santa Cruz - PMID:34599216
Rabbit anti-Pro-SPC, polyclonal, Cat. No. AB3786, Sigma-Aldrich – PMID:23637738

Immunofluorescence:
Mouse anti-Galectin 3, clone B2C10, Cat. No. sc-32790, Santa Cruz – PMID31060902
Rabbit anti-PRMT7, polyclonal, Cat. No. sc-98882, Santa Cruz – PMID:15044439
Rabbit anti-5 Lipoxygenase/5-LO, clone EP6072(2), Cat. No. ab169755, Abcam – PMID:30410359
Rabbit anti-Myeloperoxidase, clone EPR20257, Cat. No. ab208670, Abcam – PMID:30419963
Rabbit anti-IBA1, polyclonal, Cat. No. 100369–764, VWR - PMID: 32607882
Rabbit anti-iNOS, clone SP126, Cat. No. ab239990, Abcam - PMID: 31426846
Rabbit anti-CD206, polyclonal, Cat. No. ab64693, Abcam – PMID:33402181

Western Blotting:
Goat anti-PRMT7, polyclonal, Cat.No. NBP2-26135, Novus Biologicals – PMID: 15494416
Rabbit anti-PRMT6, clone D5A2N, Cat. No. 14641, Cell Signaling Technology – PMID:34330913 and PMID:34088896
Rabbit anti-mono-methylated arginine, MultiMab mono-clonal mix, Cat. No. 8015, Cell Signaling Technology – PMID:34145242
Goat anti-T1-alpha, polyclonal, Cat. No. AF3244, R&D Systems – PMID:32931478
Rabbit anti-VCAM-1, clone EPR5047, Cat.No. ab134047, Abcam – PMID:33692398
Rabbit anti-RAP1A/B, clone EPR14814, Cat.No. ab187659, Abcam - Manufacturer tested suitable for Western Blot.
Rabbit anti-phosphorylated ERK1/2, clone D13.14.4E, Cat.No.4370, Cell Signaling Technology – PMID:33853482
Rabbit anti-total ERK1/2, clone 137F5, Cat.No. 4695, Cell Signalling Technology – PMID: 33866927
Rabbit anti-phosphorylated p38, clone 12F8, Cat.No. 4631, Cell Signalling Technology – PMID:34535669
Rabbit anti-H3R2me1, clone EPR17704, Cat.No. ab176844, Abcam - Tested by the manufacturer in a peptide array against 501 different modified and unmodified peptides for specificity.
Rabbit anti-H3R2me2, clone 20.2, Cat.No. 04-808, Merck Millipore - PMID: 21131980
Rabbit anti-Histone H3, polyclonal, Cat. No. ab1791, Abcam - PMID: 19270702
Mouse anti-ACSL4, clone A-5, Cat.No. sc-271800, Santa Cruz – PMID: 34040532
Mouse anti-ALOX5, clone 33, Cat.No. sc-136195, Santa Cruz - PMID:33747208
Rabbit anti-GPX4, clone EPNCIR144, Cat.No. ab125066, Abcam – PMID:33431801
beta-actin, clone AC-15, Cat. No. A3854, Sigma-Aldrich – PMID:31295865

Flow cytometry:
VioGreen-conjugated anti-CD45, clone 30F11, Cat. No. 130-102-412, Miltenyi Biotec – PMID:32855420 and PMID:32433499

PerCP-Vio700-conjugated anti-F4/80, clone REA126, Cat. No. 130-102-422, Miltenyi Biotec – PMID: 21952799 and PMID: 29674392
PE-Vio770-conjugated anti-F4/80, clone REA126, Cat. No. 130-118-459, Miltenyi Biotec - PMID: 21952799 and PMID: 29674392
PE-conjugated anti-CD11b, clone M1/70.15.11.5, Cat. No. 130-091-240, Miltenyi Biotec - PMID: 29674392 and PMID:30683126
PE-conjugated anti-CD11b, clone: REA592, Cat. No. 130-113-806, Miltenyi Biotec - PMID: 32080625
APC-conjugated anti-CD11c, clone N418, Cat. No. 130-119-802, Miltenyi Biotec - PMID: 29674392 and PMID: 31060902
APC-conjugated anti-CD11c, clone: REA754, Cat. No. 130-110-839, Miltenyi Biotec - Epitope competition assay undertaken by manufacturer.
APC-Vio770-conjugated anti-CD80, clone REA983, Cat. No. 130-116-463, Miltenyi Biotec - Ab was compared to commercially available hybridoma clones in flow cytometry assays by manufacturer.
PerCP-Vio700-conjugated anti-CD86, clone PO3.3, Cat. No. 130-105-137, Miltenyi Biotec - PMID: 16709832
FITC-conjugated anti-MHC class II, clone REA813, Cat. No. 130-112-229, Miltenyi Biotec - PMID: 29674392 and PMID: 31060902
VioGreen-conjugated anti-CD45.2, clone: 104-2, Cat. No. 130-102-312, Miltenyi Biotec - PMID: 31216482
FITC-conjugated anti-CD45.1, clone: A20, Cat. No. 130-124-211, Miltenyi Biotec - PMID: 17398124
VioBlue-conjugated anti-Ly6g, clone: REA526, Cat. No. 130-119-986, Mitenyi Biotec - PMID: 34745127
APC-conjugated anti-CD192 (CCR2), clone: REA538, Cat. No. 130-119-658, Miltenyi Biotec - PMID: 32234475
PE-Vio770-conjugated anti-Ly6c, clone: REA796, Cat. No. 130-111-780, Miltenyi Biotec - PMID: 32234475
PE-conjugated anti-CCR2, clone 475301, Cat. No. FAB5538P, R&D Systems – PMID:34091064 and PMID:32668709
FITC-conjugated anti-CD11a, clone M17/4, Cat. No. 11-0111-82, eBioscience/ThermoFisher – PMID:29166588

MMA IP:
Rabbit anti-mono-methylated arginine, MultiMab mono-clonal mix, Cat. No. 8015, Cell Signaling Technology - Cited over 25 times including PMID: 32409666

ChIP-qPCR:
Rabbit anti-H3R2me1, clone EPR17704, Cat. No. ab176844, Abcam - Tested by the manufacturer in a peptide array against 501 different modified and unmodified peptides for specificity and validated in ChIP.
Rabbit anti-H3R2me2s, polyclonal, Cat. No. ABE460, Millipore - PMID: 22231400

PLA assay:
Mouse anti-RAP1A/B, clone 1D2-1C64, Cat. No. ab175329, Abcam - PMID: 28183814
Rabbit anti-mono-methylated arginine, MultiMab mono-clonal mix, Cat. No. 8015, Cell Signaling Technology - PMID:33782401
Mouse anti-Histone 3, ChIP grade, Cat. No. ab195277, Abcam - PMID:33349669 and PMID:31661552

Eukaryotic cell lines

Policy information about cell lines

Cell line source(s)	MHS, #CRL-2019, ATCC<br>MLE12, #CRL-2110, ATCC<br>RAW 264.7, #TIB-71, ATCC<br>SVEC4-10, #CRL-2181, ATCC<br>HEK293T, #CRL-3216, ATCC
Authentication	The cell lines were not authenticated.
Mycoplasma contamination	All cell lines were routinely tested, and tested negative for Mycoplasma.
Commonly misidentified lines<br>(See ICLAC register)	No commonly misidentified cell lines used in the study.

Animals and other organisms

Policy information about studies involving animals; ARRIVE guidelines recommended for reporting animal research

Laboratory animals	C57BL/6N-Tyrc-Brd Prmt7tm1a(EUCOMM)Wtsi/WtsiCnbc mice were obtained from the European Conditional Mouse Mutagenesis Program (EUCOMM), The Wellcome Trust Sanger Institute, Cambridge, UK. Heterozygous mice carrying a single copy of the insertion (referred to as Prmt7+/- mice), were bred with wild-type siblings to maintain the colony. 8 to 10 week old Prmt7+/- mice and their wild-type littermate controls (males and females) were used in all experiments.<br>C57BL/6N-Tyrc-Brd Prmt7tm1a(EUCOMM)Wtsi/WtsiCnbc mice were further crossed with FLPe expressing mice, B6.129S4-Gt(ROSA)26Sortm1(FLP1)Dym/RainJ (The Jackson Laboratory), to delete the FRT flanked insertion cassette, leaving a Loxp flanked exon 3 of Prmt7. FLPe expression was bred out of the mice, and the mice were then crossed with B6.129P2-Lyz2tml(cre)Ifo/J (The Jackson Laboratory) to generate Lyz2.Cre Prmt7flox/flox mice. 8- to 10-week-old Lyz2- Cre Prmt7flox/flox mice and their Prmt7flox/flox littermate controls not expressing Cre (males and females) were used in all experiments.<br>For bone marrow chimeras, female 8-10 week old C57BL/6 congenic mice, B6.SJL-Ptprca Pepcb/BoyJ (CD45.1) obtained from The Jackson Laboratory and bred in house were used as recipients.<br>All mice were housed in rooms maintained at a constant temperature of 20-24°C and 45-65% humidity with a 12 hour light cycle. Animals were allowed food and water ad libitum.
Wild animals	No wild animals were used in the study.
Field-collected samples	No field-collected samples were used in the study.
Ethics oversight	All animal experiments were approved by the ethics committee for animal welfare of the local government for the administrative

Ethics oversight | region of Upper Bavaria (Regierungspräsidium Oberbayern) and were conducted under strict governmental and international guidelines in accordance with EU Directive 2010/63/EU.

Note that full information on the approval of the study protocol must also be provided in the manuscript.

Human research participants

Policy information about studies involving human research participants

Population characteristics	Lung core samples from explanted lungs of COPD patients undergoing lung transplantation were used. Patient demographics are highlighted in Supplementary Table 1. For controls, unused donor lungs were collected. Lungs were declined for various reasons (kidney tumor, logistics, presence of microthrombi).<br>Further lung resection specimens were obtained from a 118 participants, of which 106 were from surgery for solitary pulmonary tumours (Ghent University Hospital, Ghent, Belgium) and 12 were from explant lungs of end-stage COPD patients undergoing lung transplantation (University Hospital Gasthuisberg, Leuven, Belgium). Lung tissue from the resection specimen was harvested by a pathologist at maximum distance from the tumour. Participant characteristics are highlighted in Supplementary Table 2.<br>Human primary monocytes were isolated from the PBMC of healthy study volunteers who were active or non-smokers. Participant characteristics are highlighted in Supplementary Table 3.
Recruitment	Blood donors for PBMCs and isolation of monocytes were randomly recruited among non-smokers and smokers working at Koç University Hospital.
Ethics oversight	Lung core samples were used following informed written consent and ethical approval of the University of Leuven Institutional Review Board (ML6385). Unused donor lungs were collected under existing Belgian law which allows the use of declined donor lungs for research after second opinion inspection.<br>Lung resection specimens from a 118 participants were obtained following informed written consent and approval by the medical ethical committees of the Ghent University Hospital (2011/0114; 2016/0132; 2019/0537) and the University Hospital Gasthuisberg, Leuven (S51577).<br>The study isolating human monocytes was approved by the Ethics Committee of Koc University, and informed written consents were taken from study volunteers.

Note that full information on the approval of the study protocol must also be provided in the manuscript.

Flow Cytometry

Plots

Confirm that:

☒ The axis labels state the marker and fluorochrome used (e.g. CD4-FITC).

☒ The axis scales are clearly visible. Include numbers along axes only for bottom left plot of group (a 'group' is an analysis of identical markers).

☒ All plots are contour plots with outliers or pseudocolor plots.

☒ A numerical value for number of cells or percentage (with statistics) is provided.

Methodology

Sample preparation	Single-cell suspensions of right lung tissue were generated. Lung homogenization was performed via enzymatic digestion and mechanical dissociation steps using the lung dissociation kit and gentleMACS Dissociator from Miltenyi Biotec. 10^6 cells were blocked with purified anti-mouse CD16/CD32 (clone 93, eBioscience, ThermoFisher Scientific) before incubating for 30min on ice in the dark with titrated antibody master mix and washed.<br>Cell lines and bone marrow derived macrophages were trypsinized, washed and single cell suspensions blocked as above.<br>Monocytes were isolated from the bone marrow of mice using the Monocyte Isolation Kit (BM) from Miltenyi Biotec, and single cell suspensions blocked as above.
Instrument	BD FACSCanto II flow cytometer (BD Biosciences).
Software	BD FACSDiva v6.1.3 and FlowJo v7.2.1 software.
Cell population abundance	No sorting undertaken.
Gating strategy	Lung cells were gated based on size (FSC v SSC), doublets excluded, CD45+ cells selected, the F480+ fraction gated, and then analysed for CD11b and CD11c expression.<br>Cell lines were gated on size (FSC v SSC), and then analysed for CD11a (ITGAL) and CD11b (ITGAM) expression.<br>Isolated monocytes were gated on size (FSC v SSC), doublets excluded, and CCR2 MFI determined.<br>Bone marrow derived macrophages were gated on size (FSC v SSC), doublets excluded, CD45+ cells selected, the F480+ fraction gated, and then analysed for CD11b and CD11c expression.

☒ Tick this box to confirm that a figure exemplifying the gating strategy is provided in the Supplementary Information.
